# Long shared haplotypes identify the Southern Urals as a primary source for the 10th century Hungarians

**DOI:** 10.1016/j.cell.2025.09.002

**Published:** 2025-10-16

**Authors:** Balázs Gyuris, Leonid Vyazov, Attila Türk, Pavel Flegontov, Bea Szeifert, Péter Langó, Balázs Gusztáv Mende, Veronika Csáky, Andrey A. Chizhevskiy, Ilgizar R. Gazimzyanov, Aleksandr A. Khokhlov, Aleksandr G. Kolonskikh, Natalia P. Matveeva, Rida R. Ruslanova, Marina P. Rykun, Ayrat Sitdikov, Elizaveta V. Volkova, Sergei G. Botalov, Dmitriy G. Bugrov, Ivan V. Grudochko, Oleksii Komar, Alexander A. Krasnoperov, Olga E. Poshekhonova, Irina Chikunova, Flarit Sungatov, Dmitrii A. Stashenkov, Sergei Zubov, Alexander S. Zelenkov, Harald Ringbauer, Olivia Cheronet, Ron Pinhasi, Ali Akbari, Nadin Rohland, Swapan Mallick, David Reich, Anna Szécsényi-Nagy

**Affiliations:** 1 Institute of Archaeogenomics, HUN-REN Research Centre for the Humanities; Budapest, Hungary; 2 Doctoral School of Biology, ELTE Eötvös Loránd University; Budapest, Hungary; 3 Department of Biology and Ecology, Faculty of Science, University of Ostrava; Ostrava, Czechia; 4 Department of Human Evolutionary Biology, Harvard University; Cambridge, MA, USA; 5 Department of Archaeology, Faculty of Humanities and Social Sciences, Pázmány Péter Catholic University; Budapest, Hungary; 6 Hungarian Prehistory Research group, HUN-REN Research Centre for the Humanities; Budapest, Hungary; 7 Institute of Archaeology, HUN-REN Research Centre for the Humanities, Hungarian Research Network (HUN-REN); Budapest, Hungary; 8 Institute of Archaeology of the Academy of Sciences of the Republic of Tatarstan; Kazan, Republic of Tatarstan, RusENAsia; 9 Samara State University of Social Sciences and Education; Samara, Russia; 10 R.G. Kuzeev Institute of Ethnological Studies, Ufa Federal Research Scientific Center of Russian Academy of Sciences; Ufa, Republic of Bashkortostan, Russia; 11 University of Tyumen; Tyumen, Russia; 12 National Museum of the Republic of Bashkortostan; Ufa, Republic of Bashkortostan, Russia; 13 National Research Tomsk State University; Tomsk, Russia; 14 Department of Archaeology, Kazan Federal University, Kazan, Republic of Tatarstan, Russia; 15 Institute of History and Archeology, Ural Branch of the Russian Academy of Sciences, Yekaterinburg, Russia; 16 South Ural Branch of the Institute of History and Archeology, Ural Branch of the Russian Academy of Sciences; Chelyabinsk, Russia; 17 South Ural State University, Chelyabinsk, Russia; 18 National Museum of Tatarstan Republic; Kazan, Republic of Tatarstan, Russia; 19 Institute of Archaeology, National Academy of Sciences of Ukraine; Kyiv, Ukraine; 20 Udmurt Institute of History, Language and Literature, Udmurt Federal Research Center, Ural Branch of the Russian Academy of Sciences; Izhevsk, Udmurt Republic, Russia; 21 Institute of the Problems of Northern Development, Tyumen Scientific Centre, Siberian Branch of the Russian Academy of Sciences; Tyumen, Russia; 22 Institute of History, Language and Literature, Ufa Federal Research Scientific Center of Russian Academy of Sciences; Ufa, Republic of Bashkortostan, Russia; 23 Samara Regional Museum of History and Local Lore named after P. V. Alabin; Samara, Russia; 24 Research Laboratory of Archeology, Samara National Research University; Samara, Russia; 25 Research Institute for Jochi Ulus studies, Astana, Kazakhstan; 26 Department of Archaeogenetics, Max Planck Institute for Evolutionary Anthropology; Leipzig, Germany; 27 Department of Evolutionary Anthropology, University of Vienna; Vienna, Austria; 28 Human Evolution and Archaeological Sciences Forschungsverbund, University of Vienna; Vienna, Austria; 29 Department of Genetics, Harvard Medical School; Boston, MA 02138, USA; 30 Broad Institute of MIT and Harvard; Cambridge, MA 02142, USA; 31 Howard Hughes Medical Institute; Boston, MA 02138, USA; 32 Lead Contact

## Abstract

The origins of the Early Medieval Magyars who appeared in the Carpathian Basin by the end of the 9th century CE remain incompletely understood. Previous archaeogenetic research identified the newcomers as migrants from the Eurasian steppe. However, genome-wide ancient DNA from putative source populations has not been available to test alternative theories of their precise source. We generated genome-wide ancient DNA data for 131 individuals from archaeological sites in the Uralic region in Northern Eurasia, which are candidates for the source based on historical, linguistic, and archaeological evidence. Our results tightly link the Magyars to people of the Early Medieval Karayakupovo archaeological horizon on both the European and Asian sides of the southern Urals. The ancestors of the people of the Karayakupovo archaeological horizon were established in the broader Urals by the Late Iron Age and their descendants persisted in the Volga-Kama region until at least the 14th century.

## eTOC/In Brief

Genome-wide ancient DNA data from Early Medieval populations across the broader Ural region and adjacent areas identifies the Southern Urals as the primary ancestral source of 10th-century Hungarians.

## Graphical Abstract

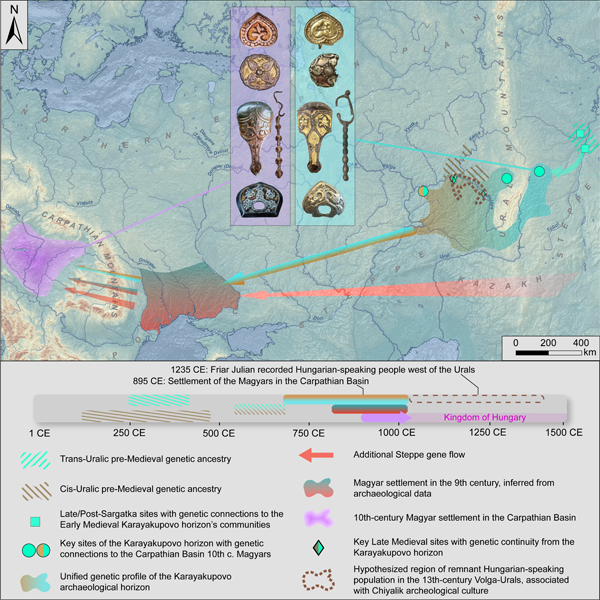

## Introduction

The Hungarians are the only Uralic-speaking ethnicity in Central Europe, with a history tracing back to the Early Medieval period, east of the Carpathian Basin. Their history became richly documented beginning with the Hungarian Conquest period (895–1000 CE), which introduced striking innovations in burial rites and artifact assemblages to the Carpathian Basin. These cultural transformations are commonly interpreted as signatures of the arrival of a tribal alliance from the Eurasian Steppe, known as the Early Medieval Magyars (EMM)(1–6). Chronicles and oral tradition trace the origin of these Magyars to an eastern homeland(1,2) and a significant body of archaeological and linguistic research(1,4,7–11) highlights the Cis- or Trans-Uralic regions as the leading candidate for their homeland. Over the past century, the reconstruction of early Magyars history has seen the emergence of diverse theories, as comprehensively reviewed by Zimonyi(12), all of which recognize the significance of the broader Volga-South Ural region in the ancestral formation process of the Magyars. The details of the migration speed and routes are more contentious. The Magyars likely encountered Turkic-speaking communities in both the Volga-Ural region and the North-Pontic steppe, based on material culture connections between these regions and the Carpathian Basin. The crossing of the Volga River by the Magyars in a westward direction has been estimated to have occurred between 460–830 CE(1,7,13–17), while their settlement areas in the Northwestern Pontic region are inferred to have commenced between 670–860 CE(7,16–22). Although more recent research supports a 9th century chronology(3,6), it is challenging to date the beginning of this migration and its intermediate steps. It also remains unclear where and how the language and community structure of the early Magyars were formed, as well as the roles that the Circum-Uralic populations played in their ethnogenesis and confederation.

Based on parallels in material culture with the 10th-century Carpathian Basin, archaeologists have attributed some burial sites located around the South Urals to Magyars(8). We hereafter introduce the term ‘Karayakupovo Horizon’ (KH) to cover the diversity of the burial traditions and artefactual assemblages of the Southern Urals, including the Cis- and Trans-Urals, dated to 750–1000 CE and associated with putative Early Medieval Magyars(8,9). East of the Urals, a reference cemetery of this horizon was excavated at Uyelgi, near Chelyabinsk(23). On the European side of the Urals, Bolshie Tigany in Tatarstan is a key site, and in the last decades, it was understood as a 9–10th century cemetery of Magyar groups that remained in the Volga-Urals(3,5,8,24–27). People attributed to the Karayakupovo Horizon lived in a multilingual and multiethnic context in the Circum-Uralic region, surrounded by Turkic, Finno-Permic, and Ugric-speaking people(28). Further evidence supporting the theory that Magyars settled in the Volga region during the Early Middle Ages are later reports of a Hungarian-speaking population in the Middle Volga and Lower Kama regions, from European travellers who visited an area known as *Magna Hungaria* in the 1230s(29). However, the survival of such communities has never been tested using ancient DNA data, the only direct way to verify population continuity and theories of ancestral origin.

Ancient DNA (aDNA) studies have generated large amounts of genetic data on ancient people of northern Eurasia which we co-analyze in this study along with our newly reported data(30–72). However, the Ural region from the Late Iron Age to Medieval times remained unstudied on the genome-wide level. *Csáky et al.*(73) and *Szeifert et al.*(74) provided insights into the connections between the 10th-11th century population of the Carpathian Basin and the Volga-Ural populations at the uniparental DNA level, while *Maróti et al.*(65) and *Gnecchi-Ruscone et al*.(61) generated genome-wide data for the Early Medieval Carpathian Basin itself. *Maróti et al*.(65) reported data from the 5th-10th centuries Carpathian Basin, showing that the Avars and Magyars represent distinct groups with East Eurasian genetic affinities. Based on their analyses, they argued that several sources were plausible for the immigrant 10th-century Magyars (named there as Conqueror Asia Core). This included modern Ugric-speaking Mansi proxy used in their canonical ancestry modeling, as well as groups descended from Huns/Xiongnu, and early and late Sarmatians. However, these sources do not align with prevailing linguistic and archaeological interpretations. Therefore, it is important to carry out tests with samples from the populations that archaeological evidence suggests are the most plausible proximate sources.

Here, we leverage the first genome-wide ancient DNA data from putative Ural source and adjacent populations of Early Medieval Magyars to understand their relationships to the new arrivals in the Carpathian Basin. We then examined the deeper population history of those Volga-Uralic groups (by using Late Bronze Age/Iron Age and Migration period reference populations) that showed especially strong connections to 10th-century Carpathian Basin Magyars to document the extent of genetic continuity from the Iron Age to Medieval times in the Volga-Urals.

## Results

**Figure 1.**
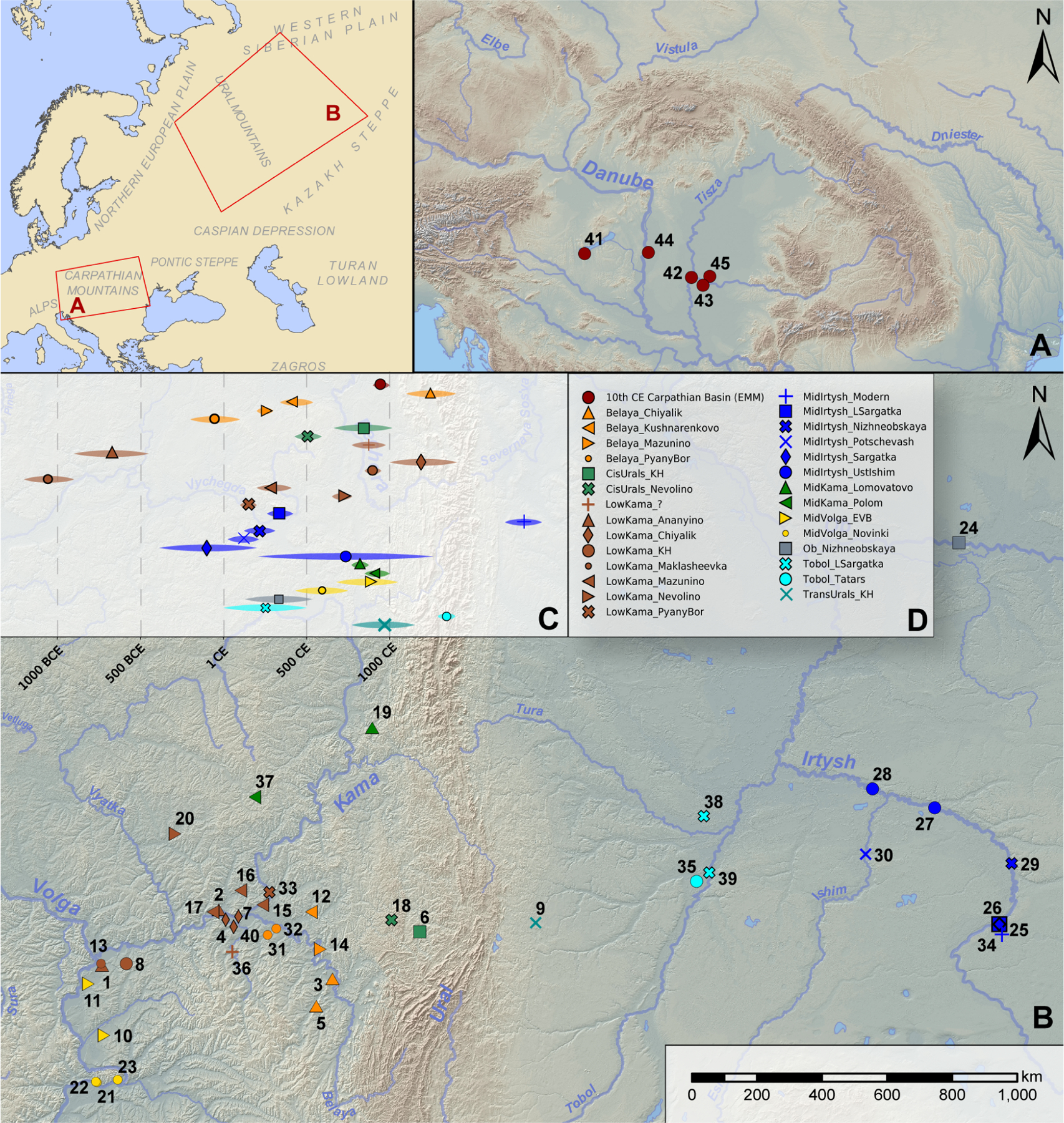
Locations and chronology of the studied burials. Archaeological sites in the Carpathian Basin (**A**) and in the Volga-Ural region (**B**) involved in this study, are colored according to ecoregions: 1: Izmeri-7; 2: Rysovo-1; 3: Gornovo; 4: Gulyukovo; 5: Novo-Khozyatovo; 6: Karanayevo; 7: Zuyevy-Klyuchi; 8: Bolshie-Tigany; 9: Uyelgi; 10: Mullovka; 11: Tankeyevka; 12: Bustanaevo; 13: Devichiy-Gorodok-4; 14: Birsk-2; 15: Boyarsky-Aray; 16: Dubrovsky; 17: Turaevo-1; 18: Bartym; 19: Bayanovo; 20: Sukhoy-Log; 21: Brusyany; 22: Malaya-Ryazan’; 23: Novinki-1; 24: Barsov-Gorodok; 25: Borovyanka-17; 26: Borovyanka-18; 27: Ivanov-Mys-1; 28: Panovo; 29: Ust-Tarsk; 30: Vikulovo; 31: Kipchakovo; 32: Starokirgizovo; 33: Tarasovo; 34: Bogdanovo-2; 35: Putilovo; 36: Mellyatamak-3; 37: Varni; 38: Ipkul; 39: Starolybaevo-4; 40: Ust-Menzelya; 41: Balatonújlak; 42: Szeged-Öthalom; 43: Kiszombor; 44: Harta-Freifelt; 45: Makó-Igási járandó. Groups defined in this study are listed in panel **D** and their chronology is given in **C**.

We used in-solution enrichment for more than 1.2 million single nucleotide polymorphisms (the “1240k” SNP capture panel(75)) to study the ancestry of 120 newly reported individuals from 40 archaeological sites in the Circum-Uralic area (see descriptions of relevant geography and sub-regions in the Data S1, summary of archeological and genetic context in Table S1A), dated from the Late Bronze Age (~1300–1000 BCE) to the Late Medieval period starting ca. 1400 CE (see Figure 1, and Data S1 for detailed archeological and genetic descriptions of the newly sampled burials). In addition, we present data for 11 newly reported individuals from the Carpathian Basin dated to the 10th century CE. For estimating genetic diversity and, in some cases, for modeling genetic origin, we grouped individuals by ecoregions/river basins and chronological periods(76); see SI, section I. for details. For brevity, these periods are labeled by prevailing cultural groups in the region, e.g., *Russia_Belaya_Chiyalik* (Fig. 1), but cultural attribution did not play a role in the grouping process with one exception (the Karayakupovo Horizon).

Recent methodological developments have made it possible to detect long shared autosomal haplotypes between pairs of ancient genomes(77,78), often termed identical-by-descent (IBD) segments(79). Previously, this method was only applicable to high-quality genomic data for modern populations(80,81). However, recent advances allow its application to ancient individuals as well, even if they have moderate fractions of their genome without high sequence coverage, leveraging the fact that human genetic variation is highly redundant so genotypes can be statistically imputed with high confidence from nearly incomplete genetic data(77). The IBD-sharing analysis is particularly useful for detecting distant relatives. We coupled this analysis with archaeogenetic methods relying on correlations of allele frequencies: PCA(82), *f*-statistics and derived methods(31,82–86), as well as *ADMIXTURE*(87).

Our research protocol included several stages. First, we utilized PCA, supervised *ADMIXTURE* analysis, and network graphs visualizing individuals linked by shared IBD segments (see Methods for further details), to obtain a broad overview of the dataset. In the second stage, we focused on IBD connections between the Volga-Ural region and the population of the 10–11th century Carpathian Basin. In the third stage, we explored the genetic history of the Medieval Volga-Uralic groups using *f*-statistics(31,82,85), which allow for formal tests of simple nonphylogenetic admixture models. To understand changes in population size and rates of close-kin marriages in this period, we explored runs of homozygosity (*hapROH*)(88).

### Genetic diversity in the Volga-Ural region

**Figure 2.**
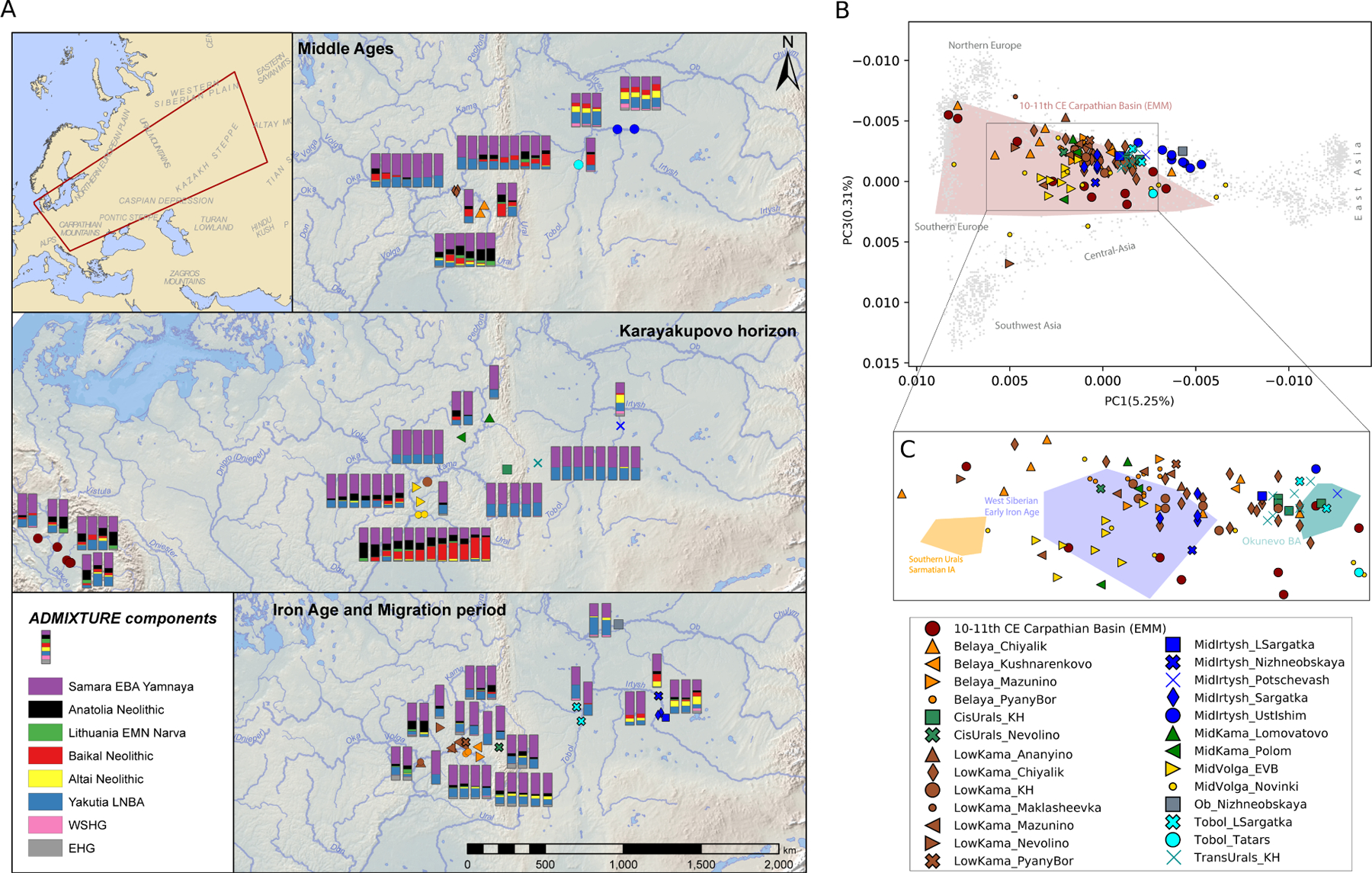
Principal component analysis and supervised *ADMIXTURE* analysis of the newly sequenced genomes. **A:** Supervised *ADMIXTURE* analysis (K=8) of the newly presented individuals, plotted on maps, which show their approximate origin and chronology. **B:** Eurasian-scale principal component analysis (PCA), with a projection of the newly sequenced individuals on modern genetic variation after Jeong et al.(50). The PC1 and PC3 dimensions are depicted with the newly presented genomes. Grey dots indicate modern Eurasian genomes on which the ancient samples were projected. Polygons (**C**) outline ancient reference sample distributions (without displaying individual points): Early Medieval Magyars (EMM) from the Carpathian Basin (red)(65), Early Iron Age Southern Urals (yellow)(49), Iron Age Western Siberia (blue)(56), and Bronze Age South Central Siberia (green)(38).

The Eurasian PCA in Fig. 2B reveals extraordinary genetic heterogeneity in the Early Medieval Volga-Ural region, with high variability in ancestry among individuals associated with certain regional and chronological groups. In the PC1/PC3 space (Fig. 2B), we observe an east-west genetic gradient from Northeast Asian (NEA) to Northwest Eurasian (NWE) genetic affinities. Most ecoregions of interest display high genetic diversity, with individuals from each region spreading over large sections of the gradient. Notably, most of the newly sequenced 10th-century individuals from the Carpathian Basin are positioned along the NWE-NEA and NWE-Eastern Asian (EA) clines, with only two of them demonstrating a Central European genomic profile. We also conducted a supervised *ADMIXTURE* analysis (Fig. 2A, Table S1D), utilizing eight Neolithic and Early Bronze Age populations as proxy ancestry sources for the clustering algorithm. In the selection for the ancestral sources, we aimed to reflect the Neolithic/Bronze Age variation of North Eurasia (for details see Methods). Our findings reveal a widespread yet varying presence of Early Bronze Age Yamnaya-related ancestry across the region. This persistent Yamnaya-related ancestry(30), contrasted with the fluctuating levels of other ancestries, such as the Yakutia LNBA, Baikal Neolithic or Altai Neolithic(68), and reflects a patchwork of local genetic influences in the region.

**Figure 3.**
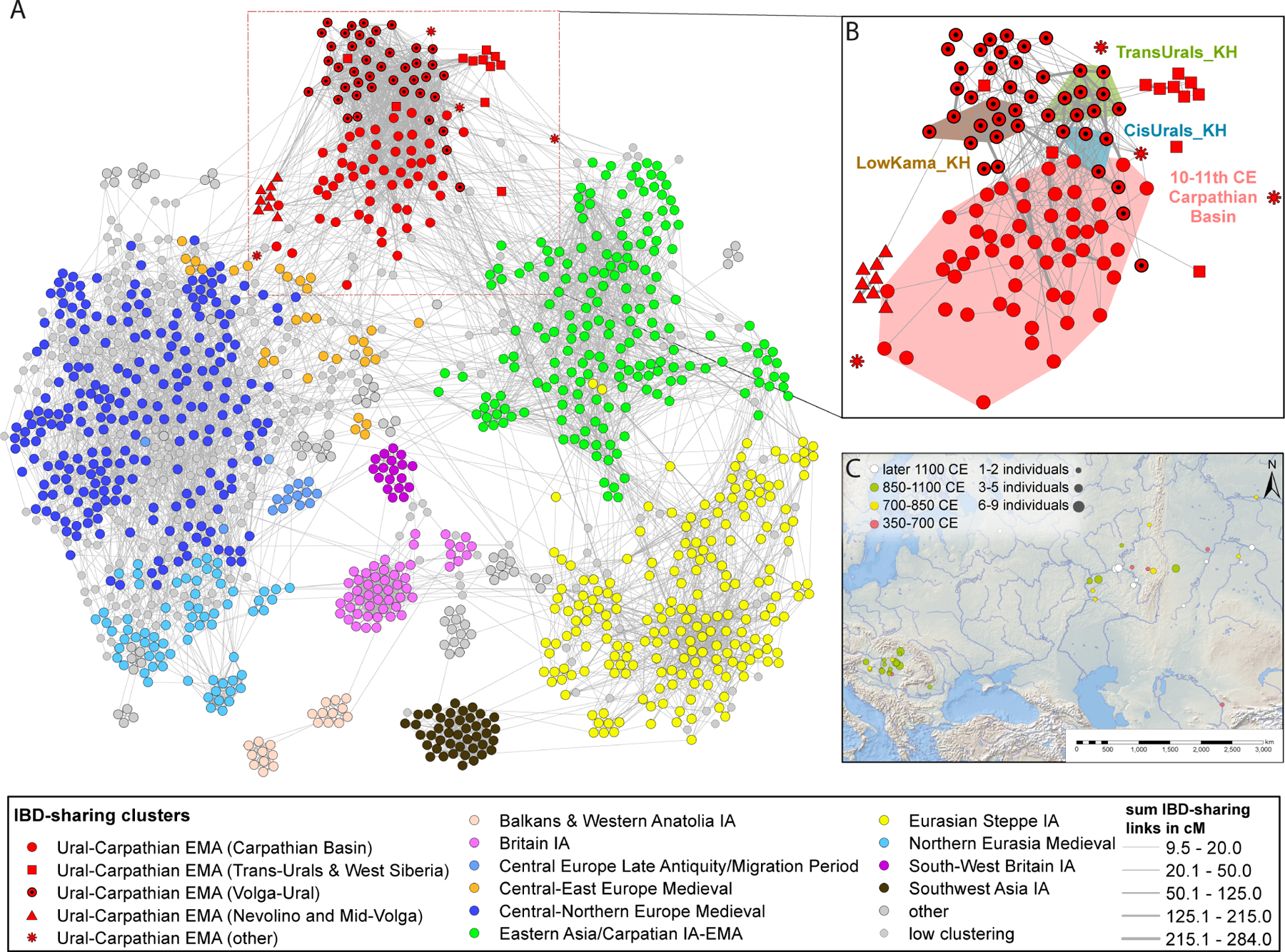
IBD network and visualization of the major IBD connections on a map. **A:** A network graph of IBD sharing visualizing clusters of distant relatives for 1,332 ancient Eurasian individuals from the Iron Age to the Medieval Period (MultiGravity ForceAtlas 2, a force-directed layout algorithm(87) was used, with additional Leiden algorithm(88) for clustering); **B:** Close-up of the *Urals-Carpathian EMA* cluster within the network, highlighting the KH and 10–11th century Carpathian Basin individuals in the cluster; **C:** Geographical distribution of the individuals from the *Urals-Carpathian EMA* IBD-sharing community.

We applied genotype imputation(77), and inferred IBD segments using the approach described in(78), and constructed a network graph(89) connecting individuals with shared IBD segments on a total of 1,332 individuals (for details see Table S1B,C), comprising published data for 1,231 individuals from Asia and Europe and 101 individuals presented in this study (Fig. 3A; see Fig. S1 for time-oriented, non-filtered, and PC-projected versions of this network). The graph’s edges were weighted based on the length of the most substantial IBD segment shared by two individuals (nodes). To de-noise the graph, we restricted the analysis to individuals connected by at least one 9 cM segment, not separated in time by more than 600 years, and focused on the largest interconnected sub-graph. Details of the de-noising, visualization, and clustering approach are described in the Methods. Twelve newly reported Iron Age individuals formed a cluster (with many previously published individuals) in the IBD network that we labeled *Eurasian steppe Iron Age (IA)* in Fig. 3A (clusters were inferred with the Leiden community detection algorithm(90); we refer to them as “IBD-sharing communities’’ or simply “IBD clusters”). A total of 118 Early Medieval individuals from both the Volga-Ural region and Carpathian Basin formed another cluster (Fig. 3C), labeled as *Urals-Carpathian Early Middle Ages (EMA)* in Fig. 3A. To discern and quantify the underlying differences among the identified network clusters, we analyzed network topology, similar to that described by Gnecchi-Ruscone et al.(91), focusing on metrics such as degree centrality (number of links held by a given node) or module strength measured based on summarized IBD-sharing between individuals (see Methods). The *Urals-Carpathian EMA* cluster’s average clustering coefficient was close to the mean of the other clusters. At the same time, its relatively high within-module (*k*w) and low between-module (*k*B) degree exhibited distributions akin to the most cohesive clusters (Fig. S2A and Fig. S2B). The *Urals-Carpathian EMA* cluster was loosely connected to the other IBD-sharing communities. Still, based on the low cluster coefficient, this separation could reflect gaps in sampling in time or space rather than true genetic isolation.

Within the Urals-Carpathian EMA cluster, the published 10th–11th century Carpathian Basin (CB) genomes (65) are grouped with our newly sequenced Volga-Ural Medieval samples. The KH groups exhibit high degree centrality, suggesting they hold a structurally central position within the cluster (Fig. S2C). In contrast, the early Medieval Carpathian Basin group displays a more diverse pattern of connectivity. The average IBD per link for both between- and within-module connections (Fig. S2D), is moderate for the KH groups compared to other modules. However, their high degree centrality suggests that they may occupy hub-like positions within the Urals-Carpathian EMA cluster. Notably, some 10th-century Carpathian Basin individuals fall into the *East-Asia/Carpathian IA-EMA* cluster, reflecting a genetically diverse migration into the region. We have observed that PCA (as well as the other allele-frequency-based methods) and the IBD network highlight distinct yet complementary aspects of population structure: the former is more sensitive to geographically structured genetic gradients, while the latter connects distant or close relatives who may occupy very different positions on these gradients (Fig.S1C,D,E).

### Early Medieval Magyars Fall within the Genetic Diversity of the Volga-Ural Region

We examined closely the genetic links between the Volga-Uralic groups and the 10th-century Carpathian Basin population forming the *Urals-Carpathian EMA* IBD cluster. The analysis showed that 10th-century Magyars in the Carpathian Basin exhibit significant genetic variation along PC1 (Fig. 2B), indicative of admixture during their migration westward or within the Carpathian Basin. As observed earlier, ancestries tracing back to the Baikal Neolithic and the Yakutia Late-Neolithic/Bronze Age varied across the EMM individuals. We mapped the proportions of these proxy ancestry sources onto our PCA (Fig. S3A). Consistent with the previously identified NWE-NEA and NWE-EA gradients, the EMMs demonstrate ancestry from two different East Eurasian sources. Specifically, those aligned with the NWE-NEA gradient exhibited a pronounced Yakutian Late-Neolithic/Bronze-Age ancestry, whereas those on the NWE-EA cline displayed higher levels of Baikal Neolithic ancestry (Fig. S3A,B). These ancestry components should not be interpreted as reflecting direct gene flows from Yakutia or the Baikal region; rather, the proxy sources are reference groups for broad geographical regions and chronological periods. All of these results suggest that substantially different genetic sources on the Siberian genetic landscape could have contributed to the *Urals-Carpathian EMA* cluster of distant relatives in the 10th-century Carpathian Basin.

Next, we focused on specific cases of strong IBD links between Early Medieval Magyars and the population of the Volga-Ural region, providing examples of long-distance migration within a few generations. We identified 28 pairs of individuals sharing more than two genome segments of 12 cM or longer (Table S2); of these, 11 pairs with the longest IBD segments are presented in Table 1. For their ancestry proportions estimated with *ADMIXTURE* see Fig. S3B. It is most likely that the degree of kinship for these pairs of individuals indicates close biological relatedness (even up to the 6th degree). (78) (Fig. S3C).

Dating based on archaeological context and radiocarbon analysis (Table S1G) show that most IBD segments link individuals within a couple of hundred years of each other. Owing to the broad chronological ranges provided by radiocarbon and archaeological dating, the observed connections between pairs of sixth-degree (or more distant) relatives may reflect either collateral relatedness through a shared common ancestor or direct ancestor–descendant relationships. The majority of the strong connections (>2 segments above 12 cM) of the EMM individuals are detected with the KH individuals from various ecoregions. To better understand the connection between the two regions, we conducted a qpWave analysis-based cladality test (86) (see Methods for details). This test assesses whether two populations of interest (referred to as left populations) form a clade or are genetically continuous, given a set of reference (right) populations (see Methods). As proxy ancestry sources for *Urals–Carpathian EMA* cluster individuals, we used Medieval Volga–Ural region groups that each included at least five individuals (Mid-Irtysh_Usthim, TransUral_KH, CisUral_KH, LowKama_KH, and MidVolga_EVB), representing a diverse genetic composition spanning the Trans-Uralian/Western Siberian to the Mid-Volga region. We paired each group with Urals–Carpathian EMA cluster individuals from the Carpathian Basin and found that individuals from the Carpathian Basin with the highest levels of genomic segment (IBD) sharing with KH groups (Table 1) primarily showed feasible models with the Cis and Trans-Uralian KH groups (Table S3) - an outcome also mirrored in their ADMIXTURE profiles (Table S1D). When paired with the Low-Kama group, only four individuals fit the model, while none fit with the Mid-Volga EVB or Mid-Irtysh group. Our cladality test thus provides a second, independent line of evidence - alongside the IBD links - supporting a genetic connection between the 10th-century Carpathian Basin EMMs and the Circum-Uralic KH groups.

We conducted a genetic mobility estimation analysis (mobest; see Methods for details, Table S1H for reference samples) to identify the most likely spatial origin of the EMMs. This analysis (Fig. S4) indicated that most EMMs from the *Urals-Carpathian EMA* cluster were associated with three potential source regions: one primarily from Europe (Fig. S4A), one from the Ural region (Fig. S4B), and another from somewhere in Central Asia (Fig. S4C). Among the EMMs, those with the strongest IBD-sharing with the Karaykupovo Horizon (KH) individuals and a high level of Yakutia-LNBA ancestry mostly displayed the highest similarity probabilities to the Ural region (e.g., the Szakony /SZAK/ individuals). The Baikal-N ancestry bearing individuals indicated the highest similarities for yet preliminarily defined Eastern Eurasian sources. In many cases, dual or tri-regional affinities were observed, with connections to all three regions; however, this pattern was not evident in the KH individuals (Fig. S4D, for reference samples see Fig. S4E).

### Iron Age genetic continuity in the Medieval Volga-Ural Region

**Figure 4.**
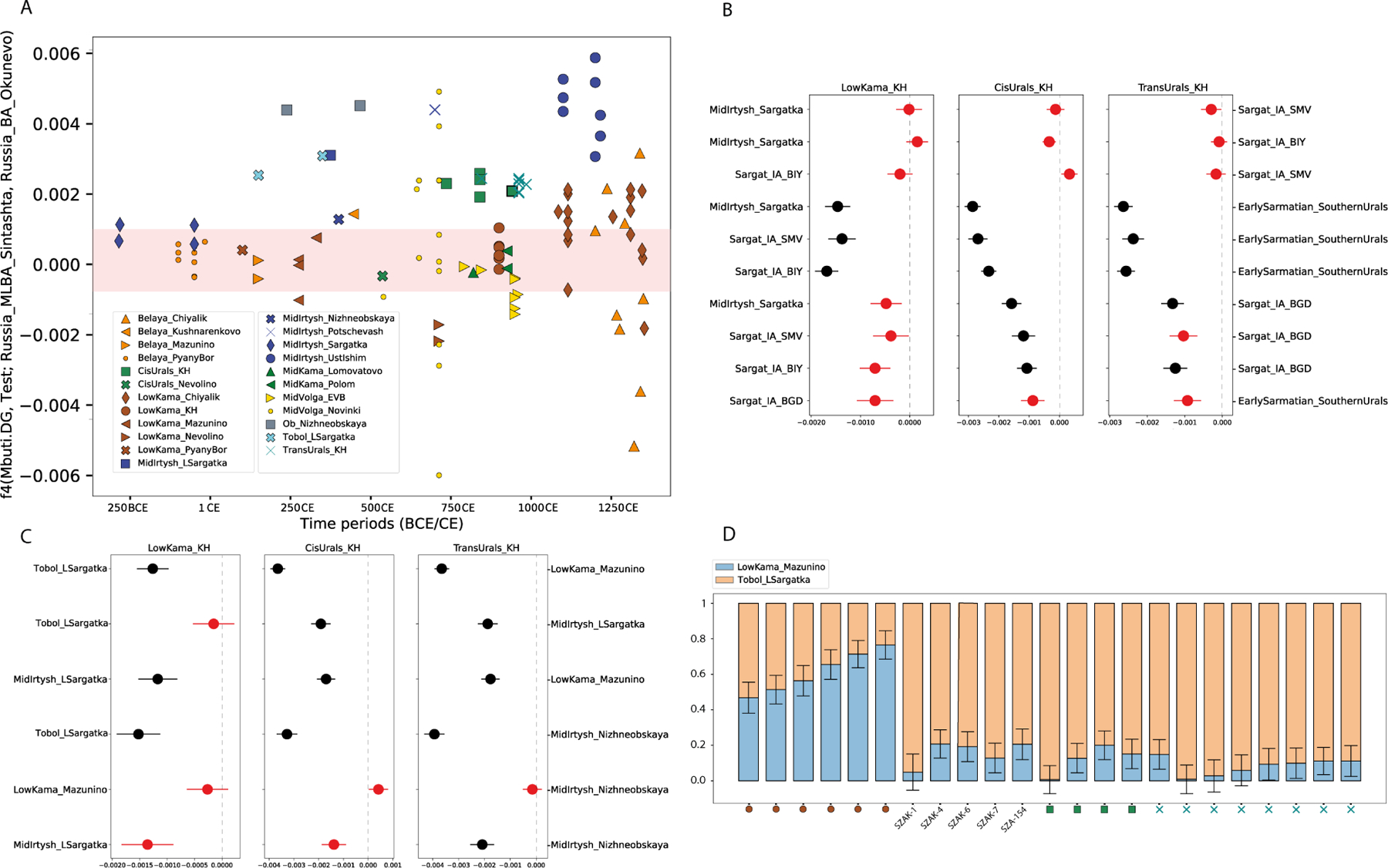
*f4*-statistics and admixture models illustrating allele sharing and genetic affinities among newly sequenced individuals and Bronze/Iron Age reference groups. **A**: *F4*-statistics for the newly sequenced individuals from the Volga-Ural regions, excluding those from the Maklasheevka and Ananyino cultural contexts. The Y-axis represents the allele sharing values with two Bronze Age reference groups (red band indicates |Z-score| < 3). The X-axis shows the timeline. **B:** *F4*-statistics in the form of *f*4(Mbuti, KH_group; EIA_test_1, EIA_test_2) assess allele sharing among KH groups and various Early Iron Age references. These analyses include individuals from the Sargatka horizon at the Shmakovo (SMV), Bogdanovka (BGD), and Mountain Bitiya (BIY) sites as described by Gnecchi-Ruscone et al.(61). Additionally, it features groups from the Southern Uralic and Sarmatian cultures as reported by Jarve et al.(49), alongside our newly introduced early Iron Age groups. Red markers denote |Z-score| < 3. **C:** *F4*-statistics comparing allele sharing between KH groups and Migration Period Volga-Uralian reference groups in the form of *f*4(Mbuti.DG, KH_test_group; MigrationPeriod_reference_group1, MigrationPeriod_reference_group2). The locations of the dots indicate affinities with left and right reference groups. Red markers denote |Z-score| < 3. **D:** A two-way admixture model (qpAdm, p>0.05) for the Karayakupovo Horizon and Early Medieval Magyar individuals from the 10–11th century Carpathian Basin (from Table 1) that exhibited strong IBD sharing (>42 cM in IBD segments longer than 12 cM; see Table S2 for additional details). For additional EMMs modeled with this two-way qpAdm setup see Table S1F.

To provide deeper insights into the genetic landscape of the Volga-Ural region, we applied *f4*-statistics (for details see Table S1E), aiming to test if there was a significant genetic shift in this region since the Bronze Age. For this purpose, we compared allele sharing between the newly sequenced individuals and selected Bronze Age reference individuals from the Southern Urals (attributed to the Sintashta culture) and South-Central Siberia (attributed to the Okunevo culture, from the Minusinsk Basin), as shown in Fig. 4A. Our analysis revealed that during the late phase of the Early Iron Age (EIA), the level of allele sharing was similar with both distant reference populations for the Circum-Uralic individuals dated to this period (culturally associated with the Pyany Bor and Sargatka contexts). Over time, particularly by the Medieval period, an increasing number of individuals displayed a stronger genetic affinity to one of these reference groups. Notably, the two individuals attributed to the transitional period from the late Sargatka to the Migration period cultural groups and buried in the Tobol region (see Data S1, I.C), stood out from the homogeneous Iron Age genetic continuum, showing a pronounced affinity with the South-Central Siberian Bronze Age (BA) reference group. One of these two individuals, ID I33844, comes from a burial dated to 250–320 CE at Ipkul in the Tobol River area - the latest site preserving the Late Sargatka tradition, which persisted in the remote periphery of its cultural area in admixture with a taiga-derived cultural environment (see Data S1 I.C, IV.B.2). Notably, this individual clusters with the *Urals–Carpathian EMA.* Similarly, the Trans and Cis-Uralian Karayakupovo Horizon individuals exhibited a strong affinity with this BA reference group. These results highlight the diverse population interactions during the Migration and, later, Medieval period compared to those of the Iron Age.

To test the Iron Age/Migration Period (for a detailed description of the archeological chronology in the region, see Data S1 I.A,B,C) individuals for evidence of continuity with early Medieval KH individuals, we used two complementary *f4*-statistics (for details see Table S1E). Initially, we tested allele sharing between our focal (KH) group, and both EIA Southern Uralic (associated with Sarmatian culture context) and Western Siberian groups (Sargatka horizon), which revealed reduced allele sharing of the KH groups with the Sarmatian cultural context when comparing to the Western Siberian groups (Fig. 4B). Furthermore, allele sharing analyses among Western Siberian groups revealed significant affinity between the Cis and Trans-Urals KH groups and EIA groups in the Irtysh River region. In the second stage, we analyzed Late Iron Age/Migration Period reference populations from the wider Volga-Ural region and tested allele sharing between them and the KH groups (Fig. 4C). This included the Low-Kama Mazunino group and groups from the Tobol and Mid-Irtysh regions from the Sargatka sites, including the latest ones in the Tobol region, and the Nizhneobskaya cultural contexts. The latter is distinct both archaeologically and genetically from the local continuum. Compared to the other reference populations, we observed significant allele sharing between the KH groups and the Tobol reference groups associated with the residual Sargatka sites. These findings indicate genetic continuity in the KH groups from the end of the Early Iron Age, suggesting their ancestry is rooted in the Irtysh and Tobol River regions.

To model possible admixture scenarios and quantify the proportion of the Migration Period ancestral sources (for KHs and EMMs with direct connections to KH individuals [Table 1]) we employed *qpAdm* analysis (Fig. 4D) (for the detailed settings, see Methods). We purposely avoided rotating modeling approaches exploring large sets of alternative proxy sources(92). Instead, we utilized a two-way modeling strategy with proxy sources on both sides of the Urals in the Late Iron Age/Migration Period: the residual Sargatka group in the Tobol basins, and Mazunino in the Low Kama basin. Their separation in the spaces of *f4*-statistics (Fig. 4A) and differences in ADMIXTURE proportions (Fig. 2A) justified the use of these sources for *qpAdm* analysis. Archaeological context also supports the significance of these groups as they potentially influenced the Kushnarenkovo and later Karayakupovo archeological cultures(8). In the case of the Mazunino group, we used the Low-Kama sub-group, which has sufficient coverage in our dataset. Out of the 20 analyzed KH individuals, the two-way model was a fit (p-value > 0.05) in 18 cases (for the list of outgroups see Methods). The Tobol Late Sargatka ancestry was notably prevalent among the Trans-Ural KH, Cis-Ural KH, and early Medieval Magyar individuals, at least ~70% (for detailed results, see Table S1F). While all EMM and KH groups likely share the same Trans-Uralic ancestry, some (Low-Kama KH, see Fig. 4D) mixed extensively with local groups to the west of the Urals.

A time-ordered IBD graph in Fig. S1A illustrates biological continuity, especially between the Early Medieval KH groups and those from the Late Medieval Chiyalik cultural contexts in the Belaya and especially Low-Kama regions. The similarity in *ADMIXTURE* profiles (Fig. 2A) further supports the continuity of the KH-type ancestry into the later Medieval period. In contrast, the Belaya region in the Late Medieval period is more diverse genetically, with several individuals having European and East Asian genetic profiles (supported by IBD connections outside the *Urals-Carpathian EMA* cluster).

To explore the demographic history of the Volga-Ural groups from a different perspective, we utilized the *hapROH* method to identify long runs of homozygosity (ROH), as shown in Fig. S5A,B(88). This analysis revealed that KH individuals probably had a small effective population size (*Ne*), evidenced by the ROH segments in their genome. The number of ROH segments per group correlated negatively with other estimates of genetic diversity used in this study. Our *Ne* analysis further indicated that both Early Medieval Low-Kama KH and Late Medieval Low-Kama Chiyalik groups had consistently smaller population sizes than neighboring groups across different periods (Table S4).

## Discussion

In this study, we report genome-wide data for 131 ancient human genomes from 1300 BCE to 1400 CE in the Circum-Ural region and the Carpathian Basin. The genetic gradients displayed on the PCA by the Volga-Ural region groups (Fig. 2B) align with the modern genetic variation found in Eurasia’s forest and forest-steppe zones (the northern one) and the steppe zone (the southern one), respectively(68). The Asian end of the northern gradient is linked to the Yakutian LNBA population, which is described as a genetic „tracer dye” for the spread of Uralic speakers in North Eurasia(68). The IBD analysis of chromosome segments revealed distant relatedness between Early Medieval Circum-Uralic individuals from the Karayakupovo Horizon sites and the EMM 10th-11th centuries population from the Carpathian Basin. We termed the IBD cluster of distant relatives as “*Urals-Carpathian EMA*” (Fig. 3C), which showed a genetic gradient stretching from Europe to Northeast Asia on PCA, and distinct from the *Eurasian steppe Iron Age* and *East Asia/Carpathian IA-EMA* IBD clusters (Fig. 3A, Fig S1C,D,E).

Our findings demonstrate that Cis- and Trans-Uralic Karayakupovo Horizon sites are linked to 10th-11th-century Carpathian Basin individuals in the IBD-sharing network. These connections are supported by similarity in *ADMIXTURE* profiles, *qpWave* based cladality tests and *mobest* mobility estimation. Notably, individuals from the western Hungarian Szakony-Kavicsbánya site displayed the highest similarities to the Volga-Uralic population in *ADMIXTURE* clustering and IBD-sharing. Archaeological artifacts from this site and burial customs show direct parallels in Uralic cultural contexts(93). These combined findings provide the first compelling genetic evidence supporting a dominant Uralic origin for a significant portion of the ancestry of 10th-century Magyars in the Carpathian Basin. EMMs from the Carpathian Basin mostly demonstrate Yakutian LNBA-type ancestry associated with the northern (forest and forest-steppe) Eurasian gradient. Still, some also demonstrate Baikal Neolithic-related ancestry associated with the southern (steppe) Eurasian gradient (Fig. S3A). The genetic mobility estimation analysis of the EMMs also indicates that a significant portion of the EMMs originated from the Ural region. Some of them may have migrated rapidly, possibly within the span of a single generation (e.g., individuals from the Szakony-Kavicsbánya site). Additionally, we identified other potential regions, likely in Central Asia, as sources of gene flow into the EMMs. However, it is important to note that this gene flow was not observed in the Early Medieval Karaykupovo Horizon (KH) individuals (Fig. S4D), suggesting that this admixture likely occurred outside the Volga-Ural region, during the EMMs westward migration. These results imply that they (or their ancestors) have at least two genetic sources outside the Carpathian Basin, and we confirmed the Circum-Uralic one. Considering the archeological, historical, and genetic results, our findings are consistent with a scenario in which the initial area of the EMM migration to the Carpathian Basin was located in the Volga and Ural regions, where traces of admixture are not observable with Central/East-Eurasian ancestry bearing groups (such as people usually attributed to the Turkic speakers(38,50). The results presented in our paper align with the Uralic (Ugric) basis of the Hungarian language, which has its first written documents only as late as 11th century Hungary(94). Among the possible Early Medieval influxes to the Carpathian Basin, the Hungarian language was most probably brought from the Southern Ural region (by descendants of the members of the Karayakupovo archaeological horizon), among others by those Magyars who shared the *Urals-Carpathian EMA cluster*. However, it is important to emphasize that the Magyar-associated archaeological assemblages demonstrate diverse cultural backgrounds and likely reflect multiethnic/multilingual communities(16,95,96). The most recent reconstructions of the Magyar migration based on material culture evidence favours the subsequent population movement from the Volga to the Pontic Steppe as late as the early 9th century CE, and from there to the Carpathian Basin by the end (or second half) of that century(3,6). The tight connectedness of the Urals-Carpathian EMA cluster and the genetic characteristics of a part of the EMM indicate a rapid migration from the Volga-Ural to the Carpathian Basin and a rather short stop in the North-Pontic area. This later area could have been the site for the integration and alliance with steppe Turkic-speaking population(3).

We propose referring to this specific type of ancestry, best observed among Uralic Early Medieval individuals and later identified in the EMMs, as the “Karayakupovo-type.” We detect the first emergence of it to the west of the Urals, by 550 CE. This ancestry did not extend as far west as the Volga-Kama confluence or the Volga’s west bank by the Samara Bend, as it is absent in the group with Novinki-type burial practices (for the description of the Novinki group see Data S1 II.F). Furthermore, our findings indicate that individuals from the Early Volga Bulghar (EVB) Mullovka and Tankeyevka cemeteries also show dissimilarity to the Karayakupovo Horizon sites (for the description of the EVB group see Data S1 II.G). Our analyses indicate a low level of IBD connection between the KH and Medieval Ob (possibly proto-Ob-Ugric) groups in Western Siberia either, despite their close geographical proximity for 1500–2000 years following their estimated linguistic split (74).

As the KH groups demonstrated notably strong IBD connectivity despite considerable geographical distances (Low-Kama, Cis-Urals, Trans-Urals), we investigated the extent of their shared population history. Using multiple *f4*-statistics, we demonstrated that the KH groups shared the most alleles with groups from the Irtysh and Tobol regions throughout the Iron Age and Migration Period. This evidence supports the hypothesis of a Trans-Uralian origin for the later Karayakupovo-type ancestry. Our proximal *qpAdm* analysis showed that the Low-Kama KH group could be modeled as a combination of Pyany-Bor/Mazunino and Tobol residual Sargatka-related ancestries, resulting in a distinct local KH variant. In contrast, the other KH groups have much lower Pyany-Bor/Mazunino ancestry. We demonstrate that the proxy ancestry sources we used in our *qpAdm* analyses (Pyany-Bor/Mazunino to the west of the Urals and Tobol Late Sargatka to the east of the Urals) are much closer to the actual sources than those used in the Maróti et al. *qpAdm* approach(65), which suggested using modern Mansis, early/late Sarmatians, and Xiongnu as proxies for modeling the ancestors of the EMMs. Based on the connections with the KH individuals, we show that an important stratum of the EMMs (named by Maróti et al. as ‘Conqueror Asia Core’) can be traced to the Early Medieval Circum-Uralic region. Also, with *qpAdm* modeling, we detected local biological continuity from the Iron Age to the Early Medieval times in these regions. However, we avoided extensive *qpAdm* screenings across multiple ancestry sources closely timed to target groups, similar to the approach used in Maróti et al.(65), due to the high risk of false-discovery rates, as demonstrated by Flegontova et al.(92). Additionally, archaeologists have determined that in the Early Middle Ages, the area east of the Ural Mountains, extending to the Ob River in the present-day Omsk region, had an extremely low population density. The total number of excavated graves from the 6th to the 10th centuries AD does not exceed 300(97). We have detected extended genetic signals indicating small population sizes both east of the Urals and in the Cis-Urals KH group. These findings provide significant evidence of sparse and low population sizes in these regions during this period.

The Late Medieval Chiyalik group that occupied the Lower Kama region shows strong continuity within the *Ural-Carpathian EMA IBD* cluster. This is indicated by a high level of connectivity within the IBD-sharing community and limited IBD sharing beyond it. Moreover, they are similar to the KH groups on an allele frequency level. In contrast, individuals attributed to the Chiyalik culture in the Belaya River Basin are more diverse genetically and fall outside the *Urals-Carpathian EMA* IBD-sharing cluster. These findings suggest the potential influx of newcomers during the Golden Horde domination, when heightened transcontinental communications likely introduced various East Eurasian genetic ancestries that were rare in the Urals before (Data S1 III.H). Considering the late 14th-century radiocarbon dates for the Chiyalik individuals, it is reasonable to assume the presence of remaining Magyars, archaeologically represented by a local variety of the Chiyalik culture, mainly in the Lower Kama River Valley(98,99). We also did not detect widespread Central European ancestry among the Chiyalik period individuals, suggesting no back migration from the Late Medieval Carpathian Basin. By analyzing the effective population size, we estimate that the Low-Kama Chiyalik group comprised at least a few thousand individuals during Late Medieval times. These results suggest that descendants of the *Uralic-Carpathian EMA* IBD-sharing community survived in Late Medieval times in considerable numbers in the Kama region. We assume that the Low Kama region near the Belaya-Kama confluence was the area that was called Magna Hungaria by Friar Julian in the 13th century(29). In addition to this historically documented data, the regional toponymy suggests the presence of Hungarian-speaking groups there until the 16th century, when, after the collapse of the Golden Horde imperial space, they were absorbed into the Late Medieval populations of modern-day Bashkortostan, Tatarstan, and Udmurtia(6,100,101).

### Limitations of the study

The results of this study have linked the 10th-century Carpathian Basin and the Medieval Circum-Uralic region through the westward migration of the EMMs. Nevertheless, the scarce availability of Medieval genomes spanning the vast space from Eastern Europe to Western Siberia means that we can say relatively little about contributions from many regions. While it was possible to identify one source of EMM ancestry in this study (in the Volga-Ural region), detecting the origin of other genetic components (e.g. the Baikal Neolithic-type ancestry) is challenging with the available data. Future research on samples from the whole migration route of the EMMs and its adjacent territories would help reveal the full set of genetic sources of the 10th century Carpathian Basin population.

## Resource availability

### Lead contact

- Requests for further information and resources should be directed to and will be fulfilled by the lead contact, Anna Szécsényi-Nagy (szecsenyinagy.anna@abtk.hun-ren.hu)

### Materials availability

- This study did not generate new unique reagents.

### Data and code availability

- Newly reported ancient sequencing data have been deposited at European Nucleotide Archive (ENA) with the following accession number ENA: PRJEB83577
- This paper does not report original code.
- Any additional information required to reanalyze the data reported in this paper is available from the lead contact upon request.

## STAR methods

### Experimental model and study participant details

- Ancient individuals: Extensive description of the archeological context for the ancient individuals analyzed in this study is available in Data S1.
- Sampling and sample selection: Based on years of collaborations with local archaeological experts governed by bilateral collaboration agreements, we selected the most relevant and available samples, which were in certain cases verified by radiocarbon dating. We did not perform a priori sample size calculations. Our aim was to achieve equal/representative sampling across the sites based on archaeological relevance and sample availability. Allocation to experimental groups was determined by geographical and chronological frameworks (see Results and Data S1 for details). We aimed to collect graves for this research with archaeological materials characteristic of local cultures (see Data S1, section I. for details). In the **Trans-Urals,** our sampling involves individuals buried in the Sargatka cultural context from the Middle Irtysh (300 BCE - 200 CE) and the Tobol (100–350 CE) river basins. The later Trans-Uralic population groups are represented by burials attributed to the Medieval (in cases of inconsistency between radiocarbon dates and archaeological context dating at Trans-Uralic sites, we preferred the dates based on the archaeological context due to the high probability of a freshwater reservoir effect in human bones from this area) Nizhneobskaya, Potchevash, and Ust’-Ishim cultures, and the Uyelgi cemetery attributed to the Karayakupovo Horizon. In the **Cis-Urals**, we undertook a dense sampling from sites attributed to the Maklasheevka Late Bronze Age (1100–900 BCE), Post-Maklasheevka Ananyino Early Iron Age (900–250 BCE), Pyany Bor Early Iron Age (250 BCE-150 CE), Mazunino (150–450 CE), and Nevolino (400–850 CE) archaeological entities. The Migration-period population archaeologically related to the Cis-Uralic groups is represented by one individual from the Kushnarenkovo cultural context (550–700 CE). The sampling of the Medieval individuals of the Volga-Ural region involves the peripheral regions of the Volga Bulgaria, to the east of the main cities and densely populated areas. Our Medieval samples originate from burials carried out according to Muslim rites with some pagan elements, a pattern typically attributed to the Chiyalik culture. The sites of the **Karayakupovo Horizon** to the west of the Urals are represented by Bolshiye Tigany from the Lower Kama region (800–900 CE). We also included some sites contemporaneous with the Karayakupovo Horizon, but archaeologically attributed to other groups: the Novinki-type sites (700–850 CE) and the Tankeevka cemetery (850–1000 CE), a local group of the Khazar-Khaganate nomads and the Early Volga Bulghars respectively (see further details in the SI). Two individuals from the Polom cultural context and one from Lomovatovo represent the Mid-Kama population groups that are contemporaneous with the people of the Karayakupovo Horizon sites.
- Ethics declaration: The individuals studied here were all analyzed with the goal of minimizing damage, with permission from local authorities in each location from which they came. Every sample is represented by archaeologists who hold the scientific copyright of the samples, have research agreements with Hungarian scientific institutions affiliated with the authors, and are leading archaeological experts with specific knowledge of the samples and their archaeological context. They are authors of this paper or named in the Acknowledgments. Open science principles require making all data used to support the conclusions of a study maximally available, and we support these principles here by making fully publicly available not only the digital copies of molecules (the uploaded sequences) but also the molecular copies (the ancient DNA libraries themselves, which constitute molecular data storage). Those researchers who wish to carry out deeper sequencing of libraries published in this study should make a request to corresponding author D.R.. We commit to granting reasonable requests as long as the libraries remain preserved in our laboratories, with no requirement that we be included as collaborators or co-authors on any resulting publications.

### Method details

- Ancient DNA data generation: 77 samples processed in the Budapest Laboratory of Archaeogenetics (Institute of Archaeology RCH) and shipped to Harvard Medical School. Sample surfaces were cleaned using mechanical methods and also UV irradiation. After that the bone powder was generated from petrous bones or teeth (74). Three samples were prepared in Vienna, and seven samples were prepared in Ostrava and shipped to the Harvard laboratory (for further details see Table S1A). In dedicated clean rooms, we extracted DNA manually with spin columns(102,103) or automated using silica magnetic beads and Qiagen PB buffer on the Agilent Bravo NGS workstation(104) and converted it into barcoded double-stranded partial Uracil-treated libraries(105) or USER-treated single-stranded libraries(106) which we enriched in solution for sequences overlapping 1.24 million SNPs [1240k: Fu et al.(33), Twist: Rohland et al.(107)] as well as the mitochondrial genome. For each library, we sequenced approximately 30 million reads pairs (median of 29.747M reads) of each enriched library using Illumina instruments [NextSeq500, HiSeq X]; we also sequenced several hundred thousand sequences of the unenriched libraries (Table S1A). Each sample was processed individually, while the laboratory procedures were conducted alongside extraction blanks. Although there was no biological replication during the laboratory processing, we performed next-generation sequencing (NGS), which generated millions of sequencing reads.
- Bioinformatic data processing: Samples were sequenced to generate raw paired-end reads; these were prepared for analysis by performing the following steps: preprocessing/alignment, and post-alignment filtering to enable variant calling. Raw reads were demultiplexed by using identifying barcodes and indices to assign each read to a particular sample, prior to stripping these identifying tags. Paired-end reads were merged into a single molecule using the base overlaps as a guide, Single-ended reads were aligned to the hg19 human reference genome (https://www.internationalgenome.org/category/grch37/) and the basal Reconstructed Sapiens Reference Sequence (RSRS)(108) mitochondrial genome using the same aligner of bwa(v0.7.15-r1140)(109). Duplicate molecules were marked based on barcoding bin, start/stop positions and orientation. For calling variants, a pseudo-haploid approach is used at targeted SNPs, where a single base is randomly selected from a pool of possible bases at that position filtering by a minimum mapping quality of 10 and base quality 20, after trimming reads by 2 base pairs at both 5’ and 3’ ends to remove damage artifacts. Sex determination, contamination estimation, mtDNA and Y-chromosomal haplogroup determination were conducted along with the mentioned other steps using tools implemented in the computational pipelines of the Harvard laboratory. Scripts with specific parameters are publicly available on GitHub at: https://github.com/dReichLab/ADNA-Tools and https://github.com/dReichLab/adna-workflow. Ancient DNA authenticity was verified using contamMix (v1.0.10511)(110) to detect heterogeneity in mitochondrial DNA sequences and ANGSD (0.921–3-g40ac3d6)(111) to detect heterogeneity in X chromosome sequences. Authenticity of the ancient samples was also evaluated by using pmdtools(v0.60.5)(112). A consensus for mitochondrial DNA was determined by using bcftools(113) and SAMTools(114). Mitochondrial haplogroups were determined using HaploGrep2(115), based on the phylotree database (mtDNA tree build 17)(116). For Y-chromosome haplogroup determination, we used YFull YTree v.8.09 (https://www.yfull.com/).

### Quantification and statistical analysis

- Principal component analysis (PCA): PCA analysis was carried out with EIGENSOFT software(117) (version 5.0) with lsqproject: YES and shrink mode: YES settings. For the calculation of the PCs, we used modern-day Eurasians from the Affymetrix Human Origin array(50), and projected the ancient samples onto the top PCs.
- ADMIXTURE analysis: Before running ADMIXTURE(87) we pruned our dataset with plink (version 3)(118). We used the -geno 0.95 option to ensure that we included sites where most individuals were covered at least once. After that we used --indep-pairwise 200 25 0.4 parameters for linkage disequilibrium (LD) pruning. We performed supervised ADMIXTURE clustering with K=8. We used Neolithic/Early Bronze Age populations as sources to reflect the overall distribution of different ancestries through Eurasia. We aimed to include well-represented groups (more than 4 individuals) with high-coverage data and no close relatedness (up to third degree). We intentionally aimed to reconstruct a similar ADMIXTURE reference set presented in Zeng et al.(68) (Table S1D): Russia_Samara_EBA_Yamnaya, Turkey_N, Lithuania_EMN_Narva, Russia_Baikal_N, Russia_Altai_N, Russia_Yakutia_LNBA, Russia_WSHG and Russia_EHG. We have found this set useful in understanding the pre-historical genetic composition of our newly analysed individuals.
- Genotype imputation: For imputation, we applied the software GLIMPSE (v.1.1.1)(77) with the 1000 Genome Project data as the haplotype reference panel to estimate genotype posterior probabilities and phased genotypes at bi-allelic SNP sites in the 1000G data, as described previously in (78). For IBD analysis, we then restricted to variants in the 1240k SNP panel, which are informative for ancient DNA studies. These VCF files were generated by downsampling the imputed 1000G SNP set to 1240k SNPs. A full description of the imputation pipeline is provided in Supplementary Note 3 (see also Figure 1b of Ringbauer et al.(78)).
- IBD-sharing analysis: To infer identical by descent (IBD) segments, we applied the program *ancIBD (v0.7)* (78) to the imputed genotype data. As recommended by (78) for accurate IBD detection, we only included individuals that have a maximum genotype probability >0.99 for at least 70% of all imputed 1240k SNPs on Chromosome 3(78) and call IBD segments >8 centimorgan long. In the downstream IBD segment analysis, we included all individuals that matched our research criteria, based on geographical location (coordinates in North Eurasia) and on timeframe (from ~1000 BCE to modern times).
- IBD-sharing network: All IBD networks were built using the Gephi (v.0.10.1) software(119). The graph’s edges were weighted based on the length of the most substantial shared IBD segment between two individuals, referred to as nodes. To filter spurious connections, we removed IBD segments below a threshold of 9 cM and connections that spanned over 600 years (for non-filtered network see Fig. S1B). Additionally, we maintained nodes connected by at least two edges and focused on the largest interconnected segment of the graph. Visualization was achieved using the MultiGravity ForceAtlas 2, a force-directed layout algorithm(90). In the processed graph, clusters were discerned using the Leiden algorithm(91), maintaining algorithmic independence. We explored several key metrics using the Python package NetworkX (120): degree centrality as well as within-module degree (kW), representing connections within each predefined cluster; and between-module degree (kB), capturing connections between different clusters. To assess the average IBD per link within and between modules, we used sum IBD segments > 12 cM as weighted edges, considering our predefined groups as modules.
- *f-*statistics: We computed *f4*-statistics (for samples with >200k SNPs covered on the 1240k panel) with the ADMIXTOOLS software package(86) with the qpDstat (f4Mode: YES; printsd: YES) packages. For *f4*-statistics we used the form (Mbuti, Target; Test1, Test2) to check the genetic affinities between two possible ancestral populations. For the pairwise cladality test, we used the ‘qpWave pairs’ test from the R software package Admixtools 2 with default settings(82, 86). We designated 10th to 11th-century Carpathian Basin individuals as targets (each individual was analyzed separately) and the five Medieval Volga-Ural region groups as left populations (MidIrtysh_Usthim, TransUral_KH, CisUral_KH, LowKama_KH, and MidVolga_EVB). The right populations included Mbuti, Italy_North_Villabruna_HG, Russia_MA1_HG, Russia_Caucasus_Eneolithic, Russia_Ekven_IA, Russia_DevilsCave_N, Russia_UstIda_LN.SG, Russia_KolymaRiver_LN, Russia_EHG, Turkey_N, and Iran_GanjDareh_N.
- Mobility estimation (*mobest*): To investigate potential origins of the Early Medieval Magyars, as well as the Karayakupovo Horizon individuals, we conducted a mobility estimation analysis (*mobest*)(121). For comparison, we included Late Iron Age/Migration Period samples, as detailed in Table S1H and Fig. S4E. The analysis was based on the first two principal components of the PCA described above, using the standard settings (https://nevrome.de/mobest).
- qpAdm analysis: For the qpAdm analysis (for samples with >200k SNPs covered on the 1240k panel), we used the *Admixtools 2* R package(83), with the following elected(85) outgroups: Mbuti, Ami, Italy_North_Villabruna_HG, Turkey_N.SG, Russia_Ekven_IA.SG, Russia_DevilsCave_N.SG, Russia_Sidelkino_HG.SG, Russia_Caucasus_Eneolithic, Tarim_EMBA1. We avoided using the rotating approach as in complex demographic histories the direction of the gene flows cannot be defined accurately(92).
- Consanguinity test (ROHs): Detecting runs of homozygous blocks with *hapROH*(88) software can provide signals of consanguinity, whereas small homozygous runs are indicative of a small recent effective population size. The program was used with default parameters for pseudo-haploid genotypes with at least 400k SNP covered. The *Ne* module of this program was also used to estimate effective population sizes with CI, considering 4–20 cM ROHs.
- Biological relatedness: We used KIN(122) to assess potential relatedness among the newly sampled individuals. KIN can detect relationships up to the third degree and distinguish between parent–child and sibling pairs. We applied a log-likelihood ratio threshold of >2 (Table S1I).
- Radiocarbon dating: Radiocarbon dating of 14 DNA samples was performed in the Penn State’s Radiocarbon Laboratory (PSUAMS codes). The BP values were calibrated in the Oxcal program 4.4 with a calibration curve IntCal 20(123, 124).

## Supplementary Material

1

2

3

4

5

6

7

8

Document S1 DataS1, archeological background on the newly presented individuals, related to Figure 1.

Table S1. A combined Excel file providing supplementary data, related to Figure1.

Table S1A: Summary of the archaeological context and genetic results for all newly analyzed individuals presented in this study.

Table S1B: Individuals of the IBD-sharing network with newly presented and published samples (in the network as nodes).

Table S1C: Levels of IBD-sharing between individuals represented as edges in the presented network.

Table S1D: Supervised ADMIXTURE (K=8) analysis of newly published individuals.

Table S1E: f-statistics of the newly presented groups and individuals.

Table S1F: Two-way proximal qpAdm modeling of Karayakupovo Horizon individuals using Migration Period sources from the Volga-Ural region.

Table S1G: Details on newly reported radiocarbon dates and associated stable isotope data used for quality control.

Table S1H: Reference samples used for mobility estimation (mobest) analysis.

Table S1I: Biological relatedness estimation with KIN

**Figure S1. Detailed analyses of the IBD network: time-ordered network and PC projections, related to Figure 3. A:** Time-ordered IBD-sharing network version of Figure 3. **B:** The non-filtered version of the IBD-sharing network, corresponding to Figure 3. The cluster symbols are simplified here for the Urals-Carpathian EMA cluster individuals for easier interpretation. Individuals of the IBD-sharing network (Fig. 3.) are also colored using PC1(**C**), PC3(**D**) and PC2(**E**) values, calculated based on modern Eurasian individuals(50).

**Figure S2. Analysis of clustering coefficients and module connections in IBD-sharing networks, related to Figure 3. A**: A scatterplot displaying the average clustering coefficient of each cluster versus the ratio of between-module connections (kB) to the total degree (k) of the cluster. Each cluster is identified using the Leiden algorithm, which determines community structure in the network. The ‘modules’ in this context refer to the clusters defined by this algorithm. **B**: A scatterplot displaying the average clustering coefficient of each cluster versus the ratio of within-module connections (kw) to the total degree (k) of the cluster. Each cluster is identified using the Leiden algorithm, which determines community structure in the network. The ‘modules’ in this context refer to the clusters defined by this algorithm. **C**: Scatterplot of Between-module Strength versus within-module strength in the Urals-Carpathian EMA Cluster (modules defined by groups). **D**: Scatterplot of degree centrality versus clustering coefficient for all individuals in the Urals-Carpathian EMA cluster.

**Figure S3. Comprehensive analysis of EMMs in the Urals-Carpathian EMA cluster: PCA projections, IBD segment distribution, and supervised ADMIXTURE analysis, related to Figure 2, Figure 3 and Table 1. A**: PCA projection with estimates of the Yakutia LNBA and Baikal Neolithic ancestry components from the supervised ADMIXTURE analysis presented in Fig. 2B are shown on the right. In the case of the Yakutia LNBA ancestry, deeper blue color means higher proportion of this ancestry, while deeper red color means higher proportion of the Baikal Neolithic ancestry. Gradients as legends represent the scale of the respective ancestry, with two given EMM samples exhibiting the highest level of each component in the analysed dataset. **B**: A supervised ADMIXTURE (K=8) analysis of 10th century Carpathian Basin individuals from the Ural-Carpathian EMA IBD-sharing community. **C**: IBD segment sharing patterns between individuals from the Volga-Ural region and Early Medieval Carpathian Basin (Table S2). Only pairs of individuals sharing at least two segments longer than 12 cM were considered.

**Figure S4. Mobest estimation for individuals in the Urals-Carpathian EMA cluster, related to Figure 2, 3 and 4. A:** Four individuals are presented with high levels of European ancestry from the Urals-Carpathian EMA cluster. **B:** Four individuals are presented with high levels of Yakutia-LNBA ancestry from the Urals-Carpathian EMA cluster. **C:** Four individuals are presented with high levels of Baikal-N ancestry from the Urals-Carpathian EMA cluster. **(A-C)** Red dots indicate the geographical coordinates of the respective sites of the samples. **D:** Three individuals are presented, each representing one sample from the Low-Kama, Trans-Urals, and Cis-Urals KH groups, with red dots marking their geographical coordinates. **E:** Distribution of reference samples used in the mobility estimation is shown; precise dating and geographical origins are detailed in Table S1H.

**Figure S5. hapROH analysis and individual ROH histograms: Volga-Uralian and 10th-11th CE Carpathian Basin individuals, related to Figure 3. A**: hapROH results for the Volga-Uralian and 10–11th CE Carpathian Basin individuals (both newly presented and previously published data(65)). **B**: Individual ROH histograms of individuals that show high (>50 cM sum IBD from >20 cM segment) signal of parental relatedness. They show marriages of at least first cousin level relatives.

## Figures and Tables

**Table:** Key resources table

REAGENT or RESOURCE	SOURCE	IDENTIFIER
Antibodies		
Bacterial and virus strains		
Biological samples		
131 newly reported ancient individuals	This paper	N/A
Chemicals, peptides, and recombinant proteins		
23 HI-RPM hybridization buffer	Agilent Technologies	5190–0403
Herculase II Fusion DNA Polymerase	Agilent Technologies	600679
Pfu Turbo Cx Hotstart DNA Polymerase	Agilent Technologies	600412
50% PEG 8000	Anatrace	OPTIMIZE-82 100 ML
0.5 M EDTA pH 8.0	BioExpress	E177
Sera-Mag SpeedBead CarboxylateModified [E3] Magnetic Particles	Cytiva Life Sciences	65152105050250
silica magnetic beads	G-Biosciences	786–916
10 x T4 RNA Ligase Buffer	New England Biolabs	B0216L
Bst DNA Polymerase2.0, large frag.	New England Biolabs	M0537
UGI	New England Biolabs	M0281
USER enzyme	New England Biolabs	M5505
Buffer PB	QIAGEN	19066
Buffer PE concentrate	QIAGEN	19065
1 M Tris-HCl pH 8.0	Sigma Aldrich	AM9856
1 M NaOH	Sigma Aldrich	71463
20% SDS	Sigma Aldrich	5030
3 M Sodium Acetate (pH 5.2)	Sigma Aldrich	S7899
5 M NaCl	Sigma Aldrich	S5150
Ethanol	Sigma Aldrich	E7023
Guanidine hydrochloride	Sigma Aldrich	G3272
Isopropanol	Sigma Aldrich	650447
PEG-8000	Sigma Aldrich	89510
Proteinase K	Sigma Aldrich	P6556
Tween-20	Sigma Aldrich	P9416
Water	Sigma Aldrich	W4502
10x Buffer Tango	Thermo Fisher Scientific	BY5
50x Denhardt’s solution	Thermo Fisher Scientific	750018
AccuPrime Pfx Polymerase (2.5 U/ul)	Thermo Fisher Scientific	12344032
ATP	Thermo Fisher Scientific	R0441
dNTP Mix	Thermo Fisher Scientific	R1121
Dyna MyOne Streptavidin C1 beads	Thermo Fisher Scientific	65002
FastAP (1 U/mL)	Thermo Fisher Scientific	EF0651
GeneAmp 103 PCR Gold Buffer	Thermo Fisher Scientific	4379874
Human Cot-I DNA	Thermo Fisher Scientific	15279011
Klenow Fragment (10 U/mL)	Thermo Fisher Scientific	EP0052
Maxima Probe qPCR 2xMM	Thermo Fisher Scientific	K0233
Maxima SYBR Green kit	Thermo Fisher Scientific	K0251
Maxima SYBR Green kit	Thermo Fisher Scientific	K0253
Salmon sperm DNA	Thermo Fisher Scientific	15632–011
SSC Buffer (203)	Thermo Fisher Scientific	AM9770
T4 DNA Ligase	Thermo Fisher Scientific	EL0012
T4 DNA Ligase, HC (30U/mL)	Thermo Fisher Scientific	EL0013
T4 DNA Polymerase	Thermo Fisher Scientific	EP0062
T4 Polynucleotide Kinase	Thermo Fisher Scientific	EK0032
23 HI-RPM hybridization buffer	Agilent Technologies	5190–0403
Critical commercial assays		
Twist Alliance Ancient Human DNA Panel	Twist BioSciences	part number 106658
High Pure Viral Nucleic Acid Large Volume Kit	Roche	part number 05114403001
HiSeq X Ten Reagent Kit v2.5	Illumina	FC-501–2521
NextSeq 500/550 High Output Kit v2.5	Illumina	Cat.# 20024906
Deposited data		
Sequencing data from 131 newly reported ancient individual	This paper	ENA: PRJEB83577
Isotopic data of newly reported ancient individual	This paper	Data S7
Experimental models: Cell lines		
Experimental models: Organisms/strains		
Oligonucleotides		
Recombinant DNA		
Software and algorithms		
Bwa	Li et al.^109^	https://maq.sourceforge.net
ADNA-Tools	https://github.com/dReichLab/ADNA-Tools	https://github.com/dReichLab/ADNA-Tools
adna-workflow	https://github.com/dReichLab/adna-workflow	https://github.com/dReichLab/adna-workflow
EIGENSOFT	Patterson et al.^117^	https://github.com/DReichLab/EIG
ADMIXTURE	Alexander et al.^87^	https://dalexander.github.io/admixture
Plink	Chang et al.^118^	https://www.cog-genomics.org/plink/2.0
GLIMPSE	Rubinacci et al.^77^	https://odelaneau.github.io/GLIMPSE
ancIBD	Ringbauer et al.^78^	https://github.com/hringbauer/ancIBD
Gephi	Bastian et al.^119^	https://gephi.org
ForceAtlas2	Jacomy et al.^89^	N/A
NetworkX	Hagberg et al.^120^	https://networkx.org
Admixtools2	Maier et al.^86^	https://uqrmaie1.github.io/admixtools
hapROH	Ringbauer et al.^88^	https://github.com/hringbauer/hapROH
mobest	Schmid & Schiffels 2023^121^	https://github.com/nevrome/mobest
KIN	Popli et al.^122^	https://github.com/DivyaratanPopli/Kinship_Inference
OxCal v4.4.2	Ramsey^123^	N/A
Other		

**Table 1:** IBD connections between Medieval Volga-Uralic and Carpathian Basin individuals. Radiocarbon dates (calibrated, 95% confidence interval) are highlighted in bold. In other cases, the dating is based on the archaeological chronology of the material culture. *summed probability densities, based on samples radiocarbon dated from the same site.

*10–11th century Early Medieval Magyars in the Carpathian Basin*				*Volga-Ural individuals*					
*Ind1*	*Date*	Sex	Y/mtDNA	*Group*	*Ind*	Sex	Y/mtDNA	*Date*	*total length of shared IBD segments >2 × 12 cM*
SEO-4	900–1000 CE	male	G2a/T2g1a	Mid-Volga EVB	I25526	male	Q/B5b4	850–1050 CE	144
SZAK-1	900–1000 CE	male	N1a/T2d1b1	Trans-Urals KH	I19117	male	N1a/N1a	***771–937 calCE***	92
K2–61	900–950 CE	male	R1/U4d2	Cis-Urals KH	I25538	male	N1a/U5a1g1	664–1016 CE*	67
SZAK-7	900–1000 CE	female	-/D5a1	Trans-Urals KH	I19118	male	G2a/A+152	772–1152 CE*	42
SZAK-7	900–1000 CE	female	-/D5a1	Cis-Urals KH	I25538	male	N1a/U5a1g1	664–1016 CE*	63
SZAK-4	900–1000 CE	female	-/HV4a2a	Cis-Urals KH	I25537	male	N1a/H6a1b	664–1016 CE*	43
SZA-154	900–1000 CE	female	-/B5b4	Trans-Urals KH	I19120	male	N1a/A12a	772–1152 CE*	42
SZAK-6	900–1000 CE	female	-/A16	Low-Kama KH	I19105	female	-/A12a	850–950 CE	45
SZAK-1	900–1000 CE	male	N1a/T2d1b1	Trans-Urals KH	I19121	male	N1a/U5a1a1	***879–1150 calCE***	46
K3–6	900–1000 CE	female	-/B4d1	Cis-Urals KH	I25536	male	N1a/C4a2	***664–827 calCE***	46

## Highlights

- Genome-wide data of 131 ancient individuals from the Volga-Urals and Carpathian Basin
- 10th-century Carpathian Basin and Southern Uralic populations show strong IBD sharing
- Primary Southern Uralic origin and rapid migration of Magyars to the Carpathian Basin
- Genetic continuity from the Late Iron Age to the Medieval Circum-Uralic region
