# Supplementary material for: Long shared haplotypes identify the Southern Urals as a primary source for the 10th century Hungarians
Source: Cell. Author manuscript; Available in PMC 2025 Dec 17. (PMC12711333; doi:10.1016/j.cell.2025.09.002)
Supplement: 1 [file NIHMS2116457-supplement-1.pdf]

Supplementary Data

for

# “Long shared haplotypes identify the Southern Urals as a primary source for the 10th century Hungarians”

# Combined archaeological and genetic context of the newly sequenced individuals


**Supplementary data guide ........ 6**
**I. Chronological terms and definitions ........ 7**
I.A. Early Iron Age, phase 1 (850 – 250/200 BCE) ........ 8
I.B. Early Iron Age, phase 2 (250/200 BCE – 100/150 CE) ........ 9
I.C. Period of Roman influence (100/150 – 350/400 CE) ........ 11
I.D. Migration period (350/400 – 650/700 CE) ........ 12
I.E. Karayakupovo Horizon (700/750 – 950/1000 CE) ........ 14
I.F. Middle Ages in the Volga-Ural and Trans-Ural regions (950/1000 – 1400/1500). ........ 16
II. The Middle Volga and Lower Kama ........ 18
II.A. Maklasheevka Culture of the Late Bronze Age (LowKama_Maklasheevka) ........ 18
II.A.1 Devichiy Gorodok-4 burial site ........ 19
II.B. The Early Iron Age Post-Maklasheevka culture of the Ananyino area (LowKama_Ananyino) ..... 20
II.B.1. Izmeri-7 burial site ........ 23
burial 5(II) (individual ID I32632, Male) ........ 23
II.B.2. Rysovo-1 burial site ........ 24
burial 2 (individual ID I32630, Male) ........ 24
II.C. The Early Iron Age Pyany Bor culture in the Lower Kama area (LowKama_Pyany-Bor) ........ 24
II.C.1. Tarasovo burial ground ........ 26
burial 731 (individual ID I32623, Female) ........ 27
burial 1133d (individual ID I32624, sex unidentified) ........ 27
II.D. The Migration period Mazunino culture in the Lower Kama area (LowKama_Mazunino) ........ 28
II.D.1. Boyarka ”Aray” burial ground ........ 30
burial 155 (individual ID I32631, Male) ........ 31
II.D.2. Dubrovsky burial ground ........ 31
burial 52 (individual ID I32627, Male) ........ 32
II.D.3. Turaevo-1 burial site ........ 32
burial 66 (individual ID I32629, Female) ........ 33
II.E. The Nevolino culture of the Migration period and Early Medieval in the Lower Kama area (LowKama_Nevolino) ........ 33
I.B.5.1. Sukhoy Log burial ground ........ 35
burial 13 (individual ID I19111, Female) ........ 36
burial 22 (individual ID I19112, Male) ........ 36
II.F. The Early Medieval Novinki-type sites (MidVolga_Novinki) ........ 36
II.F.1. Kurgan groups at Brusyany and Malaya Ryazan’ ........ 38
II.F.1.1. Brusyany-2 kurgan group ........ 38

II.F.1.2. Malaya Ryazan' burial site .......... 38
II.F.2. Novinki-1 kurgan group .......... 39
mound 7, burial 2 (individual ID I16639, Male) .......... 39
mound 8, burial 6 (individual ID I19104, Male) .......... 39
mound 9, burial 1 (individual ID I16640, Male) .......... 39
mound 9, burial 6 (individual ID I19102, Male) .......... 39
mound 12, burial 1 (individual ID I19103, Female) .......... 40
II.G. The Early Volga Bulgaria burials (MidVolga_EVB) .......... 40
II.G.1. Tankeyevka cemetery .......... 42
burial 657 (individual ID I25529, Male) .......... 42
burial 961 (individual ID I25532, Female) .......... 42
burial 1006 (individual ID I25533, Female) .......... 42
burial 1012 (individual ID I25534, Male) .......... 42
II.H. The sites of the Karayakupovo Horizon in the Lower Kama area (LowKama_KH) .......... 43
II.H.1. Bolshiye Tigany burial ground .......... 44
burial 9 (individual ID I25489, Male) .......... 45
burial 19 (individual ID I19105, Female) .......... 46
burial 22 (individual ID I19106, Male) .......... 48
burial 23 (individual ID I19107, Male) .......... 49
burial 25, individual B (individual ID I19108, Male) .......... 49
burial 30 (individual ID I25490, Male) .......... 50
burial 41 (individual ID I19109, Male) .......... 51
II.I. The Medieval Chiyalik culture in the Lower Kama area (LowKama_Chiyalik) .......... 52
II.I.1. Gulyukovo burial site .......... 54
burial 5 (individual ID I19078, Female) .......... 57
burial 17 (individual ID I34074, Male) .......... 57
burial 20 (individual ID I19079, Male) .......... 58
burial 31 (individual ID I25503, Male) .......... 58
burial 51 (individual ID I19080, Female) .......... 59
burial 60 (individual ID I19081, Male) .......... 60
burial 63 (individual ID I33730, Female) .......... 61
II.I.2. Ust-Menzelya hillfort .......... 62
burial 5 (individual ID I33733, Male) .......... 62
II.I.3. Zuevy-Klyuchi hillfort .......... 63
burial 30 (individual ID I30341, Female) .......... 63
burial 34 (individual ID I30342, Female) .......... 63
III. The Middle Kama and Cis-Urals (including the Belaya Basin) .......... 64
III.A. The Early Iron Age Pyany Bor culture in the Southern Cis-Urals (Belaya_Pyany-Bor) .......... 64
III.A.1. Kipchakovo burial site .......... 65
III.A.2. Starokirgizovo burial ground .......... 66
burial 25 (individual ID I16644, Female) .......... 66
burial 42 (individual ID I16645, Male) .......... 67
burial 53 (individual ID I16643, Male) .......... 68
burial 58 (individual ID I16642, Male) .......... 70
burial 63 (individual ID I16641, Male) .......... 71
III.B. The Migration period Mazunino culture in the Southern Cis-Urals (Belaya_Mazunino) .......... 72
III.B.1. Birsk-2 burial ground .......... 74
burial 694 (individual ID I19264, Male) .......... 75

burial 702 (individual ID I19265_d, Male) ... 76
burial 710 (individual ID I19266_d, Male) ... 77
burial 713 (individual ID I19267, Female) ... 78
III.C. The Kushnarenkovo culture (Belaya_Kushnarenkovo) ... 80
III.C.1. Bustanaevo kurgan burial site ... 82
mound 45, burial 1 (individual ID I25531, Male) ... 83
III.D. The Nevolino culture in the Cis-Urals (Cis-Urals_Nevolino) ... 84
III.D.1. Bartym burial ground ... 85
burial 16, individual B (individual ID I19110, Male) ... 86
III.E. The Lomovatovo culture (MidKama_Lomovatovo) ... 87
III.E.1. Bayanovo burial ground ... 89
burial 277 (individual ID I19113, Male) ... 90
III.F. The Polom culture (MidKama_Polom) ... 91
I.C.6.1. Varni burial ground ... 93
burial 158 (individual ID I33979, Male) ... 94
burial 260 (individual ID I33978, Female) ... 96
III.G. The sites of the Karayakupovo horizon in the Cis-Urals (Cis-Urals_KH) ... 96
III.G.1. Karanayevo burial site ... 97
burial 3 (individual ID I25535, Male) ... 98
burial 6 (individual ID I25536, Male) ... 99
burial 8 (individual ID I25537, Male) ... 100
burial 12 (individual ID I25538, Male) ... 101
III.H. The Medieval Chiyalik culture in the Cis-Urals (Belaya_Chiyalik) ... 102
III.H.1. Gornovo burial ground ... 104
III.H.2. Nizhne-Khozyatovo burial ground ... 105
IV. The Trans-Urals and the Western Siberia ... 107
IV.A. The Early Iron Age Sargatka culture (MidIrtysh_Sargatka) ... 107
IV.A.1. Bogdanovo-2 ... 109
IV.A.2. Borovyanka-18 ... 109
mound 13, burial 2 (individual ID I10117, Female) ... 109
mound 3, burial 1 (individual ID I10118, Female) ... 109
IV.B. The Migration period sites of the late phase of the Sargatka culture (MidIrtysh_LSargatka & Tobol_LSargatka) ... 109
IV.B.1. Borovyanka-17 burial ground ... 111
burial 46 (individual ID I6826, Male) ... 111
IV.B.2. Ipkul' kurgan burial site ... 111
mound 13, burial 1, ind. 1 (individual ID I33844, Male) ... 111
IV.B.3. Starolybaevo-4 kurgan burial site ... 113
mound 34, NE sector 1, ind. 1 (individual ID I32779, Male) ... 113
IV.C. The Nizhneobskaya culture (MidIrtysh_Nizhneobskaya & Ob_Nizhneobskaya) ... 114
IV.C.1. Ust-Tara-7 ... 118
mound 9, burial 1 (individual ID I19098, Female) ... 118
IV.C.2. Barsov Gorodok ... 119
burial 235 (individual ID I19095, Male) ... 119
burial 236 (individual ID I19096, Female) ... 120
IV.D. The Potschevash culture (MidIrtysh_Potchevash) ... 120
IV.D.1. Vikulovo ... 121
collective burial 1 ... 122

IV.E. The sites of the Karayakupovo Horizon in the Trans-Urals (Trans-Urals_KH) ........................ 123
IV.E.1. Uyelgi ........................ 125
Kurgan 7, burial 1 (individual ID I19121, Male) ........................ 127
Kurgan 9, burial 5 (individual IDs I19115, Male and I19116, Male) ........................ 128
Kurgan 28, burial 5 (individual ID I19114, Female) ........................ 128
Kurgan 29, burials 1 and 2 (disturbed) (individual ID I19118, Male) ........................ 129
Kurgan 30, infill of the burial mound (individual ID I19119, Male and individual ID I19120, Male) ........................ 130
Kurgan 32, burial 12 (individual ID I19117, Male) ........................ 131
IV.F. The Medieval Ust'-Ishim culture (MidIrtysh_Ust-Ishim) ........................ 132
IV.F.1. Ivanov Mys-1 ........................ 133
mound 10, burial 3 (individual ID I19087, Male) ........................ 134
mound 10, burial 6 (individual ID I19088, Male) ........................ 134
mound 10, burial 9 (individual ID I19089, Male) ........................ 134
mound 12, burial 1 (individual ID I19090, Female) ........................ 134
IV.F.2. Panovo ........................ 135
burial 1 (individual ID I19097, Female) ........................ 135
mound 1, burial 30 (individual ID I19092, Female) ........................ 135
mound 67, burial 3 (individual ID I19093, Male) ........................ 135
IV.G. The Late Medieval burials (Tobol_Tatars) ........................ 135
IV.G.1. Putilovo ........................ 136
unidentified burial (individual ID I10116, Male) ........................ 136
V. The Carpathian Basin ........................ 137
V.A. The Conquest period sites (Early Medieval Magyars of the Carpathian Basin) ........................ 137
V.A.1. Balatonújlak ........................ 138
grave 10 (individual ID I19075, Female) ........................ 139
V.A.2. Harta-Freifelt ........................ 139
burial 9 (individual ID I19077, Female) ........................ 139
burial 19 (individual ID I19070, Female) ........................ 140
V.A.3. Szeged-Öthalom, V. homokbánya ........................ 140
grave 36 (individual ID I19071, Male) ........................ 140
grave 124 (individual ID I19072, Male) ........................ 141
grave 132 (individual ID I19073, Male) ........................ 141
grave 237 (individual ID I19074, Male) ........................ 141
V.A.4. Makó-Igási járandó ........................ 142
grave 5 (individual ID I19099, Female) ........................ 142
grave 8 (individual ID I19100, Male) ........................ 143
V.A.5. Kiszombor C ........................ 143
grave 37 (individual ID I19101, Male) ........................ 143
References ........................ 145

## Supplementary data guide

The supplementary data is integrating archaeological and genetic perspectives to provide cultural-historical context of the newly published individuals. We define the chronological framework of the Circum-Uralic region from the Early Iron Age to the Middle Ages, outlining major cultural horizons and their transformations. Subsequent sections are organized regionally, covering the Middle Volga and Lower Kama, Middle Kama and Cis-Urals, Trans-Urals and Western Siberia, and finally the Carpathian Basin, each introduced with an overview of the key archaeological cultures, their material traditions, settlement patterns, and historical interactions as they are reconstructed based on archaeological evidence. Within these regional blocks, cultures are broken down into site-specific subsections following a consistent format: location and excavation history, characteristic artefacts and burial practices, and a summary of genetic results. The latter detail the number of sequenced individuals, sequencing coverage, suitability for population modelling or IBD-sharing analysis, sex ratios, evidence of relatedness, and affinities revealed by supervised ADMIXTURE and PCA. This integrated approach allows cross-referencing archaeological phases with genetic shifts, while numerous maps and figures place sites in their geographic and cultural context. The document concludes with an extensive bibliography anchoring both the archaeological and genomic interpretations.

# I. Chronological terms and definitions

The chronology of the population dynamics in the Circum-Uralic area over the first millennium BCE - early second millennium CE (~2900-650 yBP) is based on archaeological data and reflects the main stages of the development of the material culture. Despite strong criticism from the last 60 years of archaeological discussion, the term “archaeological culture” still plays an important role as the main research category in the region of study (Klein, 2014; Faifert, 2022). During the last 30 years, it lost its previously dominating "ethnical" sense (Brather, 2004; Klein, 2018) and was partially pushed out from literature by using more diverse definitions such as "archaeological horizon" (a combination of similar features that can be observed at the same time in different cultures) (Swartz 1967; Matveeva N., 2018a) and "cultural type" (an assemblage of cultural features which may not form a clearly defined area of sites, may be represented in a very limited area or even by single site) (Taylor, 1948; Klein, 1979). We summarized in the following sequence the spatial-temporal variations of those archaeological entities that are used for the general periodization of regional history.

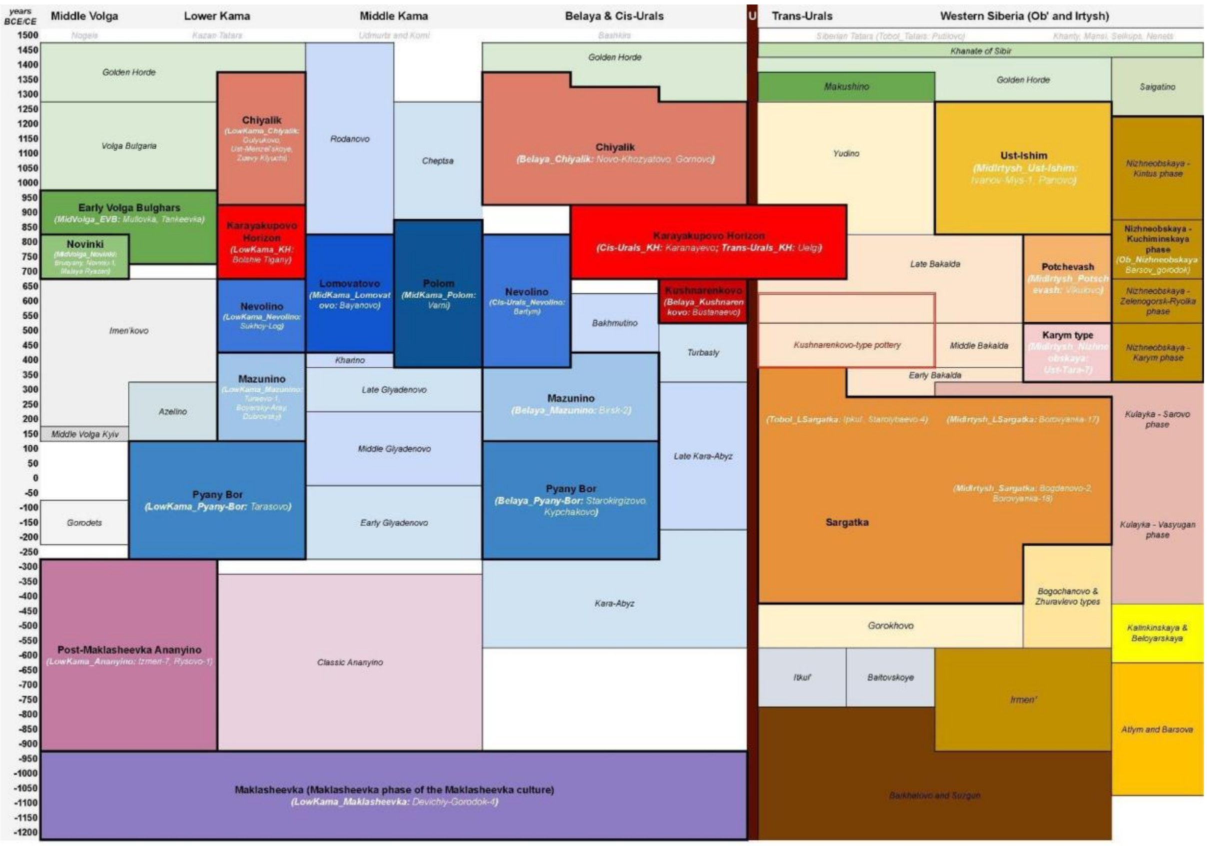


*__Figure SI.1.__ Main archaeological entities of the Circum-Uralic region from the Early Iron Age to the Middle Ages.*

The chronological boundaries slightly differ in different areas of the region and the cultural transformations could last for several decades.

## I.A. Early Iron Age, phase 1 (850 – 250/200 BCE)

The chronological frame of the earliest phase of the Early Iron Age in the Cis-Uralic part of the region is bounded by the cultures of the Ananyino area (Kuz'minykh, 2006; Kuz'minykh, Chizhevsky, 2021), shaped by long-term interaction with the Scythian-period nomads of the steppe region. The upper chronological boundary of the period coincides with the Sarmatian migration and the establishment of the Sarmatian cultures (Prokhorovka) (Chizhevsky, 2020).

***Figure SI.2.*** *Archaeological cultures existed during phase 1 of the Early Iron Age in the Circum-Uralic*

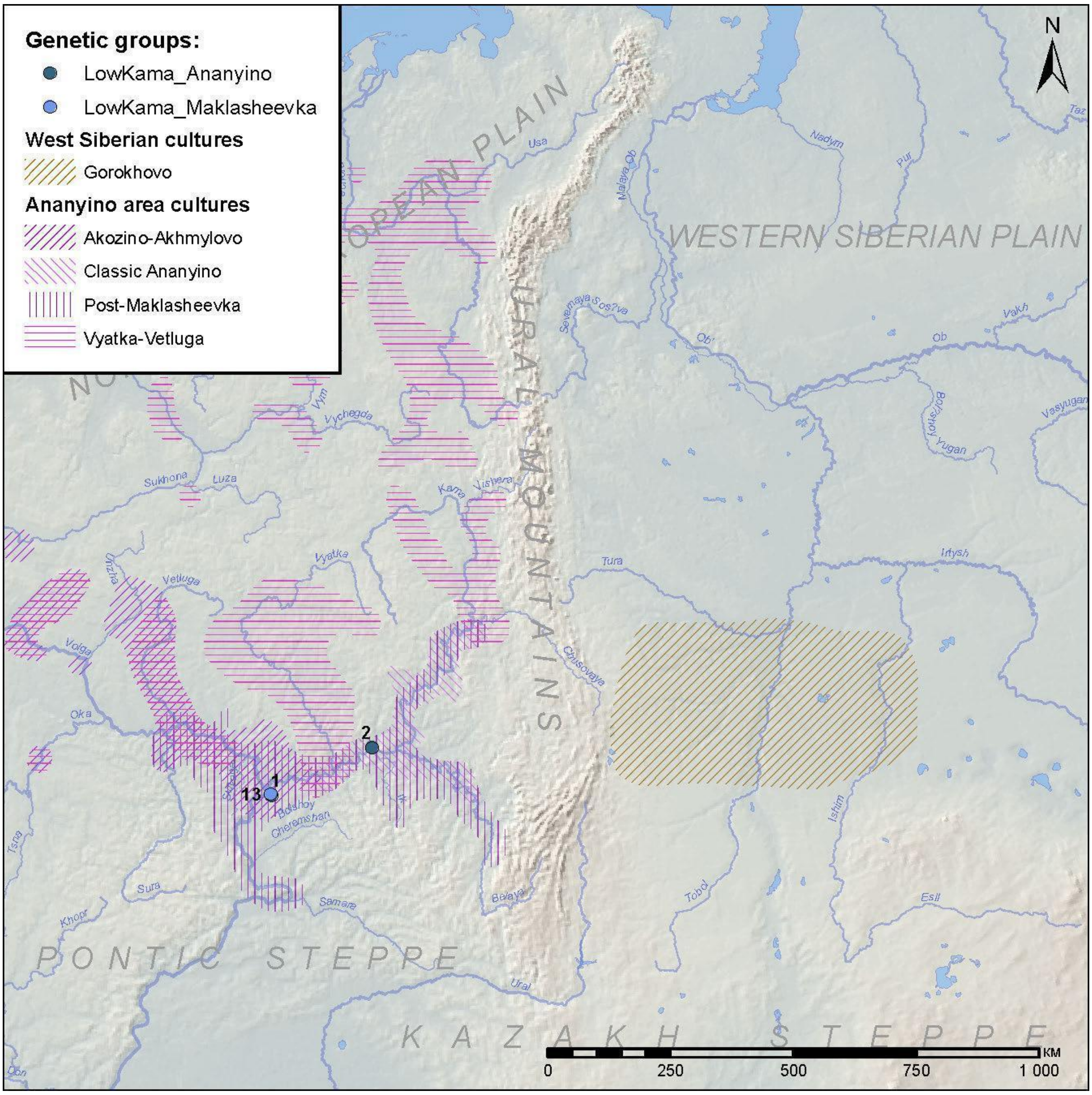


*region and the location of sites where samples were collected.*

In the Trans-Urals, the period is described as the Gorokhovo-Sargatka horizon (Botalov 2016) or as the time of the coexistence of the two cultures, the Gorokhovo and the Sargatka ones (Matveeva, 2019). The end of the period is marked by the disappearance of the Gorokhovo tradition from the Trans-Ural forest-steppe and emergence of its elements in the Cis-Ural-steppe Sarmatian population (Matveeva, 2018a).

To the north of the Gorokhovo and Sargatka area, a distinct kernel of bronze metallurgy developed in the area of the Itkul culture. The artifactual assemblage shared a common cultural subbase with the Ananyino people and interacted with the West Siberian groups which they supplied with metals (Savelyev, 2007).

## I.B. Early Iron Age, phase 2 (250/200 BCE – 100/150 CE)

The second phase of the Early Iron Age in the Circum-Uralic region coincides with the Sarmatian period in the Eurasian steppes. The Sarmatian population intensively interacted with their northern forest-steppe and forest periphery, mediating in trade exchange with the southern craft centers and shaping fashion.

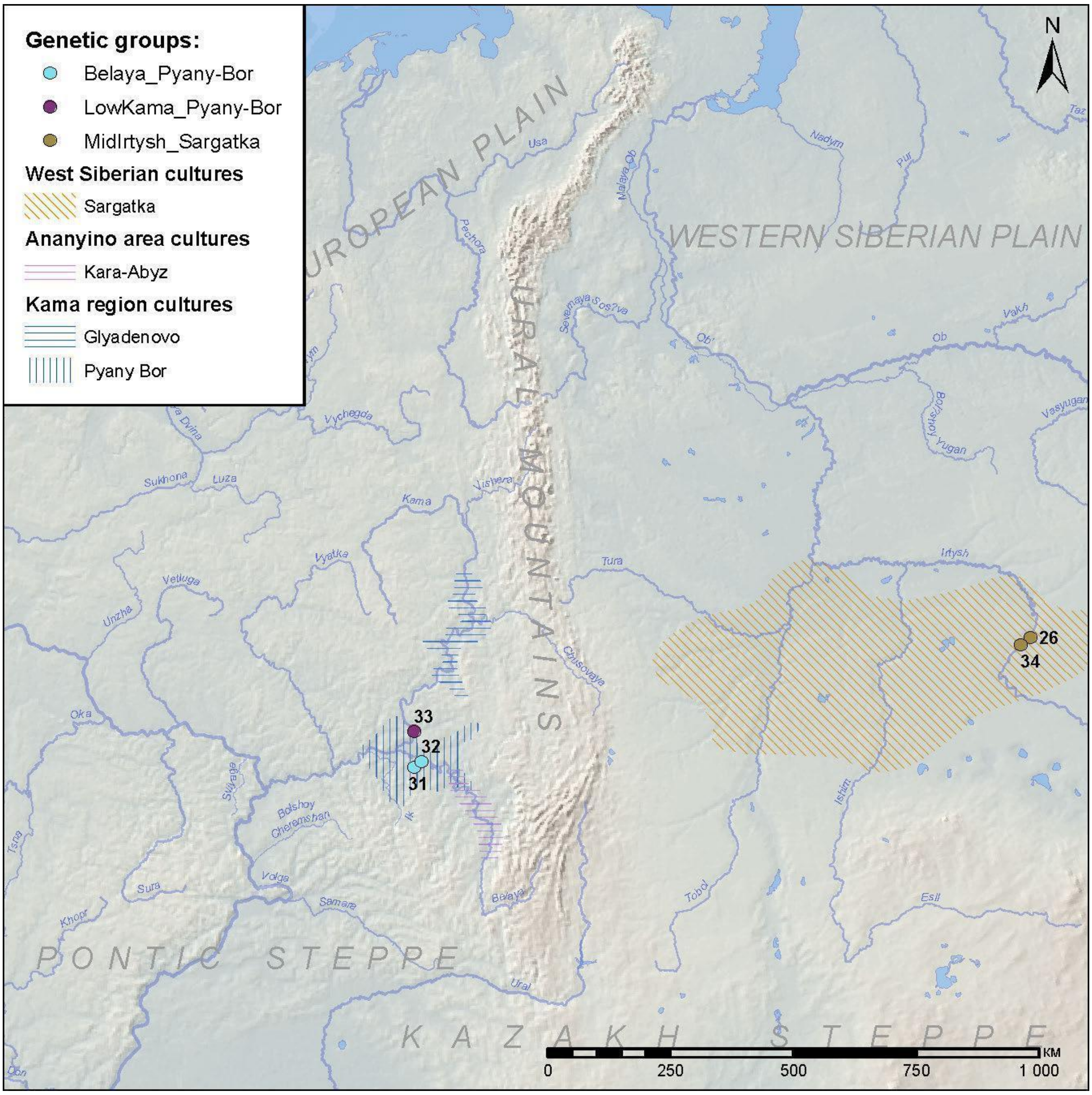


*Figure SI.3.* *Archaeological cultures existed during phase 2 of the Early Iron Age in the Circum-Uralic region and the location of sites where samples were collected.*

The Trans-Urals and the West Siberian forest-steppe during the 2nd phase of the EIA were dominated by the Sargatka people, who reached their peak in social and economic development during that period (Matveeva, 2018a).

In the Cis-Urals, the Lower Kama was occupied by the Ananyino-rooted groups, attributed to the Pyany Bor culture. Further to the north, the Pyany Bor tradition was neighbored by the Glyadenovo one.

In the Mid-Volga, the period is represented by supposedly small populations of the Gorodets and early Old-Mordovian cultural contexts.

## I.C. Period of Roman influence (100/150 – 350/400 CE)

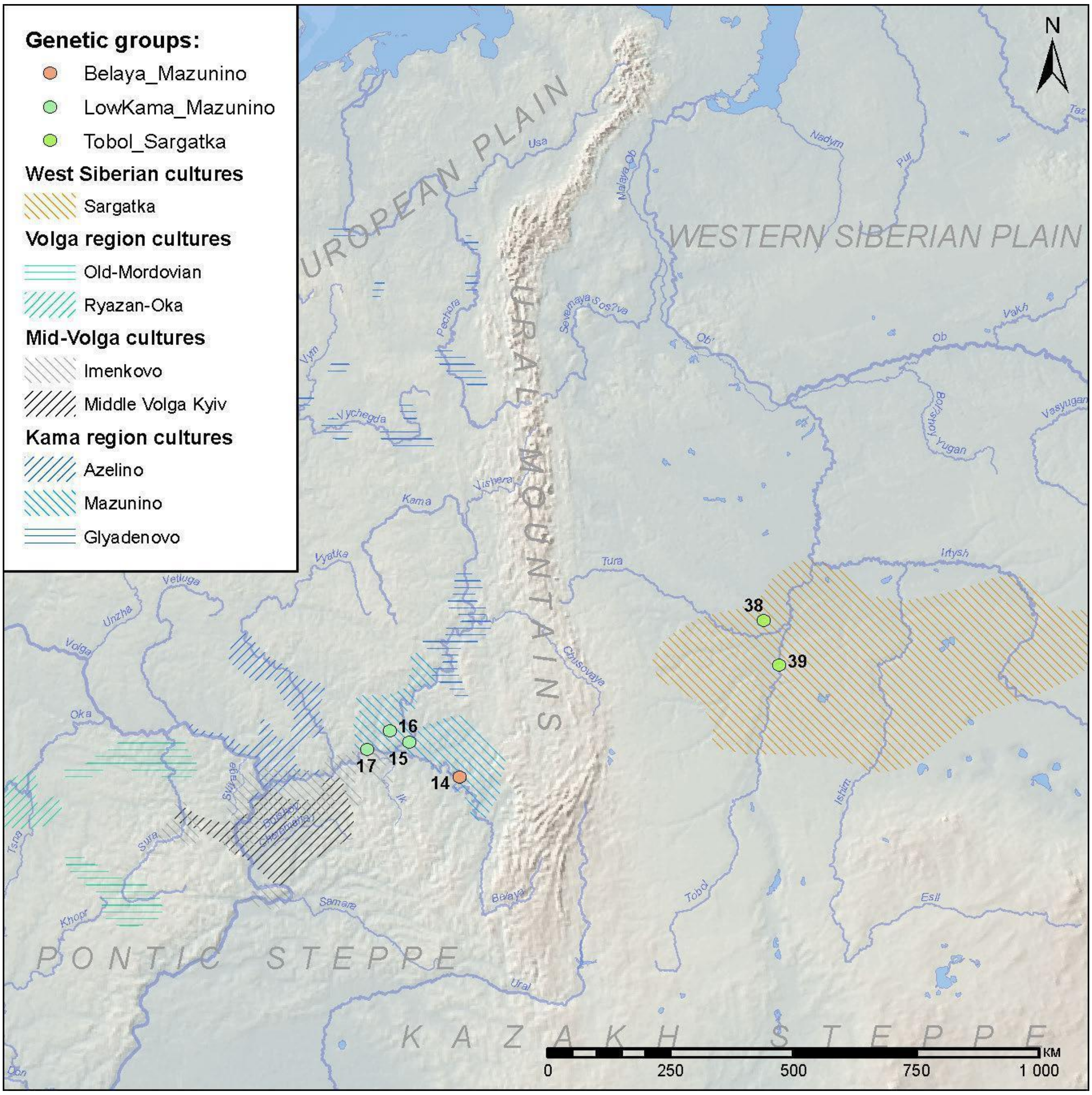


***Figure SI.4.*** *Archaeological cultures that existed during the period of Roman influences in the Circum-Uralic region and the location of sites where samples were collected.*

The period of Roman influence for the Uralic region is a conventional term, marked by the domination of the Late Sarmatian culture in the steppes, which transmitted styles and innovations from the Mediterranean-Caspian civilization to the boreal zones of the Volga and Kama regions. It continued until the profound cultural transformations in Northern Eurasia brought about by the transcontinental migrations of the Huns, along with numerous other groups. In the literature, this period is also referred to as the Late Iron Age.

The distribution of new categories of imports and subsequent development of new local styles is reflected in regional cultural nomenclature, where the period from 100/150 to 350/400 CE is described as the coexistence of two Pyany Bor-derived cultures, the Azelino in the Vyatka basin, and the Mazunino in the Lower Kama and the Belaya basins. In the Middle Kama region, cultural development was more gradual, and the Glyadenovo culture continued to its late phase.

Dramatic changes in the population pattern of the Mid-Volga forest-steppe from 100/150 CE resulted in the formation of several specific cultural groups, possibly reflecting not only cultural transmission but also a migration from the west to the east. All these groups (Kyiv, Early Imen'kovo, and Lbische) intensively interacted with the Late Sarmatian populations of the Volga-Urals.

The cultural attribution and historical dynamics of the 2nd–3rd centuries CE east of the Urals remain debated.

In earlier archaeological periodizations, the 2nd–3rd centuries CE in the Sargatka cultural sequence were generally subsumed under its Late stage, interpreted as a time of gradual decline marked by territorial contraction and increasing cultural heterogeneity (Botalov 2016). This view emphasized the persistence of core Sargatka traits alongside growing influences from southern steppe and northern taiga populations, often framed as the prelude to the culture’s eventual disappearance.

More recent models, informed by refined stratigraphic correlations, radiocarbon dating, and broader interregional comparisons, treat the 2nd–3rd centuries as a distinct terminal phase (Matveeva 2018b). In these reconstructions, the period is characterized by a mosaic of local variants—some maintaining strong Sargatka traditions, others exhibiting substantial admixture with Bakalda and taiga-derived cultural elements, together with a noticeable penetration of Late Sarmatian influences from the East European and South Uralic steppe, visible in weapon types, horse gear, and ornamental styles. These developments reflect both the fragmentation of the original cultural system and intensified cross-cultural interaction across the forest–steppe and taiga–steppe interface (Sharapova, Malashev, 2022).

The cross-Uralic cultural relationships during this period were scarce but already existed. In the Bolshoy Cheremshan River valley, a hillfort was excavated with a Late Sargatka-Bakalda architectural style house, dated to the latest edge of the Roman or the earliest phase of the Migration period (Stashenkov 2020).

## I.D. Migration period (350/400 – 650/700 CE)

The term Migration period is used to cover the Hun (Central European chronology) and post-Hun (Shipovo) periods of the East European steppes and the corresponding phases of development of the local forest-steppe cultural groups. In the Cis-Urals, this archaeological epoch is represented by the classic phase of the Imen'kovo culture in the Mid-Volga, the Late Azelino culture in the Vyatka basin, and the Bakhmutino phase of the Mazunino tradition in the Belaya basin.

In the Mid-Kama basin, the earlier part of the period is usually described as the "Kharino horizon" of the introduction of the Hun-period innovations, while the last stage is marked by the formation of local cultures: the Nevolino, the Polom, and the Lomovatovo.

In the areas to the east of the Urals, we use the term “Migration period” to determine the time span when the interaction of the Late Sargatka and taiga-zone traditions shaped the Bakalda and the Karym-phase Nizhneobskaya cultural groups in the Tobol basin, and the Potchevash one - in the Middle Irtysh area. In the boreal Urals, the period is represented with local population groups, which developed peripheral traditions of the Itkul metallurgists of the Early Iron Age.

Not later than around 550 CE the Cis- and the Trans-Urals manifested an increase in intra-regional cultural connections reflected in the spread of the Kushnarenkovo-type pottery. In the Belaya basin of the western South Urals, sites with the Kushnarenkovo-type pottery form a distinct cultural area, while further west in the Kama region and to the east of Urals it associated with a mixed type of pottery usually found in various contexts (Ivanov, 2008; Zelenkov, 2017). Explanations of these processes differ greatly among various researchers and involve different models of interaction: ethnic, cultural, chronological.

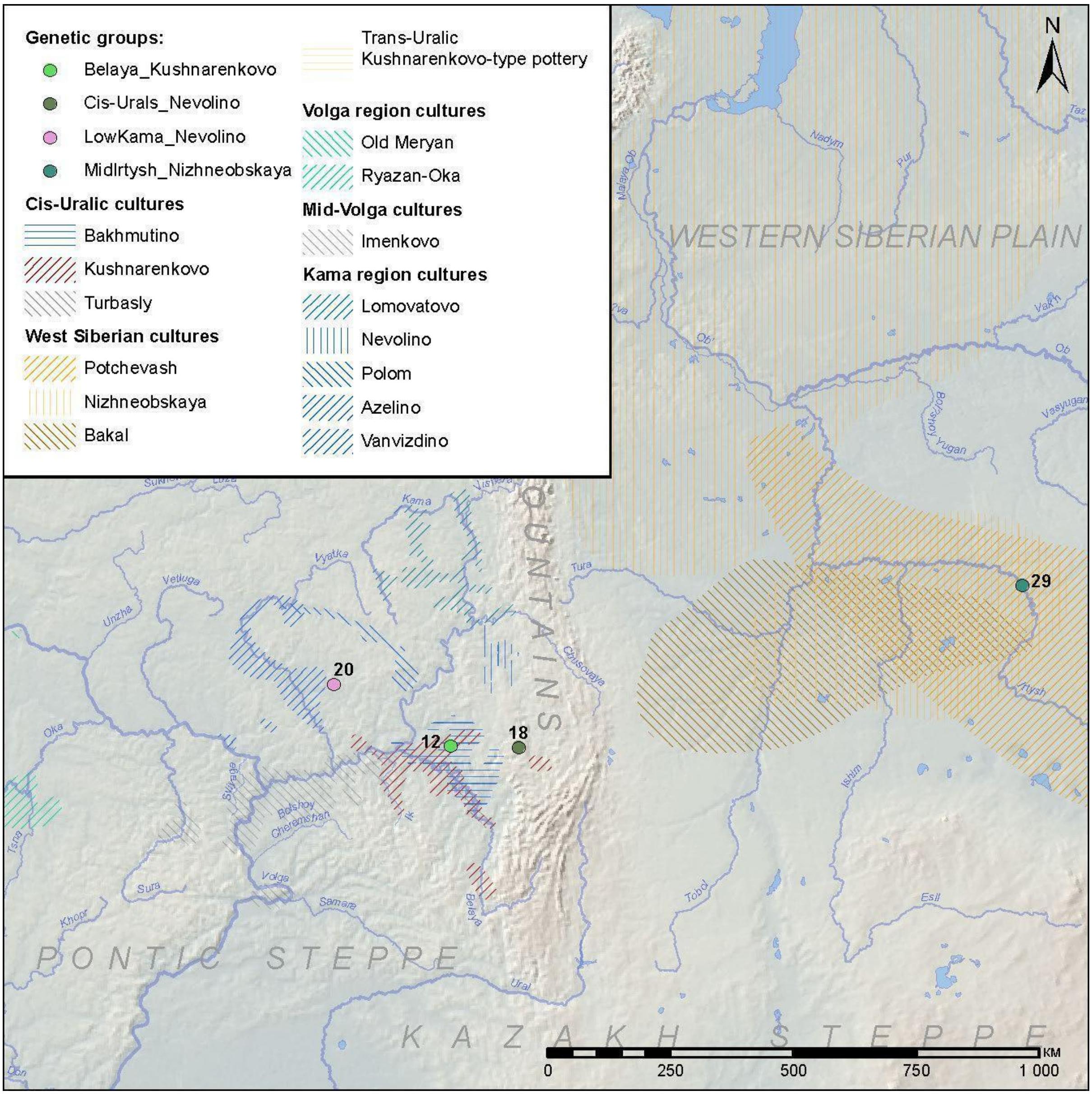


***Figure SI.5.*** *Archaeological cultures existed during the Migration period in the Circum-Uralic region and the location of sites where samples were collected.*

Kushnarenkovo-type ware is often combined in burial complexes with belt decorations of the "heraldic" style of the 6-7th centuries, while such cases are extremely rare for Karayakupovo-type ware. However, in burial grounds of the 8th-9th centuries in the Urals and Kama region, the Kushnarenkovo-type ware did not disappear but evolved into forms with poorer decoration and often combined with the Karayakupovo-type ware.

It is difficult to give an unequivocal answer whether this picture reflects the migration and infiltration of groups of the Karayakupovo population into the Kushnarenkovo area, or whether the spread of Karayakupovo-type ware was the result of some cultural processes. This uncertainty is due, in particular, to the difficulty in determining the original habitat of the Karayakupovo group in the 7th century, which still remains hypothetical. However, the fact of

the very close mutual influence of the Kushnarenkovo and Karayakupovo groups in the 8th-9th centuries prompts researchers to conclude that they are unconditionally related.

Simultaneously with the formation of the Kushnarenkovo Horizon, some traces of cross-Uralic cultural interactions emerge in the boreal parts of the studied region. They are marked by finds of the forest Trans-Uralic comb-pit ware at some Nevolino sites (Pastushenko, 2007), and penetration of the West Siberian population to the west of the mountain ridge.

Since these processes covered a wider area than the actual population centres of the Kushnarenkovo and Karayakupovo groups, it is worth determining the Kushnarenkovo and Karayakupovo horizons (stages) within the archaeology of the Cis-Urals.

## I.E. Karayakupovo Horizon (700/750 – 950/1000 CE)

We use "Karayakupovo Horizon" to refer to a period when a specific trans-Uralic cultural area formed, connecting the Tobol and the Belaya basins and the northern parts of the Kazakh steppe. The detailed archaeological definitions of the period are still discussed (Ivanov, 2022) and the researchers use various terms to define local Circum-Uralic cultural groups of the period (Petrogrom, Karayakupovo, Mryasimovo type, Early Chiyalik, etc.), but their cultural similarity and the proximity to traditions of the Trans-Urals are not disputed.

In the Mid-Volga basin, the formation of the Karayakupovo Horizon was preceded by the collapse of the sedentary Imen'kovo-culture population and the inclusion of the area into a northern periphery of the Khazar Khaganate. The earliest penetration of the nomadic groups that are believed to be warbands of the Khazarian border guards (Novinki type) (Stashenkov, 2013) was followed by a more substantial migration process, commonly associated with the Turkic-speaking Early Bulghars (Vyazov et al., 2019).

The cultural influence of the Khazar Khaganate (reflected in the Saltiv-Mayaky archaeological culture) involved the population of the Lower Kama and the Cis-Urals as well and was reflected in a specific assemblage of the Nevolino and the Polom cultures in their late phases. In the remote forest areas, the local cultural groups were less influenced by the southern steppe styles, and the Lomovatovo culture mostly continued to develop the Kama traditions (Goldina, 2022).

The Samara, Belaya, Upper Ural, and Tobol river basins in 700/750 – 800/850 CE experienced cultural unification and can be described as a core area of the Karayakupovo traditions. Those ones developed the pottery styles that were first attested as the Kushnarenkovo ceramic assemblage, but in contrast to the Kushnarenkovo period, after 650 CE, the Karayakupovo pottery is represented not only as an admixture to the other pottery types but as a dominating one in cemeteries, on both sides of the Urals.

In the 9th century, the population associated with the Karayakupovo traditions and its cultural descendants probably reached its maximum size. The appearance of the Karayakupovo-type ware is observed both to the east of the Ural Mountains in the Uyelgi cemetery (Botalov, 2017), and to the west of the mountain ridge, where such ceramics coexisted with the latest Kushnarenkovo pottery in the Bolshiye Tigany site (Khalikov, 2022). Several single graves in

the Samara Valley part of the Middle Volga region were also attributed to the Karayakupovo horizon (Stashenkov, 2020).

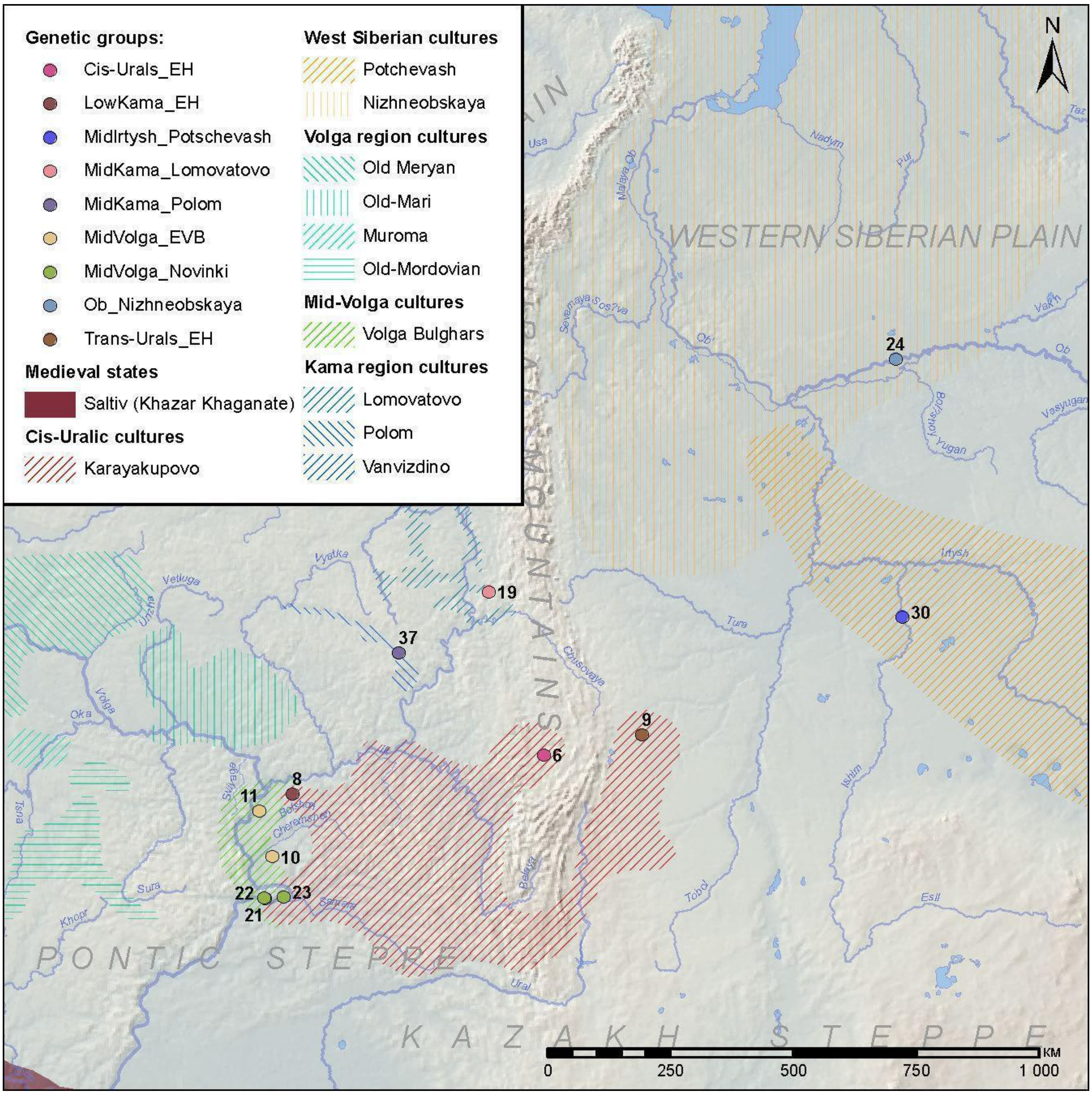


***Figure SI.6.*** *Archaeological cultures existed during the Karayakupovo Horizon period in the Circum-Uralic region and the location of sites where samples were collected.*

The appearance of the Karayakupovo-type ware at this time is observed both to the east of the Ural Mountains, e.g. Uyelgi cemetery (Botalov, 2017), and to the west, up to the Lower Kama region, where such ceramics together with late Kushnarenkovo ware were discovered on the Bolshiye Tigany cemetery (Khalikov, 2022). Also several single graves from the Volga region (in the Samara Valley) to the Karayakupovo-type were attributed (Stashenkov, 2020).

During the 9th century, the cultural influence of the Karayakupovo traditions involved various neighboring populations, including the ones archaeologically represented by the Lomovatovo and Polom cultures, and the Early Bulghars in Tankeyevka.

On the eastern side of the Urals, the cultural development did not experience dramatic changes. In the Mid-Irtysh area, from the 8th century, the influence of the Srostki cultural group on the Potchevash traditions is recorded, but it was weaker in the boreal zone of the Urals, in the areas occupied by the Nizneobskaya and, later, Yudino cultural areas. The influence of Srostki in also sufficient in Uyelgi, where it effectively prevails in the complex of horse equipment and belt decorations. In the Southern Urals, this impulse also dominates from the second half of the 9th century, completely displacing both the Saltiv influence and local types of bridle and belt decorations.

By the 10th century, the common Circum-Uralic cultural environment formed by the Karayakupovo Horizon started to disintegrate. This process was possibly driven by the Early Hungarian migration to the Carpathian basin and the occupation of the North Kazakh steppe by the Central Asian nomads, usually attested to as Oghuzs and Patzinaks (Ivanov, 2022) or Kimaks and Kipchaks (Botalov, 2017). In the southern Cis-Urals of the second half of the 9th century - the 10th century the picture of the influence of both the Srostki culture and the Oghuz-Patzinaks culture is observed in burial complexes, while the specific Ural ware and personal ornaments gradually disappeared during the 10th century. These processes likely mark the Turkization of the region’s population.

## I.F. Middle Ages in the Volga-Ural and Trans-Ural regions (950/1000 – 1400/1500).

Due to the asynchronous socio-political development of the main part of Europe and the Volga-Ural region, we conventionally start the Medieval period with the establishment of

Volga Bulgaria in the Lower Kama and the Mid-Volga basins in 900/950 CE. This turning point coincides with the start of the decline of the Karayakupovo Circum-Uralic environment and the formation of continuous and stable cultural groups in the Cis- and Trans-Uralic boreal zones. The development of these groups continued mostly gradually, with a catastrophic breaking point caused by the Mongolian invasion in the 1230s, which, however, impacted mostly urban centers and southern regions on the border of the steppe.

From the 10th century onwards, the cultural development of the Circum-Uralic area was highly influenced by the centers of technology and craft that emerged at the confluence of the Kama and the Volga. The main innovations included the spread of arable farming and trade; from the 10th century, the Great Volga trade route started connecting the remote areas of the East European North with Iran and Central Asia.

Despite the formation of Volga Bulgaria, the Belaya River basin and the eastern part of the Low Kama region were not developed by sedentary agriculturalists, and the traditions of urban life were not introduced there. Those areas were occupied by the people of the Chiyalik culture, commonly interpreted as descendants of Karayakupovo-horizon populations. The chronological frame of the Chiyalik tradition covers both the pre-Mongolian and the post-Mongolian eras.

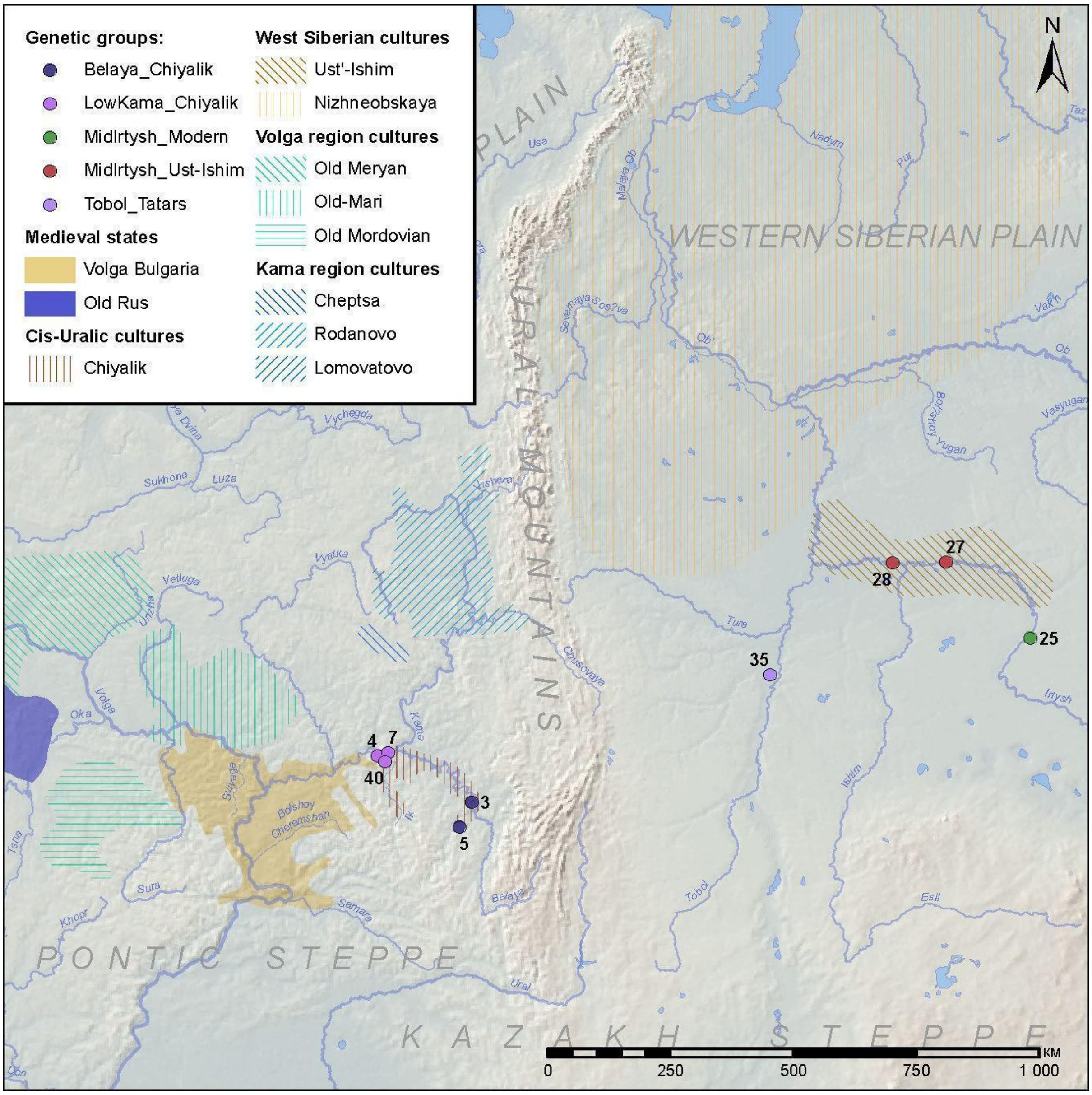


***Figure SI.6.*** *Archaeological cultures existed in the Middle Ages in the Circum-Uralic region and the location of sites where samples were collected.*

In the Mid-Kama region, the cultural tradition called Rodanovo derived from the Lomovatovo, and the Cheptsa culture succeeded the Polom one.

Only during the gradual collapse of the Golden Horde in the 15th century, peripheral populations of the former imperial space transformed into the ethnic groups recorded by the late medieval and early modern travelers and naturalists: the Kazan Tatars, Bashkirs, Udmurts, Khanty, and Mansi.

***Archaeological sites labelled on the maps Fig. S2-7: 1 - Izmeri-7; 2 - Rysovo-1; 3 - Gornovo; 4 - Gulyukovo; 5 - Novo-Khozyatovo; 6 - Karanayevo; 7 - Zuyevy-Klyuchi; 8 - Bolshie-Tigany; 9 - Uyelgi; 10 - Mullovka; 11 - Tankeyevka; 12 - Bustanaevo; 13 - Devichiy-Gorodok-4; 14 - Birsk-2; 15 - Boyarsky-Aray; 16 - Dubrovsky; 17 - Turaevo-1; 18 - Bartym; 19 - Bayanovo; 20 - Sukhoy-Log; 21 - Brusyany; 22 - Malaya-Ryazan'; 23 - Novinki-1; 24 - Barsov-Gorodok; 25 - Borovyanka-17; 26 - Borovyanka-18; 27 - Ivanov-Mys-1; 28 - Panovo; 29 - Ust-Tarsk; 30 - Vikulovo; 31 - Kipchakovo; 32 - Starokirgizovo; 33 - Tarasovo; 34 - Bogdanovo-2; 35 - Putilovo; 36 - Mellyatamak-3; 37 - Varni; 38 - Ipkul'; 39 - Starolybaevo-4; 40 - Ust-Menzelya.***

# II. The Middle Volga and Lower Kama

## II.A. Maklasheevka Culture of the Late Bronze Age (LowKama_Maklasheevka)

The Maklasheevka culture is divided into two phases: the early Atabaevka phase and the later Maklasheevka phase. The chronology of this culture is based on relative and absolute radiocarbon dating. The early phase is dated to the 14th–13th centuries BCE, while the later phase spans the 12th–10th centuries BCE (Chizhevsky, Lyganov, Kuzminykh, 2021).

**Genetic results.** *We analyzed 1 individual from the site of Devichiy-Gorodok-4 (n=1). This male had sufficient coverage for downstream population genetic modeling (>100,000 SNPs), and was suitable for IBD-sharing analysis (>600,000 SNPs). It shows eastern European genetic affinity and the supervised ADMIXTURE analysis revealed an elevated level of Samara_EBA_Yamnaya and Lithuania_EMN_Narva ancestries in this individual. The LowKama_Maklasheevka samples clustered within the no cluster. Even if it was one sample from the site, this Late Bronze Age/Early Iron Age sample showed a different genetic composition compared to other later time period samples in the region.*

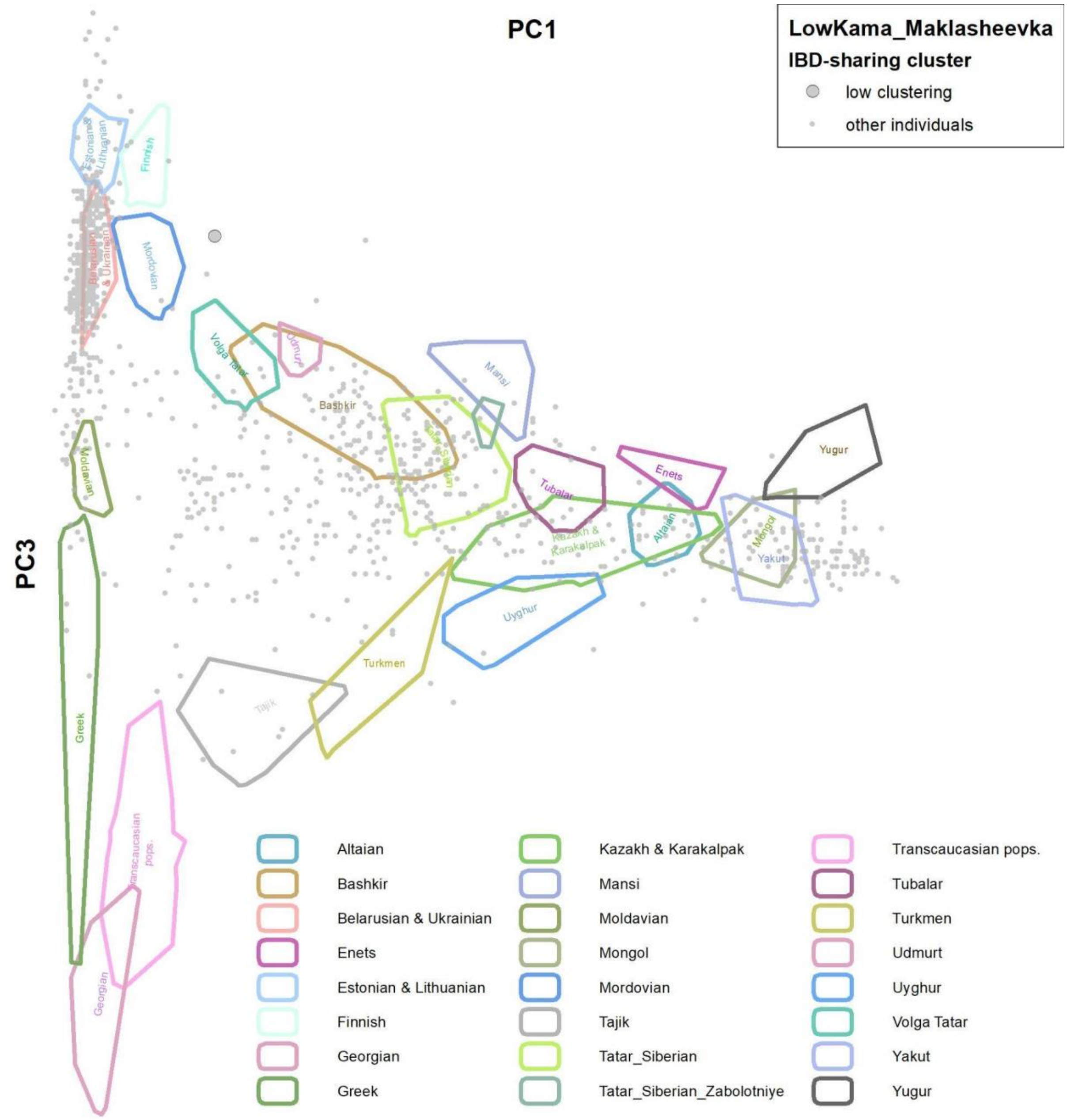


***Figure SII.1.*** *Eurasian-scale principal component analysis (PCA), with a projection of the newly sequenced individuals onto modern genetic variation following Jeong et al. 2019. The PC1 and PC3 dimensions are shown, including the newly presented genomes. Polygons represent example populations from modern Eurasian individuals onto which the ancient samples were projected, while grey dots indicate ancient reference samples from our IBD-sharing network.*

## II.A.1 Devichiy Gorodok-4 burial site

The Devichy Gorodok-4 burial site, attributed to the later phase of the Maklasheevka culture, is located 3.8 km west of Izmeri village on the edge of a second terrace, 7–8 m high, along the Kuibyshev Reservoir, which has been affected by erosional processes. Excavated over four years (1981, 1985, 1989, 2001) by E.P. Kazakov, this small burial site (approximately 20 graves) has significantly altered our understanding of the funerary traditions of the Maklasheevka culture. Unique finds, including four articulated horse hooves deposited in the

grave fill and a bone bridle set, were recorded for the first time (burial 16). The bridle set includes three-hole rod-shaped *psalia*, common in the region, along with other elements such as rings and a distributor, crafted from horse shuttle bones (Kazakov, 2002; Chizhevskiy et al., 2019).

One individual from Devichy Gorodok-4 burial site (**burial 21**, individual I32628, male) was sequenced for this study.

## II.B. The Early Iron Age Post-Maklasheevka culture of the Ananyino area (LowKama_Ananyino)

The first archaeological finds later attributed to the Ananyino group of cultures were made during the excavation of the Ananyino burial mound in 1858 (Alabin, 1860). The accumulation of data significantly increased with preparations for the 4th Archaeological Congress in Kazan (Russian Empire) in 1878 and the establishment of the Society for History, Archaeology, and Ethnography at Kazan Imperial University. In the 20th century, scholars such as A.V. Zbrueva (1952), A.Kh. Khalikov (1977), and V.S. Patrushev (1984) synthesized earlier findings and developed cultural reconstructions related to Ananyino artifacts.

S.V. Kuzminykh (2000), V.N. Markov (1988, 2007), and A.A. Chizhevsky (2008) introduced revised concepts of the Ananyino cultural complex, emphasizing its heterogeneity. These models indicate that the term "Ananyino" encompasses territories where four distinct cultural groups evolved from different Late Bronze Age cultures. By the early Iron Age, these groups converged into a single cultural formation characterized by shared metallurgical and funerary traditions. Although each group's ceramic assemblage retained its specific features, some ornamental styles were shared across the cultural area (Kuzminykh, Chizhevsky, 2009; Kuzminykh, et al., 2024).

**1. Post-Maklasheyevka culture.** The Post-Maklasheevka culture occupied the Middle Volga region, including modern Mari El Republic, Samara Luka, the Lower Kama, and the Belaya River valley. This culture developed from the Maklasheevka culture of the Terminal Bronze Age. Its defining feature was bronze socketed axes (celts) with lenticular or oval socket sections (Ananyino type). The culture exhibited a tradition of placing stone stelae on burial sites, often adorned with depictions of weapons such as celts, axes, swords, and daggers. Throughout its existence, Post-Maklasheevka pottery was characterized by pit ornamentation. By the 7th century BCE, cord impressions began to decorate vessels. During the late period (5th–3rd centuries BCE), ornamentation became sparse, with ceramics featuring rare cord impressions accompanied by pits (Chizhevsky, Volkova, 2021).

**2. Akozino-Akhmylovo culture.** The Akozino-Akhmylovo culture spread across modern Chuvashia, Mari El, and the lower Oka River region, extending into Kostroma and Tver Volga areas. This cultural complex demonstrates a mix of elements from the "textile pottery" cultural zone of Central Russia and traits of the Maklasheevka culture of the Volga-Kama region, with a dominant western component. The Akozino-Akhmylovo complex is characterized by celts with long tubular sockets and a single ear (Akozino-Mälar type), as well as ceramics with textile or net stamp impressions forming a background pattern overlaid with pit, comb, and cord ornamentation (Chizhevsky, Kuzminykh, Patrushev, 2021).

**3. Classic or the Corded Ware Ananyino culture.** This culture developed from the Middle Kama variant of the Maklasheevka culture of the Final Bronze Age, preserving traits of the early Atabaevka phase in ceramic design. The Classical Ananyino culture was compactly situated in the Middle Kama region. It is characterized by Ananyino-type celts with hexagonal socket sections. During the early phases (8th–7th centuries BCE), its ceramics featured multi-row cord impressions and depictions of hoofed animals using cord and comb stamps. In later phases, ornamentation simplified to pits along the neck of vessels. Bronze "ceremonial" Ananyino axes with wolf heads on the butt and predatory birds on the socket's upper part are also distinctive (Chizhevsky, et al, 2021).

**4. Vyatka-Vetluga, or Comb-Cord Ware Ananyino culture.** The Vyatka-Vetluga culture occupied a vast region from the Vetluga and Vyatka Rivers in the south to the Pechora and Vychegda basins in the north. This group's complex derives from the Lebyazhskaya culture of the Late Bronze Age, prevalent in modern Komi Republic and northeastern Europe. Defining features include Ananyino-type celts with hexagonal socket sections but with distinct ornamentation compared to the Classical Ananyino culture. Ceramics were decorated with comb and cord patterns, along with pits. Early ceramics were richly ornamented, while later stages saw simpler designs, mainly pits on the vessel neck. The culture also featured a highly developed bone industry, with many artistic items crafted in the animal style. Unlike other Ananyino-related cultures, iron smelting was less prevalent, with colored metallurgy and metalworking dominating (Chernykh, Chizhevsky, 2021).

**5. Chronology of the Ananyino cultures.** The chronology of the Ananyino area is based on relative dating and absolute radiocarbon dates.

*Early Period (9th–first half of the 7th century BCE)*: Synchronized with pre-Scythian sites of the steppe zone of Eastern Europe and the Caucasus, known from early nomadic burials in the Black Sea and pre-Caucasian steppes (Kuzminykh, Chizhevsky, 2014).

*Middle Period (second half of the 7th–5th centuries BCE)*: The first stage aligns with the end of the Early Scythian period, the Kelermes stage of Scythian culture, and the Senior Scythian period. The second stage corresponds to the Middle Scythian stage and the Sauromatian archaeological culture.

*Late Period (end of the 5th–3rd centuries BCE)*: Correlates with Late Scythian and, partly, Classical Scythian antiquities, as well as the entire existence of the Prokhorovka (Early Sarmatian) culture (Chizhevsky, 2017).

The Ananyino area ended with its transformation into several post-Ananyino cultures, including the Pyany Bor, Kara-Abyz, and Glyadenovo cultures (Chizhevsky, 2020).

**Genetic results.** *From the Ananyino culture, we analyzed two individuals from the sites of Rysovo-1 (n=1) and Izmeri-7 (n=1). Both individuals had sufficient coverage for downstream population genetic modeling (>100,000 SNPs), but none of them were suitable for IBD-sharing analysis (>600,000 SNPs). No close relatedness was detected among these samples, and the female-to-male ratio was 1:1. The sample from Rysovo-1 displayed a genetic composition similar to the local Iron Age group, while the sample from Izmeri-7 showed affinity to Northeastern Europe. The supervised ADMIXTURE analysis revealed elevated levels of Samara_EBA_Yamnaya and Yakutia_LNBA/Russia_EHG ancestries in this group.*

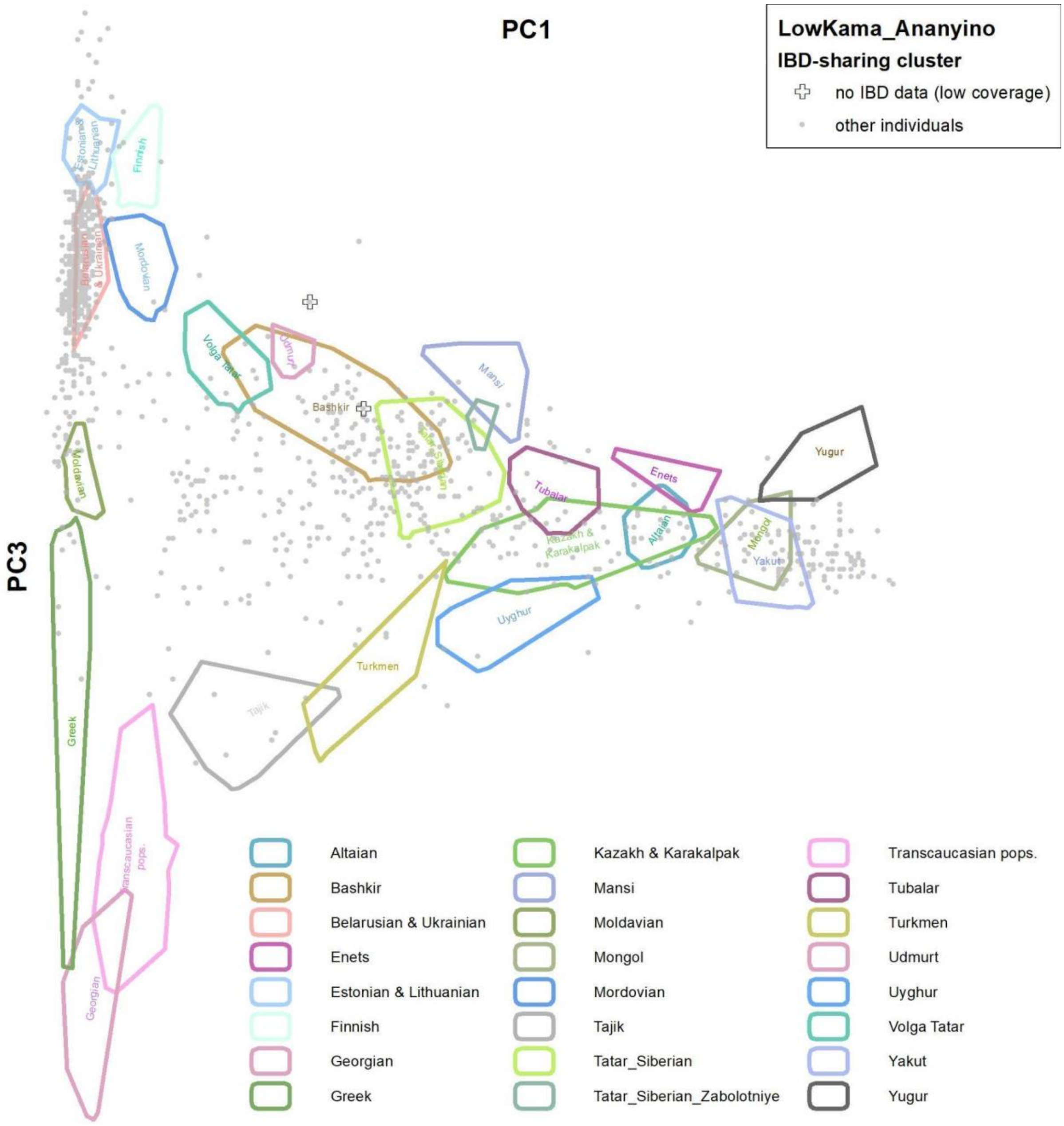


***Figure SII.2.*** *Eurasian-scale principal component analysis (PCA), with a projection of the newly sequenced individuals onto modern genetic variation following Jeong et al. 2019. The PC1 and PC3 dimensions are shown, including the newly presented genomes. Polygons represent example populations from modern Eurasian individuals onto which the ancient samples were projected, while grey dots indicate ancient reference samples from our IBD-sharing network.*

## II.B.1. Izmeri-7 burial site

The site is located 2 km northwest of Izmeri village in the Spassky District of Tatarstan and 3.5 km northeast of the Devichy Gorodok-4 burial site. Positioned on the edge of a promontory overlooking the extensive floodplains of the Kama and Volga Rivers, now submerged under the Kuibyshev Reservoir, the site was discovered by E.P. Kazakov in 1985 and excavated between 1986 and 1990. Most of the burial site has been destroyed by the reservoir.

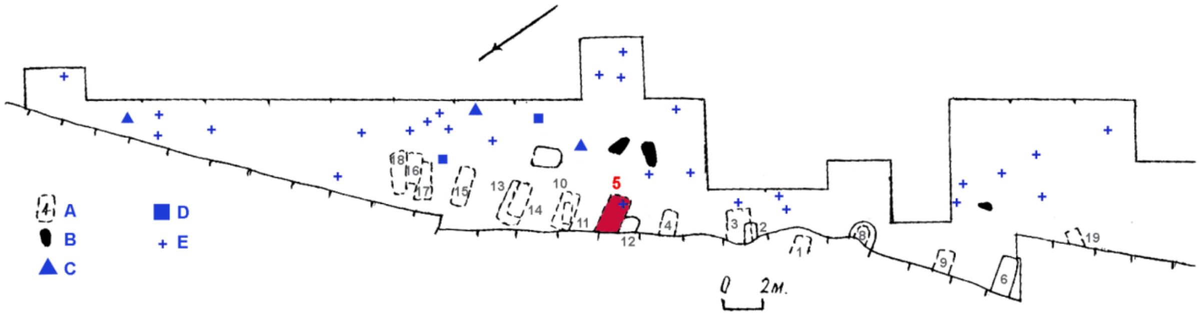


***Figure SII.3.*** *The burial ground Izmeri-7 (16, 17). A plan of the excavated area. The burial 5 is marked in red (image contributed by Andrey Chizhevskiy).*

Designated variously as Izmeri-7, Izmeri-16, or Izmeri-17 in publications, the site occupies the eroded edge of a second floodplain terrace rising 8–9 m above the reservoir. Artifacts from disturbed graves were collected by Kazakov along the shoreline, and profiles of exposed grave pits were documented. Approximately 30 graves were excavated over a 283 m² area (Kazakov, Lyganov, 2014; Kazakov, 2017). An additional paired burial (No. 20) was excavated 20 m northeast of the primary burial area.

From burial 5(II) at Izmeri-7, one individual (I32632, male) was sequenced.

### *burial 5(II) (individual ID I32632, Male)*

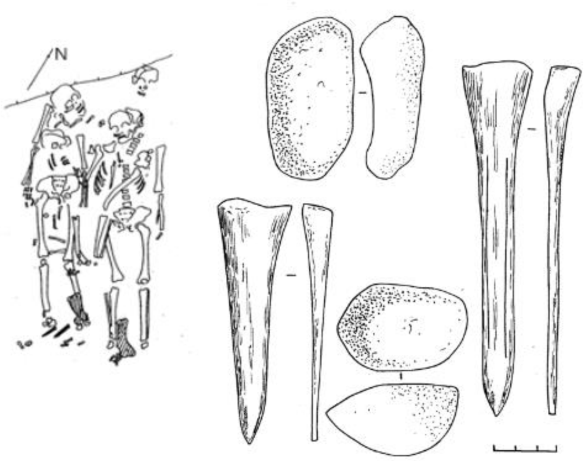


In the collective burial 5, the bones of a child and an adult were placed above two adult skeletons. The upper layer of skeletons represents a secondary burial, with the position and orientation of these remains differing from those in the lower layer (Kazakov, 1994).

***Figure SII.4.*** *Burial 5 at Izmeri-7 site (image contributed by Andrey Chizhevskiy).*

## II.B.2. Rysovo-1 burial site

The Rysovo archaeological complex was discovered in 1985 by E.P. Kazakov (Rysovo-1 burial site on an island) and V.N. Markov (Rysovo-3 settlement along the reservoir). Since 1995, A.A. Chizhevsky has conducted annual surveys of Rysovo sites, discovering the Rysovo-2 cemetery in 2001.

Rysovo-1 is situated 8–8.5 km southwest of Salaush village in the Agryzsky District of the Republic of Tatarstan, 3 km southwest of Rysovo-3 settlement, and 12 km east of Izhevka village in the Mendeleevsky District. Positioned on a second floodplain terrace 8–10 m above

the Nizhnekamsk Reservoir at the confluence of the Izh and Kama Rivers, the site became an island after the reservoir's formation.

In the eastern part of the island, E.P. Kazakov discovered burial 1 in 1985, containing a vessel attributed to the Ananyino area. A.A. Chizhevsky later discovered burial 2 (Chizhevsky et al., 2004; Chizhevsky et al., 2014).

### *burial 2 (individual ID I32630, Male)*

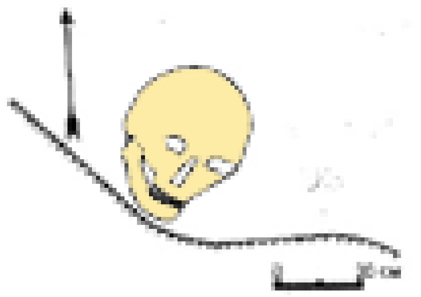

The grave pit boundaries were not identified. At a depth of 55 cm, a skull was found, oriented with the head to the northeast. Postcranial bones were destroyed by shoreline erosion. The individual may have been buried on their back with feet toward the river.

***Figure SII.5***. *Burial 2 from Rysovo-1 site.*

The skull was examined by anthropologist I.R. Gazimzyanov, who identified the remains as a young woman who died from cervical vertebrae damage. No burial inventory was found in situ, but a cast bronze plaque depicting a griffin with a loop on the reverse side was recovered below the cliff. Since the ceramic vessel in burial 1 belongs to the Ananyino cord-impressed pottery tradition, burial 2 is also attributed to the Ananyino period.

## II.C. The Early Iron Age Pyany Bor culture in the Lower Kama area (LowKama_Pyany-Bor)

The first findings later attributed to the Pyany Bor culture became known in 1878. A significant volume of materials was acquired as a result of A.A. Spitin's extensive expedition throughout the Vyatka Province in 1898. Initially, all post-Scythian period sites in the Perm Cis-Urals were unified within a single culture. It was subsequently designated as an independent culture by A.V. Schmidt in the 1920s. Key consolidations were achieved through the dissertation research of V.F. Gening, B.B. Ageev, D.G. Bugrov, and R.R. Sattarov.

**Burial grounds** have been extensively studied, they are flat and have an average size of 200-400 graves. Burials were arranged in rows, in shallow, often simple pits, sometimes accompanied by poorly preserved wooden structures of uncertain form. Adjacent to the skeletons (extended in a supine position), numerous costume ornaments (worn during life) were found, alongside tools and weapons.

Finds and features of **settlements** have scarcely been described, largely due to the peculiarities of layer formation and the poor methodologies of early excavations. Economic aspects remain understudied, although evidence suggests advanced animal husbandry and hunting practices. Agriculture, while probable, lacks detailed investigation. Pottery vessels are handcrafted, round-bottomed, and ornamented with belt-like rows of pits around the neck.

**Chronology.** The core of this culture's formation lies within a local group of sites in the lower and middle reaches of the Syun' River (a left tributary of the Belaya River, itself a left tributary of the Kama River). These sites are challenging to date precisely. The Ik burial ground, located significantly apart from the "core," provides insights into early complexes but poses difficulties in attribution. The inception of the culture may be traced to 150 BCE. A chronological gap exists between it and the preceding Ananyino cultures. A distinct period of peak development for the classical Pyany Bor culture is evident in 200-225 CE, while the delineation of materials from 100 BCE to the 50 CE is more intricate. A substantial portion of the 1st century BCE likely comprises unadorned burials, identifiable primarily through their planimetrics.

During 200-250 CE, some of the population remains in their previous habitation areas, gradually transforming into the Mazunino culture. Simultaneously, another segment shifts westward, founding sites associated with the Azelino culture.

**Genetic results.** *In this group, we analyzed two individuals from the site of Tarasovo (n=2). Of these, one had sufficient coverage for downstream population genetic modeling (>100,000 SNPs), while neither sample met the higher threshold for IBD-sharing analysis (>600,000 SNPs). No close relatedness was detected among these samples, and we identified one female. The single sample with sufficient coverage displayed genetic similarities to other Low Kama Iron Age groups. The supervised ADMIXTURE analysis revealed elevated levels of Samara_EBA_Yamnaya and Yakutia_LNBA ancestries.*

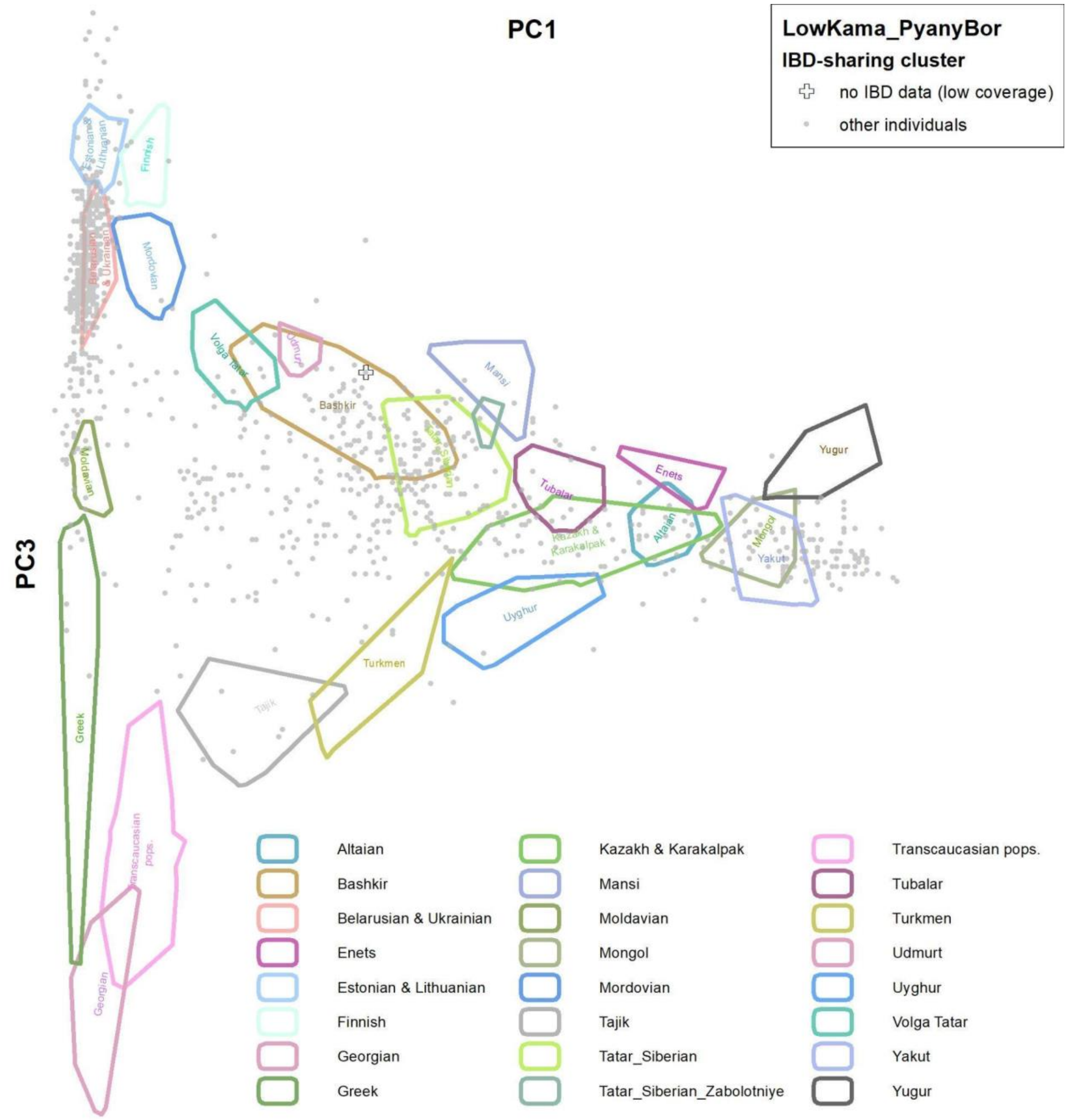


***Figure SII.6.*** *Eurasian-scale principal component analysis (PCA), with a projection of the newly sequenced individuals onto modern genetic variation following Jeong et al. 2019. The PC1 and PC3 dimensions are shown, including the newly presented genomes. Polygons represent example populations from modern Eurasian individuals onto which the ancient samples were projected, while grey dots indicate ancient reference samples from our IBD-sharing network.*

## II.C.1. Tarasovo burial ground

Tarasovo village, Karakulinsky District, Udmurt Republic. Located on a high promontory of the right bank of a stream, a tributary of the Tarasovka River, a right tributary of the Kama River. Discovered by N.L. Reshetnikov in 1979 during the installation of an oil well, 1 km southeast of the village of Tarasovo. The site was excavated from 1980 to 1997 by the Kazan Military-Archaeological Expedition under the direction of R.D. Goldina. A total of 1880 burials were studied over an area of 16,000 square meters. This is the largest Finn-Ugric burial ground of

the first half of the 1st millennium CE. The site represents a transition from the early Cheganda culture to the Mazunino culture. Burials are arranged in rows with varying orientations. Numerous weapons were found, including swords, spearheads, and arrowheads, as well as defensive items such as helmets, armor, and chainmail. Some burials contained "sickles-humps". Ornaments include plaques, brooches, overlays, pendants used for headwear, chest ornaments, belts, and footwear accessories, as well as clothing adornments. Many different types of fasteners were found, including buckles, buckles with fixed hooks, belt loops, and butterfly fibulae.

### *burial 731 (individual ID I32623, Female)*

The pit has a flat bottom and is oriented along the northeast-southwest line. The upper part was damaged during the installation of an oil well. At the bottom, poorly preserved skeletal remains of a female individual 25-30 years old were found, suggesting that the deceased was placed lying on her back, with her head to the northeast. No artifacts were found.

### *burial 1133d (individual ID I32624, sex unidentified)*

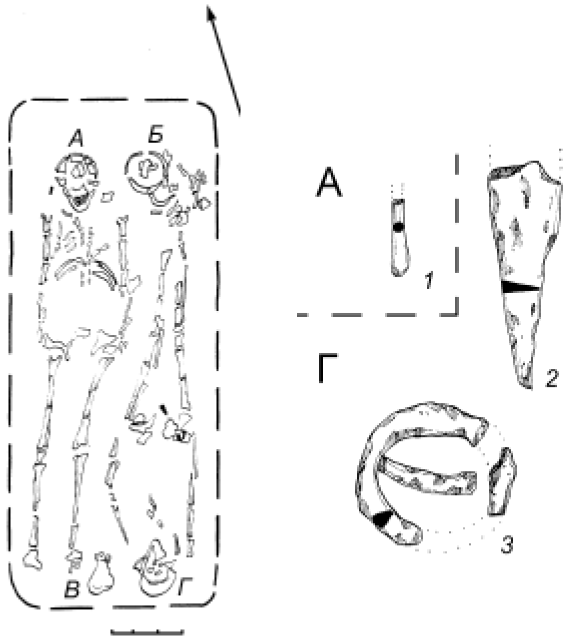


The pit has a flat bottom and is oriented along the southwest-northeast line. The upper part of the pit was damaged by plowing. At the bottom, four poorly preserved skeletal remains were cleared.

Skeleton A: the deceased was laid out lying on her back, arms along the body, head to the northeast. A fragment of a bronze object was found to the right of the skull bones. Skeleton B: the buried individual was laid to the left of buried A; the preserved skull allows determining the orientation of the deceased - head to the northeast. No artifacts were found.

***Figure SII.7.*** *Burial 1133d at the Tarasovo cemetery.*

Skeleton C: only the skull remains, indicating that the deceased was laid with the head to the southwest, between buried A and D. No artifacts were found. Skeleton D: the deceased was laid out lying on her back, arms along the body, head to the southwest. An iron buckle was found on the pelvic bones, on the right side, and a fragment of an iron knife was found between the femur bones.

Anthropologically, the excavated skeletons were determined as: A - female, 40-50 years old; B - sex?, 8 years old; C - sex, age?; D - female, 25-30 years old.

## II.D. The Migration period Mazunino culture in the Lower Kama area (LowKama_Mazunino)

The earliest finds of the artifacts later attributed to the Mazunino culture were first described in the late 19th century, and extensive excavations, culminating in a systematic description of the cultural assemblage, started in the second half of the 1950s (Gening, 1967). The unearthed data was summarized at the turn of the 1970s/1980s (Ostanina, 1983), but the results (Ostanina, 1998) were only published 15 years later without updates (Ostanina 1997).

The earliest manifestations of the elements of the Mazunino assemblage are recorded at the late Pyany Bor sites located on the right bank of the Kama River opposite the mouth of the Belaya and dated to 150-200 CE by the Sarmatian-derived elements of belt sets. One can find there the earliest forms of Mazunino-type temple pendants, a rite of placing a belt in the grave not worn by the deceased but stretched along the body develops, characteristic "butterfly-shaped" fibulae evolved from “Sarmatian” prototypes (with a coil at the end of the receiver, Ambroz-13 group).

In 200-250 CE, the Pyany Bor tradition gradually declined, while the Mazunino traditions strengthened. Overall, by the mid-3rd century CE, a classic set of all ritual and inventory features were developed.

The settlement clusters comprise hillforts of two distinct types: smaller ones characterized by insignificant cultural layers, and larger ones with thicker layers, alongside unfortified settlements. Fortifications may entail multiple rows of ramparts and ditches. The habitation areas within these settlements feature rectangular on-surface houses equipped with hearths and cash pits.

Pottery is predominantly handmade, incorporating a mixture of bird droppings, crushed shells, sand, dry clay, and calcined bones in the pottery clay. Vessels typically exhibit flattened round bottoms and rims, occasionally adorned with notches and pinches, while their bodies are embellished with round, square, or triangular corded stamps, arranged in one or several horizonal lines, or less frequently, in clusters of pits.

Cemeteries are characterized by flat grave pits sized according to the sex and age of the deceased, arranged in rows and groups. Skeletons are typically placed in supine positions, primarily within individual graves, occasionally within wooden coffins, or covered by timber overlays or logs. Collective burials have also been documented, and interpreted as family graves. Costume details and jewelry are often found on the bodies of the deceased, although belts are frequently aligned along the body. A common feature of burial practices involves the placement of decorations and small tools in separate niches (referred to as "mortuary assemblage"). Additionally, small vessels were sometimes included as part of grave goods. Finds of weapons and tools are infrequent, predominantly consisting of imported swords and helmets, with approximately 10-30% of burials lacking any inventory.

Costume decorations of the Mazunino culture include various items such as temple pendants, chainmail braid covers, torques, necklaces made of beads and threads, fibulae (including later, more specific types like "butterfly"-shaped, curved "bow"-shaped, and abundantly decorated

rectangular-shaped), belt sets, numerous pendants and overlays, as well as knife sheaths typically featuring protrusions and pendants.

The economy of the Mazunino population was primarily based on livestock farming and beaver hunting, likely supplemented by slash-and-burn agriculture.

Around 300-350 CE, the cultural assemblage of the Mazunino underwent significant transformation, possibly triggered by the breakdown of interactions with the steppe Sarmatian population. There was a decline in the number and variety of bronze decorations, while iron artifacts began to increase in quantity. The newly introduced artifacts continued the stylistic development of Late Sarmatian prototypes, which had influenced Mazunino craftsmanship in the period leading up to the breakdown of communication. Concurrently, Late Sarmatian-style weapons disappeared, replaced by common types from the Migration period, which became more numerous and prevalent.

By 300 CE, the Mazunino population had vacated the right bank of the Kama River, with no documented finds of Mazunino-attributed artifacts dating from 300-450 CE in that area. However, a group of sites in the Belaya River valley continued to be inhabited, and influences from the Mazunino tradition persisted in the Middle Kama region (Nevolino culture) and the Cheptsa basin (Polom culture), as well as sporadically in Western Siberia.

In the later stages of evolution, the Belaya Mazunino group introduced innovations in pottery production, resulting in new vessel shapes such as ribbed bodies adorned with stamp decorations (referred to as the "Imendyashevo" type) and ceramics featuring numerous, irregularly arranged or row-like notches across the vessel body (known as the "Bakhmutino" or "Chandar" type) (Ovsyannikov, Sungatov 2004). These innovations coexisted with traditional elements of the cultural assemblage, suggesting ongoing cultural development within the population. However, the origins of these pottery-making innovations remain unclear. The most recent burials associated with the Mazunino tradition date back to 500-550 CE. During this final stage of cultural development, there was a gradual replacement of traditional styles with more "fashionable" items.

It is noteworthy that certain regional archaeological schools tend to identify distinct cultures within the Belaya Mazunino group, primarily based on variations in pottery styles (Belyavskaya, Protsenko, 2018). However, these approaches overlook the typological consistency observed in other categories of artifacts, suggesting the cultural coherence of the Mazunino Belaya group, despite the presence of numerous specific ceramic types.

**Genetic results.** *In this group, we analyzed four individuals from the sites of Turaevo-1 (n=2), Dubrovsky (n=1), and Boyarka "Aray" (n=1). All the samples had sufficient coverage for population genetic modeling (>100,000 SNPs) and one was suitable for IBD-sharing analysis (>600,000 SNPs). The female-to-male ratio was 1:3. Supervised ADMIXTURE analyses revealed elevated Samara_EBA_Yamnaya and Yakutia_LNBA ancestries. The one sample suitable for IBD-sharing had low clustering coefficient.*

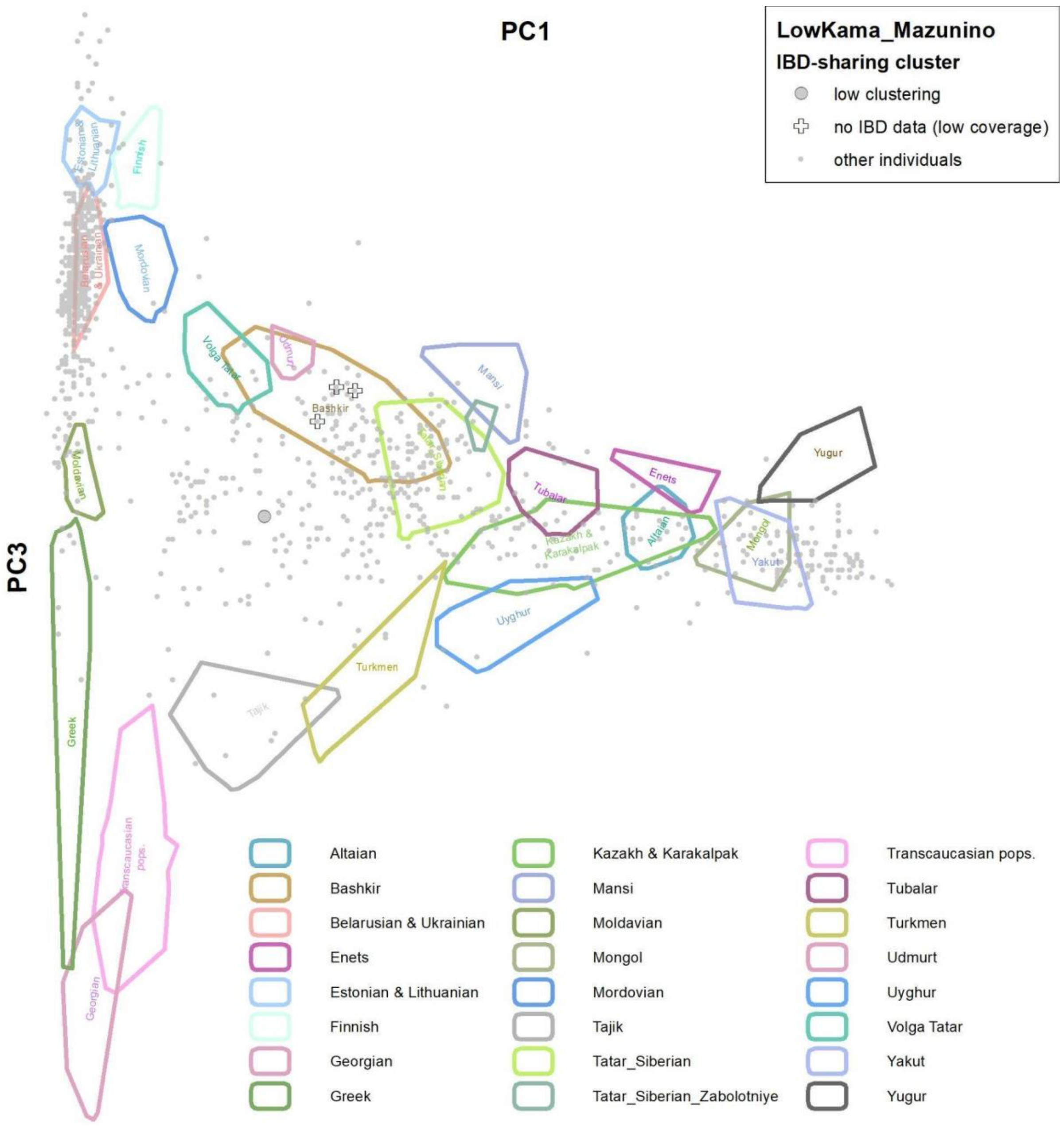


***Figure SII.8.*** *Eurasian-scale principal component analysis (PCA), with a projection of the newly sequenced individuals onto modern genetic variation following Jeong et al. 2019. The PC1 and PC3 dimensions are shown, including the newly presented genomes. Polygons represent example populations from modern Eurasian individuals onto which the ancient samples were projected, while grey dots indicate ancient reference samples from our IBD-sharing network.*

## II.D.1. Boyarka ”Aray” burial ground

The site is located 4 km northeast of the village of Boyarka (Karakulinsky district, Udmurt Republic) and 5 km south of the Kama River, on an elongated promontory of the river bank known as "Aray." The site surface is flat and gently slopes southeastward, measuring approximately 50 m in length and 10-15 m in width, with steep, actively eroding slopes, and ravine bottoms overgrown with forest.

In 2002, N.L. Reshetnikov from the Sarapul Museum collected artifacts from citizens of Boyarka, including iron knives and their fragments, iron temple pendants with bronze wrapping, and 2 bronze “butterfly” fibulae. Rescue excavations at the site were initiated by E.M. Chernykh of Udmurt University, leading to the discovery of 183 burials.

***burial 155 (individual ID I32631, Male)***

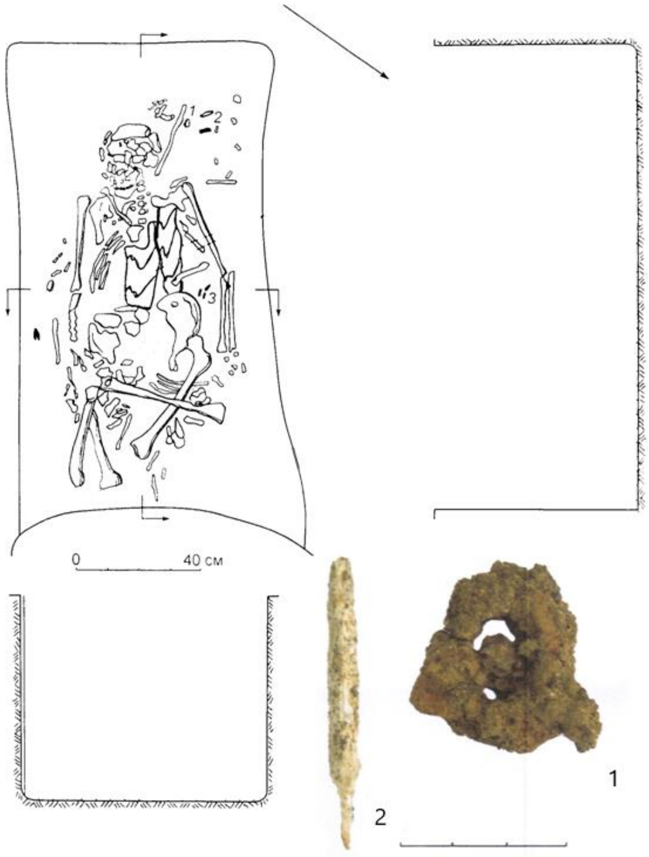


The grave pit found at a depth of -30 cm was oriented along the southwest-northeast line. The northeastern part of the grave pit was disrupted by burial 156. The surviving portion of burial 155 had a rectangular shape with slightly concave long walls, measuring 150x80 cm and reaching a maximum depth of 96 cm. At a depth of 90 cm, small traces of decayed organics up to 0.3 cm thick were found. The skeletal remains of the deceased were unearthed at the bottom of the grave pit.

Initially, the deceased was placed supine with the head to the northeast, arms alongside the body.

***Figure SII.9.*** *Burial 155 at the Boyarka “Aray” cemetery.*

The bones of the upper half of the skeleton were in correct anatomical order except for the rib cage, part of which was found near the feet of the deceased. The leg bones were shifted towards the pelvis, with the tibiae seemingly detached and placed on the femurs. The radius of the right arm of the deceased and several smaller bones of the skeleton were found to the left, near the skull, with the sternum occupying the position of the radius of the right arm.

In the area of the skull, an iron buckle and a bone arrowhead were found on the left side. A fragment of an iron object of uncertain purpose, which disintegrated during cleaning, was found near the pelvic bones.

## II.D.2. Dubrovsky burial ground

The Dubrovsky burial ground is located 0.5 km south of the settlement of Dubrovsky and 1 km south of the village of Kalashur in the Kiyasovsky district of the Udmurt Republic, on an elevated promontory of the terrace on the left bank of the Shekhostanka River. At this location, the river makes a sharp loop, creating whirlpools and rapids in the channel. A total of 204 burials have been excavated at the site.

***burial 52 (individual ID I32627, Male)***

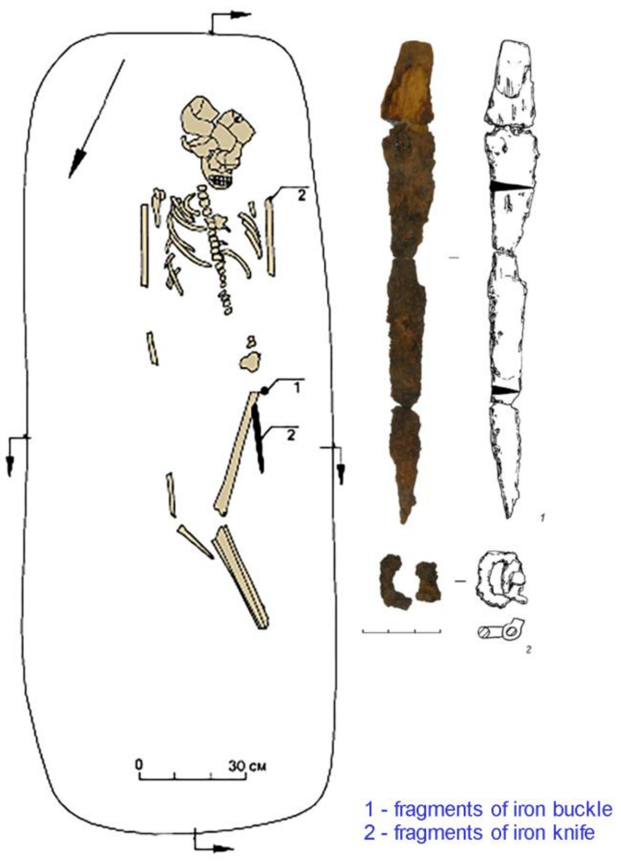


The grave pit (excavated in two years, 2010 and 2012) was unearthed at -30 cm from the modern surface. The shape of the pit was rectangular with rounded corners and a slight widening in the northern half, measuring 224x85 cm. The walls of the pit were vertical to the south and west, and inclined to the north and east. The bottom was flattened along the longitudinal axis and flat along the transverse axis. The orientation of the pit was along the southwest-northeast line. The maximum burial depth was -65 cm from the modern surface. At the bottom of the grave, closer to the southern and western walls, a skeleton of satisfactory preservation was cleared.

***Figure SII.10.*** *Burial 52 at the Dubrovsky cemetery.*

Based on the position of the bones, it can be assumed that the deceased was laid out supine with the right arm along the body, and the legs bent at the knees to the left so that the shins touched. The head was facing south-southeast. Discoveries at the bottom included a fragment of an iron buckle near the right thigh and an iron knife along the femur below the buckle.

## II.D.3. Turaevo-1 burial site

The burial site is located on the right bank of the Chukma River (also known as Chemek), a right tributary of the Kama River, on an elevated plateau of the terrace gradually descending towards the Kama River, not far from it, to the southwest of the village Turaevo (Mendeleevsky District, Republic of Tatarstan). The cemetery includes two parts, a group of kurgans excavated by Vladimir Gening in 1969, and a flat part, excavated by N.V. Vodolago from 1986 to 1990. The latter excavation of an area of 2688 square meters yielded 269 burials attributed to the Mazunino culture. The burials are arranged in rows and oriented along the southwest-northeast line. Numerous artifacts were found near the skeletons, including belts laid along the body of the deceased, pendants, beads, overlays, bracelets, fibulae of local forms, and clay vessels.

In this paper, we publish two individuals buried in the flat graves of Turaevo, one from burial 66 (ID I32629) and another one from unidentified burial (ID I32621, Male).

***burial 66 (individual ID I32629, Female)***

Anthropologically identified as female, 18-20 years old. The grave pit had sloping ends, vertical walls, a flat bottom, and was oriented along the southwest-northeast line. Judging by the poorly preserved skeletal remains, the deceased was laid out supine, arms along the body, with the head to the southwest. To the left at the head of the grave, a mortuary assemblage was found, consisting of fragments of bronze spiral-threaded pins (7 fragments preserved), glass beads (5 specimens), fragments of fused bronze and iron chains, an iron awl with fragments of a wooden handle (attached to it was a fragment of a bronze spiral-threaded pin), and an iron knife with fragments of a wooden handle and a bronze pin; underneath the items, fragments of birch bark (a round box) were found. In the shoulder area (to the right and left of the mandibula), four iron temple pendants with bronze wrapping were discovered (two specimens on each side). The sleeves of the deceased's garment were adorned with large round bronze pins "beads" (116 specimens preserved), arranged in the area of the arms from the shoulders to the elbows; fragments of fabric and fur were also found there. Bronze and iron necklaces with bronze wrapping lay in the chest area. Additionally, slightly below the necklaces, an iron three-part fibula with bronze hemispheres, iron and bronze pendant chains, was found. Four bronze pins (pipe pins), zoomorphic: a bear pin and two duck pins, were attached to the chains, as well as six pendants, five of which were bronze (3 lobed), one trapezoidal and one zoomorphic pendant (horse), and one square iron pendant. The deceased wore three bronze bracelets with flattened ornamental ends on her arms: two specimens on the right arm and one on the left (found in the hip area). Furthermore, to the left along the body of the deceased, from the chest to the feet, a unfolded leather belt was laid. Preserved from it were an iron buckle with six bronze rivets in the chest area, bronze overlays (a total of 85 specimens), and an assembled tip made of four iron plates (each with four bronze rivets) with four iron pendants; the tip lay between the shin bones. Of the 85 bronze belt overlays, one had two bronze pins, twelve had three bronze hemispheres (nail caps), and the remaining 72 overlays had two bronze hemispheres (nail caps) and a bronze ring. The total recorded length of the belt was at least 95 cm, and the width of the leather base of the belt was 3.0-3.5 cm.

## II.E. The Nevolino culture of the Migration period and Early Medieval in the Lower Kama area (LowKama_Nevolino)

Finds later attributed to the Nevolino culture are known from the mid-19th century. The eponymous site, Nevolino burial ground, was studied in the 1920s and 1950s (materials were published unsystematically). Extensive field research, nearly fully published to date, led to the substantiation of the characteristic features of the site group, conducted by Rimma Goldina (Goldina & Vodolago 1990).

The sites attributed to the Nevolino culture usually form spatial clusters which include hillforts and unfortified settlements. In the occupation area, separate homesteads are distinguishable, often arranged in rows. Houses were small, semi-subterranean or slightly dug into the ground, square or rectangle with rounded corners in shape. There were two round (diameter 4-5 m) sunken structures with 1-2 main pillars in the center. Sometimes, pillar constructions and porches can be traced. A hearth constructed with stones was usually located in a corner of a dwelling. Pottery was handmade and round-shaped; by proportions, there were pot-shaped, bowl-shaped, and dish-shaped vessels; with or without neck; paired handles-ears were rare.

In later periods, the number of ornamented vessels increased; the upper part was decorated with notches, pinches, cord impressions, comb stamps, depressions, incised lines forming bands of horizonal lines, zigzags, fir trees, nets. There were wooden vessels with metal inlays, including those with zoomorphic handles, made on a potter's wheel from southern centers, including the Saltiv-Mayaky culture, and others. Burial mounds (small mounds with ring ditches) around the beginning of the 7th century were replaced by flat cemeteries, where graves formed groups and rows. Deceased were placed in rectangular pits, often in wooden coffins or frames; laid out on the back, the predominant orientation varied across necropolises. The inventory was mainly arranged in the order of wearing, but belts could be stretched along the body, and some items were placed in boxes. Male burials often contained weapons, horse gear (bridles could also be found with women). Artificial cranial deformation is known. There were many pendants in the decorations. Belt set arrangements are typical for the Perm region. Female belts, dated to the second half of the 7th-8th centuries, with wide pendant strips, abundantly decorated with overlays resembling an inverted heraldic shield with slits resembling a face, Ж-shaped overlays, round overlays, "triplets," slotted tips, and others, are attributed to the specific “Nevolino type”. A relatively large number of weapons and horse gear sets distinguish the Nevolino culture from other traditions of the Upper and Middle Kama regions. About 20 hoards with metal utensils and coins (mostly Sasanian silver; there are also from Central Asia, Byzantium), jewelry, and metal ingots are known. The economy was based on agriculture (slash-and-burn), animal husbandry (horses, cattle, less sheep and goats, few or no pigs), hunting (furs were important), and crafts (Goldina, Vodolago, 1982).

Five phases of the development were distinguished by Rimma Goldina, they are as follows: “Brody”, late 4th-5th centuries; “Verkh-Saya” (Goldina, Perevozchikova, 2012; Goldina, 2018), mainly 6th century; “Bartym” (Goldina, et al., 2011), late 6th-7th centuries; “Nevolino”, late 7th-8th centuries; “Sukhoy-Log”, late 8th - 1st half of the 9th centuries (Goldina, 2012).

Some elements of the Nevolino pottery derive from the traditions of the Glyadenovo population. The Mazunino component may have had some influence as well. In the mid-9th century, marked by the spread of Saltiv types, the territory became deserted. In the late stage, the spread of typical Nevolino ornaments (belts, pendants) over a vast territory to Finland, Sumy Oblast of Ukraine, Omsk Oblast of Russia, and Kazakhstan was recorded. Sites directly left by late Nevolyinsk groups spread southeast of the original territory, to the headwaters of the Ufa River with its tributaries (settlements of Rakhmangulovo, Abdulinsky, Rakhmangulovskoe, Tornalinsky, Bolshoustikinsky hillforts, Kzytausky, Yangatausky settlements) and southwest (settlements Osh-Pando-Ner, Podgory-1) (Karavashkina, 2017).

**Genetic results.** *In this group, we analyzed two individuals from the site of Sukhoy-Log (n=2). These samples had sufficient coverage for downstream population genetic modeling (>100,000 SNPs) but not for IBD-sharing analysis (>600,000 SNPs). No close relatedness was detected among these samples, and the female-to-male ratio was 1:1. The samples exhibited distinct genetic characteristics compared to other Low Kama groups, with detectable West Eurasian (likely Caucasian or Eastern European) gene flow. The supervised ADMIXTURE analysis revealed elevated levels of Samara_EBA_Yamnaya and Turkey_N ancestries in this group. In contrast to Cis-Urals Nevolino-associated individuals, these samples displayed a different ancestral composition, suggesting gene flow from the Caucasian, Pontic Steppe, or Eastern European regions.*

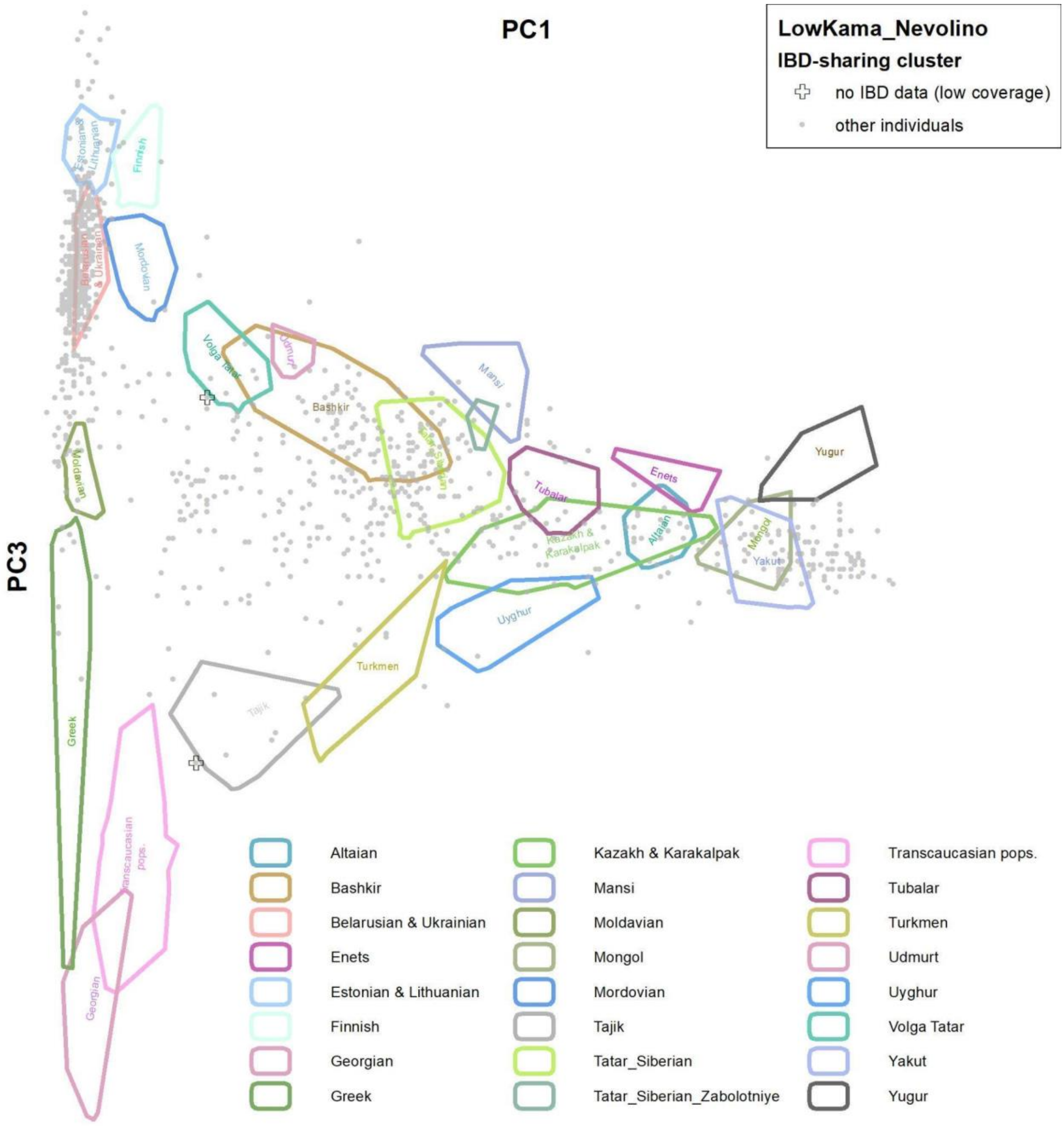


***Figure SII.11.*** *Eurasian-scale principal component analysis (PCA), with a projection of the newly sequenced individuals onto modern genetic variation following Jeong et al. 2019. The PC1 and PC3 dimensions are shown, including the newly presented genomes. Polygons represent example populations from modern Eurasian individuals onto which the ancient samples were projected, while grey dots indicate ancient reference samples from our IBD-sharing network.*

## I.B.5.1. Sukhoy Log burial ground

The Sukhoy-Log burial ground is located in the Kishertsky District of the Perm Krai, on the right bank of the Sylva River, a left tributary of the Chusovaya River, which is in turn a left tributary of the Kama River. The site is situated on the southeastern outskirts of the village of Sukhoy Log, on a high 17-meter terrace of the right bank of the Sylva River. At this location, the terrace takes on the shape of a rectangular promontory, formed by the steep bank of the river and a depression perpendicular to it, transitioning into a hollow that cuts through the

terrace and defines the platform of the site from the west. To the east, the terrace gradually rises. The platform of the site is occupied by buildings and gardens of the modern village.

The burial ground was discovered in 1986 by E.E. Kirzhner. A trench with an area of 9 m2 was excavated at the edge of the terrace, revealing fragments of pottery, bronze, and iron artifacts, as well as two burials (1-2). In the same year, a larger excavation, covering an area of 95.5 m2, was conducted by N.V. Vodolago, where seven more burials were found (3-9). In 1987, 10 burials (10-19) were discovered on an area of 157.5 m2. In 1988, E.E. Kirzhner continued the investigations, excavating an area of 125 m2 and studying three burials (20-22). As a result, over the course of three years, an area of 387 m2 was investigated, revealing a total of 22 burials.

### *burial 13 (individual ID I19111, Female)*

A 14-16 years old female was identified in burial 13. Along the presumed southern longitudinal wall of the burial, at the boundary of the mainland, at a depth of 40-56 cm, the remains of a fragmented skeleton were found in disorder. The bones of the pelvis and legs were relatively undisturbed. Based on their position, it appears that the deceased was laid with her head to the northwest. No inventory was found in association with this burial.

### *burial 22 (individual ID I19112, Male)*

A male, aged 40-50 years old, was identified in burial 22. The burial was documented at a depth of 100 cm. The grave pit had a rectangular shape, with dimensions of 270x87x118 cm, oriented along the northwest-southeast axis. At the bottom, remnants of a wooden coffin measuring 171x38 cm were found. Upon clearance, a moderately preserved skeleton was uncovered, including cranial bones, partial rib cage, arm bones without hands, and leg bones. Based on the arrangement, it appears that the deceased was laid with his head to the northwest, lying on his back, with the skull facing upwards, arms alongside the body, and legs extended. Vertebrae and likely parts of the scapulae were found at the head. A gold pendant with an amber insert was discovered on the cranial bones, while iron fittings, fragments of clothing, belt buckle, and bronze fittings and tweezers were found around the waist and pelvis area. A bronze collar and scabbard tip, as well as iron knives were found near the right femur. An iron saber was placed near the left foot, an iron axe near the right knee, and an iron buckle near the left knee. Iron spurs and stirrups were detected near the right foot.

## II.F. The Early Medieval Novinki-type sites (MidVolga_Novinki)

See Szeifert et al., 2022 for the general description of the Novinki-type sites.

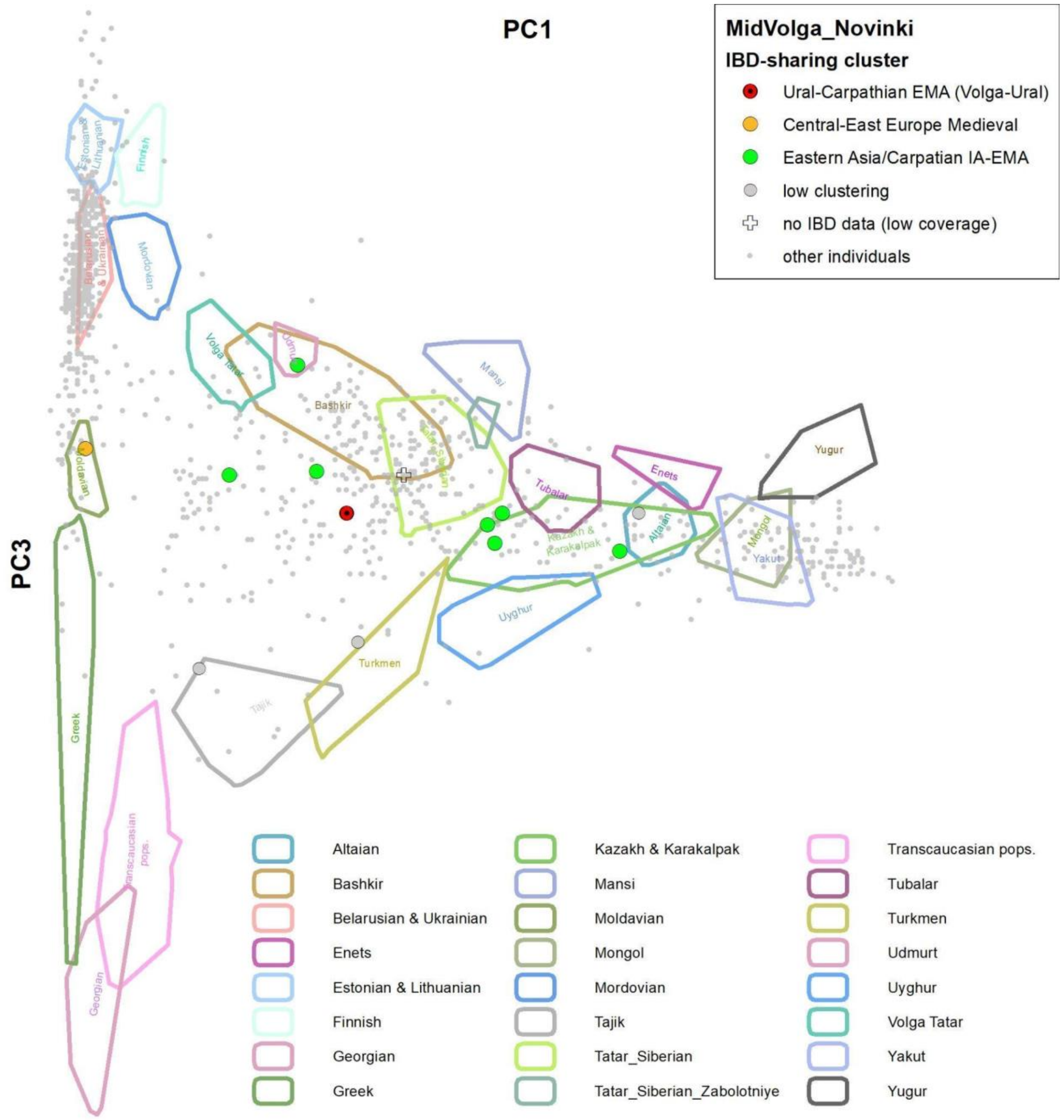


***Figure SII.12.*** *Eurasian-scale principal component analysis (PCA), with a projection of the newly sequenced individuals onto modern genetic variation following Jeong et al. 2019. The PC1 and PC3 dimensions are shown, including the newly presented genomes. Polygons represent example populations from modern Eurasian individuals onto which the ancient samples were projected, while grey dots indicate ancient reference samples from our IBD-sharing network.*

**Genetic results.** *In this group, we analyzed thirteen individuals from the sites of Novinki-1 (n=5), Brusyany-2 (n=4), Brusyany-'unidentified' (n=3), and Malaya-Ryazan' (n=1). All of them had sufficient coverage for downstream population genetic modeling (>100,000 SNPs), and twelve were suitable for IBD-sharing analysis (>600,000 SNPs). The individuals I32663 and I32375 from the Brusyany site are first-degree relatives. In this group the female-to-male ratio was 5:8. The samples displayed diverse genetic compositions, suggesting possible continuity from the local Low Kama Iron Age groups alongside heterogeneous gene flows from both Western and Eastern Eurasia. The supervised ADMIXTURE analysis revealed elevated levels*

*of Samara_EBA_Yamnaya and Baikal_N ancestries across the group. The MidVolga_Novinki samples clustered mostly within the Eastern Asia/Carpathian IA-EMA and Urals-Carpathian EMA clusters, although some individuals did not cluster within any one in the Central-East Europe Medieval cluster. This group's genetic diversity indicates the presence of individuals with ancestry from the local, broader region, Eastern Europe, the Caucasus/Pontic-Steppe, and the Eastern Eurasian steppe. These findings suggest that the group was composed predominantly of newcomers to the region, with a few exceptions reflecting local continuity.*

## II.F.1. Kurgan groups at Brusyany and Malaya Ryazan’

See Szeifert et al., 2022 for the general description of the kurgan groups at Brusyany.

### *II.F.1.1. Brusyany-2 kurgan group*

**mound 22, burial 3 (individual ID I19085, Female)**

At the southern border of the kurgan mound, in the layer of buried soil, poorly preserved bones of a child were found, placed on its back with the head to the west. To the left of the skull, there was a rounded-bottom pot vessel.

**mound 23, burial 3 (individual ID I32663, Male)**

The burial was unearthed in the northeast sector of the mound, outside the rubblework. The skeleton of a female person lay stretched out on its back, with the head to the northwest in the center of the rectangular burial with rounded corners. The bones of the ankles were strongly pressed together (bound?); the skull was turned to the right side. Near the wrist of the right hand, there was a spindle; near the skull and the right shoulder - bronze pendant balls with loops and bronze open rings-earrings.

**mound 23, burial 5 (individual ID I19086, Male)**

Fragments of the infant's skull were found in the northwest sector outside the stone rubblework. The determined radiocarbon age of the burial is 600-666 cal CE (1400±30 BP, Poz-136205) (based on human bone).

Three more individuals from Brusyany lack archaeological description because of mislabeling of their grave numbers. Their individual IDs are I25530 (Male), I32375 (Female), and I32376 (Female).

### *II.F.1.2. Malaya Ryazan' burial site*

See Szeifert et al., 2022 for the general description of the sites at Malaya Ryazan’.

**burial 4 (individual ID I19082, Female)**

The determined radiocarbon age of the burial is 576-654 cal CE (1440±30 BP, Poz-136234) (based on human bone).

## II.F.2. Novinki-1 kurgan group

See Szeifert et al., 2022 for the general description of the sites at Novinki.

### *mound 7, burial 2 (individual ID I16639, Male)*

A burial was performed in a rectangular pit measuring 260 cm in length and 70-80 cm in width. The burial has been disturbed, with the skull broken into two parts. Presumably male, the age of the deceased is estimated to be around 40 years. Anthropological analysis indicates that the bones have not been preserved. Fragments of iron objects (plates) and two bronze overlays were found in the burial.

### *mound 8, burial 6 (individual ID I19104, Male)*

The burial occupies a central position within the mound and is oriented in an east-west direction. It likely intersected another burial pit oriented to the south, or this pit may represent a cenotaph.

A small recess was made in the northern wall of the burial. The skeleton is fragmented, with the bones scattered. Only the lower leg bones remained in place, indicating that the burial was oriented with the head to the west. Two items were found in the burial: a piece of charcoal at the feet and an iron rod.

The determined radiocarbon age of the burial is 610-680 cal CE (1377±24 BP, DeA-16588) (based on human bone).

### *mound 9, burial 1 (individual ID I16640, Male)*

The determined radiocarbon age of the burial is 656-775 cal CE (1320±20 BP, PSUAMS-10312) (based on human bone).

### *mound 9, burial 6 (individual ID I19102, Male)*

The burial of a 5-6-year-old child was in a rectangular pit excavated 0.2 meters into the subsoil and oriented east-west. A sparse stone rubblework layer was observed in the vicinity of the burial. The burial, likely oriented to the west, is heavily damaged. The skull and part of the tubular bones are concentrated in the western part of the pit. The burial inventory includes a bent bronze earring of Saltiv type, fragments of an iron knife, and a multi-component iron object of unclear purpose.

The determined radiocarbon age of the burial is 576-654 cal CE (1440±30 BP, Poz-136244) (based on human bone).

***mound 12, burial 1 (individual ID I19103, Female)***

The outlines of the burial pit are not discernible. The buried individual, a child aged 6-7 years, was oriented with the head to the west, lying supine with arms extended along the torso. The spine is poorly preserved.

Two large buttons made of bronze and covered with gold foil were found under the mandibula. The buttons were filled with a white paste on the inner side, into which bronze loops were inserted for suspension. A glass bead with a bronze lining was found on the chest of the deceased. At the bottom, underneath the spine, there are several poorly preserved glass beads. No other items were present.

The determined radiocarbon age of the burial is 433-592 cal CE (1545±30 BP, Poz-136245) (based on human bone).

## II.G. The Early Volga Bulgaria burials (MidVolga_EVB)

The collapse of the Imen'kovo culture in the second half of the 7th century CE led to depopulation in the Middle Volga region, with settlements appearing only on the periphery. By the late 7th century, recolonization began, continuing until the second quarter of the 10th century, marking the Early Bulgarian period. This era coincided with the Khazar period in Eastern Europe and unfolded in stages.

The first stage (c. 690–750 CE) saw the arrival of Turkic nomads (likely the Khazars and Bulgars) whose burial mounds appeared along the Volga. Small sedentary groups also emerged, as seen at the Zhiguli complex in Samara Luka. The second stage (c. 750–850 CE) followed the Arab defeat of the Khazar Khaganate in 737 CE, prompting migration from the Saltiv culture area to the Middle Volga.

The third stage (850–910 CE) coincided with the Khazar conversion to Judaism, further intensifying the cultural mix in the region.

Tankeevka stands out as one of the largest burial grounds, with over 5000 graves dating from the late 8th to the early 10th century, reflecting the growing diversity and population in the region. This period marked increased migration from the Don basin, introducing Saltiv cultural traditions, while other groups arrived from the Middle Kama.

Despite increasing settlement, peripheral areas like the Upper Sura remained sparsely populated, while the eastern Middle Volga saw limited habitation until the 10th century. Tankeevka's prominence underscores the central role of burial grounds in tracing the demographic and cultural changes that reshaped the Middle Volga during this transformative period (for the further reading, see references in Vyazov et al., 2019).

**Genetic results.** *In this group, we analyzed eight individuals from the sites of Tankeyevka (n=7) and Mullovka (Cheremshan) (n=1). All eight samples had sufficient coverage for downstream population genetic modeling (>100,000 SNPs), and six were suitable for IBD-sharing analysis (>600,000 SNPs). No close relatedness was detected among these individuals, and the female-to-male ratio was 3:5.*

*The samples displayed a genetic composition similar to the local Low Kama Iron Age groups but with a detectable level of Western Eurasian gene flow across the group. The supervised ADMIXTURE analysis revealed elevated levels of Samara_EBA_Yamnaya and Yakutia_LNBA/Turkey_N ancestries. The MidVolga_EVB samples are clustered in IBD within the Urals-Carpathian EMA cluster, however, its ancestry was distinct from the Early Medieval Magyars and Karayakupovo horizon groups. Differences in genetic composition suggest a separate genetic history for this group.*

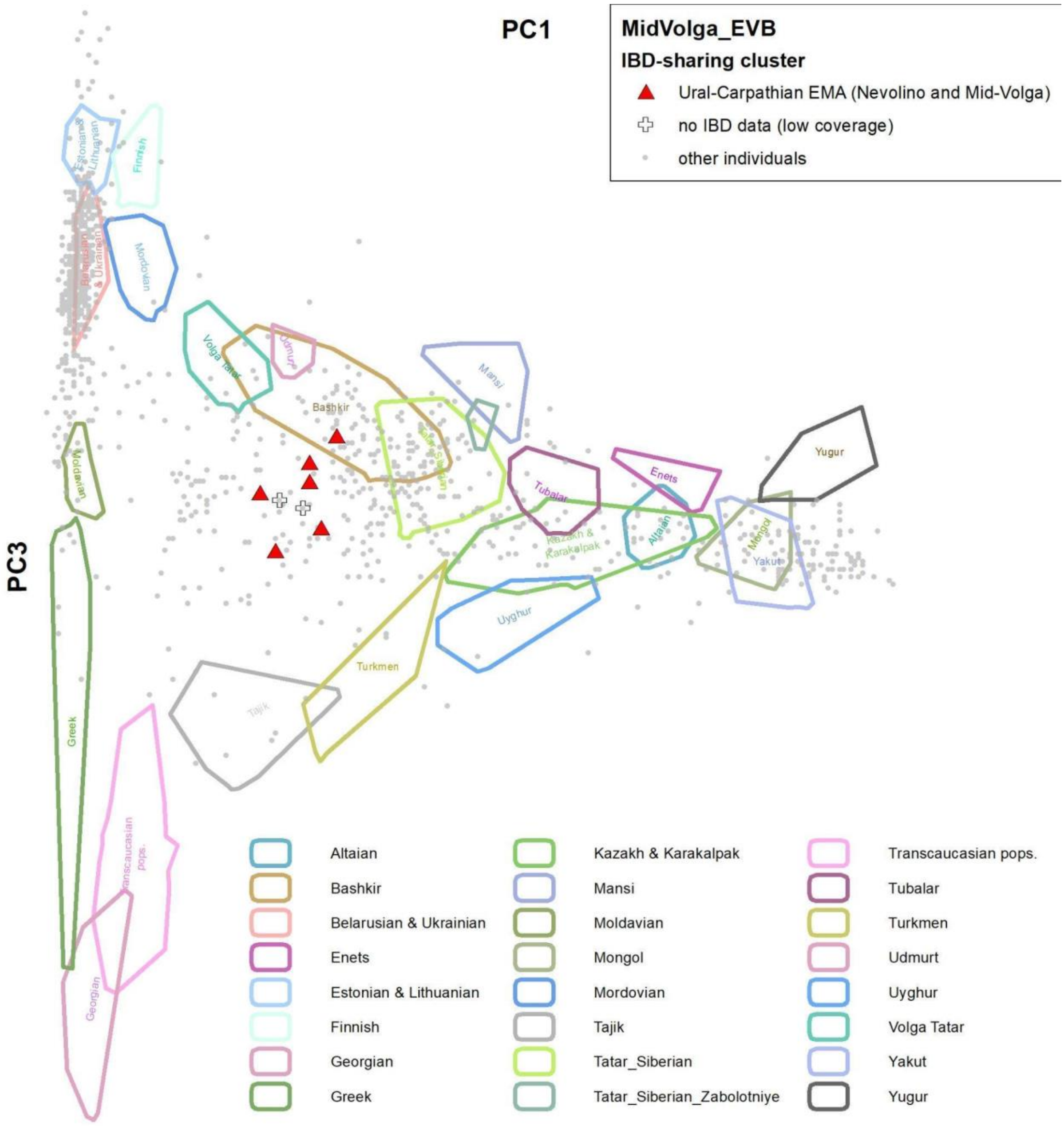


***Figure SII.13.*** *Eurasian-scale principal component analysis (PCA), with a projection of the newly sequenced individuals onto modern genetic variation following Jeong et al. 2019. The PC1 and PC3 dimensions are shown, including the newly presented genomes. Polygons represent example populations from modern Eurasian individuals onto which the ancient samples were projected, while grey dots indicate ancient reference samples from our IBD-sharing network.*

## II.G.1. Tankeyevka cemetery

See Szeifert et al., 2022 and Khalikova and Kazakov, 1977 for the general description of the Tankeyevka burial ground.

### *burial 657 (individual ID I25529, Male)*

The grave was excavated by E.A. Khalikova and E.P. Kazakov in 1966. The burial pit of 50 cm width was partially damaged by later earthworks. Only the teeth of the deceased were preserved in the grave. Based on their placement within the burial pit, the individual was oriented with their head to the northeast. Grave goods included a round-bottomed, handmade clay vessel containing an admixture of mollusk shell. Within the vessel, a bone spoon was discovered. Beneath the teeth, a bronze Saltiv-type earring was recovered.

### *burial 961 (individual ID I25532, Female)*

The burial was excavated by E,P. Kazakov in 1974. The outline of the grave pit, measuring 220x50 cm, appeared at a depth of 100 cm. The burial was made at a depth of 150 cm.

From the deceased in situ, the bones of the legs were preserved, indicating that the buried individual was oriented with their head to the southwest and lay extended on their back. The remaining bones of the skeleton were scattered in disarray in the central and western parts of the pit. At the western end of the pit, a fragment of a shield was uncovered, a bronze mirror with a wooden handle, and a bronze wheel-shaped pendant. In the center of the grave, a polychrome bead was found. During the collection of bones, a lead overlay, a bronze fragment, and a brass ring were uncovered in the northwestern corner of the pit.

The determined radiocarbon age of the burial is 710-957 cal CE (1185±30 BP, Poz-136155) (based on human bone).

### *burial 1006 (individual ID I25533, Female)*

The burial was excavated by E.P. Kazakov in 1975. The grave pit measured 200 × 45 cm. Approximately 60 cm above the level where the grave pit boundaries were identified, an animal bone was unearthed. The burial itself was located at a depth of 135 cm. The grave had been robbed, leaving only the long bones of the legs in situ. Based on their position, the skeleton had been placed in a supine position and oriented with the head to the west-southwest. In the western part of the grave, human teeth were found alongside an animal bone and two glass beads containing silver foil. In the central part of the grave, remnants of decayed wood, likely belonging to a coffin, were discovered.

### *burial 1012 (individual ID I25534, Male)*

The burial was excavated by E.P. Kazakov in 1975. The burial pit measured 260 to 120 cm and was oriented from west to east. Its borders were recorded at a depth of 130 cm, and in the upper part the grave pit was disturbed by a robbery dig and a later cash pit. At a depth of

170 cm from the surface, two skeletons were found in a grave supplemented by rich grave goods.

The "southern" skeleton was represented by a partially destroyed skull found in the western part of the pit. In the eastern part of it, a collapsed single-handled jar and an iron buckle were found. A horse's skull was located here, adjacent to the lower wall. To the north of the skull, the bones of two horse legs with their hooves to the west were discovered. A poorly preserved bridle was found on them. The bones of the other two horse legs were placed to the east of the skull and laid perpendicular to the grave pit axis.

The "northern" skeleton was represented by a broken skull and some tubular bones remained in the infill of the eastern part of the pit. The tubular bones and lower part of the human skeleton were scattered in the eastern half of the pit. Under these bones, fragments of a handmade single-handled ceramic jar were found.

The inventory items include iron arrowheads and bronze plaques of four types. In the western half fragments of a silver decorative face mask, silk fabric, and fur clothing were found. In the eastern part, beneath tubular bones and sections of legs, fragments of iron knife and unidentifiable iron artifacts were uncovered. Finally, in the northern part, a poorly preserved horse skull was found lying on its right side, facing west, along with bones from parts of its legs and western limbs.

For the last three burials from Tankeevka (burial 1065 (individual ID I25526, Male), burial 1139 (individual ID I25527, Male), and burial 1143 (individual ID I25528, Male)), we lack archaeological descriptions. The individual ID I25527 from burial 1139 was dated to 892-1023 cal CE (1080±30 BP, Poz-136157) (based on human bone).

## II.H. The sites of the Karayakupovo Horizon in the Lower Kama area (LowKama_KH)

See section III.C for the general description of the Karayakupovo Horizon in the Volga-Ural region.

**Genetic results.** *In this group, we analyzed seven individuals from the site of Bolshie-Tigany (n=7). All had sufficient coverage for downstream population genetic modeling (>100,000 SNPs) and IBD-sharing analysis (>600,000 SNPs). The individuals I19105 and I19108; I19106 and I19107 are second-degree relatives and the female-to-male ratio in this site was 1:7. The samples exhibited admixture of local Iron Age genetic elements and Trans-Uralian Late Iron Age groups, as indicated by qpAdm analyses (Figure 4 in the Main text). The supervised ADMIXTURE analysis revealed elevated levels of Samara_EBA_Yamnaya and Yakutia_LNBA ancestries in this group. The LowKama_KH samples are clustered within the Urals-Carpathian EMA cluster. Genetic results showed a composition similar to other Karayakupovo Horizon groups, but with a detectable local Low Kama Iron Age component, suggesting recent admixture between these two groups in the Low-Kama region.*

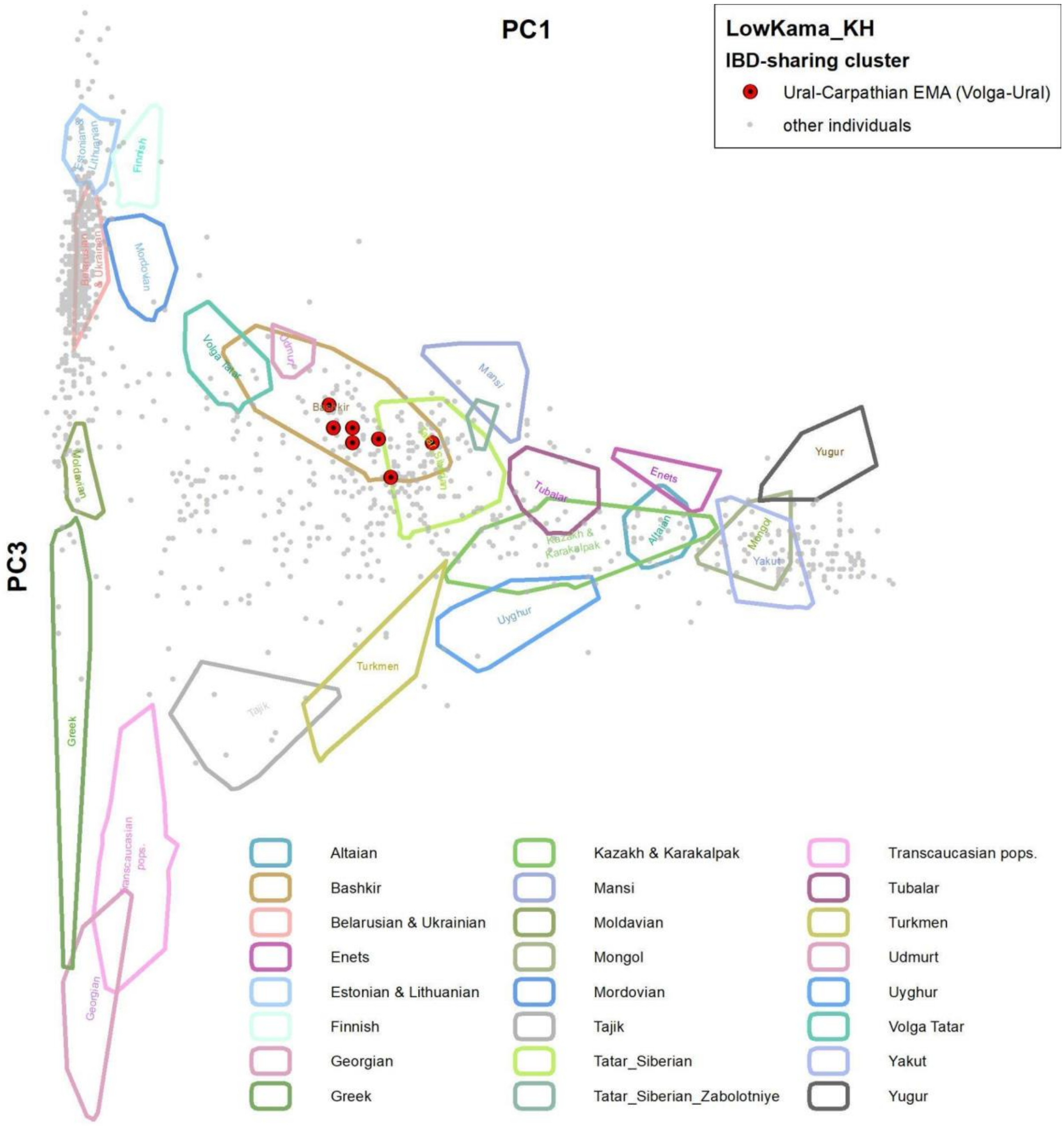


***Figure SII.14.*** *Eurasian-scale principal component analysis (PCA), with a projection of the newly sequenced individuals onto modern genetic variation following Jeong et al. 2019. The PC1 and PC3 dimensions are shown, including the newly presented genomes. Polygons represent example populations from modern Eurasian individuals onto which the ancient samples were projected, while grey dots indicate ancient reference samples from our IBD-sharing network.*

## II.H.1. Bolshiye Tigany burial ground

See Szeifert et al. (2022) and Khalikova & Khalikov (2018) for the general description of the Bolshiye Tigany site.

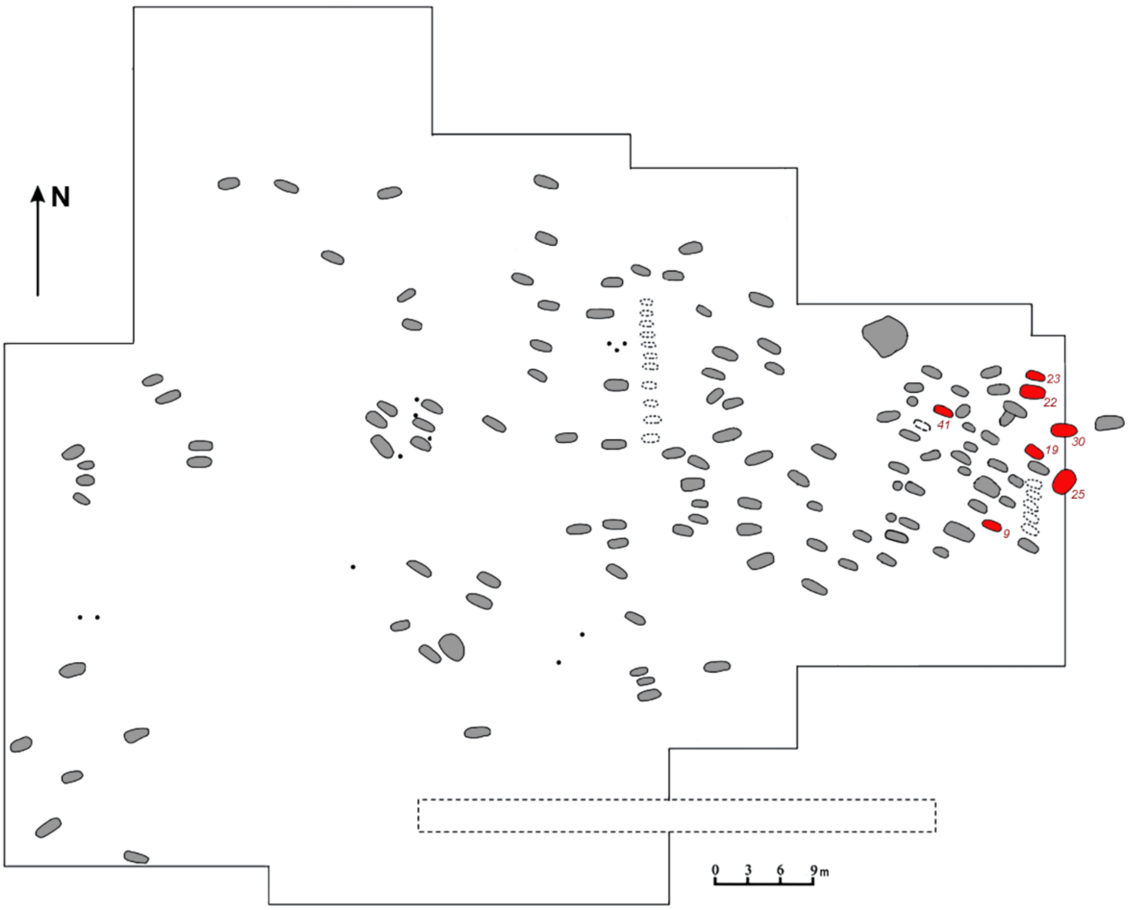


***Figure SII.15.*** *A plan of excavated burials at Bolshiye Tigany.*

## *burial 9 (individual ID I25489, Male)*

Grave pit (230×85 cm) was oriented along the west-south-west - east-north-east line. At the bottom, the poorly preserved skeleton of a male aged 30-40 years was found lying stretched out on his back, with his head to the west-south-west, faced upwards.

To the right of the skull, a horse femur (number 15 on the figure) and sheep tail vertebrae (16) were found. To the right of the mandibula of the deceased there was a small vessel (14) and a silver Saltiv-type earring (1). On the left side of the jaw, a leaf-shaped pendant (2) and a bronze bead (3) were discovered. On the elbow bones of the right arm, a bronze bracelet made of flattened wire (8) was found, and on the phalanx of the right middle finger, a silver ring with a pink stone in a bezel setting (9). Nearby, an iron knife (13) and an awl (12) were located. On the belt, remnants of a leather strap with two end straps decorated with silver fittings (10) were found. To the right of the belt, there was presumably a leather purse, from which a part of the round bronze lid with a punch ornament remained (11).

To the left of the skeleton, an iron saber, presumably placed in a wooden scabbard (6, 7), was found. The total length of the saber is 76 cm, with a slightly curved blade length of 67 cm. The sharp end of the blade, 11 cm long, is double-edged, while the rest is single-edged. The width of the blade is 3.0 cm. The crossguard has expansions at the ends. The handle is positioned at an angle towards the blade relative to the crossguard. The scabbard had a width of up to

4.5 cm. At a distance of 38 cm from the end, along the edge of the scabbard, there was an iron loop with a wooden attachment (7), and another one at the crossguard.

At the feet on the right side, the skull of a foal was uncovered. The bottom of the grave was lined with bast, and traces of fur and wooden coverings were preserved on the metal objects (Khalikova & Khalikov 2018).

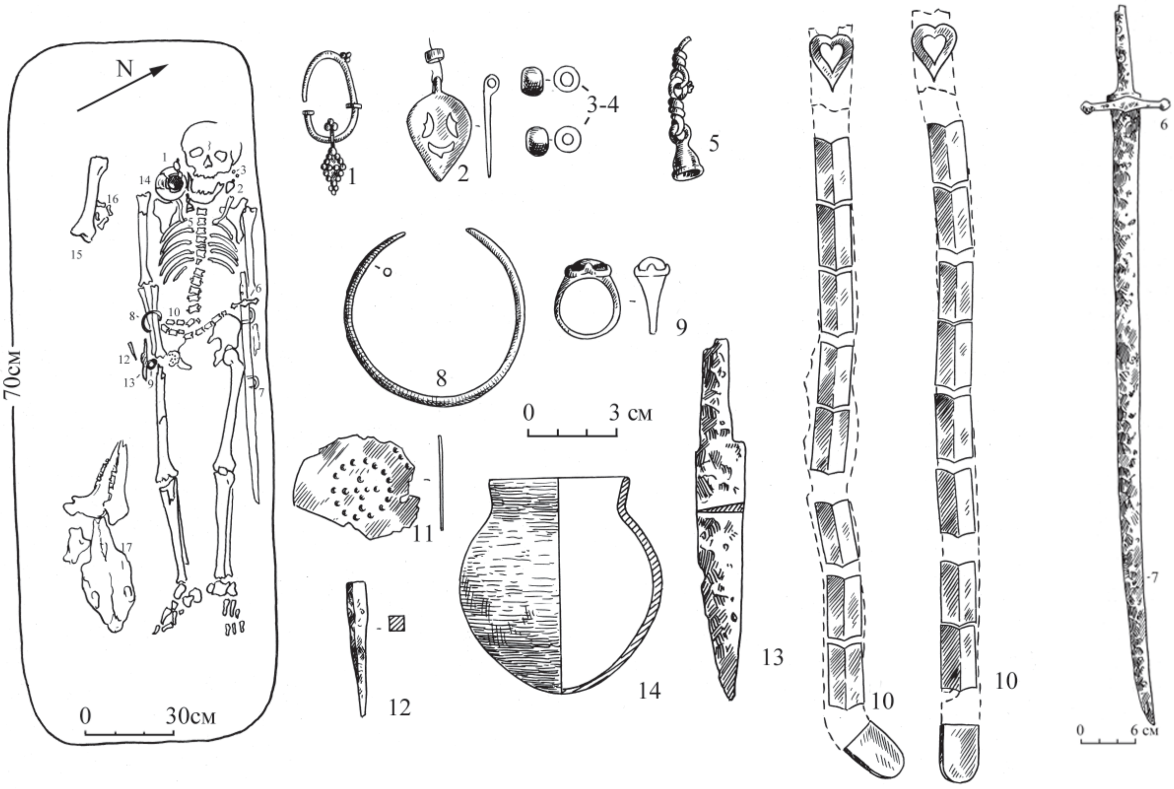


***Figure SII.16.*** *Burial 9 at Bolshiye Tigany.*

### *burial 19 (individual ID I19105, Female)*

The grave pit was initially traced at a depth of 70 cm as a sub-rectangular dark spot (80-100×215 cm) against subsoil loam. This spot was oriented from east to west and contained charred wood inclusions along the northern edge. At a depth of 77-80 cm, the pit narrowed on the western side, forming a ledge 25 cm wide. At the bottom of this ledge, a horse femur and small bones (36) were uncovered. At a depth of 80-90 cm, the main pit revealed: in the northern half, an “equine complex” consisting of a skull (35) and separately placed lower limbs (34) (horse was aged 5-6 years); in the southern half, a moderately preserved skeleton of a young female aged 16-17 years, lying on her back, head to the west, face to the south, with arms extended along the body. The deceased was apparently placed on a bedding of wood, felt, and fur. Carbonaceous inclusions were noted in the southeastern part of the grave (37).

The deceased was interred in relatively rich clothing, from which the following items were preserved: remnants of a headdress on the skull, with a forehead band adorned with leather and copper fittings (1-37 pieces), and a strap with silver buckles (3) and a tip (2) on the back of the head and crown; bronze earrings with metal beads ( 5, 6) and a simple gold ring (7) at

the temples; remains of two necklaces on the neck – one with beads (white and blue glass – 37 pieces, 11), yellow beads (150 pieces, 11), multi-linked glass beads with silver lining (9 pieces, 10), bi- and tri-linked glass beads (8 pieces, 9), and five leaf-shaped bronze pendants (4 pierced - 13; 1 solid cast - 14), spiral-wound bronze beads (18), and three leaf-shaped pendants (15, 16, 17); nine cowrie shells on the left side of the chest (12), possibly adorning the collar or edge of the clothing; the back of the shirt collar was adorned with silver leaf-shaped appliques and pendants (22, 23); the clothing was belted with a leather strap with a silver buckle (26), two pierced fittings (27-28), and an end strap with a silver tip (29). The good preservation of the details allows for a proposed reconstruction of the woman's clothing based on the materials from this burial. Accompanying the deceased were: silver oxide powder from an applique in the left eye socket (4); a bronze bracelet with notches and bumps on the wire and bent ends (19) on the right hand; a pair of bronze rings with bezels on the fingers; two spherical bone beads (25) and an iron buckle (24) on the left hand; an iron knife pointing towards the feet (32) and a bronze sub-triangular ring (31) between the shins; fragments of an iron awl (30) and a round-bottomed cylindrical-necked vessel (33) near the left temple and back of the head (Khalikova & Khalikov 2018).

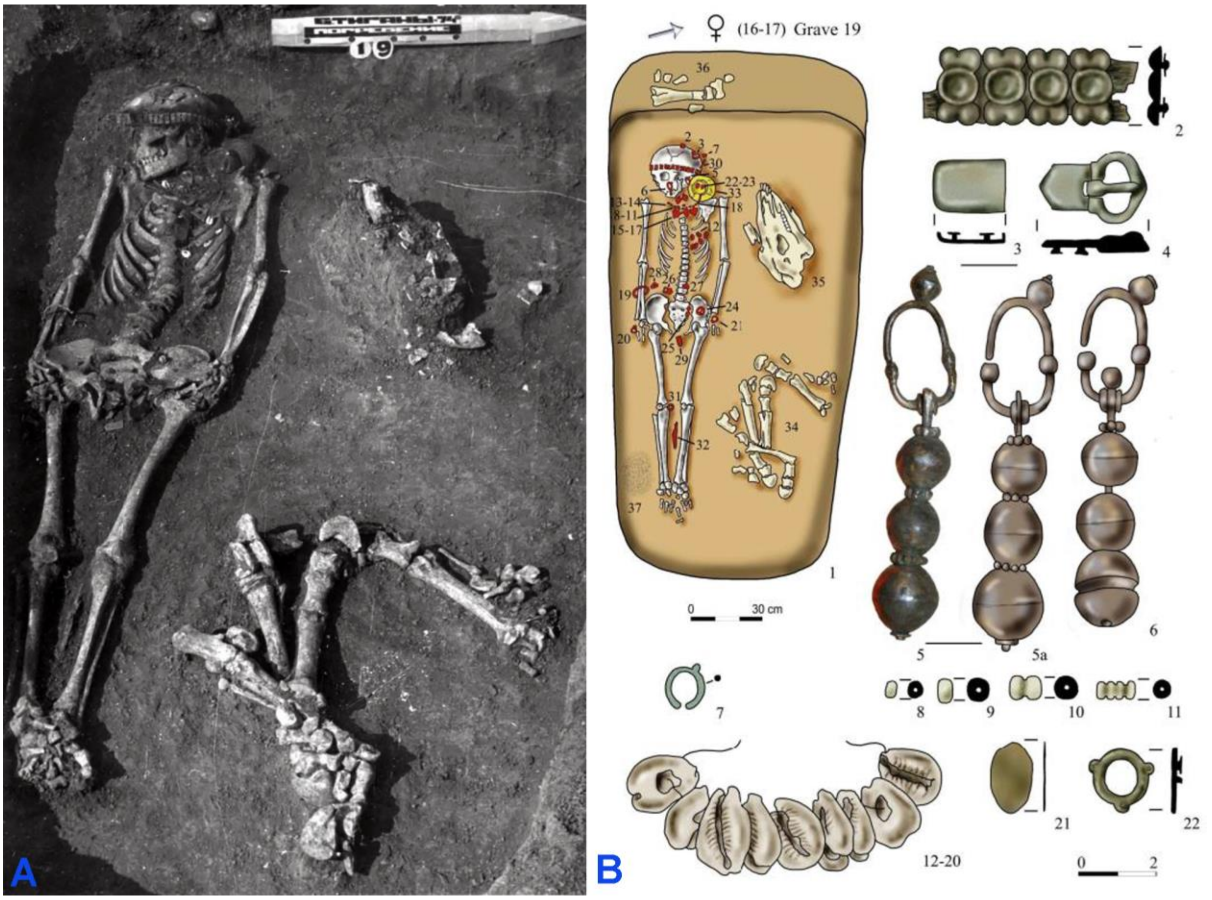


***Figure SII.17.*** *Burial 19 at Bolshiye Tigany. A - photo, B - plan and grave goods.*

### ***burial 22 (individual ID I19106, Male)***

The grave pit was initially traced at a depth of 70 cm as a large dark spot (280×175 cm) oriented east to west. At a depth of 97-100 cm, the pit narrowed sharply towards the southwestern corner and continued as an oval spot (90×240 cm) down to a depth of 115 cm, leaving a large empty space in the rest of the pit (figure 26). The overall fill contained carbonaceous inclusions and a fragment of a handmade vessel (13). At the bottom of the main pit, at a depth of 105-115 cm, the moderately preserved skeleton of a male of 50-60 years old was uncovered, lying on his back, head to the west. Accompanying goods were: a horse femur at the head (1); a round-bottomed, thin-walled vessel with elegant carved Kushnarenkovo-type ornamentation under the right side of the skull (2); a saber in scabbard decorated with silver fittings along the left side (6); a bridle made of leather straps decorated with bronze fittings and connected by triplets was presumably thrown over the right knee and thigh (8) – its good preservation allows for a proposed reconstruction; two bone fittings from a bow were presumably placed on the left thigh (7), and a quiver with four iron socketed arrowheads of different types was placed on the right thigh (9). From the clothing and personal adornments of the deceased, the following items were preserved: a bronze Saltovo-type earring found near the right temple (14); remnants of a necklace with a large coin-like silver pendant (3) and a bronze bead (15); a bronze bracelet made of a sub-rectangular wire with bent ends on the right hand (10); a belt set consisting of six round silver fittings, six semicircular fittings with a ring, and three semicircular slotted fittings ( 4), and a bronze tip (5); an iron knife in the remnants of a leather sheath (11), and a pouch with a fire striker, from which a flint and rust from the steel were preserved, were presumably suspended on the right side of the belt (17).

Traces of fur and wooden bedding were observed under the metal items (Khalikova & Khalikov 2018).

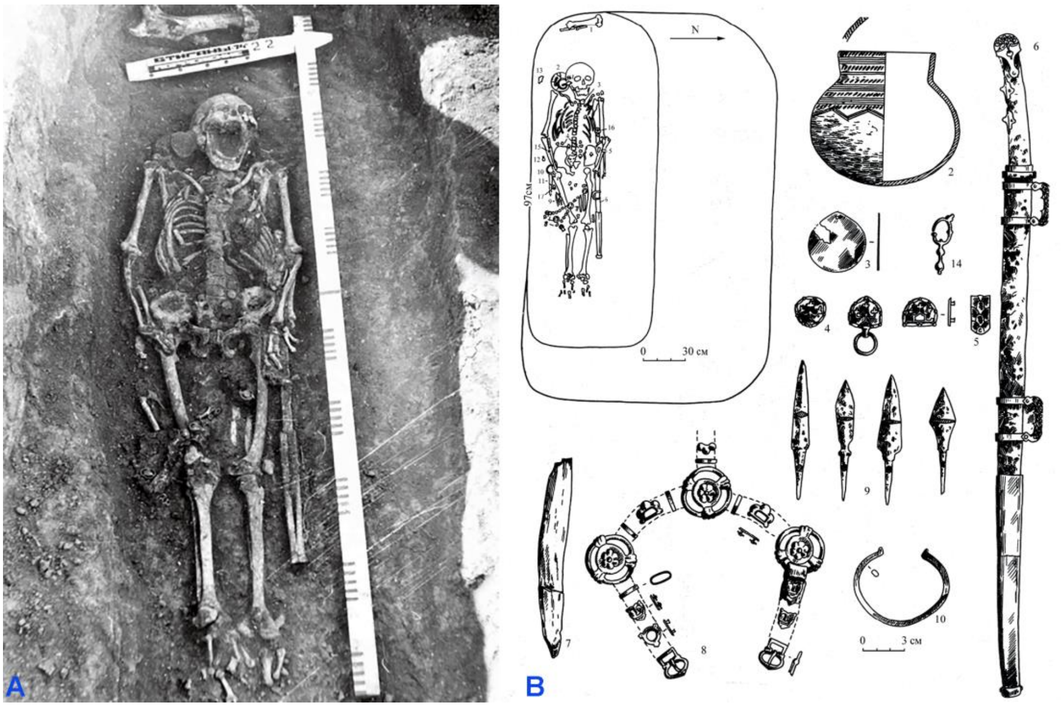


***Figure SII.18.*** *Burial 22 at Bolshiye Tigany. A - photo, B - plan and grave goods.*

### *burial 23 (individual ID I19107, Male)*

The grave pit was initially traced at a depth of 70 cm in the subsoil loam as a spot with rounded ends (65-80×200 cm) oriented east to west was outlined. At a depth of 90 cm, the pit revealed the moderately preserved remains of an elderly male (50-60 years old), laid on his back, head to the west, with the skull slightly turned to the right. The deceased was accompanied by grave goods. On the left side of the head, a cluster of horse bones (burial food?) (9) and a Kushnarenkovo-type clay vessel (11); on the right leg's shin bones, a horse skull (8); on the right forearm, remains of a bridle, from which several bronze fittings and a bit ring were preserved (2a-2c); along the right side of the upper torso, presumably a quiver, from which three iron socketed arrowheads (1) and an iron tip (10) were preserved. There was tarnish from silver eye covers on the skull's eye sockets (12). From personal adornments and clothing of the deceased, the following were preserved: a Saltiv-type earring near the right temple (13); around the neck area, three spherical bells possibly from clothing fasteners or a necklace (3); on the waist, remnants of a belt with a buckle (5), a semicircular fitting (4), and two sub-rectangular fittings (6), the surfaces of which were decorated with an original ornament depicting winged horses; on the index finger of the right hand, a ring with a stone (7); on the right side of the belt, an iron knife (15) and a fire striker, from which a flint and remains of a steel striker (14a-14c) were preserved (Khalikova & Khalikov 2018).

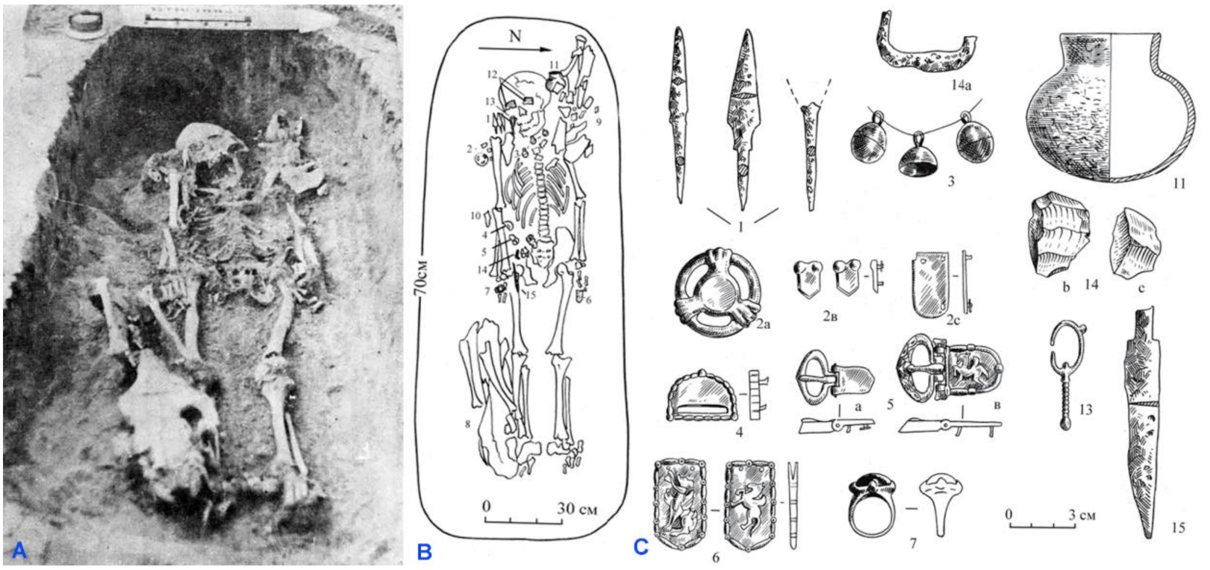


***Figure SII.19.*** *Burial 23 at Bolshiye Tigany. A - photo, B - plan, C - grave goods.*

### *burial 25, individual B (individual ID I19108, Male)*

The grave pit was initially traced at a depth of 65-70 cm as an oval-shaped spot (120×185 cm) oriented east to west.

Individual A. In the southern part of the spot, at a depth of 75 cm, the poorly preserved remains of an infant were uncovered, apparently laid on its back with the head to the west (only the decayed bones of the upper half of the torso were preserved). At the head, a large Kusnarenkovo-type clay vessel was found (1), along with beaver bone pendants (5). On the right side of the skull, there was a bronze Saltiv-type earring (2); around the neck area, remains

of a necklace made of small glass beads with silver inserts and paste beads (3); on the waist, remnants of a belt with copper hemispherical fittings (3 pieces) and a trefoil fitting (4).

Individual B (individual ID I19108). The northern part was deepened by 20 cm, and at a depth of 90 cm, the relatively better-preserved remains of a 6-7-year-old boy were uncovered, also laid on his back with the head to the west (the skull was crushed and turned to the left temple). The deceased was accompanied by: at the head and above the skull, a fragment of a large horse bone (shoulder blade?) (1); in front of the skull, a crushed clay vessel (2). From the clothing and personal adornments of the deceased, the following were preserved: on the right side of the skull, a bronze Saltovo-type earring (3); under the mandibula, remains of a necklace with a silver coin-like pendant (6) and glass beads with silver inserts (4); on the neck, a bronze button with a convex head (5); on the lower chest, a leaf-shaped openwork pendant (7) and a bronze spherical button (8); on the right wrist, several dark blue paste and glass beads with silver inserts (9). An iron knife was found on the left side of the pelvic bone (10) (Khalikova & Khalikov 2018).

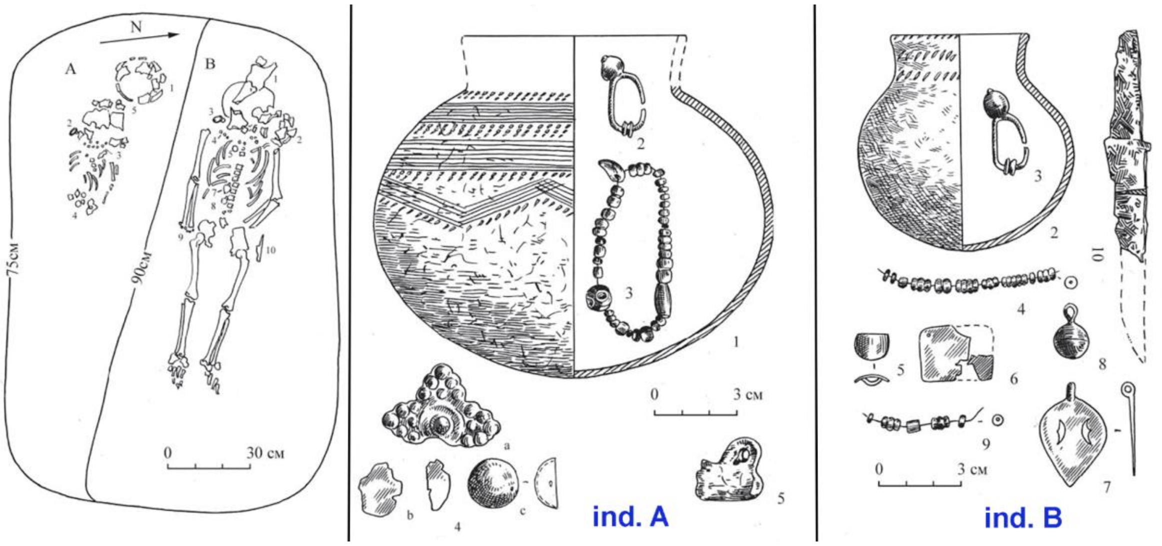


***Figure SII.20.*** *Burial 25 at Bolshiye Tigany, grave goods from individuals A and B.*

## *burial 30 (individual ID I25490, Male)*

The grave pit was initially traced at a depth of 80 cm, oriented fairly strictly from east to west. At the bottom of the grave pit, the remains of a female aged 35-45 were uncovered, laid on her back with her head to the west. A round-bottomed cylindrical-necked vessel was placed between the left side and the left elbow (9).

From the clothing and personal adornments of the deceased, the following were preserved: on the forehead and occipital parts of the skull, remnants of a headband made from appliqués attached to a leather strap with paired protrusions (1 - 27 pieces) and a bronze bead (16); under the right part of the occiput, a part of a bone comb with fine carved ornamentation was found (18); on both sides of the jaw lay massive earrings made of poor-quality silver with pin-shaped pendants (2); on the left eye socket, a silver eye guard fragment remained (4); under the jaw, in the upper part of the chest, remnants of a necklace made of beads and three bronze leaf-shaped openwork pendants (3, 12); in the upper part of the chest, an iron plate (fastener?;

5) and two small bronze spirals (7); below them, fragments of a bronze stick (6) and two bronze cast buttons (8); on the radial bones of the left hand, a bronze octagonal bracelet at the ends (10); on the middle finger of the right hand, a poor-quality silver ring with a dark stone in a bezel setting (11); in the upper part of the pelvis, remnants of metal belt details were preserved - a bronze buckle and bronze appliqués - four-part and heart-shaped openwork (12), a silver tip with an oval end (14) and two bell-shaped pendants (13); to the right of the belt, an iron knife was suspended, sheathed in a leather scabbard with a silver overlay (17), with a bronze bracket at the top of the scabbard (15).

Under the skeleton, especially in places where metal items were located, traces of bedding made from wood, felt, and fur were found (Khalikova & Khalikov 2018).

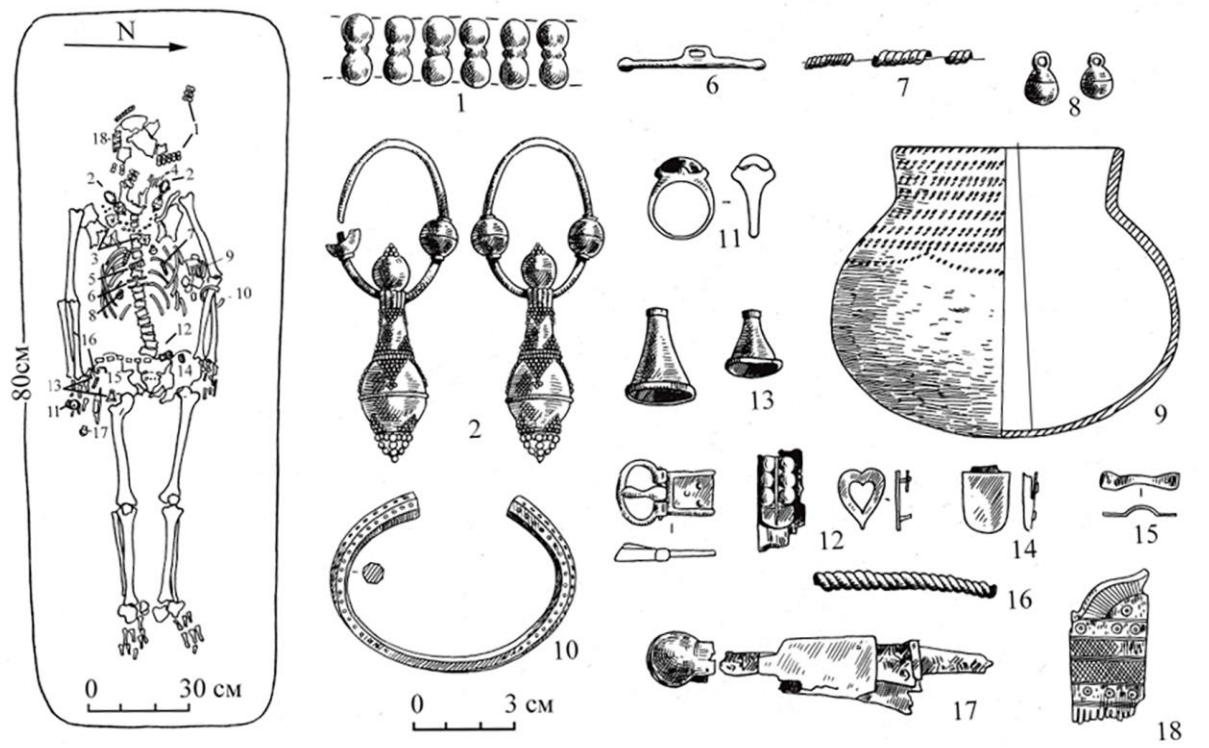


***Figure SII.21.*** *Burial 30 at Bolshiye Tigany. A - photo, B - plan and grave goods.*

### ***burial 41 (individual ID I19109, Male)***

The grave pit had dimensions 230×85 cm. At a depth of 65 cm, the poorly preserved remains of a male individual (aged 45-55 years) were unearthed, positioned supine with the head extended towards the west. The skull is turned to the left temple, with the left leg bones showing signs of rodent damage.

Accompanying the interred were placed: along the left side of the torso - a quiver, from which a set of six iron tang arrowheads (8) and an iron loop (11), likely from the binding of the upper part of the quiver, were preserved; a bow (bone overlays preserved - 9) and possibly a bridle with two bronze bit rings (10); at the right ulna - an iron knife (5).

Fragments of a silver plate from an eyeshield (3) were discovered around the left orbit.

From the details of the clothing and personal adornments of the deceased, the following were preserved: on the right temple - a bronze ring-shaped earring (4); around the neck area - remnants of a necklace made of glass beads (1) and fragments of two copper rattles (2); on the wrist of the right hand - a bronze bracelet with flattened and curved ends (6); on the right side of the pelvis - a bronze buckle (7).

Metal objects preserved traces and fragments of wood on top, likely from the lid of the burial or the wooden covering of the grave.

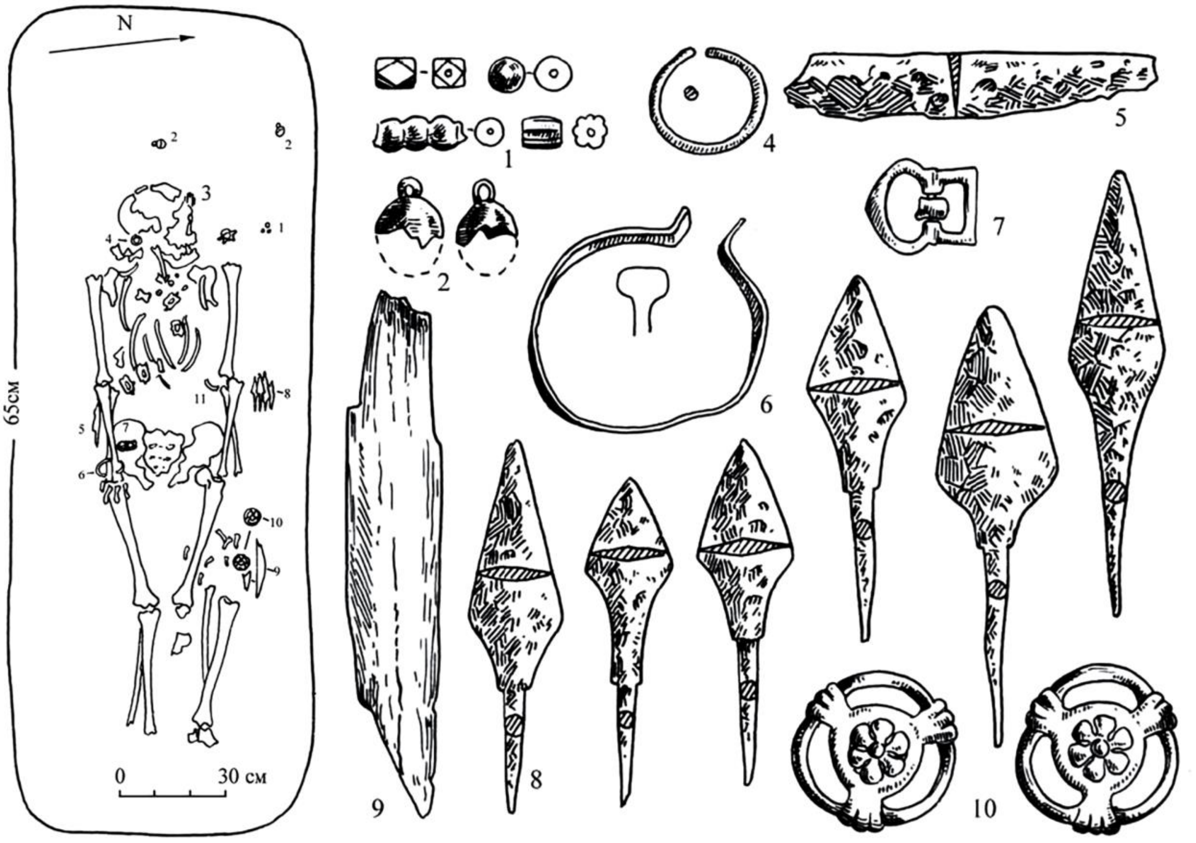


***Figure SII.22.*** *Burial 41 at Bolshiye Tigany.*

## II.I. The Medieval Chiyalik culture in the Lower Kama area (LowKama_Chiyalik)

See Szeifert et al., 2022 and Garustovich, 2015 for the general description of the Medieval Chiyalik culture.

**Genetic results.** *In this group, we analyzed eighteen individuals from the sites of Gulyukovo (n=7), Zuyevy-Klyuchi (n=10), and Ust-Menzelya (n=1). All 18 individuals had sufficient coverage for downstream population genetic modeling (>100,000 SNPs), and 17 were suitable for IBD-sharing analysis (>600,000 SNPs). No close relatedness was detected among these samples, and the female-to-male ratio was 11:7. The supervised ADMIXTURE analysis revealed elevated levels of Samara_EBA_Yamnaya and Yakutia_LNBA ancestries in this group. The LowKama_Chiyalik samples clustered within the Urals-Carpathian EMA, with only*

*a few individuals not clustering in any specific cluster. Both the genetic composition and IBD-sharing patterns suggest continuity from Early Medieval Karayakupovo individuals to the Late Medieval period in the Kama River region.*

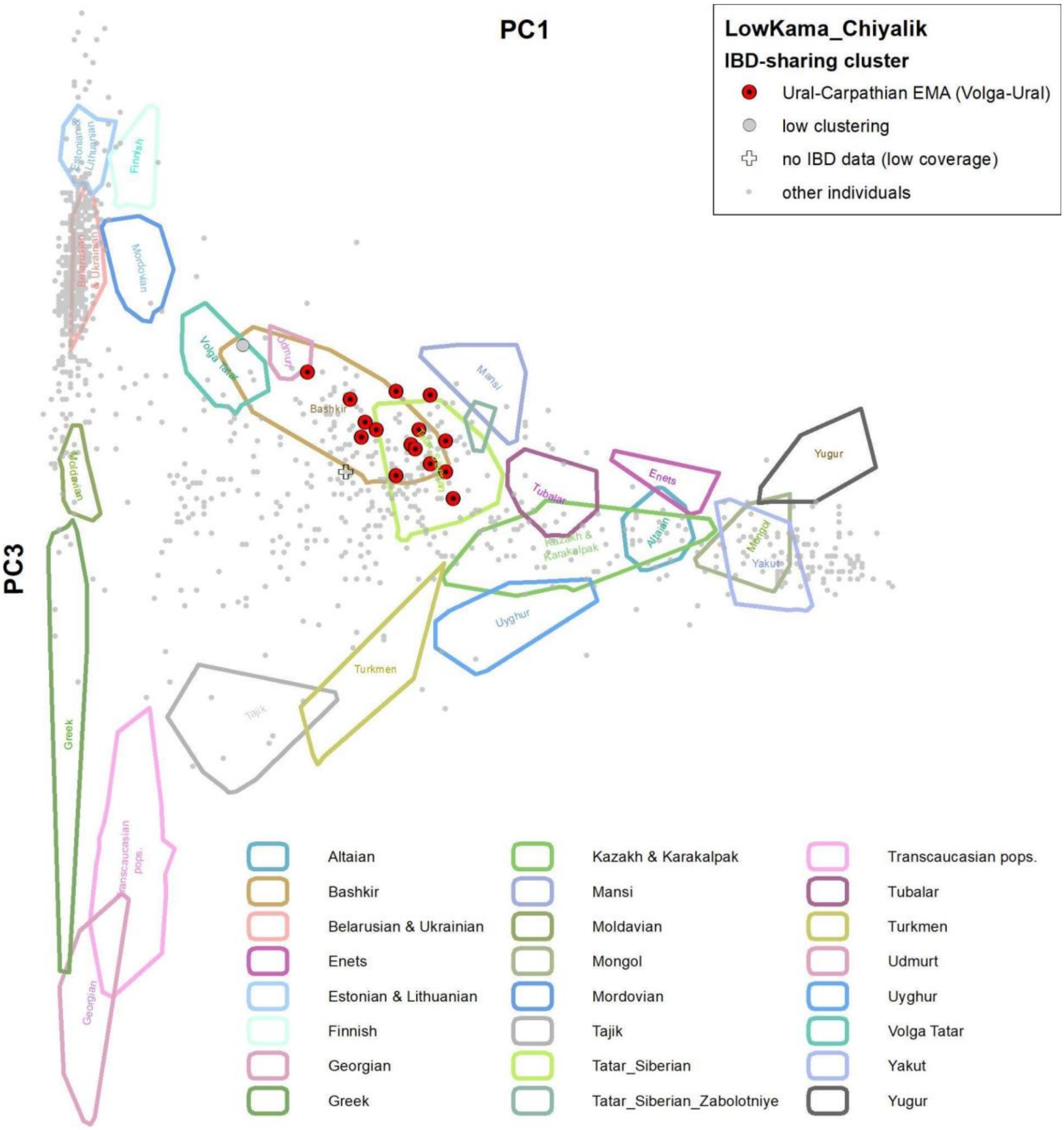


***Figure SII.23.*** *Eurasian-scale principal component analysis (PCA), with a projection of the newly sequenced individuals onto modern genetic variation following Jeong et al. 2019. The PC1 and PC3 dimensions are shown, including the newly presented genomes. Polygons represent example populations from modern Eurasian individuals onto which the ancient samples were projected, while grey dots indicate ancient reference samples from our IBD-sharing network.*

## II.I.1. Gulyukovo burial site

The archaeological site is located at the northern tip of the watershed between the Igat and Tirgaush rivers, left tributaries of the Ik River, where they flow into the Nizhnekamsk Reservoir

(formerly, before the reservoir's formation, where they entered the low floodplain of the Ik River). The relief of the watershed from the village of Gulyukovo to the reservoir consists of a very sloping ridge with elevations ranging from 85 to 70 meters above sea level. The surface features gentle slopes (10-12°) sloping towards both the reservoir and the valleys of the side streams. Towards the reservoir, the surface gradually levels off (down to 3°), and as it approaches the edge, within a strip 10-20 meters wide, it becomes almost horizonal (1-3°). However, in cross-section, the final segment retains a gently convex profile, visually well observed from the reservoir shoreline and recorded by height measurements of the erosion ledge: it varies from 5-6 meters in the area of excavations 4 and 5 in 2009 (at the center of the terraced slope) to 1 meter near the modern channels of the Igat and Tirgaush rivers (on the flanks) (Khisyametdinova, 2012).

The opposite banks of the Igat and Tirgaush rivers, facing the watershed, consist of the ridge tips extending to the Ik River with elevations of 100-150 meters and steep slopes complicated by gullies and ravines. As a result, the Igat-Tirgaush interfluve represents the most convenient (and perhaps the only sufficiently wide and low) passage into the Ik-Kama floodplain from the village of Kulushevo (Tukaevsky district) to the town of Menzelinsk.

The occupation site at Gulyukovo, attributed to the Neolithic and Late Bronze Age, has been known since 1979. The burials eroded by the abrasion of the Nizhnekamsk Reservoir were first documented in the Gulyukovo area by Andrey Chizhevsky in 1995-1996, who excavated 8 of them and called the site the Guluykovo Cemetery. From 1997 to 2001, 2003-2004, and 2007, N.M. Kaplenko, a teacher from Secondary School No. 8 in Naberezhnye Chelny, excavated approximately 538 m2, investigating 56 burials identified as "early Muslim" and attributed to the period from the 12th to the early 14th centuries CE (4 of them belong to the late Sarmatian part of the cemetery and are dated to the mid-second half of the 4th century CE). In 2006, Dmitry Bugrov and Ayrat Sitdikov excavated approximately 293 m2, studying 43 burials (7 identified as late Sarmatian, 36 as "early Muslim" from the second half of the 11th to the beginning (first third?) of the 13th centuries CE. In 2007 Dmitry Bugrov excavated another 103.5 m2 of the site area, revealing 15 more "early Muslim" burials. Further rescue excavations covering an area of approximately 225 m2 were conducted by Ruslan Matveyev in 2009, Anton Lyganov and Renat Valiev in 2012, Pavel Krasilnikov in 2014 under the supervision of Dmitry Bugrov. These excavations did not yield any medieval burials; instead, layers of the settlement, 6 Late Sarmatian burials, and a Medieval household were discovered. In total, at the moment approximately 1160 m2 area are unearthed, revealing 111 burials attributed to the Chiyalik culture (see Bugrov, Asylgaraeva, 2020 for detailed information and references).

The total area of the Medieval cemetery at Gulyukovo reaches 2 hectares. The burials were arranged in more or less dense groups within the excavation area, organized into 2-4 vague rows, with rows stretched parallel to the edge of the terrace (along the SSE-NNW and SSW-NNE lines), with gaps between groups ranging from 3-4 to 10-15 meters.

The burial rite of the site is characterized by individually arranged inhumations in simple graves (elongated rectangular in shape with rounded corners or ends), approximately corresponding to or slightly exceeding the body size. In 8 cases (burials 5, 7, 8, 19, 20, 39, 44, 47), a "leaning" of the northern long wall of the pits was observed (similar to the "lahd" feature).

The buried individuals were laid out in the supine positions stretched on their backs, with their heads mostly oriented towards the southwest and south-southwest (36 and 32 cases, respectively), followed by west (12 cases), south-southwest (11 cases), and one case each towards the south, northwest, and west-northwest. In 42 cases, a pronounced turning of the head (less frequently the torso) of the deceased was observed: in 30 burials to the right, facing south and southwest (towards Mecca), and in 12 cases to the left, facing south and south-southwest and facing north and north-north-west.

In three cases (burials 49, 51, 54), a specific disturbance of the anatomical order of the skeletons was observed: some bones, primarily limbs, retained their position, while the bones of the chest, spine, pelvis, and less frequently the skull and part of the limb bones, were mixed and lay in disorder, but approximately in their anatomical positions (possibly reflecting a "neutralizing the deceased" ritual).

Inventory items were found in 16 burials, including iron coffin nails in one burial (burial 31K). In other cases, the grave goods are represented by decorations, clothing accessories, and tools: burial 1K - bracelet, round pendant-button, beads; burial 2K - iron knife; burial 8K - bone plate, iron knife, and two poorly preserved arrowheads (?); burial 11K - pendant-amulet, bracelet; burials 15K and 19K - one belt (?) plate in each; burials 18K and 12 - one each of bronze temple rings; burial 21K - a pair of similar rings and a knife; burial 2K - bronze ring, glass bead; PB (burial in the bank exposure) 3K - bronze ring, glass bead, and a knife; burial 4 - metal plate, possibly a pin (?), burin (?); burial 25 - fragment of a bronze ornament with grain, copper plate; burial 38 - knife, glass bead, a broken object made of lead-tin alloy; burial 51 - plate-like pendant made of copper. Items from destroyed burials were also present in the excavation material: temple rings made of "white bronze" and gold, a bronze drop-shaped faceted pendant-button, a bone needle, and iron knives.

The reference assemblages for the chronological interpretation of the site were found in burials 1K, 11K, and 4 (Bugrov et al., 2010, fig. 2).

In burial 1K, the earliest chronologically indicating item is a glass bead in the form of a "lemon" type (Bugrov et al., 2010, fig. 2: 6). Similar beads made of yellow semi-transparent glass with protrusions around the holes are characteristic of the second half of the 10th to the early 11th century in Eastern Europe, including Volga Bulgaria, persisting until the end of the 11th century. Individual finds in layers from the 12th century are considered residual. Spherical beads made of rock crystal and bipyramidal beads made of carnelian from burial 1K have their peak distribution in the Volga-Kama region in the 11th-13th centuries.

Round pendant-buttons from burial 1K and drop-shaped faceted "buttons" from the excavation material were common from the late 10th to the 11th century and were found in Bulgarian sites and the periphery of Volga Bulgaria, including the Mari Volga region, Permian Cis-Urals, Trans-Urals, and Western Siberia.

Bracelets with expanded rounded ends from burials 1K and 11K are represented in sites dated from the 11th-12th centuries in the Southern Urals, Upper Kama region, and the Vyatka basin.

The pendant-amulet from burial 11K dates within the 11th-12th centuries.

The bronze plate with a wide plate loop for suspension from burial 4 is similar to silver plates from the Butaikha hoard, interpreted as decorations for horse trappings and dated from the 11th to the first half of the 13th century. In the context of the Chally Ensemble, such plates serve as female necklaces, and the complexes with them are dated to the turn of the 11th-12th centuries to the first third of the 13th century or the 12th-13th centuries. Such decorations are one of the culturally defining features in the women's costume of the Chiyalik culture. Similarly, as the central medallion of a necklace or chest pendant, bronze and silver plaques of various types were used by the population who left the burial grounds of Selyanino Ozero, Kishert, Kushulevo, Azmetyevo-1, Taktalachuk, Derbyoshki in the Cis-Urals (Pastushenko, 2005, p. 37-40), Pylayevo (Pastushenko, 2005, p. 39) and “Kozyr” (Garustovich, et al., 2008, p. 57) in the Trans-Urals. Some of them are extremely similar to the specimen from burial 4 in size and design (Pastushenko, 2006, Fig. 2: 2; 9: 6; 14: 15; Yutina, 2007, Fig. 7: 1, 8; Garustovich et al., 2008, Fig. 2: 23).

Thus, the overall dating of the site falls within the period from the second half of the 11th century to the beginning (possibly the first third?) of the 13th century. Narrower chronological phases of the site have not been delineated due to insufficient evidence. Presumably, the earlier part (associated with "inventory" burials) may have been obliterated by abrasion, while the later part (lacking inventory) does not have indicators for precise dating.

Specific elements of material culture left by the population buried at Gulyukovo include round bronze plaque with a plate loop, used as a neck-chest pendant (burial 4). Such ornaments are one of the culture-defining traits in the female costume of the Chyalyk culture. Similarly, bronze and silver plaques of various types were used by the population of other burial grounds in the Urals and Trans-Urals as central medallions of necklaces or chest pendants. Some of them closely resemble the specimen from burial 4 in size and construction.

Cultural influences on the population that left the Gulyukovo burial ground are traceable through characteristic elements identified in burial 31K. In this burial, the deceased was placed in a coffin secured with 11 iron nails, which is typical for Muslim cemeteries in the central regions of the Volga Bulgaria during the pre-Mongol period (11th – first third of the 13th centuries) and is not characteristic of the Chyalyk culture.

Taken together, based on the combination of burial ritual features (overall Muslim tradition with some manifestations of paganism - deviations in orientation and position of the buried, presence of inventory, post-burial manipulations with the skeleton), as well as the composition and chronology of the artifact complex, the site can be attributed to the early stage of the Chiyalik culture.

### ***burial 5 (individual ID I19078, Female)***

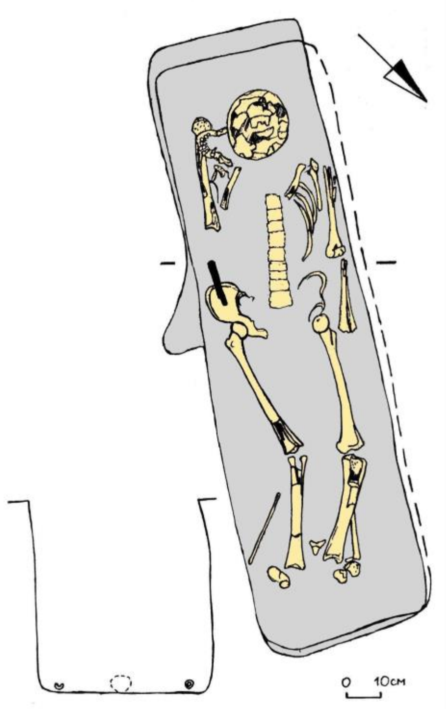

The burial was excavated in 2006. The grave has an elongated sub-rectangular shape with rounded corners, oriented towards the south-southwest (azimuth 216°), and measures 185 x 50 cm at the fixation level. The filling consists of dense gray loam with traces of reddish-brown clay. The walls are sheer, with the northwest (long) side slightly overhanging at the upper part, while the southwest side is disturbed by a rodent burrow. The depth of the pit from the fixation level is 58 cm, and the bottom is flat.

The skeleton of a female, approximately 25 years old, as anthropologically identified by Ilgizar Gazimzyanov, and poorly preserved, was positioned on her back with the head facing southwest, and the arms extended along the body.

***Figure SII.24.*** *Burial 5 at the Gulyukovo cemetery.*

The remains of the right forearm are placed on the right pelvic bone. The skull lies with the cranial part upwards, while the mandibula is displaced to the right, resting on the shoulder joint.  The legs are slightly bent at the knees and turned to the left. In the fill of the burial pit was found a shard of a Late Bronze Age pottery vessel, originating from the cultural layer of the settlement.

### ***burial 17 (individual ID I34074, Male)***

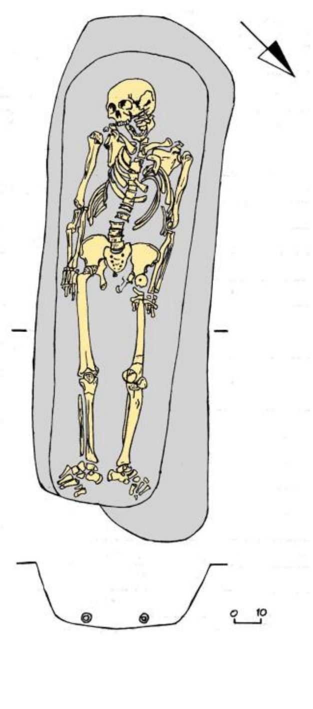

The burial was excavated in 2006. The grave pit is elongated in shape, with a rounded southwestern end and an irregular northeastern end, oriented southwestward (azimuth 230°). The dimensions of the pit at the fixation level are 200 x 70 cm. The fill consists of heterogeneous soil (dense gray loam with traces of reddish-brown clay). The walls are sloping, and the depth of the pit from the fixation level is 25 cm, with a concave bottom.

The skeleton was positioned elongated on its back with the head facing southwest; the arms were stretched alongside the body, with the radial bones passing under the edge of the pelvic bones. The skull lies on the occiput facing upwards, slightly tilted to the right (southeast); the feet are turned outwards. The buried person is male, aged 35-45 years, as anthropologically identified by Ilgizar Gazimzyanov.

***Figure SII.25.*** *Burial 17 at the Gulyukovo cemetery.*

The right ulna is fractured, with non-union of the fracture (the ends of the fracture overlap, a large bony callus "false joint" formed on the upper fragment of the ulna, and a bony outgrowth formed opposite the fracture on the radius). The vertebrae are deformed (flattened), and the vertebral body edges are sharpened; there are traces of fused fractures on the right ribs.

### *burial 20 (individual ID I19079, Male)*

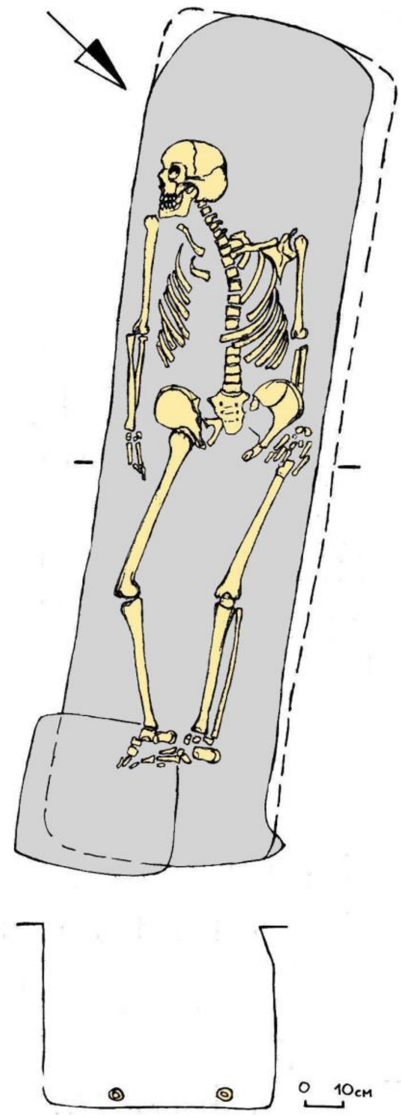


The burial was excavated in 2006. The grave pit is elongated in shape with rounded corners, oriented southwestward (azimuth 235°), measuring 225 x 56 cm at the fixation level. The fill consists of heterogeneous soil (dense gray loam with traces of reddish-brown clay). The walls are steep, with the northwest wall overhanging; the eastern corner of the grave pit up to a depth of -152 cm is damaged by post hole 11. The depth of the pit is 48 cm.

The skeleton of a male, aged 20-30 years, as determined by Ilgizar Gazimzyanov, was positioned elongated on its back with the head facing southwest with a slight turn to the right (southeast); the arms were stretched alongside the body, with the left forearm passing under the edge of the pelvic bone. The right leg is slightly bent at the knee. The skull lies on its right side, facing southeast.

In the fill of the grave pit a fragment of a vessel rim of unclear cultural and chronological affiliation (Late Sarmatian period?; Middle Ages?) was found, with the inclusion of coarse grog in the molding mass.

***Figure SII.26**. Burial 20 at the Gulyukovo cemetery.*

### *burial 31 (individual ID I25503, Male)*

The burial was excavated in 2006. The grave pit is elongated in shape with rounded ends, oriented southwestward (azimuth 233°), measuring 195 x 50 cm at the fixation level. The fill consists of heterogeneous soil (dense gray loam with traces of reddish-brown clay). The walls are steep, and the depth of the pit from the fixation level is 12 cm, with a flat bottom.

The skeleton of a male, aged 16-20 years, as determined by Ilgizar Gazimzyanov, was positioned elongated on its back with the head facing southwest; the arms were pressed

against the body (the deceased may have been wrapped?), with the left shoulder bone lying on the ribs, and the hands placed on the pelvis (the bones of the left forearm are displaced and lie on the sacrum and the right side of the pelvis, to the right of the spine). The skull lies on its right side, facing south. The feet are turned to the right (southeast).

In the fill of the grave pit, fragments of a vessel of unclear cultural and chronological affiliation (Late Sarmatian period?; Middle Ages?) were found, with the inclusion of coarse grog and sand in the molding mass (possibly natural impurities in the clay): 2 fragments of a flat slightly concave bottom and 2 fragments of walls.

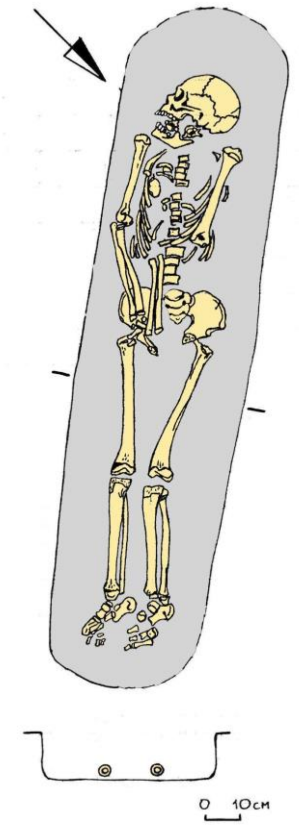


***Figure SII.27.*** *Burial 31 at the Gulyukovo cemetery.*

### *burial 51 (individual ID I19080, Female)*

The burial was excavated in 2007. The grave pit is elongated with a nearly rectangular shape and rounded corners in the western part, and a rounded eastern end, oriented southwestward (azimuth 227°), measuring 220 x 55 cm. The fill consists of heterogeneous soil (dense gray loam and reddish-brown clay in approximately equal proportions). The walls are slightly sloping, almost vertical, with a depth of 30 cm from the fixation level and a flat bottom.

The skeleton of a female, aged 30-40 years, as determined by Ilgizar Gazimzyanov, was positioned elongated on its back with the head facing southwest, and the anatomical order is partially disrupted. The preserved bones include the shoulder blades, right ulna, right pelvic bone, and leg bones (except for the left femur); the feet are turned to the right. Partially preserved are the original positions of the left femur (rotated 90° around its longitudinal axis, with the greater trochanter upward and the head of the femur downward) and the hands (located on the outer side of the pelvic bones). Based on the position and orientation of the preserved in situ long bones, the arms of the deceased were likely extended along the body.

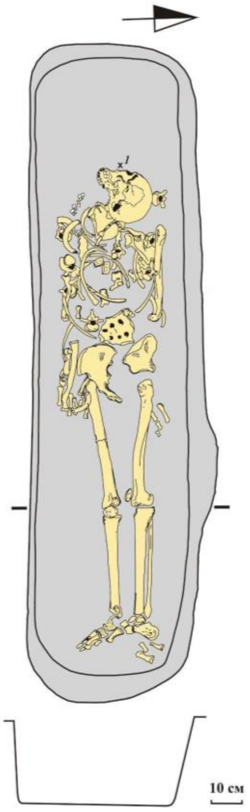


***Figure SII.28.*** *Burial 51 at the Gulyukovo cemetery.*

The skull is inverted, lying on the right temporal bone with the facial aspect facing southwest; other bones such as the mandibula, ribs, sternum, scapulae, clavicles, and vertebrae

(including the sacrum) are scattered in the chest area of the deceased; the left pelvic bone is rotated with the iliac crest upward. Overall, the displaced bones are close to their original positions. It is difficult to determine whether the disruption of the anatomical order of the skeleton was caused by animal burrowing activity or intentional human actions (robbery? ritual post-mortem treatment?) with certainty.

In the western part of the grave pit, in front of the facial aspect of the skull, there was a narrow, thin copper plate (unfolded rim of a copper vessel?) with a elongated-triangular hole at one end (a pendant?) (preserved in three fragments); within the fill of the grave pit, without a precise location, there is a small flint knife-like plate (a knife with two blades for cutting soft material and a point as identified by Madina Galimova), originating from the cultural layer of the settlement.

### *burial 60 (individual ID I19081, Male)*

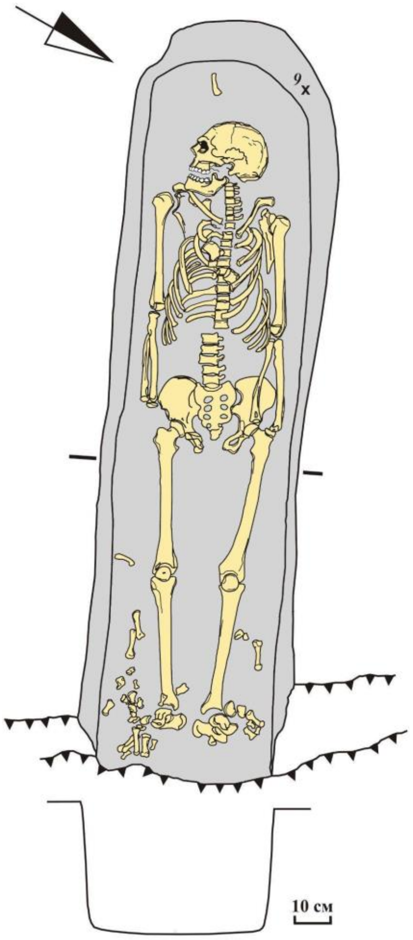


The burial was excavated in 2007. The grave pit has an elongated shape, oriented southwestward (azimuth 250°), with the eastern end destroyed by an edge, and the western end irregularly rounded. The preserved portion measures 195 x 58 cm at the fixation level, while at -220 cm depth, the length increases to 210 cm due to the slope of the edge that destroyed the eastern end of the pit. The fill is heterogeneous: dense dark-gray loam with occasional inclusions of reddish-brown clay (across the entire area of the pit at the primary fixation level, and in the middle part of the pit and below) and variegated fill (dense gray loam and reddish-brown clay in approximately equal proportion) (along the walls and at the bottom, at the contouring level and below). The walls are slightly sloping, almost vertical, and uneven in the western (end) part; the depth from the fixation level ranges from 51 to 54 cm, with a flat bottom.

The skeleton of a male, aged 25-35 years (?), as determined by Ilgizar Gazimzyanov, was positioned elongated on its back with the head facing southwest, and the arms extended along the body, with the left forearm lying on the left hip joint. The skull is lying on the right side, facing south.

***Figure SII.29.*** *Burial 60 at the Gulyukovo cemetery.*

The hands are missing; the foot bones (all phalanges, part of the metatarsals, and cuneiform bones) are displaced and unevenly distributed in the fill, mainly in the eastern half of the pit: some phalanges and metatarsals were found in the upper fill layer; the majority of the phalanges and metatarsals formed a cluster at the bottom of the eastern end of the pit, primarily between the right shinbone and the southeast wall of the grave; one cuneiform bone

was found at the western end of the grave, behind the skull. This is likely the result of animal burrowing activity, although the outlines of burrows in the fill are not evident.

In the upper fill layer at the western end of the pit, a flint chip without traces of use was found. Additionally, from the fill without a precise location, fragments of flint plates (chisels for wood, cutters, and knives) were recovered, as identified by Madina Galimova, as well as small fragments of ceramic vessel walls from the Volga-Kama Neolithic culture and the Late Bronze Age (?); all findings originate from the cultural layer of the settlement.

### *burial 63 (individual ID I33730, Female)*

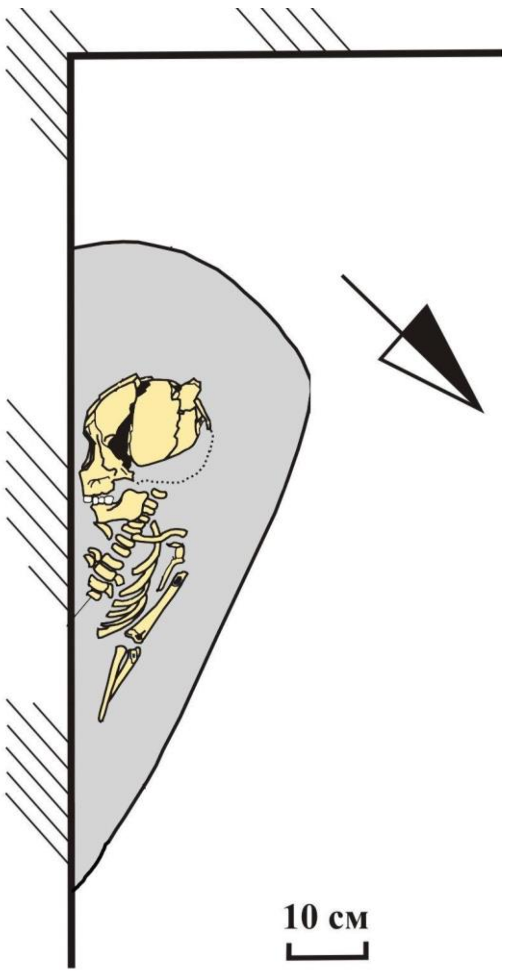


The burial was excavated in 2007. The contours of the bottom part of the grave pit, elongated with a rounded end, oriented southwestward (azimuth 247°), were traced at a depth of -182 cm; the majority of the burial remains outside the excavation area, with the portion that entered the excavation measuring 80 x 30 cm at the fixation level. The fill is variegated (dense gray loam with inclusions of reddish-brown clay). The walls were not identified during clearing; the profile of the southeast wall of the excavation reveals overhanging of the end wall of the burial pit. Judging by the profile, the depth of the pit from the surface level is 40 cm, with a flat bottom slightly raised towards the walls.

The skeleton of a child, approximately 5 years old, as determined by Ilgizar Gazimzyanov, partially within the excavation area (upper half down to the thoracic region of the spine), was positioned elongated (?) on its back with the head facing southwest; the left arm is extended along the body. The skull is lying on the right side, facing southeast.

***Figure SII.30.*** *Burial 63 at the Gulyukovo cemetery.*

## <u>II.I.2. Ust-Menzelya hillfort</u>

The hillfort at the Ust-Menzelya locality has been known since 1929. The cultural layers and finds from the enclosure determine the Early Iron Age (Pyany Bor culture) as the main period of inhabitants, with layers from the Eneolithic period (Novoilyinka and Garino cultures); sporadic finds include ceramics from the Neolithic period (Kama culture) and from the end of the Bronze Age to the beginning of the Early Iron Age (Lugovoy culture, Textile Ware, or Ananyino culture). The burials in the fortified area were exposed by ravine erosion and documented in 1998 by L.V. Mil’chakov in 1998 (hunting inspector for the Menzelinsk and Aktanysh districts of the Republic of Tatarstan; one partially destroyed non-inventoried burial

was inspected). In 2001, during a survey by Dmitry Bugrov, in a control trench approximately 1.5 m2 of heavily damaged child burial (3-4 years old, with undetermined cultural-chronological position) was unearthed. In 2007 Dmitry Bugrov and Madina Galimova carried out excavations of a total area of 183.5 m2, which yielded five burials identified as "early Muslim" and attributed to the early phase of the Chiyalik culture.

***burial 5 (individual ID I33733, Male)***

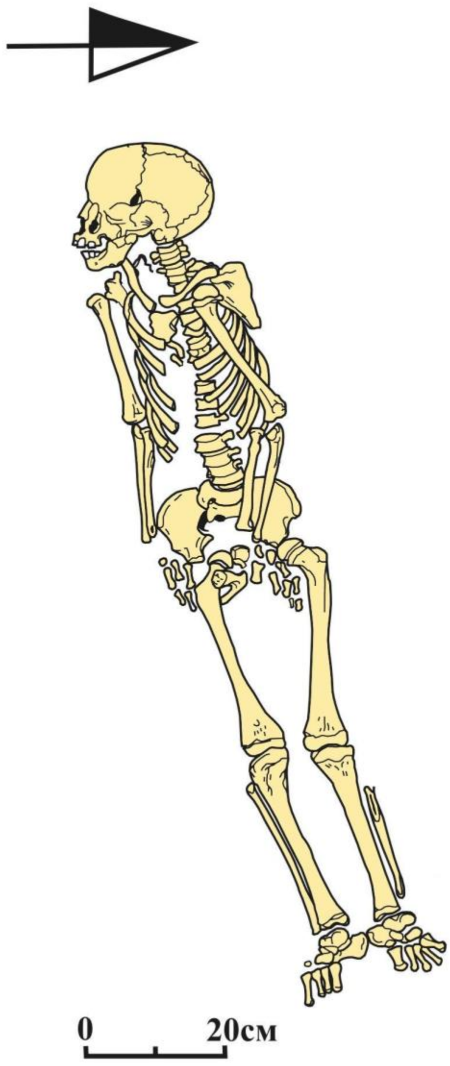


The outline of the burial pit was not traced; the skeleton of a child aged 8-10 years old as determined by Ilgizar Gazimzyanov, was found in a layer of yellow clay with admixtures of lime crumbs and with streaks and layers of gray-brown clay. It was oriented with the head to the southwest; the legs were extended, the right foot was turned outward, the left was extended; the torso from the pelvic bones and above was turned to the right side, the left arm was slightly bent at the elbow, lying over the body with the hand on the pubic bones, the right arm was extended along the body, the humerus with the proximal end passed under the right ribs, the forearm bones under the outer edge of the right ilium, the phalanges and metacarpals under the greater trochanter of the right femur. The skull lies on the right side, with the facial part to the south.

A fragment of an unornamented wall of a ceramic vessel with admixtures of crushed shell in the molding mass (Pyany Bor culture) was found at the level of excavation; it originates from the cultural layer of the hillfort.

***Figure SII.31.*** *Burial 5 at the Ust-Menzelya hillfort.*

## II.I.3. Zuevy-Klyuchi hillfort

The site is located on the right bank of the Kama River, 1.5 km southwest of the village of Zuevy Klyuchi in the Karakulinsky district of the Udmurt Republic. The settlement occupies a promontory with a roughly triangular shape on the slope of the southern exposure of the third floodplain terrace, formed by two steep slopes with a depth of 20-25 m. Excavations conducted by R.D. Goldina, V.F. Gening, and L.I. Ashikhmina yielded approximately 5000 square meters of the unearthed cultural layer, yet the site remains largely unpublished. Materials from the settlement were published by V.F. Gening and L.I. Ashikhmina in 1984, with a characterization of early Iron Age dwellings identified at the site in 1986. From 1997 to 2006, the hillfort and

several adjacent sites were excavated by E.M. Chernykh. To the west, across the ravine, lies the Zuevy-Klyuchi burial ground of the Anan'ino period, excavated by A.A. Spitsyn in 1898 (unpublished).

In the course of the excavations of the Zuevy-Klyuchi hillfort in 1971-1972, at the southeastern edge of the promontory and beneath the cultural layer of the Late Anan'ino period, numerous burials were unearthed. 25 of them were attributed to the Seima-Turbino period, as reported by V.F. Gening in 1975 while the others were dated to the Late Anan'ino and "later" periods. The anthropological material from the excavations was stored in the Tomsk State university.

In total, we sequenced eight individuals from the burial ground at the Zuevy-Klyuchi hillfort. Five of them were dated by the 14C, and all of them show similar ages between 1227 and 1394 CE. The other three individuals are genetically close to the distinct group of the dated samples. Based on this observation, we attribute all eight individuals from Zuevy-Klyuchi to the Late Medieval period.

### *burial 30 (individual ID I30341, Female)*

The grave pit, of sub-rectangular shape measuring 120 x 80 cm, oriented from the west-northwest to the east-southeast, was identified at a depth of -63 cm. The depth of the pit is 16 cm. The burial contained a skeleton in satisfactory preservation, lying in the supine position, with the head to the west-northwest. The upper part of the skeleton, from the position on the back, is partially turned onto the right side, the left arm is semi-flexed at the elbow, and the right arm is extended alongside the body. Near the knee joint of the left leg of the deceased, a fragment of pottery was found at a depth of -73 cm from the surface, and a human tooth was found in the northeast part of the burial.

The determined radiocarbon age of the burial is 1292-1395 cal CE (635±20 BP, PSUAMS-13158) (based on human bone).

### *burial 34 (individual ID I30342, Female)*

Human bones were discovered in the sod-plowed layer at a depth of 14 cm in the southeastern corner of the plot. The skeleton is heavily damaged by plowing and is preserved fragmentarily, with better preservation of the leg bones. Due to the damage to the skeleton, it is difficult to judge its orientation. Apparently, it was oriented from the northwest to the east, with the head to the northwest. Two pottery fragments were found near the skeleton: the first near the presumed pelvis at a depth of 14 cm from the surface, and the second at a depth of -24 cm near the knee joint of the right leg.

The determined radiocarbon age of the burial is 1303-1401 cal CE (605±20 BP, PSUAMS-13394) (based on human bone).

For eight more graves from Zuevy Klyuchi hillfort the archaeological context description is not available. These are burial 43 (individual ID I32547, Female), burial 42 (individual ID I30343, Female), burial 45 (individual ID I32546, Female), burial 101 (individual ID I32801, Female), burial 102 (individual ID I32802, Male), burial 103 (individual ID I32803, Female), burial 104

(individual ID I32804, Female). Unfortunately, the archaeological descriptions from these graves are missing in the field report on the excavations.

The determined radiocarbon age of the burial ID I32547 from grave 43 is 1296-1394 cal CE (635±15 BP, PSUAMS-12694), ID I30343 from grave 42 it is 1318-1407 cal CE (585±15 BP, PSUAMS-13159), and ID I32546 from grave 45 is 1227-1278 cal CE (775±15 BP, PSUAMS-12708). All dates were obtained from human bones.

## III. The Middle Kama and Cis-Urals (including the Belaya Basin)

### III.A. The Early Iron Age Pyany Bor culture in the Southern Cis-Urals (Belaya_Pyany-Bor)

**Genetic results.** *In this group, we analyzed ten individuals from the sites of Starokirgizovo (n=5) and Kipchakovo (n=5). All 10 individuals had sufficient coverage for downstream population genetic modeling (>100,000 SNPs), and 8 were suitable for IBD-sharing analysis (>600,000 SNPs). No close relatedness was detected among these samples, and the female-to-male ratio was 1:9. The samples displayed a genetic composition similar to that of the Low Kama region Iron Age groups. The supervised ADMIXTURE analysis revealed elevated levels of Samara_EBA_Yamnaya and Yakutia_LNBA ancestries in this group. The Belaya_PyanyBor samples are predominantly clustered within the Eurasian Steppe Iron Age (IA) cluster. Similar to the Low Kama Pyany Bor groups, these genetic results suggest the presence of a previously undescribed Iron Age population in the region.*

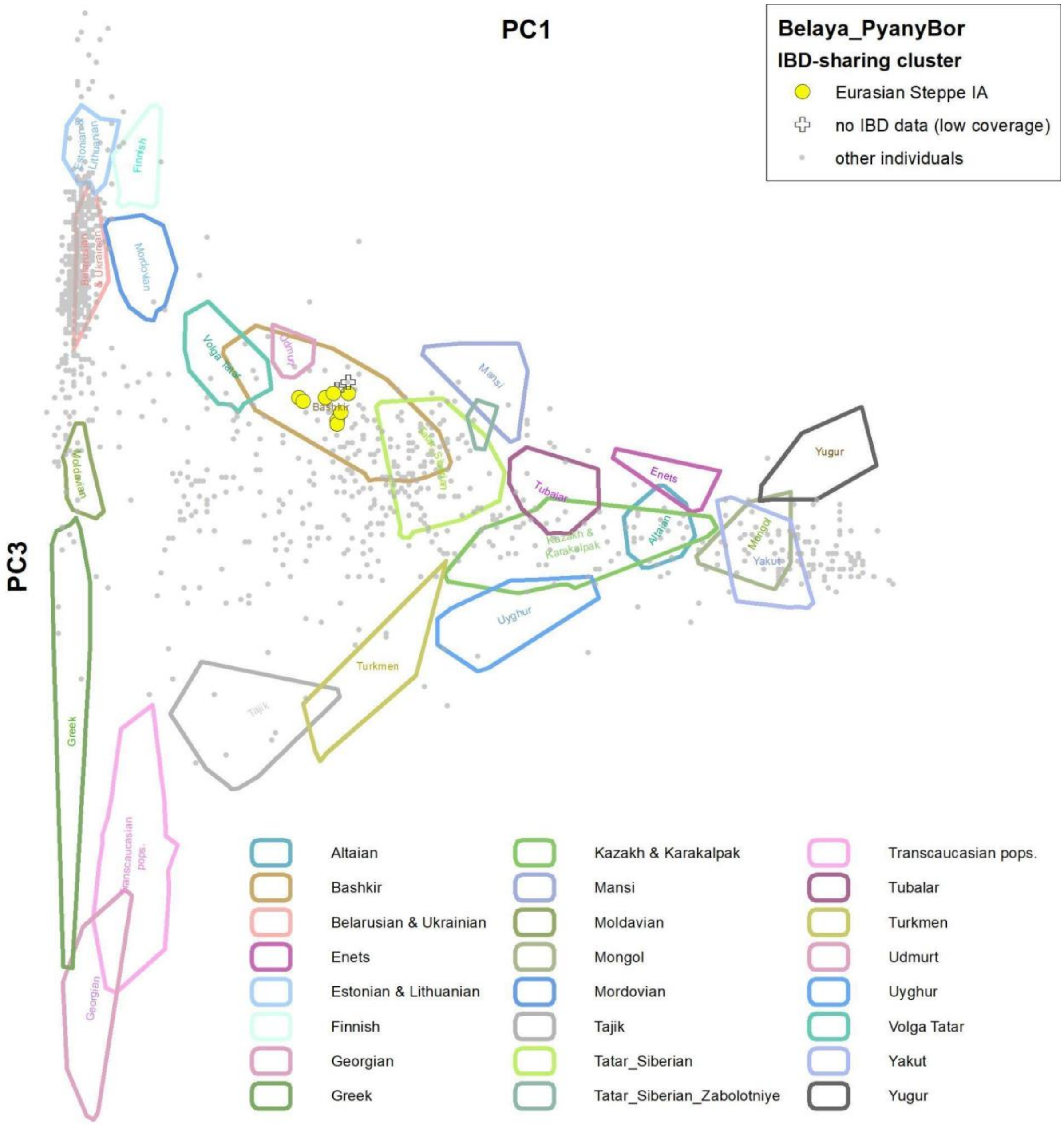


***Figure SIII.1.*** *Eurasian-scale principal component analysis (PCA), with a projection of the newly sequenced individuals onto modern genetic variation following Jeong et al. 2019. The PC1 and PC3 dimensions are shown, including the newly presented genomes. Polygons represent example populations from modern Eurasian individuals onto which the ancient samples were projected, while grey dots indicate ancient reference samples from our IBD-sharing network.*

## III.A.1. Kipchakovo burial site

The Kipchakovo burial site is located 1 km east of the village of Minnyarovo (Aktanyshsky district, Republic of Tatarstan) and 2.5 km southwest of the village of Kipchakovo (Ilishovsky district, Republic of Bashkortostan), on the main terrace of the right bank of the Syun River, 70-80 m southwest of the outer rampart of the Kipchakovo settlement.

Over the course of four field seasons (1990–1991, 1994, and 2001), F.M. Tagirov excavated 4 kurgans (numbered 15, 41, 51, 52) and two adjacent excavations, one near kurgan 41 (Excavation 1) the other near kurgan 15 (Excavation 2). A total of 64 burials were excavated during this time. Later, the site was excavated by Sergei Zubov.

We sequenced five individuals from the site, from the graves burial 12 (individual ID I27117, Male), burial 15 (individual ID I27017, Male), burial 19 (individual ID I27018, Male), burial 6 (individual ID I27020, Male), burial 61 (individual ID I27019, Male).

### III.A.2. Starokirgizovo burial ground

The burial site at Staro-Kirgizovo village, Ilishovsky District, Republic of Bashkortostan, is located on the right bank of the Myadym (Minueshta) River, a tributary of the Belaya River, which flows into the Kama River. The site lies on the eastern outskirts of the village near the tractor park and fire station, on a prominent elevation formed by the edge of the right floodplain terrace and a ravine.

The site was first discovered by A.Kh. Pshenichnyuk in 1971, with initial excavations uncovering two burials (though details are unavailable in reports, and materials are not preserved in museum collections). Subsequent excavations in 1971–1972 revealed 51 or 53 additional burials. In 2016–2017, further investigations led by N.A. Lifanov uncovered 75 more burials.

The buried individuals were interred in extended supine positions, oriented with their heads to the south or southwest. The burials contained a rich assemblage of artifacts, including adornments (beads, temple pendants, bronze plaques, pendants, and openwork overlays), weapons (arrowheads and knives), and ceramics, reflecting the cultural and material complexity of the site.

#### ***burial 25 (individual ID I16644, Female)***

The burial pit was not documented and had been completely destroyed by several trenchings; two lines of cables (now inactive) ran through the burial. Overall, the skeletal remains retained anatomical order, although some bones were displaced, mixed, or missing. The buried woman, aged between 45 and 65 (closer to 65), was laid out extended on her back with her head to the northeast.

The burial inventory included items made of bronze, iron, and glass. Some of them, apparently, had been displaced from their original positions. For instance, fragments of bronze temple spiral pendants were found in various locations (to the northeast and southwest of the skull, near the right elbow, abdomen, sacrum, and by the right knee). Two round bronze plaques were found to the east of the skull and on the sacrum. Three round bronze discs were discovered near the right pelvic bone, and a bronze rhombic plaque was found under the pelvis. Iron items were concentrated in the lumbar-pelvic region. Fragments of iron items (knives?) were found around the abdomen and under the right pelvic bone. Fragments of iron bracelets were found on both sides of the pelvis, and an iron waist hook was fixed to the southeast of the skull. Yellow glass beads, totaling five specimens, were located to the east of the skull, on the sacrum, and by the right knee. Blue glass beads (two specimens) were

found to the east of the skull and on the right side of the chest. Adjacent to the latter were green beads. A cluster of various beads, including white and blue, gilded, as well as polychrome beads ornamented with longitudinal blue and white stripes (totaling 49 specimens), was cleared on the left side of the chest.

The determined radiocarbon age of the burial is 94 cal BCE - 58 cal CE (2030±20 BP, PSUAMS-10313) (based on human bone).

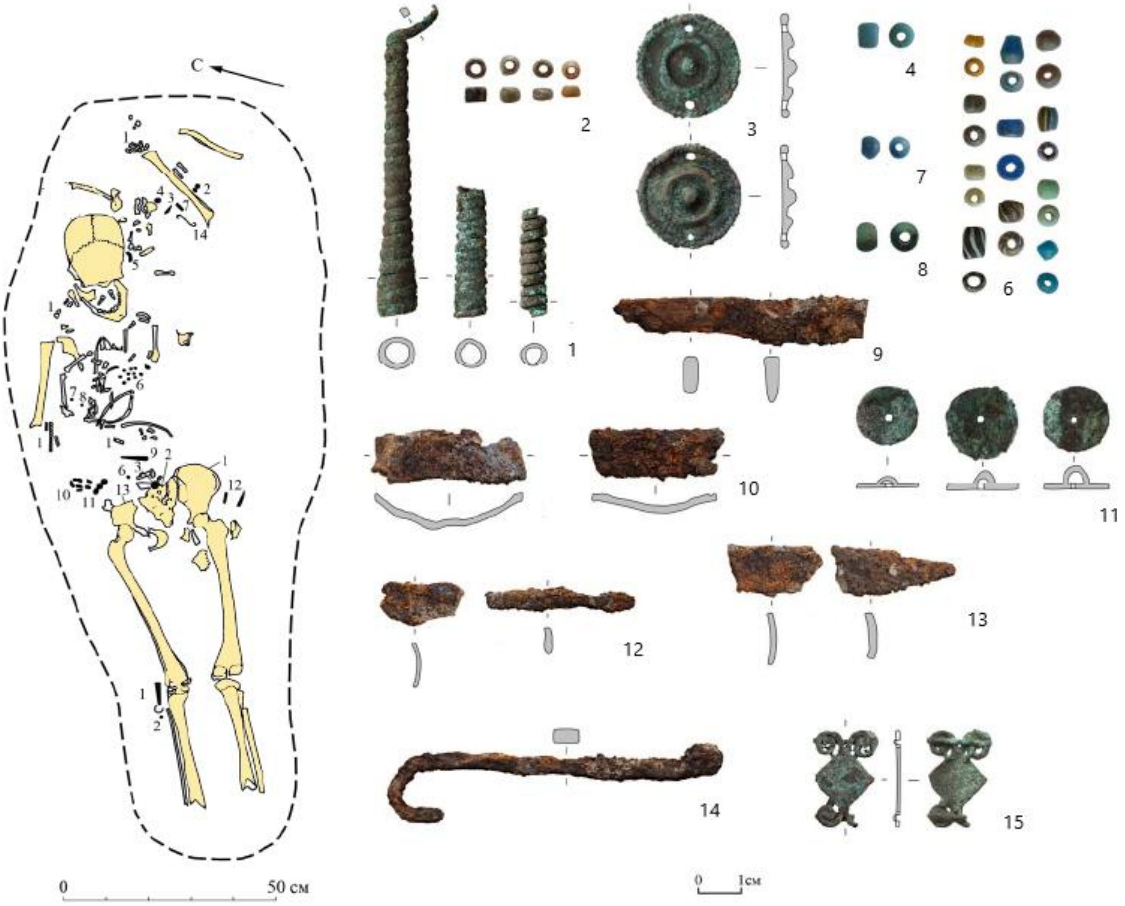


***Figure SIII.2.*** *Burial 25 at Starokirgizovo cemetery.*

### *burial 42 (individual ID I16645, Male)*

The burial pit was elongated with rounded corners and oriented along an east-west axis. The dimensions of the burial pit were as follows: length – 224 cm; width – 68-75 cm; maximum depth of the pit from the discovery level – 21 cm (at the western wall). The walls were steep, and the bottom was flat.

Overall, the skeletal remains retained anatomical order, although some bones had decayed. A cluster of small chalk fragments was found under the skull. The buried man, aged between 20 and 28, was laid out extended on his back, with his head oriented towards the eastern sector.

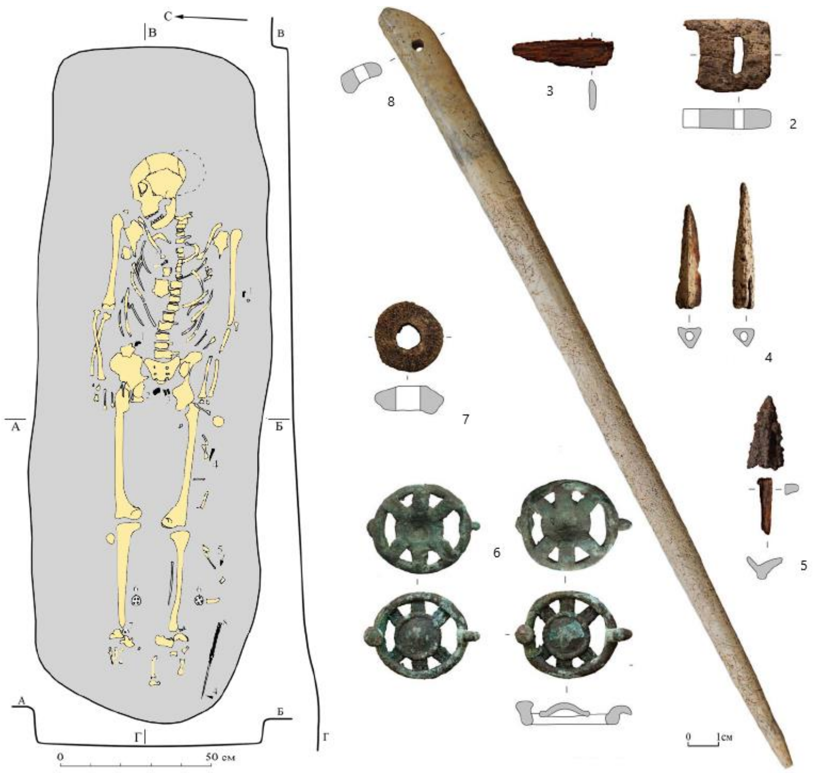

The burial inventory mostly retained its in situ position. Fragments of a bone buckle and a wooden object with iron oxides were found between the pelvic bones. Two bone triangular socketed arrowheads, one of which showed traces of iron oxide, were found near the left femur and the western wall of the pit. Fragments of an iron three-bladed tang arrowhead with remnants of a shaft were found between the left tibia and the southern wall of the burial pit.

***Figure SIII.3.*** *Burial 42 at Starokirgizovo cemetery.*

South of each of the major tibia bones, there was a bronze circular framed buckle with a single pin, likely belonging to footwear. Among the bones of the right foot, a bone disc with a through hole in the center was found. In the southwest corner of the pit, adjacent to the bones of the left foot, there was a long bone awl with a hole. Additionally, fragments of burnt animal bone were found near the left scapula and right pelvic bone.

### *burial 53 (individual ID I16643, Male)*

The burial pit was elongated with rounded corners and oriented along a northwest-southeast axis. The dimensions of the burial pit were as follows: length – 200 cm; width – 70 cm; maximum recorded depth of the pit from the discovery level – 19 cm (at the southern wall). The walls were mostly steep, and the bottom was flat, with a slight slope towards the south.

Overall, the skeletal remains retained anatomical order, although some bones were displaced due to earthmoving activities. The buried child, aged 7-8 years, was laid out extended on their back, with the head oriented towards the north-northwest. The arm bones were laid alongside the body, and the skull was positioned on the base of the skull and the occipital bone, slightly tilted to the left side.

To the left side of the skull, a bone socketed arrowhead with a rhombic cross-section was found. In the neck area, west of the skull, two yellow beads and one blue bead were found east of the skull. Near the left shoulder, a small fragment of calcined animal bone was found. In the waist area, there was a rectangular bone buckle with a fixed hook and two through slots perpendicular to the buckle axis for threading a belt. Three bronze elliptical eyelets were found near the left wrist and hand. Another similar eyelet was found between the buried individual's

thigh bones, next to which was a triangular bone socketed arrowhead. Another bone socketed arrowhead with a rhombic cross-section was found near the right knee.

On the outer side of the right thigh bone, there was a poorly preserved iron knife approximately 30 cm long. The knife was in wooden sheaths, from which wood decay was detected during clearance. In the area of the handle, near the thigh, there was a trapezoidal bone clasp with a slit in the wide part and two oval-shaped holes in the narrow part. Judging by its position, the clasp served to attach the sheaths to the belt. Four claw phalanges of an adult badger from the front limbs were found on this clasp. Nearby, on the inner side of the right thigh bone, a stone spindle whorl with a hole was found.

Between the knife and the upper part of the thigh bone, from top to bottom, there were the following: a permanent premolar of an adult wild boar from the right mandibula, a canine tooth of an adult horse from the left upper jaw (with an artificial round hole in the root and the end of the root cut off), a fragment of the canine tooth of an adult wild boar from the right upper jaw (with an artificial round hole in the root), and a fragment of a jawbone of a badger with a canine tooth.

In the area of the knife tip, near the right shin, there was a fastening for attaching the end of the sheath to the leg in the form of a composite "bracelet". This consisted of a bronze clasp with a fixed hook of elongated trapezoidal shape, decorative bone plates of bow-like shape with a central through hole for a leather strap (7 pieces), and separating bronze eyelets between them (7 pieces). The terminal part of the attachment was decorated with five bone pieces with a central hole, imitating animal fangs. Below the knife sheath, a circular bronze plate with a hole in the center and two ears on the back for threading a strap was found. This plate was presumably part of the ornamentation system for attaching the terminal part of the combat knife to the leg.

On the inner side of the right thigh bone, there was a complex consisting of cut upper and mandibles of small predatory animals, originating from at least two badgers, two foxes, and one otter. From top to bottom (from the groin to the knee), cuts made during the detachment of the front part of the skull were evident on the upper parts of the jaws. The cut passed through the ends of the roots of the upper canines.

Between the legs, in the area of the left foot, a cluster of bone socketed (18 pieces) and iron three-bladed tang arrowheads (6 pieces), apparently stored in a quiver, was found. At the end of the shins, two bronze round shoe fasteners with a fixed hook and a corresponding pin were discovered.

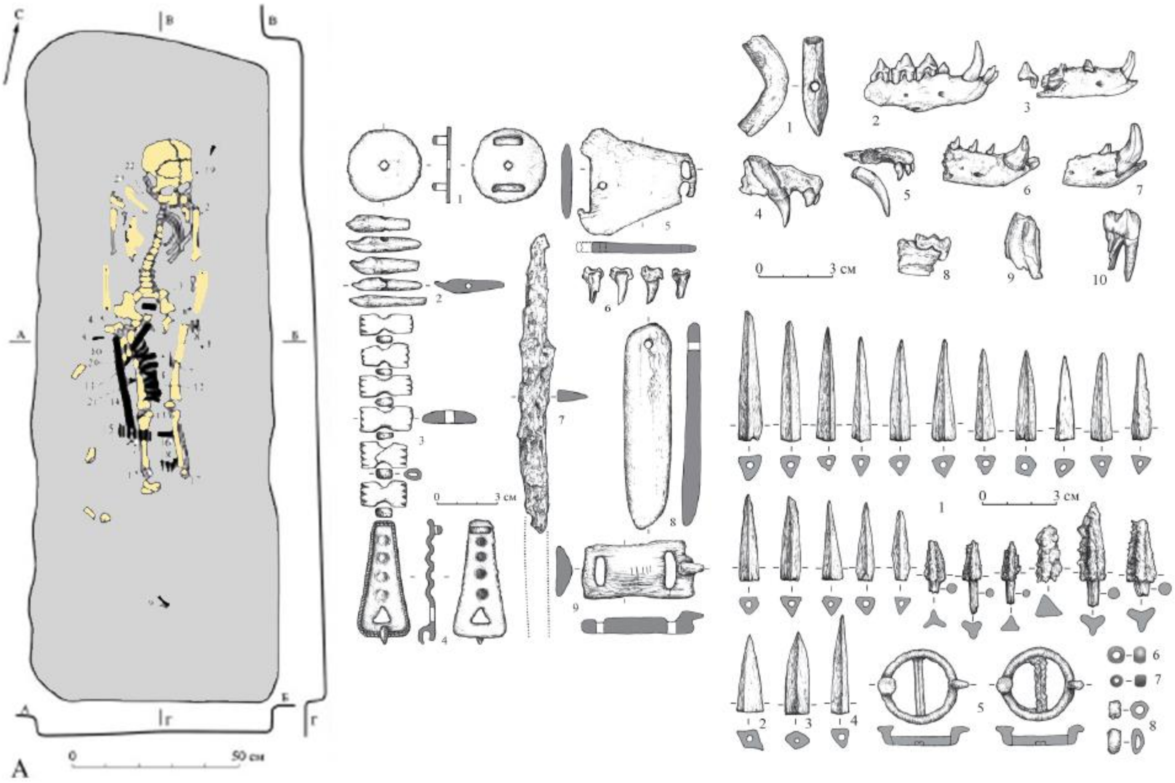

***Figure SIII.4.*** *Burial 53 at Starokirgizovo cemetery.*

### *burial 58 (individual ID I16642, Male)*

The burial pit was elongated with rounded corners and oriented along a west-east axis. The dimensions of the burial pit were as follows: length – 164 cm; width in the central part – 56 cm; the maximum recorded depth of the pit from the discovery level – 28 cm (in the western part). The actual depth of the pit from the ancient soil surface, judging by the profiles, was at least 45 cm. The walls of the pit were mostly steep, and the bottom was flat with a slight slope towards the west and south.

The skeletal remains were partially damaged by the cable trench. Four arrowheads were found in the burial: two triangular bone socketed and two three-bladed bronze socketed arrowheads. The bronze arrowhead was positioned in the left part of the chest of the buried individual, with the tip pointing south, while the second bronze arrowhead and both bone arrowheads were scattered disorderly along the left shin of the skeleton, likely displaced as a result of the intervention that also damaged the foot bones.

Along the right thigh of the buried individual, an iron knife was found, with remnants, likely part of its handle, consisting of small fragments of iron and wood, near the right wrist. A fragment of a bone was fixed in the chest area, and a complete round bronze buckle was found near the left thigh. On the right side of the skull, a silver(?) pendant was unearthed.

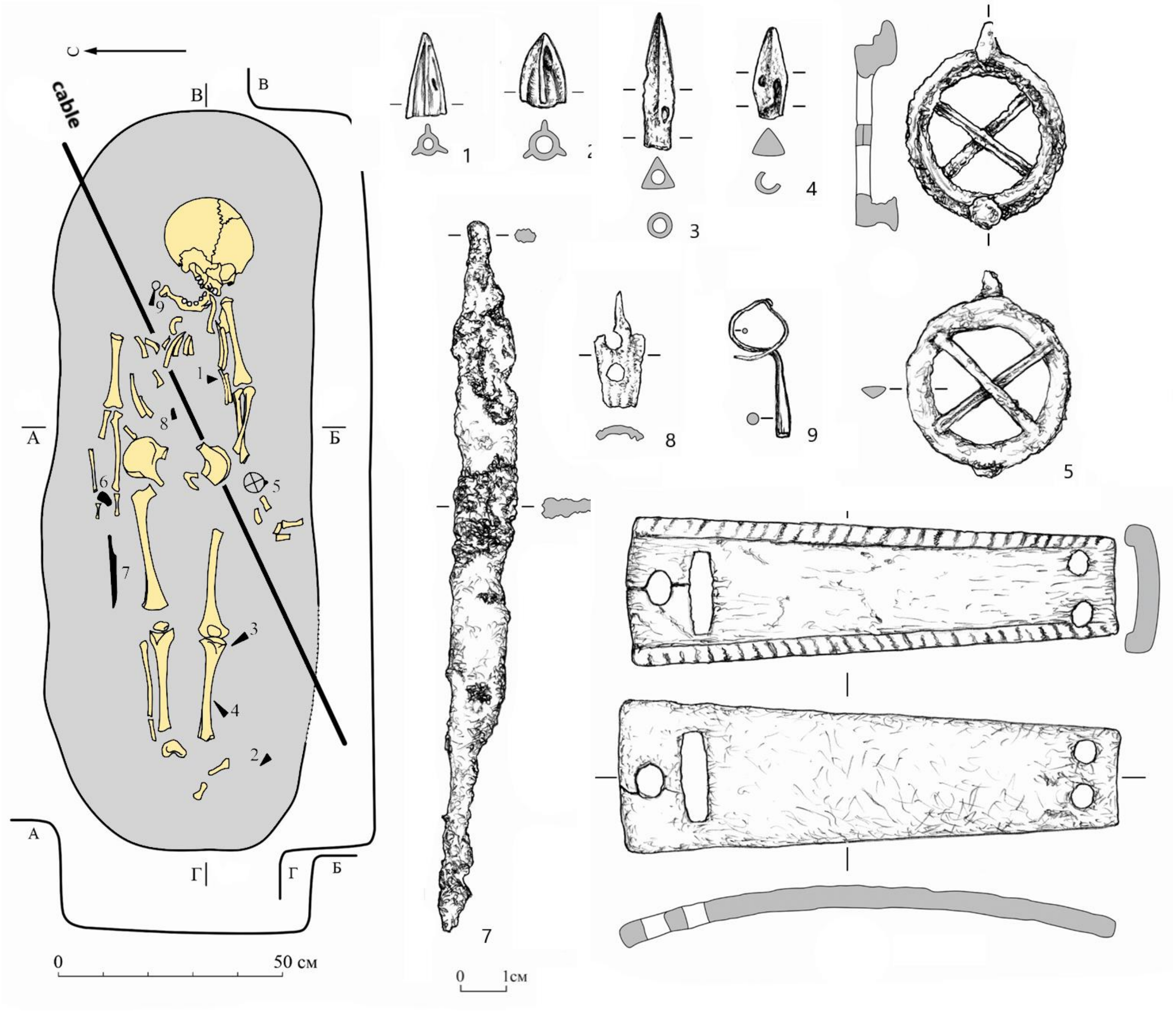


***Figure SIII.5.*** *Burial 58 at Starokirgizovo cemetery.*

## *burial 63 (individual ID I16641, Male)*

The burial pit was elongated with rounded corners and oriented along a west-east axis. The dimensions of the burial pit were as follows: length – 208 cm; width – 70 cm; the maximum recorded depth of the pit from the discovery level – 17 cm (at the western wall). The walls of the pit were steep, and the bottom was flat with a slight slope towards the west and south.

The skeletal remains of an adult male, approximately 45-55 years old and in good condition, were located closer to the southern wall of the pit. The buried individual was lying stretched out on his back with the head to the east, the right arm stretched along the body, and the left hand on the pelvic bone.

The burial inventory included an iron knife lying above the right femur and a set of quiver remnants near the left shin. The attachment of the quiver to the leg was marked by bronze spiral-wound plates around the tibia bones and a bronze pin. The best-preserved plate was wound three times, while the others were fragmented. The pin had a truncated biconical shape. Judging by the preserved decay, the quiver was made of leather. Its contents consisted of arrowheads made of bronze and bone with partially preserved shafts. There were 18 bronze

three-bladed socketed arrowheads and 2 triangular bone socketed arrowheads. Fourteen shaft fragments were found.

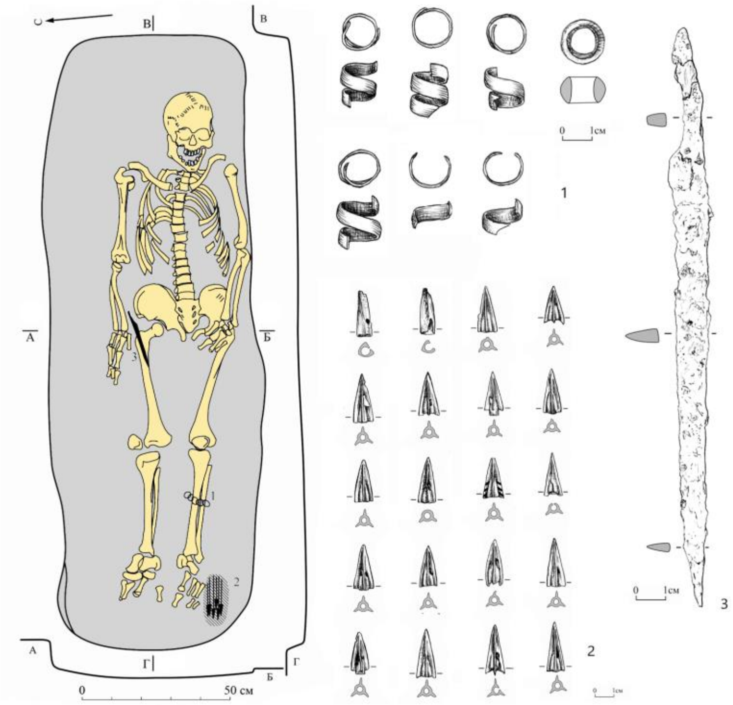

***Figure SIII.6.*** *Burial 63 at Starokirgizovo cemetery.*

## III.B. The Migration period Mazunino culture in the Southern Cis-Urals (Belaya_Mazunino)

The description of the Mazunino culture assemblage of the Lower Kama region was given in the II.D section of this Supplementary material.

In the Cis-Ural area, the Mazunino sites demonstrate similar cultural traits. In this region, however, the Mazunino sites do not disappear after the 5th century CE, but gradually evolve to the Bakmutino cultural tradition.

The first finding of the artifacts later attributed to the Bakhmutino tradition became known in the late 19th century (Bulychov, 1902), and systematic research started from the 1920s. In the late 1950s and 1960s, the Bakmutino cultural assemblage was characterized as a distinct phase of the local cultural development (Mazhitov, 1963; 1968).

Fortified settlements with ramparts and ditches attributed to the Bakhmutino culture are located on high floodplain terraces, while settlements are situated on lower ones. Dwellings consisted of above-ground log structures up to 120 square meters with hearths and storage and cash pits. The pottery of the early period is represented by small, low-necked round-bottomed bowl-shaped vessels ornamented with dimples across the entire surface; of the late period - by large vessels of the same type ornamented with dimples and triangular punctures across the entire surface, with incisions on the rim and shoulders. The main branches of the economy were animal husbandry, agriculture, hunting, fishing, iron, and bronze metallurgy.

The Bakhmutino burial grounds are flat, with rows of grave pits. During the early phase of the culture, the deceased were buried in shallow graves in a supine position. The burial inventory consisted of tools (spindle whorls, iron knives, awls, sharpening stones, and sickles), weapons

(iron and bone arrowheads, spears, axes), decorations (bronze bracelets, temple pendants, neck rings, appliqué plaques, pendants in the form of ducks, bears, necklaces made of glass beads). The "sacrificial complexes" consisting of women's jewelry are specific details of the Early Bakhmutino burials.

In the late period, the deceased were buried in deep graves (sometimes in wooden log coffins or wrapped in bark) in a supine position. Clay vessels and remnants of sacrificial food (horse and sheep bones) were found at the head, in the corners. Male burials were characterized by the presence of weapons (iron arrowheads, spears, swords), and female burials by jewelry (bronze, gold, silver lunar pendants, zoomorphic temple pendants, bronze mirrors, bronze and iron buckles). For adult burials, the presence of belt fittings (bronze and silver overlays, buckles, pendants, belt tips, including those covered with gilded foil) was characteristic.

We publish four individuals from the Cis-Ural Birsk-2 cemetery, the three are attributed to the Mazunino culture and one to the early phase of the Bakhmutino tradition.

**Genetic results.** *In this group, we analyzed 4 individuals from the site Birsk-2 (n=4). Of these, 2 had sufficient coverage for downstream population genetic modeling (>100,000 SNPs), and 2 were suitable for IBD-sharing analysis (>600,000 SNPs). No close relatedness was detected among these samples, and the female-to-male ratio was 1:3. Samples show similar genetic composition to the Low Kama region Iron Age groups. The supervised ADMIXTURE analysis revealed an elevated level of Samara_EBA_Yamnaya and Yakutia_LNBA ancestries in this group. These samples did not belong to any of the IBD-sharing clusters. This group exhibited genetic similarities to the Mazunino group from the Low Kama region, suggesting a shared ancestry across the wider area.*

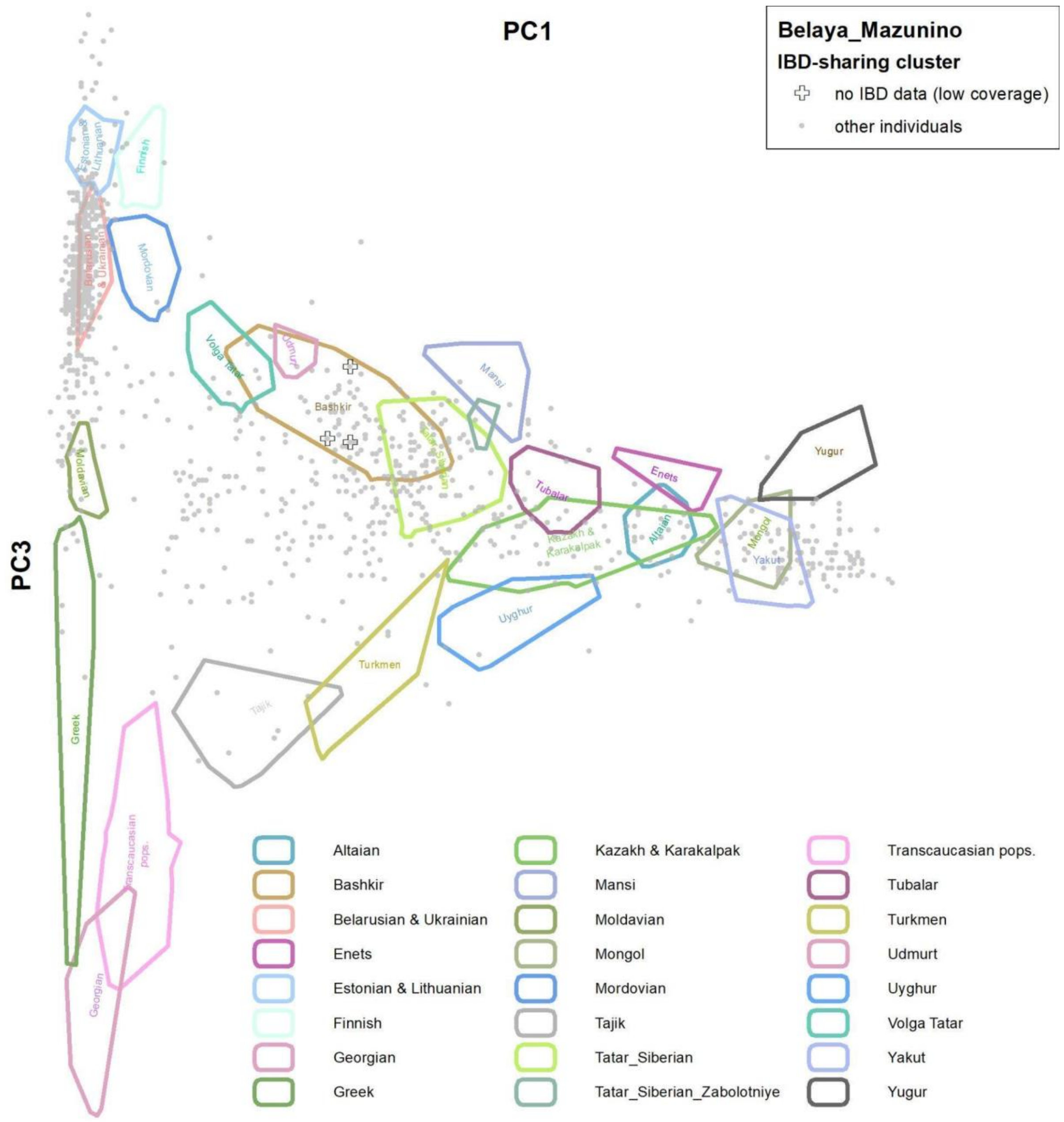


***Figure SIII.7.*** *Eurasian-scale principal component analysis (PCA), with a projection of the newly sequenced individuals onto modern genetic variation following Jeong et al. 2019. The PC1 and PC3 dimensions are shown, including the newly presented genomes. Polygons represent example populations from modern Eurasian individuals onto which the ancient samples were projected, while grey dots indicate ancient reference samples from our IBD-sharing network.*

## III.B.1. Birsk-2 burial ground

The Birsk-2 burial ground is located at the southern outskirts of the city of Birsk and occupies a vast area of the native terrace of the right bank of the Belaya River, 0.6 km northeast of the Birsk (Chertovo) hillfort. The total area of the site reaches 350x450 m or approximately 157,000 m².

The Birsk-2 burial ground has been known since 1902, when N.I. Bulychev described finds stored in the Ufa Governorate Museum (now the National Museum of the Republic of Bashkortostan) (Bulychev, 1902). Over many years of research on the burial ground, conducted by Niyaz Mazhitov, six excavations were conducted, and 692 burial complexes dated to the 8th-10th centuries CE were studied (Mazhitov, 1968). In 2017 and 2021, the work was continued by Rida Ruslanova.

### *burial 694 (individual ID I19264, Male)*

The burial pit of a sub-rectangular shape and oriented south-south-east - north-north-west was discovered at a depth of -0.62. It measures 1.9x0.64 m (width at the bottom 0.32 m) with a depth of 0.35 m. An oval-shaped spot of small charcoal pieces (15x24 cm) was detected inside the burial pit infill of black soil with clay inclusions. The grave pit had shoulders, possibly used for construction of wooden covering, which remains were recorded in the profile of the burial pit. Deeper in the infill, fragments of a human mandibula (in the southwest corner) and traces of almost completely decayed skull (in the central part) were unearthed.

On the bottom of the grave pit, postcranial bones of a skeleton of an adult aged 25-30 years was excavated, positioned supine. It is possible that the skull, previously cleared higher, belongs to this individual. There are no signs of trauma on the cervical vertebrae. Below the skeleton (in its upper part), remnants of organic bedding were found.

In the infill, remains of a collapsed thick-walled clay vessel of reddish-brown color with dreg impurities in the clay, placed along the burial axis, were cleared. On the ledge on the eastern side, a fragment of a red clay vessel with coarse sand impurities and a fragment of black ceramic with dreg impurities were identified.

At the level of the cervical vertebrae, a bronze pendant in the form of a bear with well-defined protruding ears was found. In the area of the cervical vertebrae, a fragment of a ring wire temple pendant with a wound faceted ribbon was positioned. Under the elbow joint of the left arm, a heavily corroded iron buckle with an oval frame and previously movable tongue was identified. Decayed remnants of an iron knife were preserved on the pelvic bones. Above it, parts and decayed remnants of iron bits were discovered.

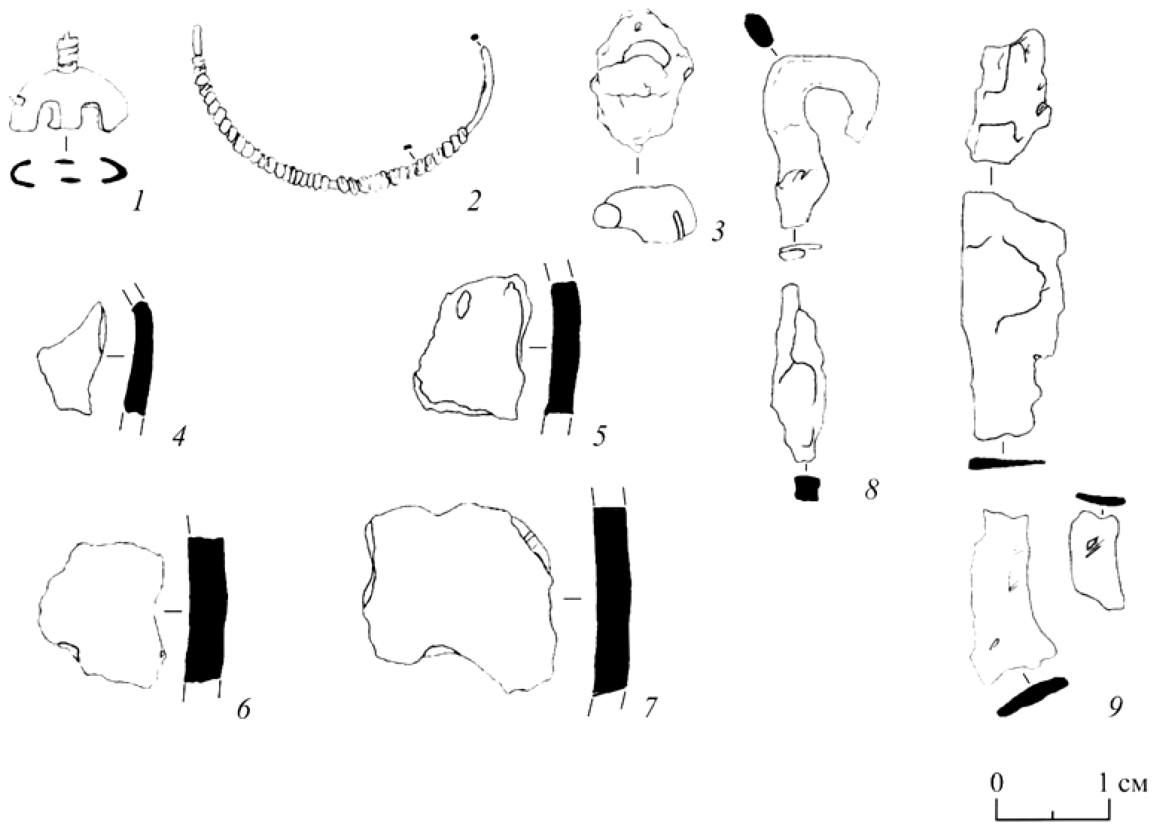


***Figure SIII.8.*** *Grave goods from burial 694 at Birsk-2.*

## ***burial 702 (individual ID I19265_d, Male)***

An oval burial pit 2.1x0.63 m oriented along the north-north-west - south-south-east line was recorded at a depth of -0.80 m. The depth of the grave pit was The filling contained scattered calcified bones and 2 teeth (fangs), from which it is possible to determine the approximate age of the buried person as 35-45 years old.

A collapse of a handmade pottery vessel and an iron buckle with a round-shaped frame and a movable tongue were found at the bottom of the grave pit.

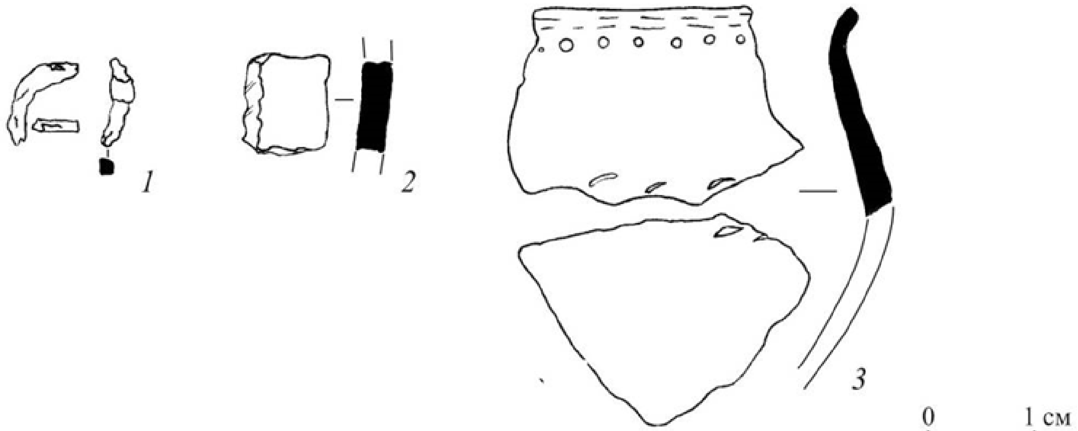


***Figure SIII.9.*** *Grave goods from burial 702 at Birsk-2.*

### *burial 710 (individual ID I19266_d, Male)*

The grave pit oval in shape oriented along the north-north-west - south-south-east line was discovered at a depth of -0.59 m. Its dimensions were 2.44 x 0.90 m, and depth 0.32 m. The grave pit was covered by wooden boards, placed in ledges in the northern, eastern and southern walls.

At the bottom of the grave pit, a skeleton of a 30-35 year old male was found, stretched out on his back and with his arms located along the body. In the area of the ribs, remains of a leather belt and a bronze belt-tip were found made from a bent plate, with an extension in the bent part. A bead of green glass was also found here.

At the knees of the skeleton, a cluster of objects was discovered, including an axe, an iron spearhead, and a belt tip. In the southern part of the burial, near the bones of the right foot, a ritual assemblage was discovered, consisting of a bracelet, inside of which were placed 4 Mazunino-type pendants (2 of them had embedded beads of white and blue glass). A bronze temple wire ring wrapped in a bronze ribbon and an iron tetrahedral awl were also cleared here.

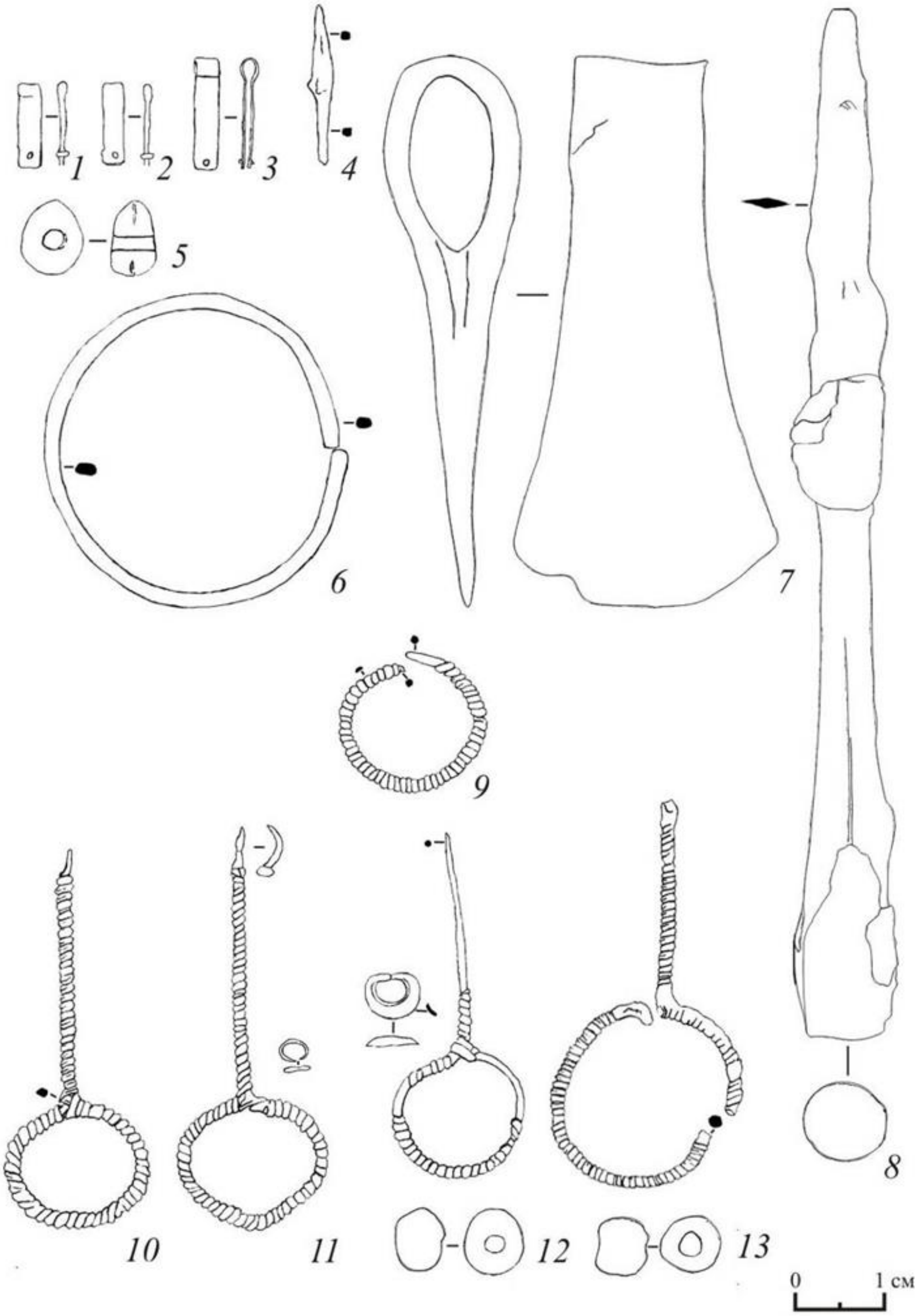


***Figure SIII.10.*** *Grave goods from burial 710 at Birsk-2.*

## *burial 713 (individual ID I19267, Female)*

The oval-shaped burial pit measuring 1.4x0.62 meters was discovered at a depth of -0.41 m and had a depth of 0.22 meters. The burial pit had a flat bottom and a ledge in the southeastern

wall. The orientation of the burial was northwest-southeast. The skeleton was placed within a wooden structure, secured by decayed material. From the skeletal remains, a crushed skull turned to the left and a shoulder bone were preserved. The age of the individual, approximately 4 years old, was determined based on the discovered bones (teeth).

The skull was likely covered with a cloth with beadwork, which fell over a ritual assemblage placed near the head of the deceased person. The assemblage included a ring of Mazunino type, a twisted temple ring, rhombic-section torque, beads, chains (small and large), round temple pendant, and a triple-shaped appliqué. A belt buckle was discovered in the central part of the burial, with two additional buckles found in the southern part of the burial.

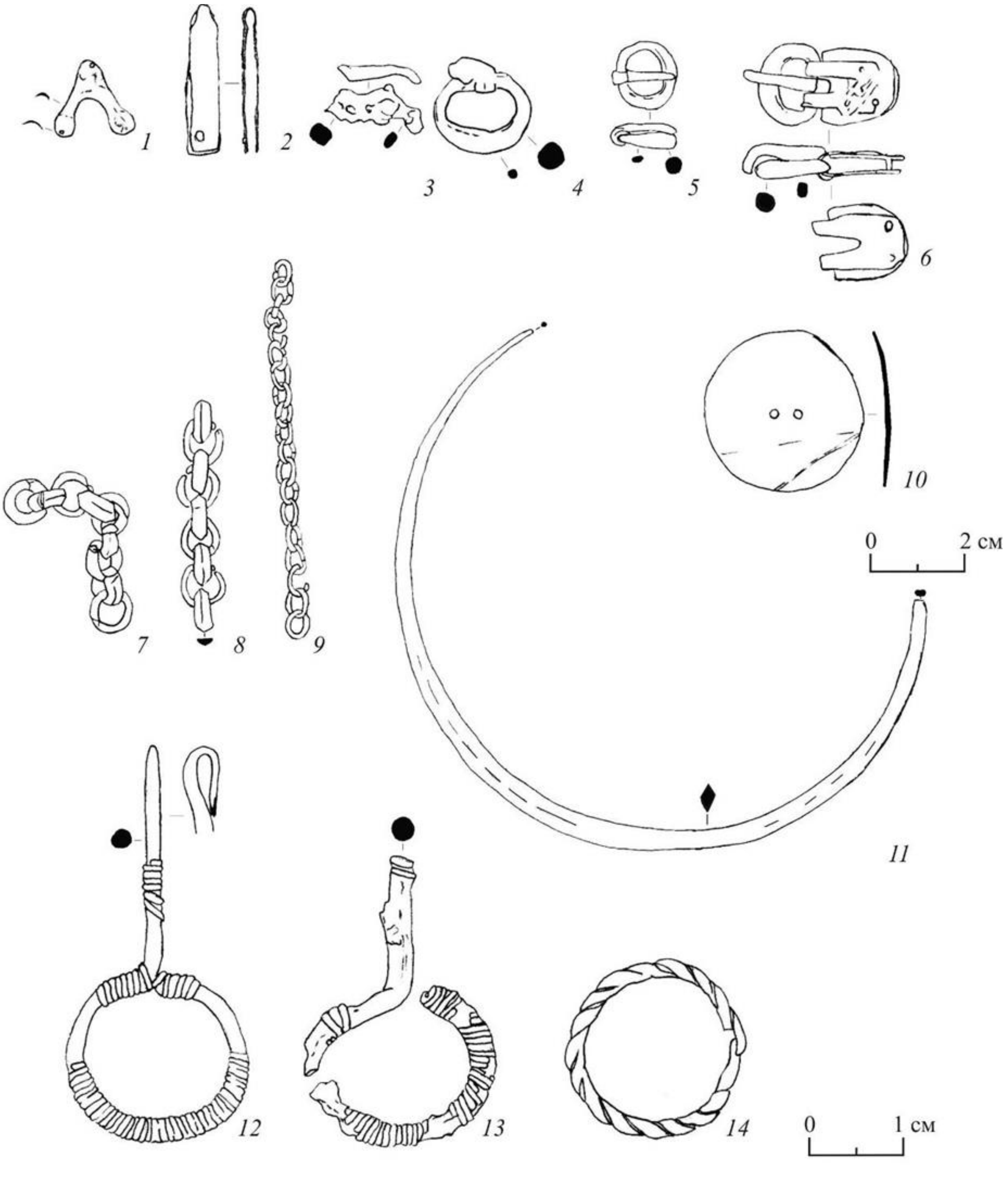


***Figure SIII.11.*** *Grave goods from burial 713 at Birsk-2.*

## III.C. The Kushnarenkovo culture (Belaya_Kushnarenkovo)

The terms Kushnarenkovo and Karayakupovo cultures refer to cultural traditions of the Cis-Ural population dated to the 6th-9th century CE and distinguished mainly on the features of handmade pottery making. However, these definitions have been actively discussed for more than half a century. Some researchers consider burials and settlements with the Kushnarenkovo-type pottery as an archaeological type (Khalikova, 1976) or clearly defined archaeological culture of the 6th-10th centuries (Vasyutkin, 1968; 1992; Kazakov 1981; 2001). Other researchers believe that this is the early chronological stage of the Karayakupovo culture (Mazhitov 1977; 1981a; 1981b), united Kushnarenkovo-Karayakupovo culture (Botalov, 2019), two independent synchronous cultural groups (Matveeva G., 2007; Ivanov A., 2008) or two asynchronous cultures of the late 6th – early 8th and mid 8th - 9th centuries (Ivanov V., 1999b; 2009). The most radical version supposes the origin of handmade Kushnarenkovo-type ware as a product of “traveling potters” (Matveeva N., 2021). This discussion is too far from the ending, therefore we have to use the term "horizon", denoting the time of the highest spread of this cultural phenomenon.

**Kushnarenkovo horizon**. The distinctive features of Kushnarenkovo pottery were characterized only by the results of the excavations of an ensemble of sites discovered near the village of Kushnarenkovo in the second half of the 1950s, despite some previous discoveries (Gening, 1977). Interestingly, Kushnarenkovo pottery was not predominant or even notably represented on any of these sites, including burial grounds, settlements, and hillforts. Despite the presence of Kushnarenkovo ceramics, systematic characterization of the culture is lacking, and its chronology remains undeveloped. Existing concepts suggest a prehistory of ceramic tradition from the Trans-Urals region, although the date of the culture's origin remains uncertain.

There is evidence of the use of semi-subterranean dwellings at the Kushnarenkovo settlement, while others were likely light surface structures. Ceramic vessels are characteristic, with medium and tall vessels featuring high vertical necks decorated with fine diagonal notches around the rim, horizonal carved lines along the neck and upper body, rows of inclined impressions from toothed stamps, and fine diagonal mesh patterns. Kurgans at burial sites range from 6 to 10 meters in diameter and up to 0.5 meters in height, with 1-3 (rarely up to 5) burials in rectangular or oval pits 40-60 cm deep from the burial soil level. The bodies are placed supine, with the head facing north or west, occasionally ritually disarranged. Among the artifacts are ornaments, horse equipment, weapons; vessels are usually placed at the head, with food remains (horse or sheep bones) nearby. Skulls and limb bones of horses are found under mounds or in graves, possibly with the animal's skin. Other findings include an iron sword in wooden scabbards, arrowheads, fishhooks, bridle rings, stirrups in later complexes; bone inlays for bows; a silver chain necklace (Taktalachuk burial ground); silver and bronze belt fittings, earrings, pendants, rings; beads, including cornelian with white inlay.

The earliest datable artifacts found together with the Kushnarenkovo-type pottery are belt sets and various strap ornaments of “heraldic” styles (late 6th – early 8th centuries). Later the characteristic features of the Kushnarenkovo pottery traditions are observed in complexes with the Karayakupovo-type ware (Matveeva G., 2007; Ivanov V., 2009). However, the Karayakupovo-type tradition dominates most of these sites with the exception of the Bolshiye Tigany cemetery (9th – early 10th century).

**Genetic results.** *In this group, we analyzed one individual from the site of Bustanaevo, with sufficient coverage for downstream population genetic modeling (>100,000 SNPs), and for IBD-sharing analysis (>600,000 SNPs). This male showed similarities to Late Iron Age Tobol region individuals in genetic composition. The supervised ADMIXTURE analysis revealed an elevated level of Samara_EBA_Yamnaya and Yakutia_LNBA ancestries. The Belaya_Kushnarenkovo sample clustered within the Urals-Carpathian EMA cluster. These genetic results present the first appearance of the Trans-Uralian ancestry in the Belaya river region, dating this gene flow to the period between the 3rd-4th and 6th-7th centuries CE.*

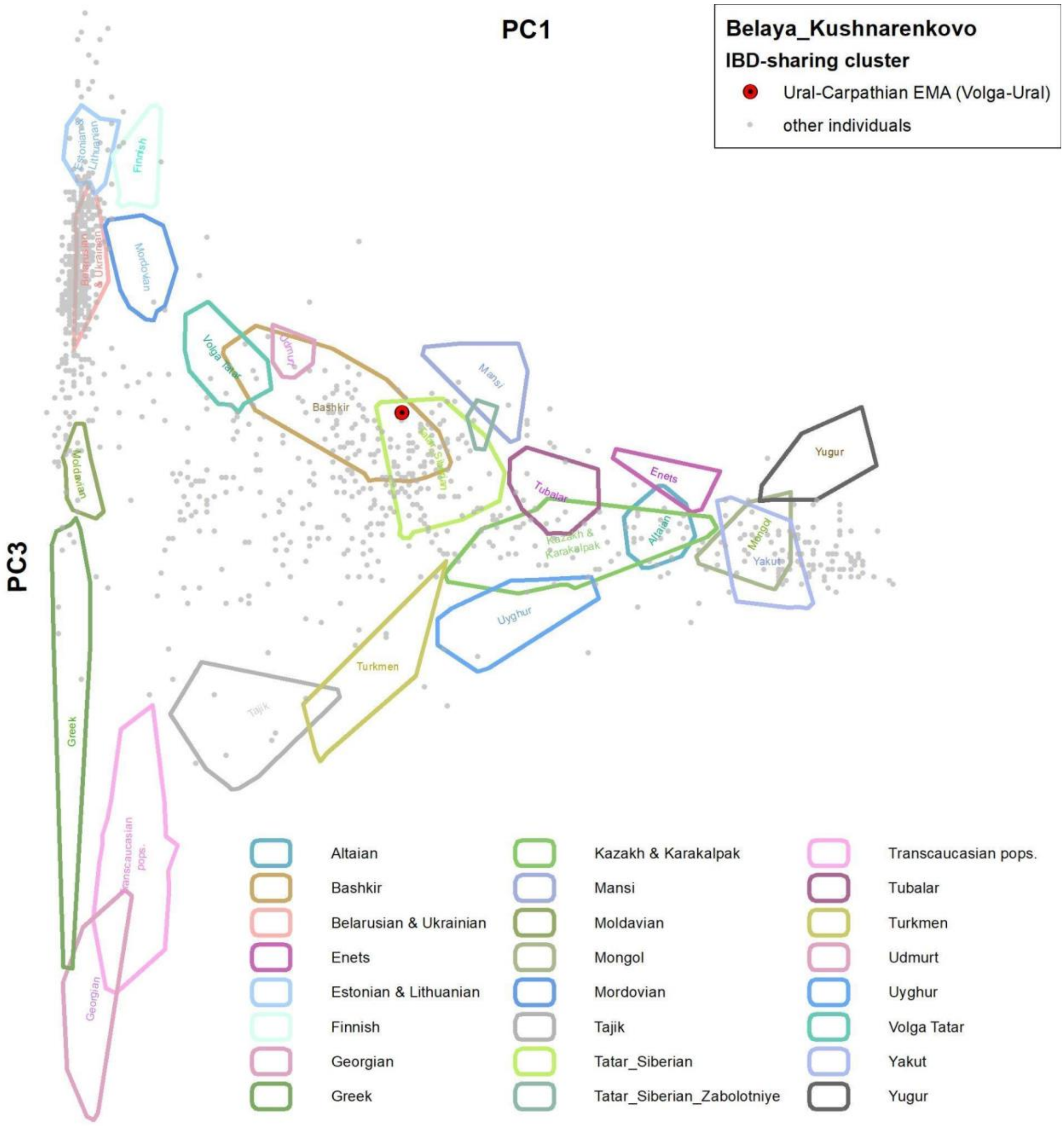


***Figure SIII.12.*** *Eurasian-scale principal component analysis (PCA), with a projection of the newly sequenced individuals onto modern genetic variation following Jeong et al. 2019. The PC1 and PC3 dimensions are shown, including the newly presented genomes. Polygons represent example populations from modern Eurasian individuals onto which the ancient samples were projected, while grey dots indicate ancient reference samples from our IBD-sharing network.*

**Karayakupovo Horizon.** The eponymous hillfort of the Karayakupovo culture has been studied since the early 1960s (Matveeva G., 1975; Ivanov V., 1990; 1999b).

Hillforts are located on elevated promontories (some without traces of earthworks; some are shelters), while settlements are on river terraces with a sparse cultural layer. Characteristic pottery is round-bodied, with ovoid, spherical, or pear-shaped bodies and straight, slightly curved necks. The ornamentation consists of diagonal impressions from short smooth or toothed stamps on the rims, horizonal lines on the neck and shoulder, waist belts of pits and protruding "pearls." Karayakupovo-type ceramics are found in subterranean(?) and sub-barrows burials at sites with a broader chronological range (Manyak, Karanaevo, Beketevo, Idelbaevo). The associated context contains spindle whorls of truncated conical shape, crucibles, spoon-molds, fishhooks. The economy was likely based on livestock and farming (charred grains of spelt, millstone fragments were found).

The earliest Karayakupovo-type ware was found in complexes with "heraldic" style ornaments of the late 7 – early 8th centuries CE. Starting from the mid 8th century, the prestigious fashion of the population of the region (belt ornaments, harnesses, weapons etc.) changes under the impulse of the early Saltiv culture, on the other hand - under the influence of the Turkic nomads of Central Asia. In the middle of the 9th century they were both replaced by new nomadic impulses of influence, among which the Proto-Magyar and Srostki culture, and later in 10th century CE also Oghuz-Patzinaks culture.

## III.C.1. Bustanaevo kurgan burial site

The Bustanaevo burial kurgans were discovered in 2011, with initial investigations conducted by Aleksandr Kolonskikh in 2015 (Kolonskikh et al., 2024). During reconnaissance work, 43 mounds were identified, and the presence of two concentration zones of mounds (NW and SE) was established. The necropolis is located on the edge of the main terrace on the watershed of the Bystry Tanyp and Gareika rivers. We sequenced one individual found in burial 1 under mound 45.

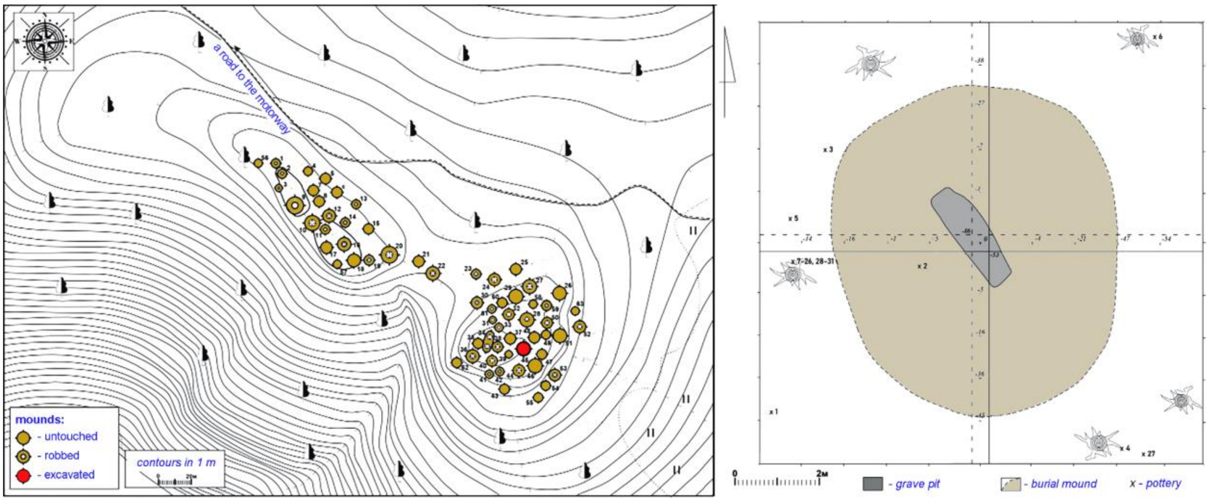


***Figure SIII.13.*** *Bustanaevo kurgans. A plan of the kurgan group and a plan of the excavated kurgan 45.*

***mound 45, burial 1 (individual ID I25531, Male)***

Burial 1 under mound 45 had a sub-rectangular shape with rounded corners. The length of the burial pit is 235 cm, the width in the southeast part is 57 cm, in the northwest part is 50 cm, and at its widest point is 64 cm. The depth of the burial pit varies from 79 to 91 cm from the ground level. The bottom of the pit had a slight depression in the center. The walls are vertical, without additional steps, ledges, or shoulders, although a small recess was made under vessel 1 in the western wall. The mound is oriented along the northwest – southeast line, with the deceased placed head to the northwest, which is typical for the Kushnarenkovo burial rite. The skeleton was poorly preserved, only fragments of the skull, shoulder, and femur bones were discovered.

In the burial, flower-shaped four-petal overlays, a buckle fragment, a belt tip, two vessels, five arrowheads, elements of horse equipment, knife fragments, and a pendant were found. The Kushnarenkovo-type vessels were located in the northwest part of the burial: vessel 1 stood west of the skull in the niche at the western wall, vessel 2 laid on its side and was crushed. Fragments of animal bones, likely from sheep, were found in vessel 2, indicating food offerings.

The determined radiocarbon age of the burial is 340-540 cal CE (1640±25 BP, DeA-16570) (based on human bone), however, the grave goods confirm its irrelevance. Based on archaeological data, the grave must be dated to 560-630 CE. Pottery confirms that the burial is associated with the Kushnarenkovo archaeological culture.

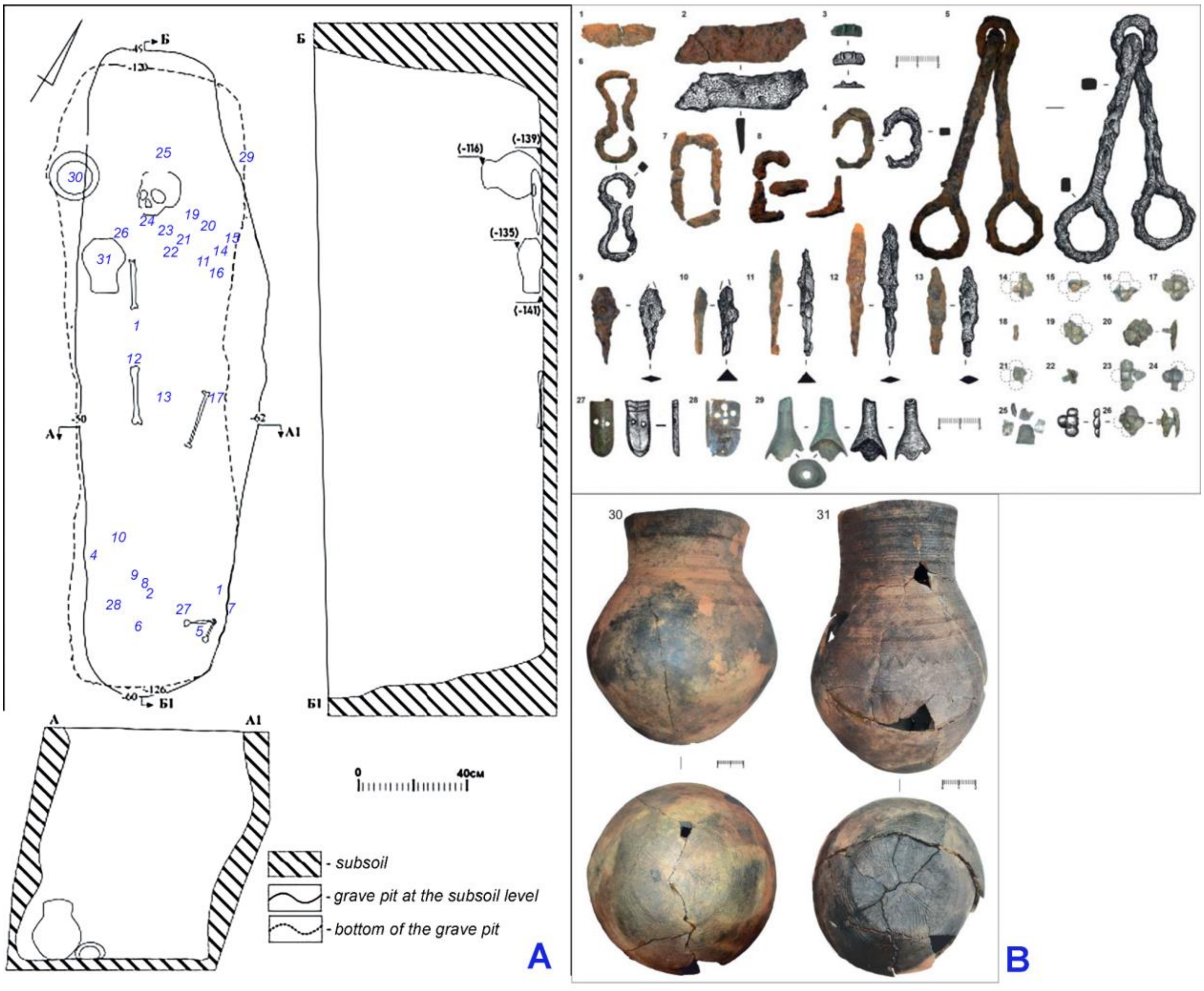


***Figure SIII.14.*** *Bustanaevo, mound 45, burial 1. A - a plan of the burial, B - grave goods.*

## III.D. The Nevolino culture in the Cis-Urals (Cis-Urals_Nevolino)

The main features of the Nevolino culture were described in the section II.E.

**Genetic results.** *In this group, we analyzed one individual from the site of Bartym, who had sufficient coverage for downstream population genetic modeling (>100,000 SNPs) and IBD-sharing analysis (>600,000 SNPs). This male displayed possible genetic continuity from the Iron Age, with a similar genetic composition. The supervised ADMIXTURE analysis revealed elevated levels of Samara_EBA_Yamnaya and Yakutia_LNBA ancestries, suggesting continuity from Late Iron Age times. This individual clustered within the Urals-Carpathian EMA cluster.*

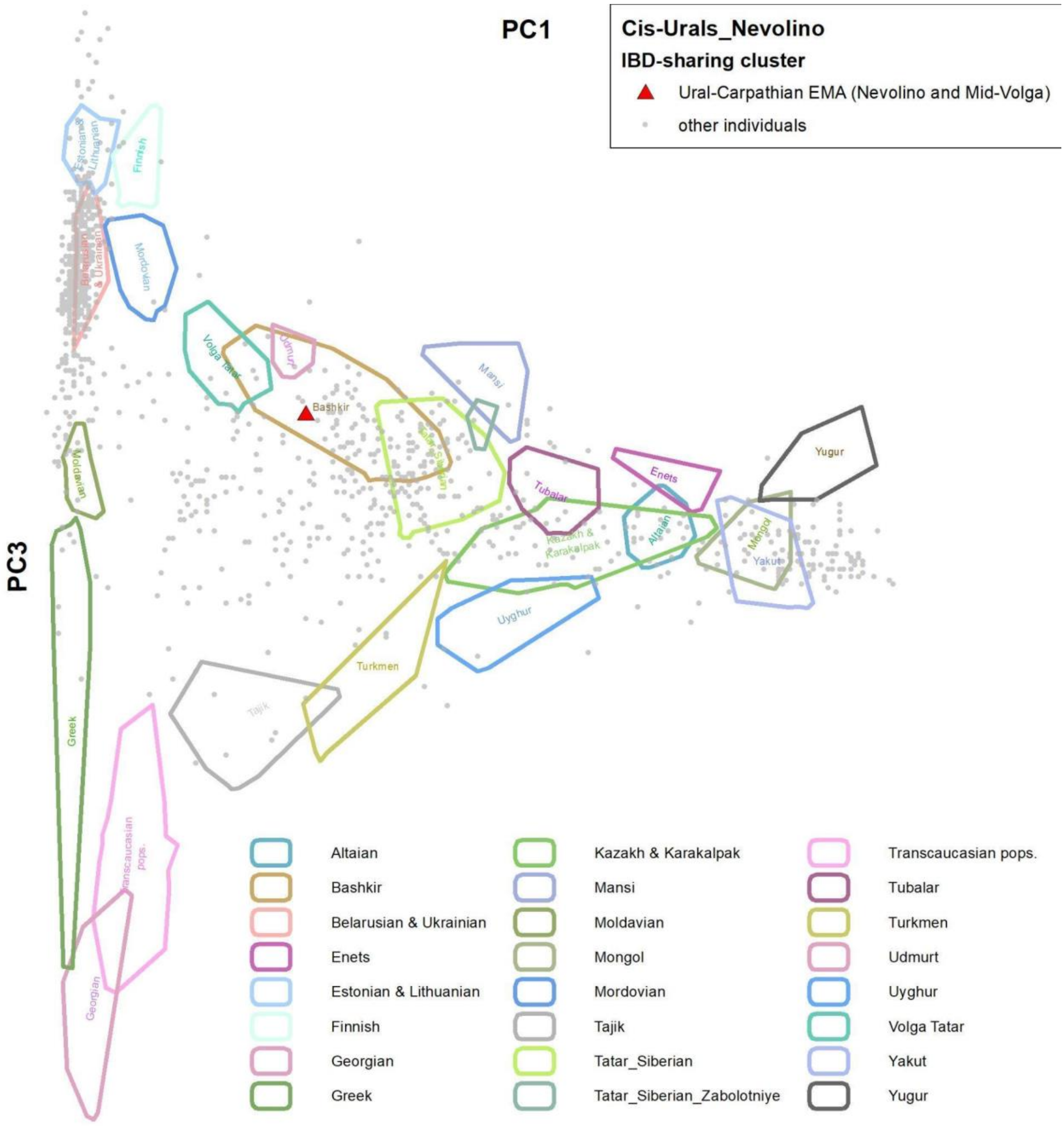


***Figure SIII.15.*** *Eurasian-scale principal component analysis (PCA), with a projection of the newly sequenced individuals onto modern genetic variation following Jeong et al. 2019. The PC1 and PC3 dimensions are shown, including the newly presented genomes. Polygons represent example populations from modern Eurasian individuals onto which the ancient samples were projected, while grey dots indicate ancient reference samples from our IBD-sharing network.*

## III.D.1. Bartym burial ground

The Bartym-1 burial ground occupies the southwest part of Bartym-1 settlement of the Nevolino culture. In 1980, 1981, and 1983, during excavations conducted by the archaeological expedition of the Udmurt State University, an area of about 2900 square meters was excavated, revealing 19 burials containing 60 skeletons.

The upper part of the cultural layer in this area was completely destroyed by plowing. The burial pits were identified after the removal of the plow layer at a depth of 20-25 cm. The bedrock at the site is limestone with clay lenses. Some burial pits were carved into solid rock (8 graves - 42.1%), while others were dug into clay (11 graves - 57.9%). One of the burials was made within a cash pit of the settlement. Some graves were disturbed by plowing.

### *burial 16, individual B (individual ID I19110, Male)*

The burial level is -65 cm. The grave has square outlines with rounded corners, dimensions 183x158x80 cm, oriented along the east-south-east - west-north-west line. At the level of -65-80 cm, nine skeletons were found, oriented with their heads to the ESE. Four skeletons (A, B, C, G) were arranged in a row; four belonging to children (D, E, F, H) were found at the feet of skeletons A, B, and C; the fifth (I), from which only a fragment between the thigh bones was preserved, had 3 bronze plates, a bronze buckle on the right shin, 3 plates, and a ornament consisting of bird-shaped, swollen, and horn-shaped ornaments, 10 glass beads. At the northern end, between the skulls B and F, a set of 9 bronze tubular ornaments and a bronze rattling pendant were found. In the area of the belt of skeleton C, next to the tubular arm bones, 4 glass beads, 2 bronze wheel-shaped pendants, and 3 plates were found. To the left of these tubular bones, 6 bronze plates, a buckle, a belt tip, and a fragment of an iron knife were found. Between the fragments of skeletons C, F, and E, fragments of an iron knife and a bronze buckle were found. Slightly below, at the head of skeleton E, bronze plate fragments and a buckle were found to the left of the legs of skeleton F.

Anthropologically determined sex and age-at death of the buried individuals is as follows: A - male, 18-20 years old; B - male, 35-45 years old; C - male, 30-40 years old; D - sex?, 5 years old; E - gender?, 4 years old; F - gender?, 5 years old; G - gender?, 6 years old; H - gender?, 0.5 years old.

The determined radiocarbon age of the burial is 428-591 cal CE (1535±24, DeA16585) (based on human bone from individual D).

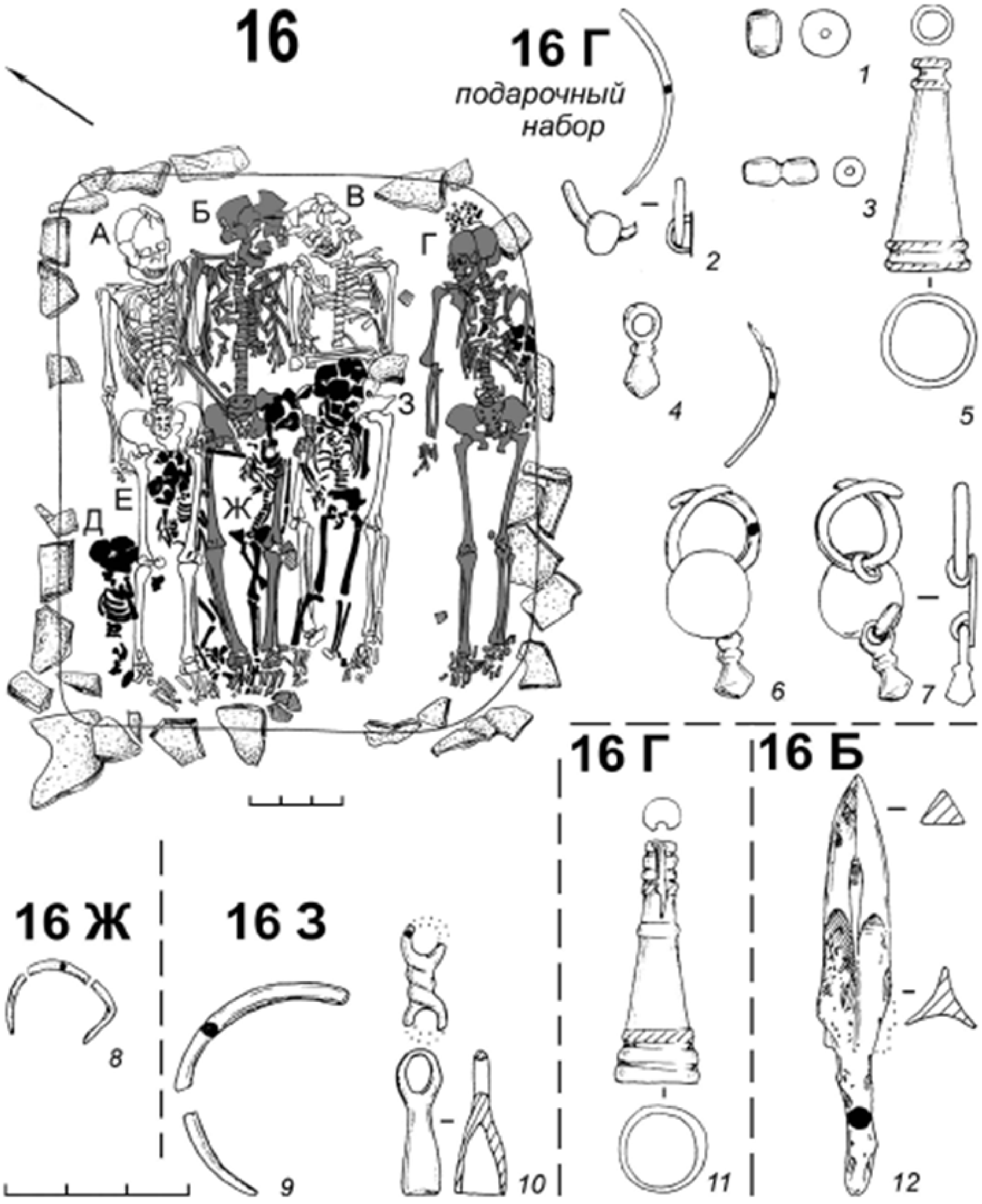


*Figure SIII.16.* Burial 16 at Bartym.

## III.E. The Lomovatovo culture (MidKama_Lomovatovo)

The interest in the "cult" finds of the Lomovatovo type emerged in the mid-19th century (Spitsyn, 1893). Systematic excavations primarily targeted burial complexes and were generally characterized by Rimma Goldina in her dissertation (Goldina, 1970; 1971; 1985; Goldina, Kananin, 1989). Subsequent work became sporadic, only intensifying in recent years.

Synthesizing a broader chronological range led A.M. Belavin to establish a new periodization (Belavin, Krylasova, 2016).

Settlement clusters encompassed hillforts, settlements, and burial grounds. The sites attributed to the Lomovatovo culture also include several small late *kostisches* (from Russian *kost'* "bone", a specific type of ritual sites of the Kma Iron Age with layers of burned fragmented animal bones). Houses had sunken floors, featuring hearths and storage pits, pillar constructions, and adobe floors, with some displaying sidewall trenches and earthbank reinforcements. Pottery was handmade (often shell-tempered), low-profiled, with rounded or flattened bottoms, decorated with comb, cord, and other stamps, lines, forming bands of simple compositions on the upper vessel part, and incisions (including stamping) on the rim.

Burial grounds until the 7th century comprised mounds (sometimes with up to 12 burials) and adjacent (including synchronous) ground burials (with rows discernible), typically featuring supine body placements. Wooden structures and charcoal finds were common; animal bones and pottery appeared in fills (mostly in cremation burials). Burial items included attire details, usually worn, ceramic vessels, occasionally weapons, horse gear, and household items; silver masks near the eyes, silk face covers are known. Attire featured temple pendants, necklaces (beads, coins, etc.), bracelets, rings, belts, often of local Ural types, diverse pendants (numerous jingling ones) and awls. Hoards were numerous, including Iranian and Byzantine metalware, coins, jewelry; a special group featured Permian animal-style artifacts. Economy was based on slash-and-burn agriculture, animal husbandry, with hunting and other crafts being important.

Several local groups and 4 phases are distinguished: Kharino (6th century), Agafonovo (late 6th - 7th centuries) (Goldina, 1980), Demenki (late 7th - 8th centuries), Uryino (late 8th - 9th centuries), Lavryata (10th - late 11th centuries). The formation of the Lomovatovo tradition is associated with the colonization of the taiga and subtaiga of the Kama area by the Kharino-Glyadenovo population. The Medieval Rodanovo culture derived from the Lomovatovo tradition.

**Genetic results.** *In this group, we analyzed one individual from the site of Bayanovo. This sample had sufficient coverage for downstream population genetic modeling (>100,000 SNPs) and was suitable for IBD-sharing analysis (>600,000 SNPs). This male exhibited a genetic composition similar to the Low Kama groups but with elevated steppe ancestry. The supervised ADMIXTURE analysis revealed high levels of Samara_EBA_Yamnaya and Yakutia_LNBA ancestries and clustered within the Urals-Carpathian EMA cluster.*

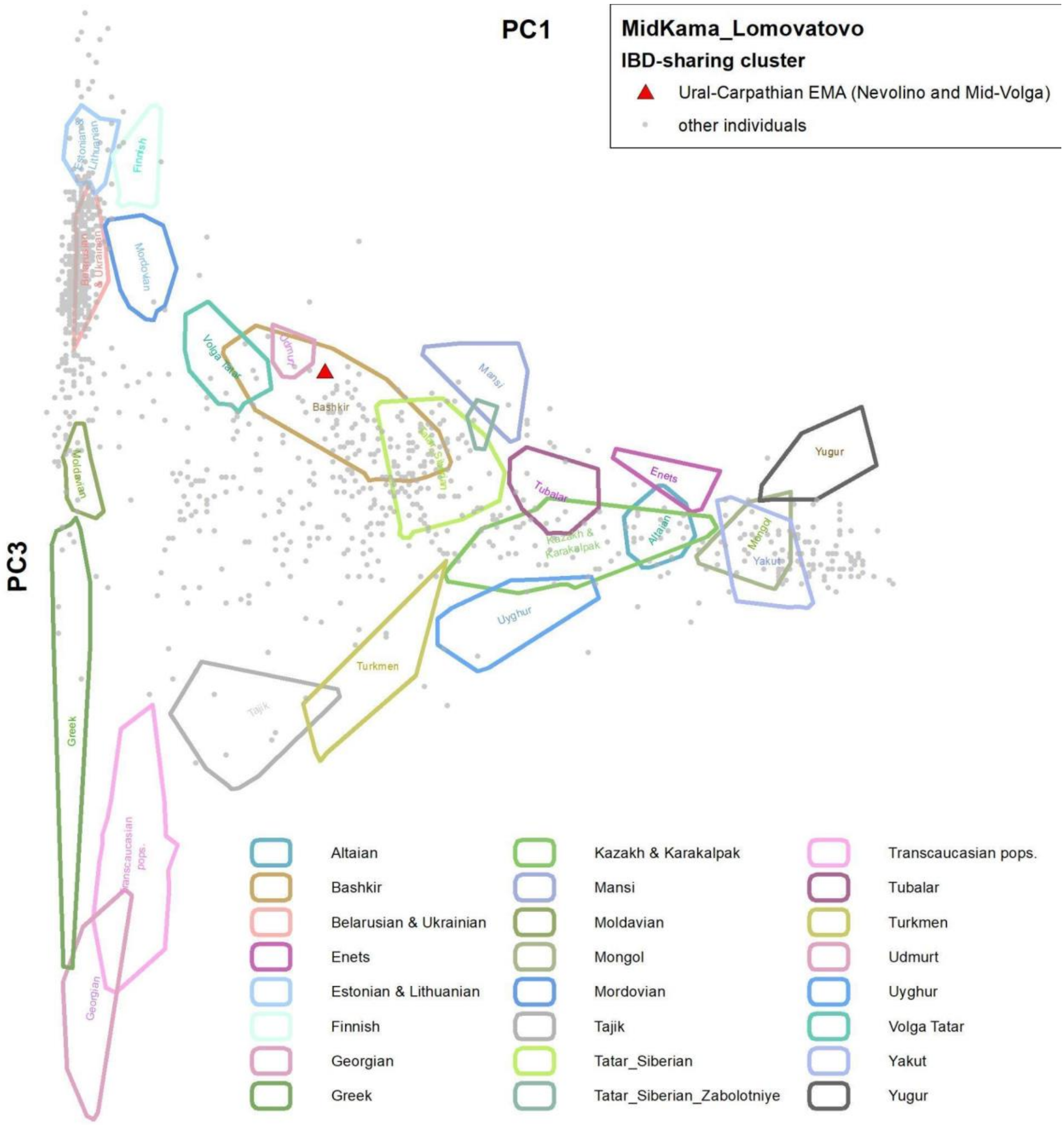


***Figure SIII.17.*** *Eurasian-scale principal component analysis (PCA), with a projection of the newly sequenced individuals onto modern genetic variation following Jeong et al. 2019. The PC1 and PC3 dimensions are shown, including the newly presented genomes. Polygons represent example populations from modern Eurasian individuals onto which the ancient samples were projected, while grey dots indicate ancient reference samples from our IBD-sharing network.*

## III.E.1. Bayanovo burial ground

The site was discovered in the spring of 1951 during quarrying activities for the construction of the Levshino-Kizel railway, which partially destroyed the site. Soldiers from the military unit constructing the road collected some items and reported the findings to the Molotov (Perm) Regional Local Lore Museum, and later to the Molotov State University.

On July 18, 1951, V.A. Oborin and Z.P. Sokolova visited the site of the finds to survey and collect items. An excavation was initiated at the edge of the quarry in its southeast part. In 1951 and 1953, excavations were conducted at the burial ground by V.A. Oborin. In August 2005, excavations at the burial ground were commenced by A.V. Danich. By 2012, a total of 283 burials had been studied across an area of 1472 m2.

### *burial 277 (individual ID I19113, Male)*

The burial is oriented along the northeast-southwest axis and was identified at a depth of -0.84 to -1.10 meters from the arbitrary excavation datum. Its dimensions are 1.91 x 0.73 meters, with a maximum depth of -1.29 meters from the excavation datum. The bottom is flat, and the walls are vertical.

The burial contains the remains of a single individual arranged anatomically. In the northern part of the burial, a poorly preserved skull and mandibula are found. The skull lies with its facial aspect upward, while the mandibula stands on its base, oriented vestibularly to the south. The remains include the temporal bones, diaphyses of both forearms, metacarpal bones, and phalanges, as well as fragments of the pelvis and both femurs. The pelvis remains unopened. The long bones are aligned parallel to each other and to the burial's arbitrary axis.

The skeletal remains in the burial belong to a male aged 18-25 years. Based on the position of the bones, the deceased was placed on his back with his head to the northeast.

The burial yielded rich inventory:

1. Two solid bronze beads with a slight widening in the middle and an unornamented edge.
2. Fragments of a silver mask.
3. Fragments of a silver mask.
4. Silver wire temple ring of oval shape with a slight thickening at one end of the wire.
5. Silver pendant depicting a horse rider. The pendant hung around the neck using a bronze spiral-wound pin.
6. Fragment of a solid bronze bead with a slight widening in the middle and an unornamented edge.
7. Flint.
8. Pouch made from the skin of a large animal. Trimmed along the edge with small bronze ferrules. Suspended from the belt using a bronze spiral-wound pin and 6 solid bronze beads with a slight widening in the middle and an unornamented edge.
9. Round wire bronze bracelet, hexagonal in section. Ornamented on the outer side with three rows of circular ornamentation. Located on the left arm.
10. Round wire bronze bracelet, hexagonal in section. Ornamented on the outer side with three rows of circular ornamentation. Located on the left arm.
11. Bronze tube-shaped pipe tamper.
12. Fragments of a leather belt with bronze overlays. The front part of the belt is decorated with small oval overlays adorned with two groups of horizonal lines, with 3 lines per group. The side and rear parts of the belt are decorated with flat figurative overlays with protrusions on the lateral sides.

13. Flint with a bronze handle. The handle is carved with an image of two horse riders riding in different directions. Along the base, the flint is decorated with an ornamental band resembling a wavy line.
14. Solid bronze bead with a slight widening in the middle and an unornamented edge.
15. Knife with a wooden handle.
16. Flat figurative bronze overlay with protrusions on the lateral sides.
17. Flat figurative bronze overlay with protrusions on the lateral sides.
18. Laurel-leaf arrowhead without a socket.
19. Two small solid bronze beads with a slight widening in the middle and an unornamented edge.
20. Bronze openwork buckle depicting a wolf's head, with a rectangular ring and an oval receiver. The buckle is decorated with notches along the edges and the front edge of the ring.
21. 28-31,33. Four arrowheads without sockets.
22. Bronze shield-shaped buckle with an oval ring and a sharpened unornamented rear plate.
23. Coin die with a narrow, elongated, triangular blade and a hammer-like butt. The cross-section of the butt is rectangular.
24. Saber. The length of the preserved portion is 70.3 cm, the length of the preserved double-edged portion is 11.8 cm, the length of the handle is 10 cm, the width of the blade at the hilt is 3.6 cm, the width at the double-edged portion is 2.6 cm, the width of the handle is 2.2 cm, the curvature of the blade is 0.03 cm, the thickness of the handle is 4 mm, and the thickness of the blade is 5 mm. The handle has wooden spherical grips and a slight tilt toward the blade. The saber was housed in wooden scabbards, traces of which are visible along the entire length of the blade. The saber has a block-shaped crossguard with spherical ends, made of two parts. At a distance of 17 cm from the crossguard, there is an iron suspension consisting of two parts, each 1 mm thick, riveted together with two rivets.

The burial is dated to 770-875 cal CE (1213±22, DeA11172).

## III.F. The Polom culture (MidKama_Polom)

The first excavations on the sites later attributed to the Polom culture, were conducted in the late 19th century, and their materials remain unpublished. A brief description of the cultural assemblage was provided in the late 1950s, while the key site, the Varni burial ground, has been under study since 1970.

The sites along riverbanks consist of 1-2 fortified settlements, burial grounds, and open settlements. Fortified settlements on promontories with steep slopes feature ramparts and ditches, sometimes in two lines. The houses are rectangular, timber-framed, with areas ranging from 24 to 85 square meters, with clay or sandy-gravel floors and hearths on clay bases with stones, with 1-2 hearths along the walls; a gabled roof is reconstructed. Within or near the dwellings are 1-3 pits lined with boards or bark. Among the outbuildings, timber stables are prominent. Traces of metal and bone processing have been found. The pottery (round-bottomed bowls) is handmade, with shell admixture in the clay; predominant

ornamentation includes comb-stamped and geometric patterns, filled with incised lattice stamps, cord impressions.

Burial grounds are large and flat; graves are arranged in rows. In the early part of the burial ground near the village of Varni, there are mounds with circular trenches. The bodies are laid on their backs, in coffins or wrapped in bast or birch bark, often oriented with their heads to the west (early graves) or north (later ones). Clay or metal vessels were placed in the graves, sometimes with antler spoons; pieces of meat were placed at the feet; at a later stage, burial rituals involved the inclusion of horse parts (skull or jaw, leg bones). Male burials contain earrings, bracelets, rings, belts sets, axes, spears, swords, sabers, bows with arrows, chisels, planes, knives, and horse harness items; female burials—earrings, temple and chest pendants, necklaces of beads, bracelets, rings, belts with appliqués and pendants, knives with elaborately decorated sheaths, awls, whorls, spindles. Various rattling pendants, including zoomorphic ones, are common. Southern trade (?) connections of the Polom population are confirmed by finds of "eastern silver" hoards (coins, vessels from Iran and Central Asia). Subsistence activities include slash-and-burn agriculture and animal husbandry, with hunting, including fur trapping, and fishing being important.

The evolution of the Polom culture passed four phases: in the early (Gyrkeshur, 4th quarter of the 4th(?) – 5th-6th centuries; Varni, late 6th-7th centuries) the sites are concentrated in the upper reaches of the Cheptsa River, while in the late stages (Karavaless, late 7th-8th centuries; Mydlan'shay, late 8th-9th centuries) the middle course of the river with its tributaries was developed.

The cultural assemblage demonstrates influence from the Glyadenovo, Azelino, and (less significant) Mazunino traditions. The later evolution of the Polom tradition formed the Cheptsa culture, which spread over the same territory (Ivanov A., 1998).

**Genetic results.** *In this group, we analyzed two individuals from the site Varni. Both of them had sufficient coverage for downstream population genetic modeling (>100,000 SNPs), and for IBD-sharing analysis (>600,000 SNPs). No close relatedness was detected among these samples, and the female-to-male ratio was 1:1. The samples show similar genetic composition to the LowKama Iron Age groups. The supervised ADMIXTURE analysis revealed an elevated level of Samara_EBA_Yamnaya and Yakutia_LNBA/Turkey_N ancestries in this group. Despite differences in genetic composition with this cluster, these two samples were grouped in the Urals-Carpathian EMA cluster*

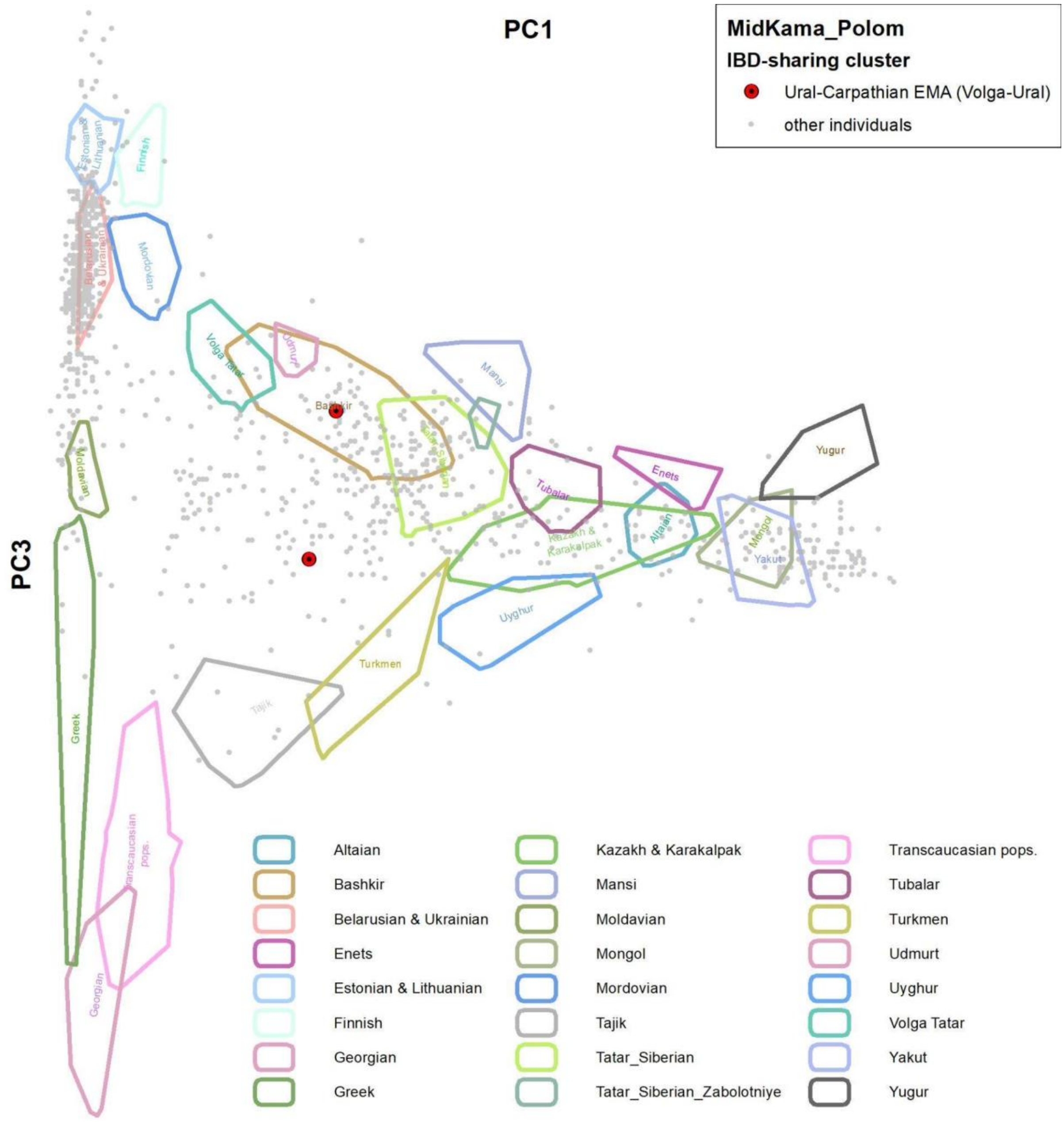


***Figure SIII.18.*** *Eurasian-scale principal component analysis (PCA), with a projection of the newly sequenced individuals onto modern genetic variation following Jeong et al. 2019. The PC1 and PC3 dimensions are shown, including the newly presented genomes. Polygons represent example populations from modern Eurasian individuals onto which the ancient samples were projected, while grey dots indicate ancient reference samples from our IBD-sharing network.*

## I.C.6.1. Varni burial ground

The Varni burial ground, dated from the 5th century to the first half of the 10th century, is located on the northern outskirts of the village of Varni and occupies a promontory-like part of the gentle southern slope of the high right bank of the river, transitioning into the main terrace of the Cheptsa River. Discovered by V.A. Semenov in 1970, the burial ground underwent systematic excavations in 1970-1973, 1984, and 1990 (Semenov, 1980, 1989). In 1991, Semenov's work was continued by N.I. Shutova (Shutova, 1999), and in subsequent years,

from 1994 to 1998, 2003, and 2006, archaeological research was led by Aleksander Ivanov (Gavritukhin, Ivanov, 1999). By the mid-2000s, an area of about 3303 square meters had been excavated, and 689 burials had been studied. From 2021 to 2023, excavations at the burial ground were conducted by Tatiana Sabirova.

Here we publish two individuals from the cemetery.

### *burial 158 (individual ID I33979, Male)*

The clear outlines of the burial pit, with a quadrangular shape and rounded corners, were observed at a depth of 40 cm. The grave, measuring 292 x 75 x 59 cm, was oriented along the south-north line. At all four corners of the grave, traces of stakes with a diameter of 5 cm were recorded, located 7-10 cm away from them. The profile of the northeast stake was square-shaped, but only for the first 15 cm from the end. The grave had an expanded northern end and very smooth, well-worked walls. At a depth of 100 cm, well-preserved skeletal remains were uncovered. The deceased was laid out on their back, with the head to the north, and the arms extended alongside the body. Traces of birch bedding were found at the bottom of the grave under the iron objects. To the right of the head, closer to the northwest corner of the grave, stood an iron cauldron assembled from several plates, with a massive iron handle. Beneath the cauldron lay an axe-dagger and a flint fragment. On both sides of the skull, near the temporal bones, lay temple pendants. Along the western wall, touching the right hand, lay a sabre. Only the bow holders of the scabbard were preserved. The handle retained traces of wood and the pommel. Near the left femur, lay a whetstone, and below the bones of the left hand, outside of the femur, a compact heap contained a gray-colored sharpening stone made of fine-grained sandstone, a seat in the form of a plate, and a tube for whetstone oil. At the feet of the deceased were the bones/ribs of a horse and fishing tackles (Semenov, 1980).

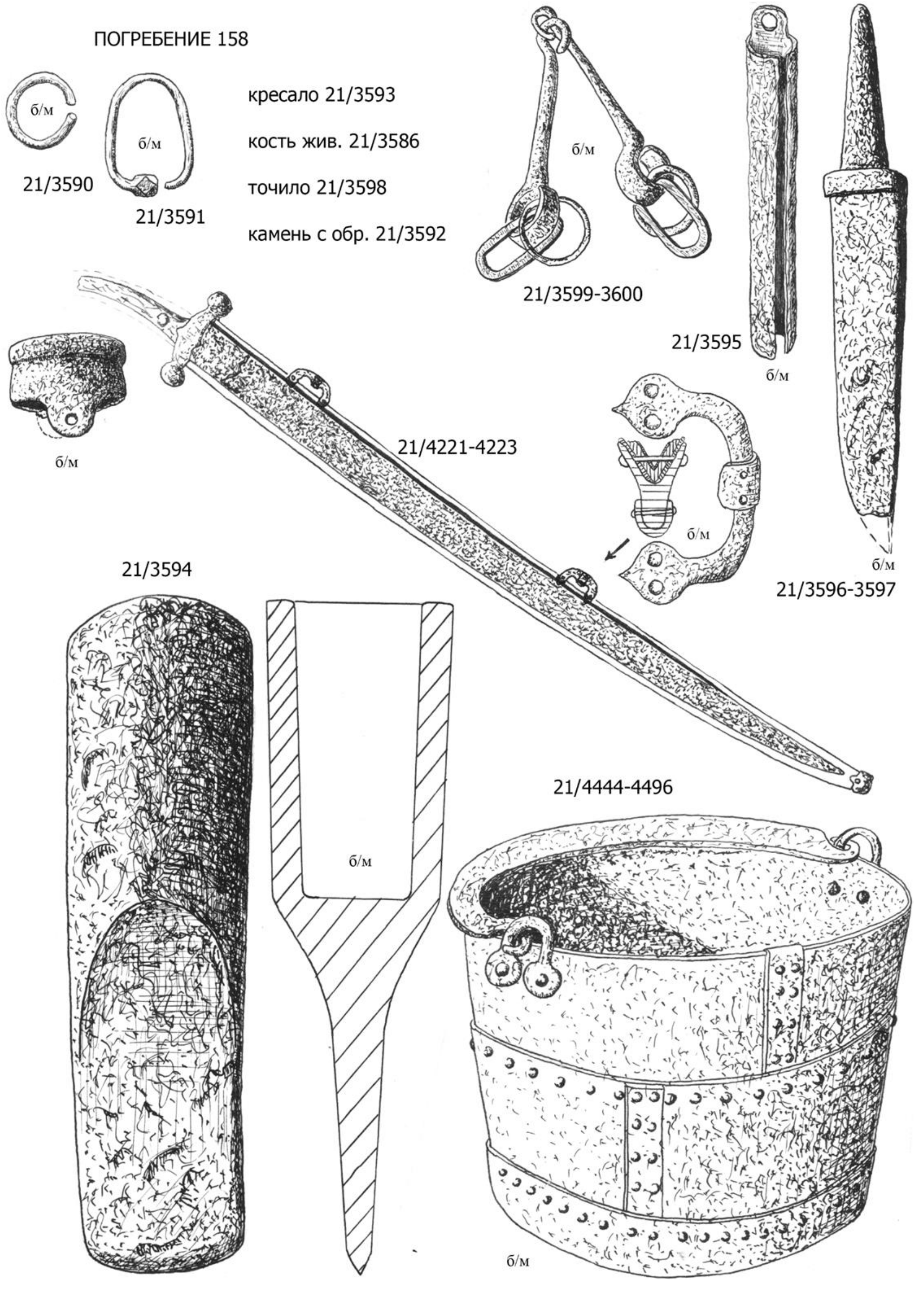


*Figure SIII.19.* Grave goods from burial 158 at Varni.

***burial 260 (individual ID I33978, Female)***

Fairly distinct outlines of a burial pit with a quadrangular shape and strongly rounded corners near the southern wall were discovered at a depth of 70 cm. The burial pit, measuring 200 x 70 x 50 cm with a wider northern end, was oriented along the south-north line. The central part of the grave was overlapped by a grave robber's trench, which partially destroyed the outlines of the eastern wall. At a depth of 90 cm, a burial was uncovered, disturbed in the chest and waist area. The skull with the shoulder girdle, as well as the lower limbs together with the pelvis, remained undisturbed. Judging by their position, the deceased was buried lying on their back, with the head to the north. The position of the arms could not be determined, as the arm bones and part of the ribs were found in a mixed condition near the pelvic bones. Near the skull, to the right of it, stood a vessel, while ordinary twisted pins and a copper bead lay on the pelvic bones, and a knife was found between the thighs. Remnants of bark from a coffin were found between the shins, and occasional coal inclusions were noted in the fill (Semenov, 1980).

## III.G. The sites of the Karayakupovo horizon in the Cis-Urals (Cis-Urals_KH)

**Genetic results.** *In this group, we analyzed five individuals from the site of Karanayevo (n=5). All five individuals had sufficient coverage for downstream population genetic modeling (>100,000 SNPs), and all were also suitable for IBD-sharing analysis (>600,000 SNPs). No close relatedness was detected among these samples, and the female-to-male ratio was 0:5. The samples displayed genetic similarities to a Late Iron Age Tobol region individual. The supervised ADMIXTURE analysis revealed elevated levels of Samara_EBA_Yamnaya and Yakutia_LNBA ancestries in this group, similar to those observed in the Migration Period Belaya Kushnarenkovo and Iron Age Tobol Sargatka groups. The Cis-Urals_KH samples clustered within the Urals-Carpathian EMA cluster, showing strong connectedness to other Karayakupovo Horizon groups and Early Medieval Magyars.*

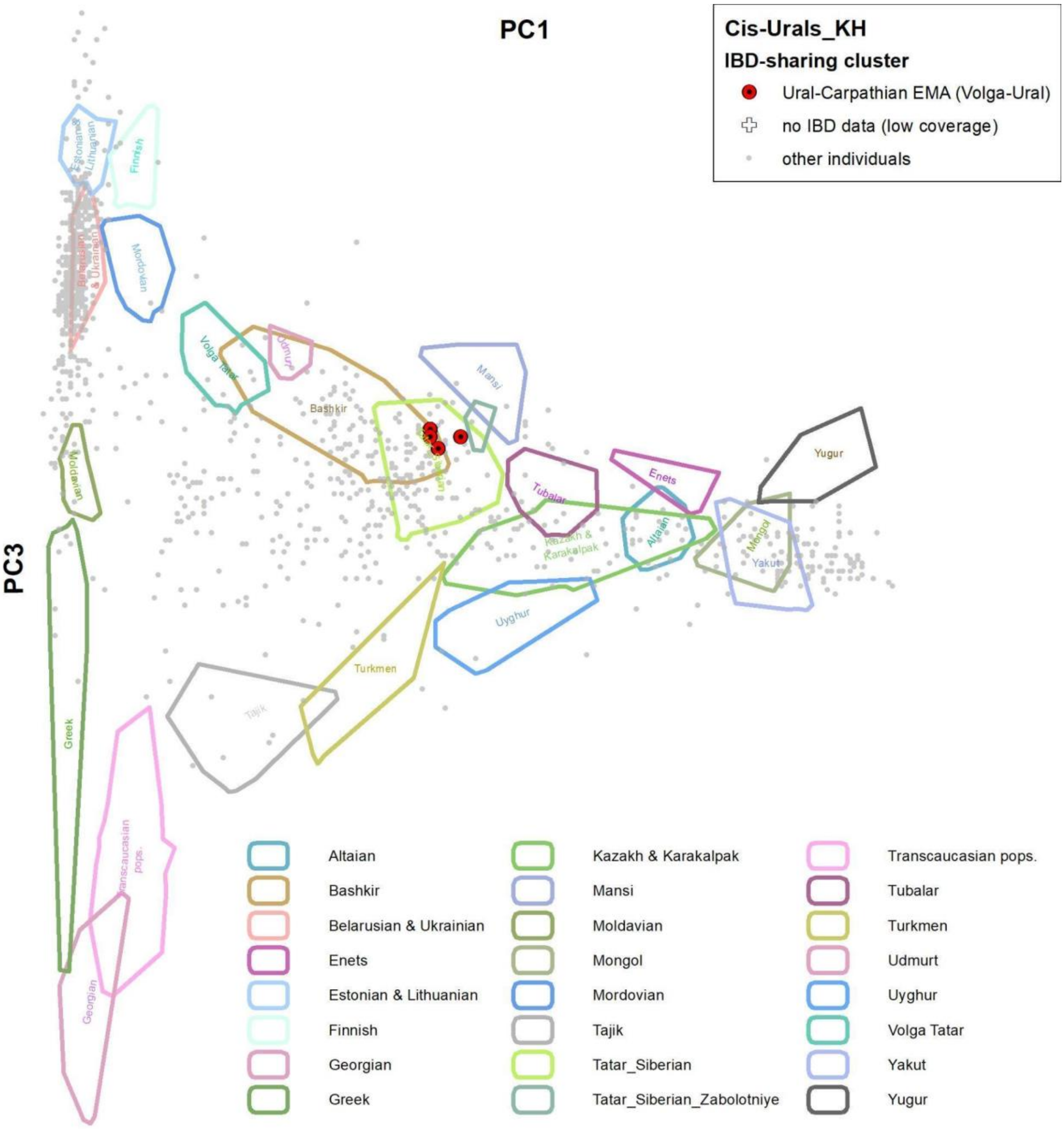


***Figure SIII.20.*** *Eurasian-scale principal component analysis (PCA), with a projection of the newly sequenced individuals onto modern genetic variation following Jeong et al. 2019. The PC1 and PC3 dimensions are shown, including the newly presented genomes. Polygons represent example populations from modern Eurasian individuals onto which the ancient samples were projected, while grey dots indicate ancient reference samples from our IBD-sharing network.*

## III.G.1. Karanayevo burial site

The burial site is located one kilometer south of the village of Karanaevo in the Mechetlinsky district, three kilometers north of the village of Abdrashitovo in the Duvansky district of the Republic of Bashkortostan, on a high (253.6 m) plowed elevation called Barag, 750 meters to the left of the Ufa–Yekaterinburg highway. The burial mounds covered an area of no less than 10,000 square meters.

The site was discovered and first investigated by Niyaz Mazhitov in 1964 and 1966, with 18 burial mounds excavated, providing rich material on the history and culture of the nomads of the 10th-12th centuries. In 2000 archaeologists were informed about intensive looting of the cemetery, and the rescue excavations of the site were organized. Despite the fact that the burial ground was previously recorded as a kurgan site, long-term plowing of the surface led to the complete leveling of the burial mounds in the 2000s, and they were no longer visually identified on the surface. This circumstance was the main reason why excavations were carried out on it as on a flat burial ground. The new excavation block was located on the plan drawn up by Niyaz Mazhitov in 1964, and it was recognized that the new excavation covers the area over three mounds located west of those excavated by N.A. Mazhitov (mounds 8, 9, 10). Thus, in 2001, 1080 square meters were excavated at the Karanaevo burial ground and 12 burials were investigated, providing new data on the material culture and burial rites of the population of the Southern Urals in the 10th-12th centuries.

We sequenced five individuals from the Karanayevo burial site.

### *burial 3 (individual ID I25535, Male)*

The dimensions of the burial structure could not be determined. The anatomical position of the skeleton was disturbed, likely due to plowing. The human bones were arranged in an arc, with the skull oriented southward by the occipital part. The burial belonged to a male (anthropologically attributed as female) aged 20–25 years.

Beneath the base of the skull, a belt plate with a smooth surface and wavy edges was discovered (1). Near this item, amidst the vertebrae and rib bones, two iron (3, 4) and two bone (5, 6) arrowheads were found. To the north of these finds, beneath the sacral bone, five bone bow overlays were uncovered (7–10). To the northeast of the skull, near a humeral tubular bone, a pendant in the form of a half-jingle with a loop was found (2).

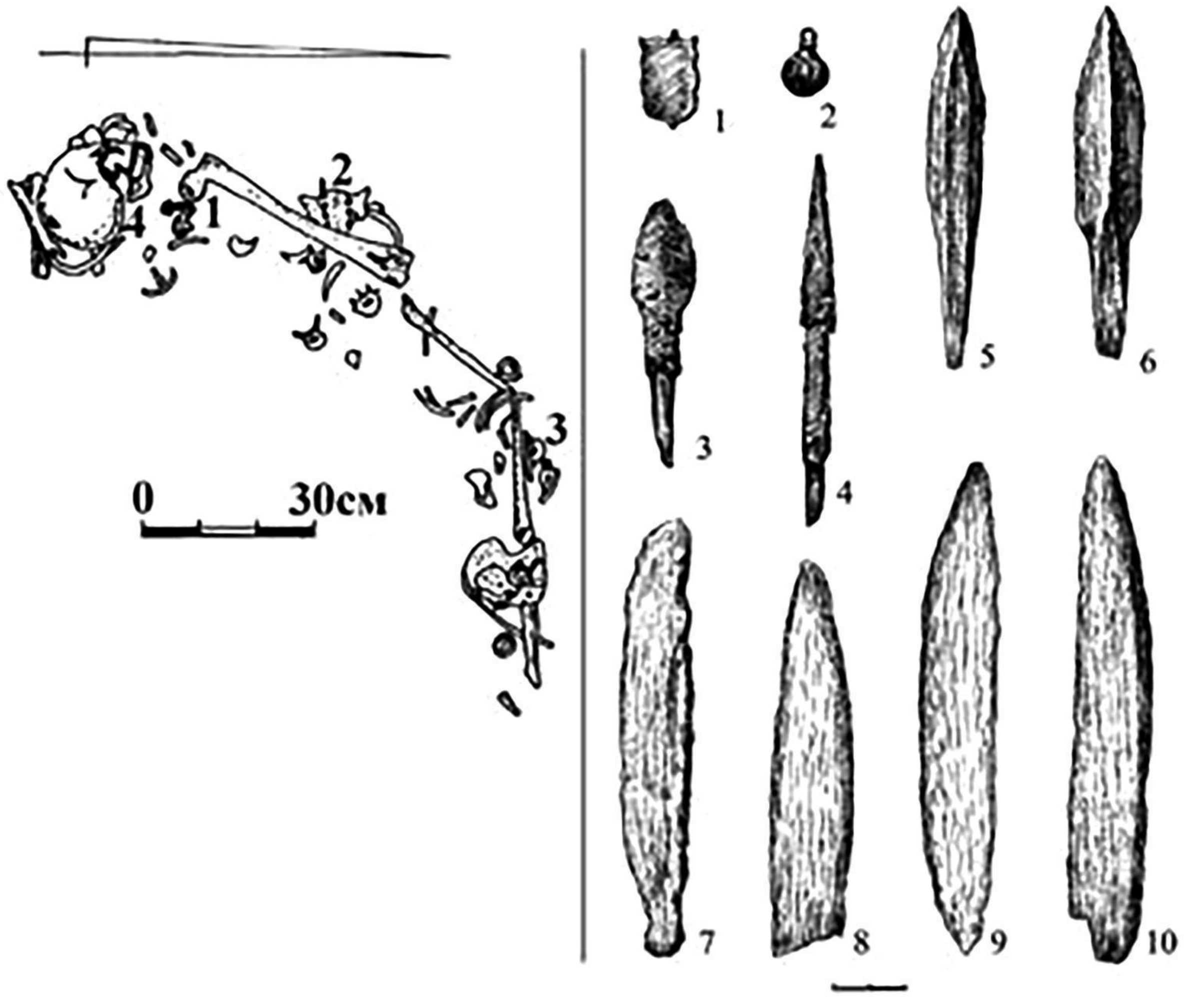


***Figure SIII.21.*** *Burial 3 from excavations at Karanaevo in 2001. Left: plan of the burial, right: grave goods.*

### *burial 6 (individual ID I25536, Male)*

The outline of the burial pit was identified at the subsoil level. The burial chamber, measuring 2.30 × 0.90 m, was dug into the clay to a depth of 0.35 m and oriented along the SSW–NNE line. Along the long walls of the northwestern half of the grave, traces of decayed wood were observed, likely remains of a coffin or covering.

The burial had been looted. Human bones were scattered across the bottom of the grave pit and were also found in its fill. The skull, overturned, was found in the central part of the burial chamber, 5 cm above its floor. Based on the skull, the burial was identified as that of a male aged 35–40 years.

Next to the skull were the mandible, a rib, a humerus, and a piece of decayed wood. In the southwestern half of the grave, pelvic bones, tibias, and a vertebra were found, while in the northeastern half, femurs, pelvic bones, and fragments of wood were uncovered. In the central part, near the western long wall, a humerus and a rib were identified.

Grave goods included an iron arrowhead (6) and two belt fittings (1, 2) found near the skull. In the fill of the southern half of the grave, at a depth of 15–20 cm from the mainland level, a complete stirrup with a wide footrest and a reinforcing rib (7), a fragment of another stirrup (5), a bone bow overlay (4), and an unidentified bone object (3) were discovered.

The determined radiocarbon age of the burial is 664-827 cal CE (1270±30 BP, Poz-136206) (based on human bone).

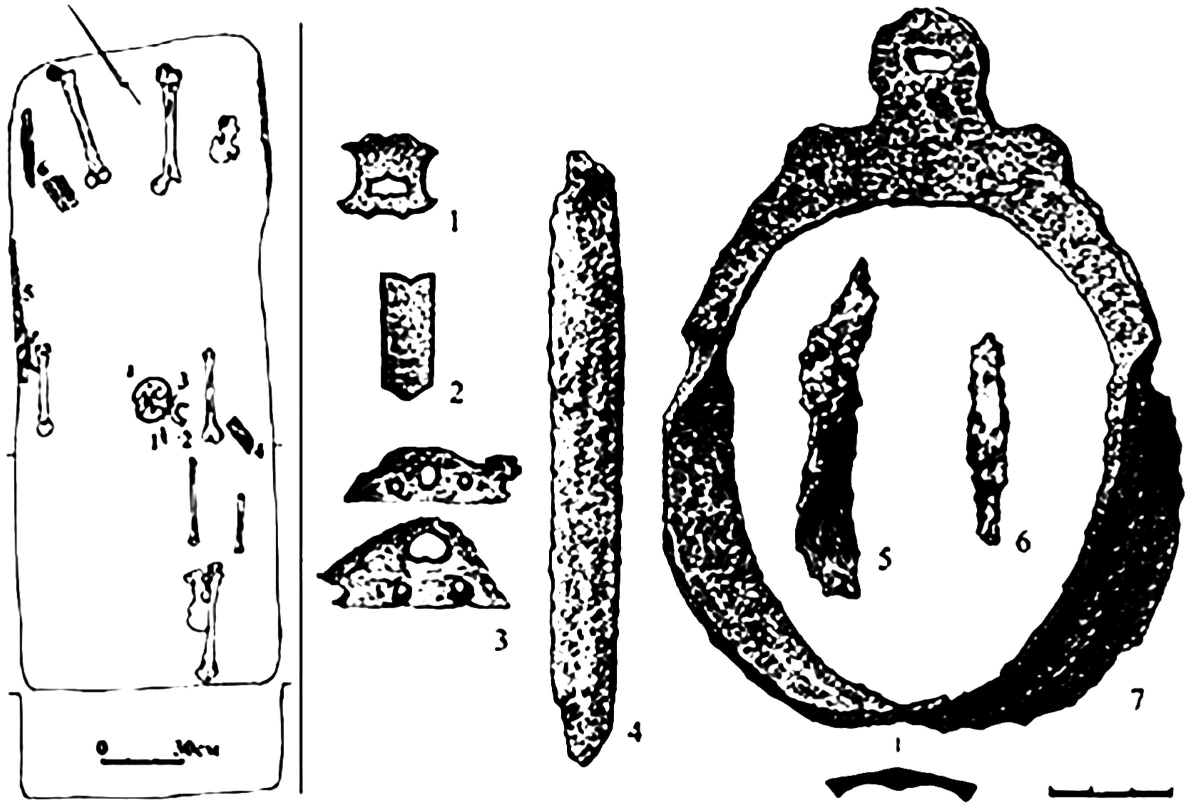

***Figure SIII.22.*** *Burial 6 from excavations at Karanaevo in 2001. Left: plan of the burial, right: grave goods.*

### *burial 8 (individual ID I25537, Male)*

The outline of the burial pit was identified at the subsoil level. The chamber measured 2.0 × 0.65 m and was dug into the clay to a depth of 0.20 m, oriented along the E–W line. It appears that this burial disturbed an earlier one, oriented along the N–S line, with approximate dimensions of 1.0 × 0.30 m.

The burial had been looted. Human bones were found scattered in disarray on the floor of the grave, primarily in its central part. These included tubular leg bones, the lower jaw, parts of the pelvis, ribs, and vertebrae. Animal bones were also found in the same area. At a depth of 5 cm from the mainland level, within the fill of the burial chamber, a human skull was discovered. It belonged to a male aged 40–45 years.

Artifacts found in the burial included two beads: a three-segmented bead made of dark blue glass (1) and another made of green glass (2). Other finds included a nail with a spherical

head (3), a heraldic-shaped overlay (4), a fragment of a bronze faceted bracelet with dot ornamentation (5), a fragment of a narrow bronze plate from an unidentified object (6), and two fragments of an iron knife (7).

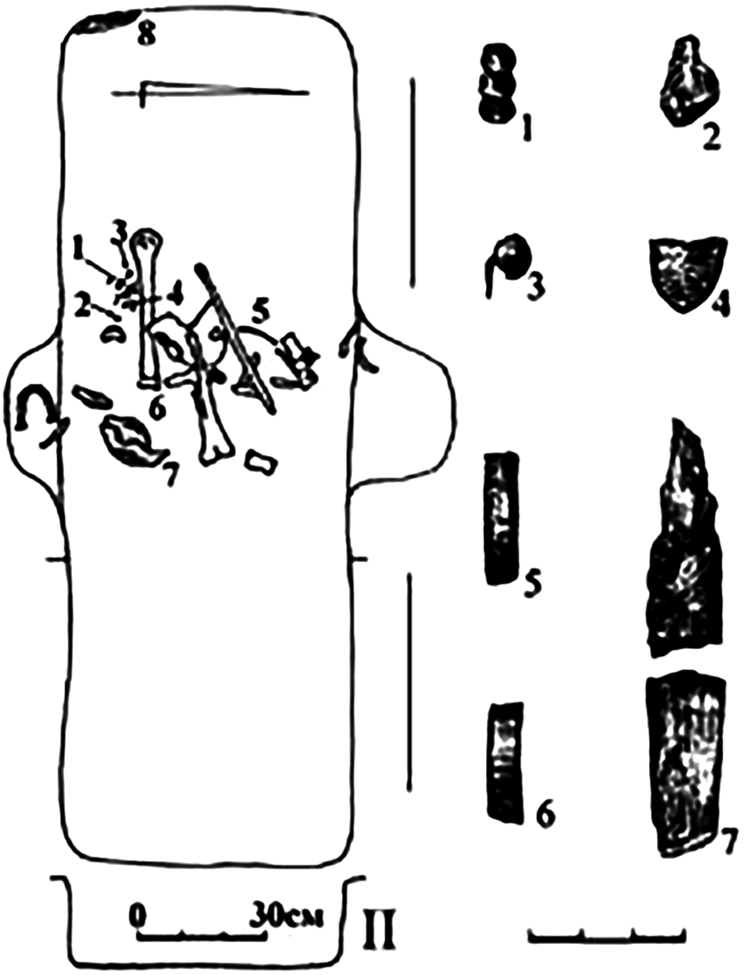


*Figure SIII.23. Burial 8 from excavations at Karanaevo in 2001. Left: plan of the burial, right: grave goods.*

### burial 12 (individual ID I25538, Male)

The outline of the burial pit was identified at a depth of (-45) cm from the ground surface at the subsoil level. The burial chamber measured 1.60 × 0.70 m, dug into the mainland clay to a depth of 0.15 m, and oriented along the E–W line with a slight deviation to the south. It had a rectangular shape with rounded corners and vertical walls.

The burial belonged to a male (anthropologically identified as female), aged 40–45 years. The deceased was laid out extended on his back, with his head oriented to the west, slightly deviated to the south. The skull rested on its occipital bone. There was partial disturbance of the skeleton, including the displacement of ribs and the absence of the tibiae.

A clay round-bottomed vessel (27) was placed slightly above and to the left of the skull. The vessel featured a straight neck, a pronounced shoulder, and a spherical body. The neck was 2.5 cm high, with a rim diameter of 9.3 cm, a maximum body diameter of 11.2 cm, and a height of 9.5 cm. The clay mixture contained fine crushed shells and sand inclusions. The surface was light brown in color, while the sherd's interior was black. The rim and upper part of the vessel's body were decorated with an ornament consisting of two horizonal bands of impressions made with a cord. The body of the vessel featured a multi-row horizonal zigzag pattern with vertically descending "towels," applied using a fine comb.

To the right of the skull, six beads were found: two flat-disk-shaped glass beads (1, 2), one carnelian bead of the same shape (3), two biconical carnelian beads (4, 5), and one biconical rock crystal bead (6).

To the left of the skull, near the mandible, a bronze ring with overlapping ends 23) was discovered. In the same area, 16 pear-shaped decorated pendants-jingles (7, 8, 10–13, 15–22, 24, 25) and an umbo-shaped plaque (10, 14) were found. A lyre-shaped buckle (9) was located near the pelvic bone.

At the feet of the buried individual, the skull and lower limbs of a horse were laid. The horse's skull faced west with its incisors, while the legs, oriented parallel to each other, pointed westward with their hooves toward the human skull. Near the horse's skull, a single-piece iron bit with movable rings (Fig. 10, 26) was found.

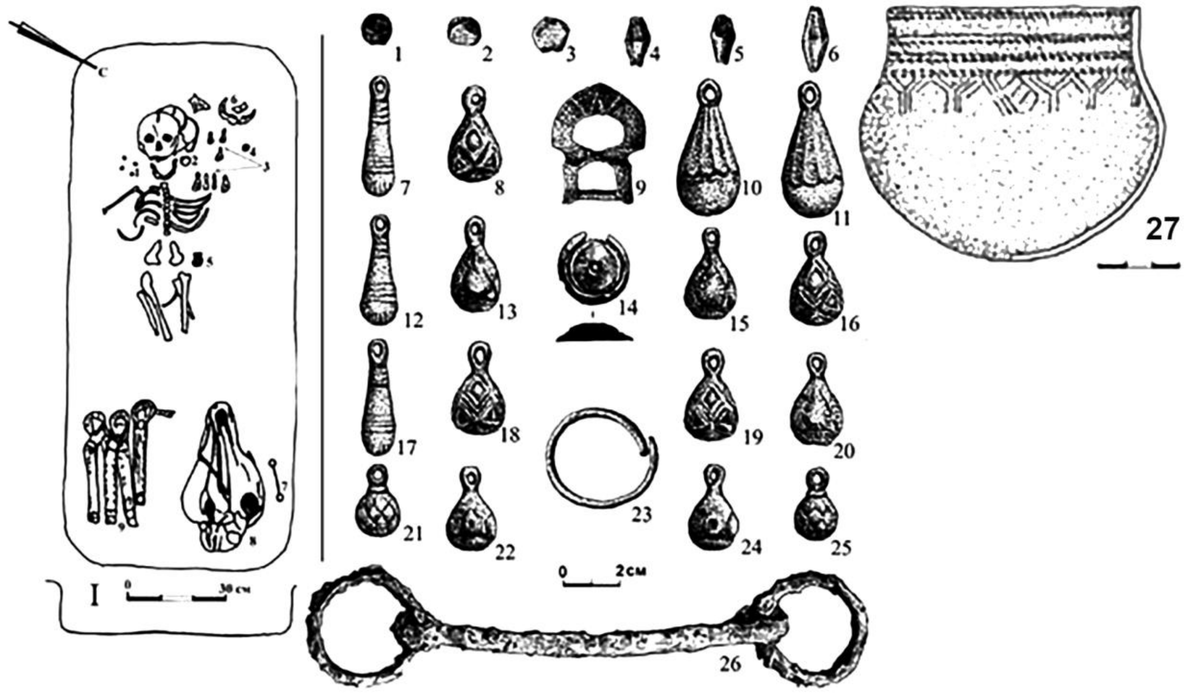

***Figure SIII.24.*** *Burial 12 from excavations at Karanaevo in 2001. Left: plan of the burial, right: grave goods.*

One more individual from Karanaevo labeled as excavation 2, grave 3 (individual ID I25539, Male). Unfortunately, we failed to identify the archaeological context of this individual. The determined radiocarbon age of the burial is 885-1016 cal CE (1105±30 BP, Poz-136207) (based on human bone).

## III.H. The Medieval Chiyalik culture in the Cis-Urals (Belaya_Chiyalik)

See Szeifert et al., 2022 and Garustovich, 2015 for the general description of the Medieval Chiyalik culture.

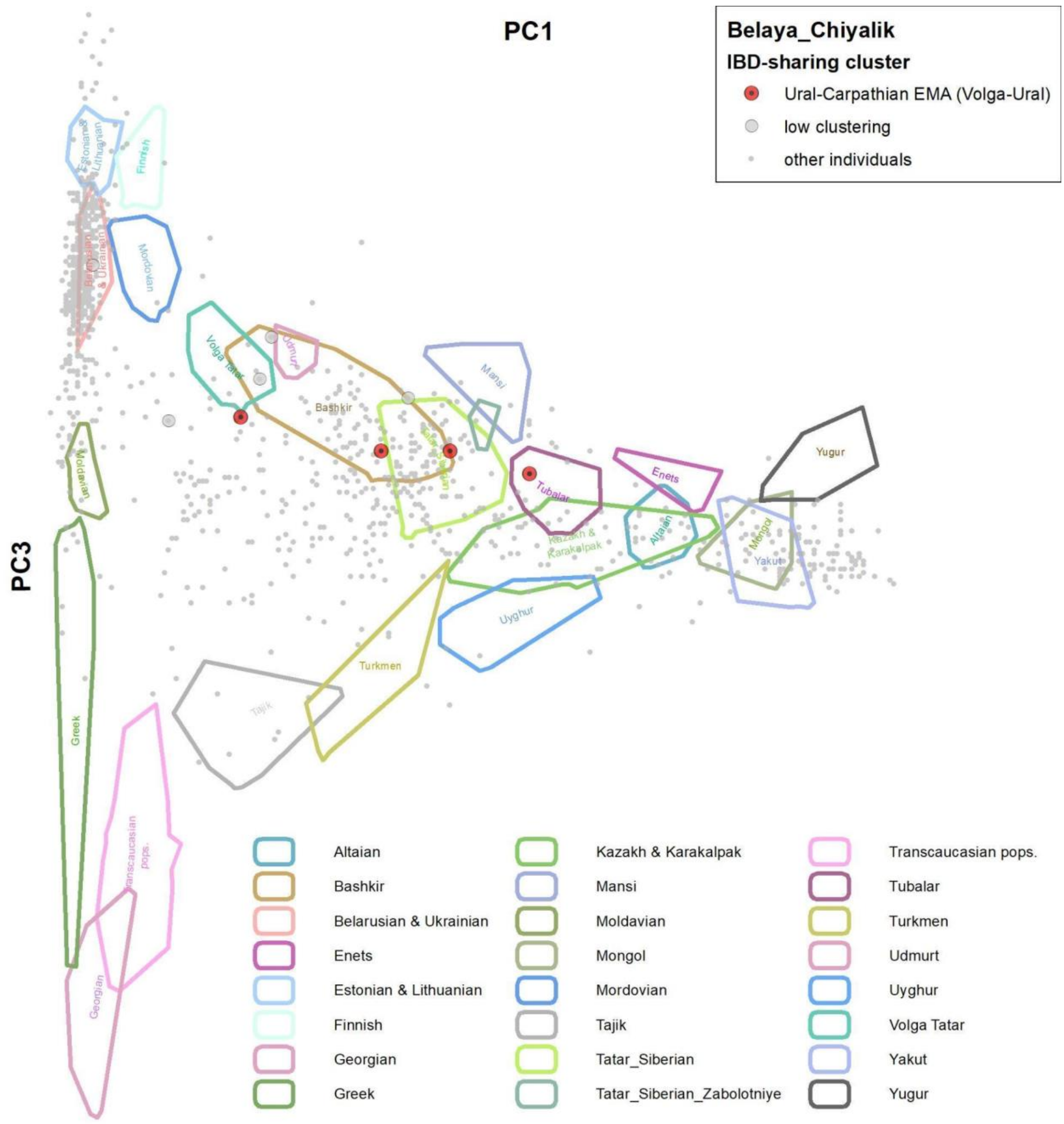


***Figure SIII.25.*** *Eurasian-scale principal component analysis (PCA), with a projection of the newly sequenced individuals onto modern genetic variation following Jeong et al. 2019. The PC1 and PC3 dimensions are shown, including the newly presented genomes. Polygons represent example populations from modern Eurasian individuals onto which the ancient samples were projected, while grey dots indicate ancient reference samples from our IBD-sharing network.*

**Genetic results.** *In this group, we analyzed nine individuals from the sites of Nizhne-Khozyatovo (n=7) and Gornovo (n=2). All individuals had sufficient coverage for downstream population genetic modeling (>100,000 SNPs) and IBD-sharing analysis (>600,000 SNPs). The individuals I25542 and I25545 from the Nizhne-Khozyatovo site are second-degree relatives. In this group the female-to-male ratio was 3:6. The samples display a diverse genetic profile, including some individuals with possible local Iron Age ancestry and others with ancestries from Europe and Eastern Eurasia. The supervised ADMIXTURE analysis revealed elevated levels of Baikal_N and Anatolian_N ancestries in this group. Four Belaya_Chiyalik*

*samples clustered within the Urals-Carpathian EMA cluster, while five samples had low clustering coefficients. These genetic results suggest possible genetic continuity in the Belaya region from the Early Medieval Karayakupovo Horizon, while also indicating gene flow from unexplored Eurasian regions.*

## III.H.1. Gornovo burial ground

**burial 2 (individual ID I25548, Male)**

The burial pit was recorded at level -111 from point 0 in square 3. It was sub-rectangular in shape with rounded corners, measuring 1.25 m in length and 0.4–0.43 m in width, and deepened 0.03–0.05 m into the subsoil. The bottom level was at -1.16 from the benchmark. The pit was filled with black humus mixed with subsoil sandy loam.

Layers of possible coffin decay were observed above and along the sides of the skeleton during excavation. The skeleton belonged to a child aged 2–3 years, lying extended on its back, slightly turned to the right. The head was oriented to the west, facing south. The right arm was extended along the body, while the left arm was half-bent with the hand resting on the pelvic bones. The legs were extended. The skeleton was in satisfactory preservation.

Flattened clay balls were discovered at the location of the cervical vertebrae. Radiocarbon dating of the burial, based on human bone, provided an age range of 1178–1276 cal CE (810±30 BP, Poz-136209).

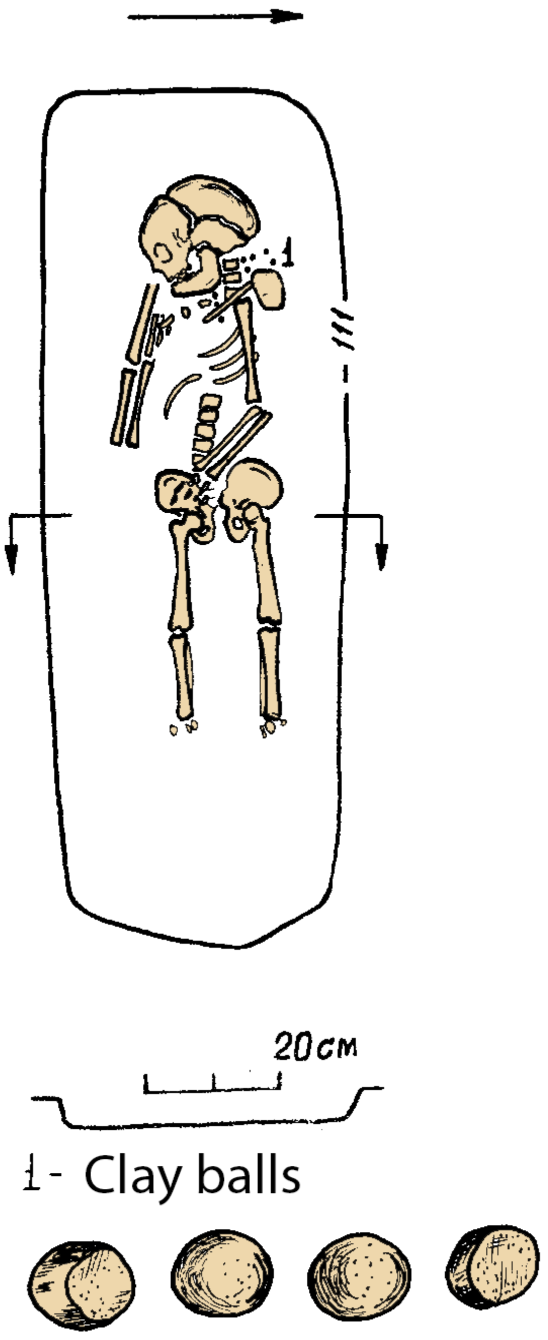


***Figure SIII.26.*** *Burial 2 at Gornovo.*

**burial 4 (individual ID I25547, Female)**

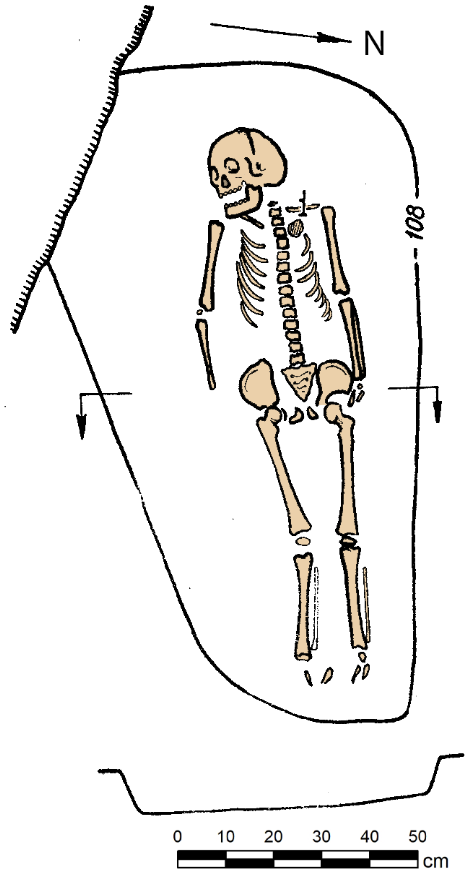


The grave pit was of a sub-rectangular shape with rounded corners, identified at a depth of 108 cm. The pit measured 1.38 m in length and 0.45–0.9 m in width, narrowing significantly at the feet. It was deepened 0.05–0.1 m into the subsoil and filled with humus.

The remains of a 4–5-year-old child were found lying extended on their back, with her head oriented to the west, slightly deviating to the southwest, and facing south. The arms and legs were extended. Notably, the body was buried in a manner such that the legs were positioned higher than the head.

The bones are in a satisfactory state of preservation, and no grave goods were present.

***Figure SIII.27.*** *Burial 4 at Gornovo.*

## III.H.2. Nizhne-Khozyatovo burial ground

**burial 9 (individual ID I25543, Male)**

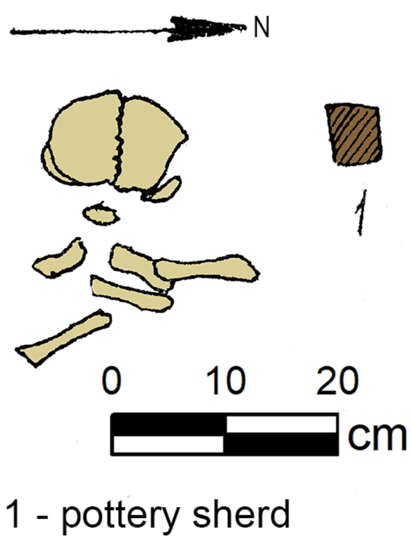


The boundaries of the grave pit were not discernible. The poorly preserved skeleton belonged to a newborn. The deceased was likely placed in a supine position, with the head oriented to the west. The arm and leg bones were positioned closely together. To the north of the skull, a large pottery sherd attributed to the Srubnaya culture was uncovered, likely an accidental inclusion in the grave infill. No grave goods were identified.

The determined radiocarbon age of the burial is 1274-1389 cal CE (685±30 BP, Poz-136325) (based on human bone).

***Figure SIII.28.*** *Burial 9 at Nizhne-Khozyatovo.*

## burial 10 (individual ID I25544, Male)

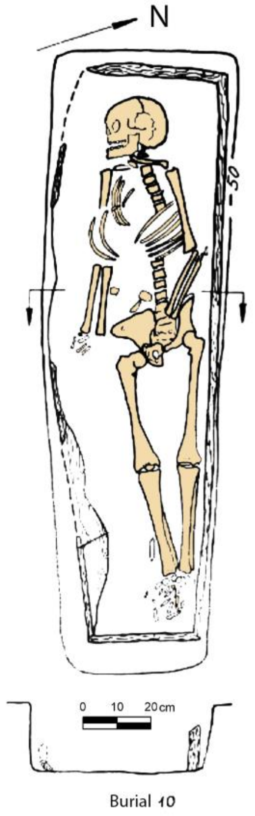


The burial extended beyond the northern wall of the trench, requiring an extension during the clearing process. The contours of the burial pit were identified at a depth of 0.5 m. The pit is rectangular with rounded corners, measuring 1.85 m in length and 0.54 m in width. It is simple in structure, incised into loamy subsoil with a depth of 0.2 m, featuring a flat bottom and filled with black humus.

The remains belonged to an adult interred in a poorly preserved coffin that narrowed at the feet. The coffin measured 1.71 m in length, 0.34–0.5 m in width, approximately 0.15 m in height, and included a lid but possibly lacked a bottom. The individual was positioned extended on their back, slightly turned to the right side, with the head oriented between west and southwest, facing south. The right arm was extended, while the left arm was bent at the elbow, with the hand resting on the pelvic bones. The legs were extended and joined at the ankle joints. No grave goods were found. The skeleton was in satisfactory condition.

The determined radiocarbon age of the burial is 1225-1295 cal CE (745±30 BP, Poz-136326) (based on human bone).

***Figure SIII.29.*** *Burial 10 at Nizhne-Khozyatovo.*

## burial 11 (individual ID I25545, Male)

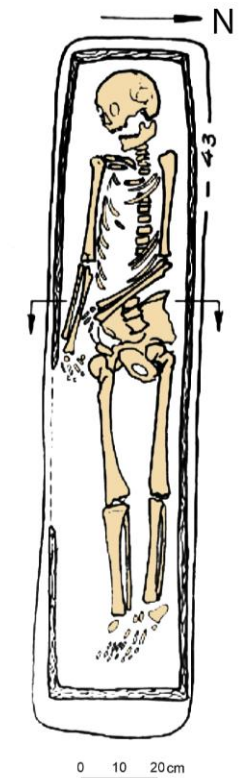


The contours of the grave pit were recorded at the level of 0.43m, the pit is rectangular, simple, length 1.8m, width 0.4m, buried in the soil by 0.16m. The bottom is flat. Filling - black humus.

The skeleton rested in a coffin made in the form of a rectangular box, without bottom and lid (?), 1.67m long, 0.32-0.36m wide, 0.12m high, poorly preserved.

The position of the deceased stretched out on his back with a slight turning of the body on the right side, head oriented to the west, face - to the south. The right arm and legs are stretched out, the left arm is half-bent, with the hand on the right side. There are no belongings. Preservation of the bones is satisfactory.

***Figure SIII.30.*** *Burial 11 at Nizhne-Khozyatovo.*

**burial 13 (individual ID I25546, Female)**

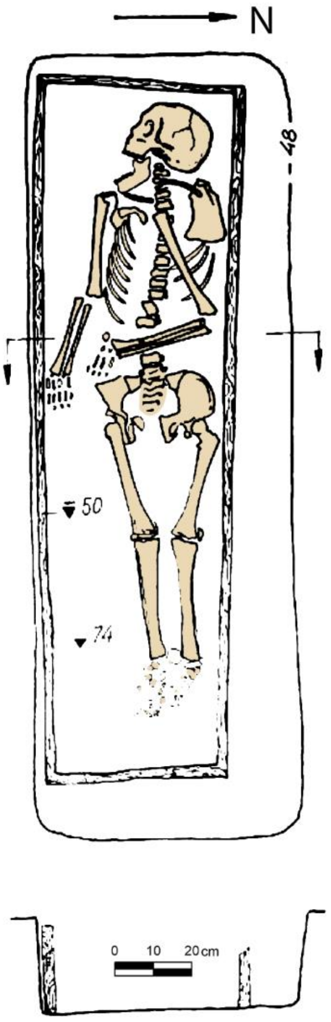


The contours of the grave were recorded at a depth of 0.48 m. The grave pit was rectangular with rounded corners, simple in design, measuring 2.1 m in length, 0.7 m in width, and incised 0.25 m into the soil. The bottom is flat, and the filling consists of humus.

The deceased individual, genetically female, was placed in a wooden coffin-frame without a bottom or lid, rectangular in shape, measuring 1.9 m in length, 0.48–0.55 m in width, and 0.23 m in height.

The skeleton was positioned stretched out on the back with a slight turn of the body to the right. The head was oriented to the west, facing south. The right arm was extended and positioned away from the torso on the right side. The left arm was bent at the elbow, with the hand resting on the right side. The legs were fully extended. No grave goods were found. The preservation of the skeleton is good.

The determined radiocarbon age of the burial is 1226-1377 cal CE (735±30 BP, Poz-136327) (based on human bone).

***Figure SIII.31.*** *Burial 13 at Nizhne-Khozyatovo.*

For burials 1 (individual ID I25540, Male), 2 (individual ID I25541, Female), and 6 (individual ID I25542, Male) archaeological descriptions are not available. The determined radiocarbon age of burial 1 is 1281-1395 cal CE (650±30 BP, Poz-136208) (based on human bone), burial 2 - 1300-1406 cal CE (605±30 BP, Poz-136246) (based on human bone), burial 6 - 1280-1395 cal CE (655±30 BP, Poz-136324) (based on human bone).

# IV. The Trans-Urals and the Western Siberia

## IV.A. The Early Iron Age Sargatka culture (MidIrtysh_Sargatka)

For the general description of the culture, see Matveeva N., 1997, 2000, 2016, 2018.

**Genetic results.** *In this group, we analyzed four individuals from the sites of Bogdanovo-2 (n=2) and Borovyanka-18 (n=2). All had sufficient coverage for downstream population genetic modeling (>100,000 SNPs), and for the IBD-sharing analysis (>600,000 SNPs). The individuals I30386 and I30387 are second-degree relatives. In this group the female-to-male*

*ratio was 4:0. The supervised ADMIXTURE analysis revealed elevated levels of Samara_EBA_Yamnaya and Yakutia_LNBA ancestries in this group. The MidIrtysh_Sargatka samples are clustered within the Eurasian Steppe IA cluster. This group displayed a distinct ancestral composition compared to the Tobol_Sargatka group, emphasizing the presence of diverse ancestry-bearing populations in the area. The IBD network suggests a much closer affinity of these individuals to the steppe population of the Saka and Sarmatian groups, as these samples clustered in the Eurasian Steppe IA cluster.*

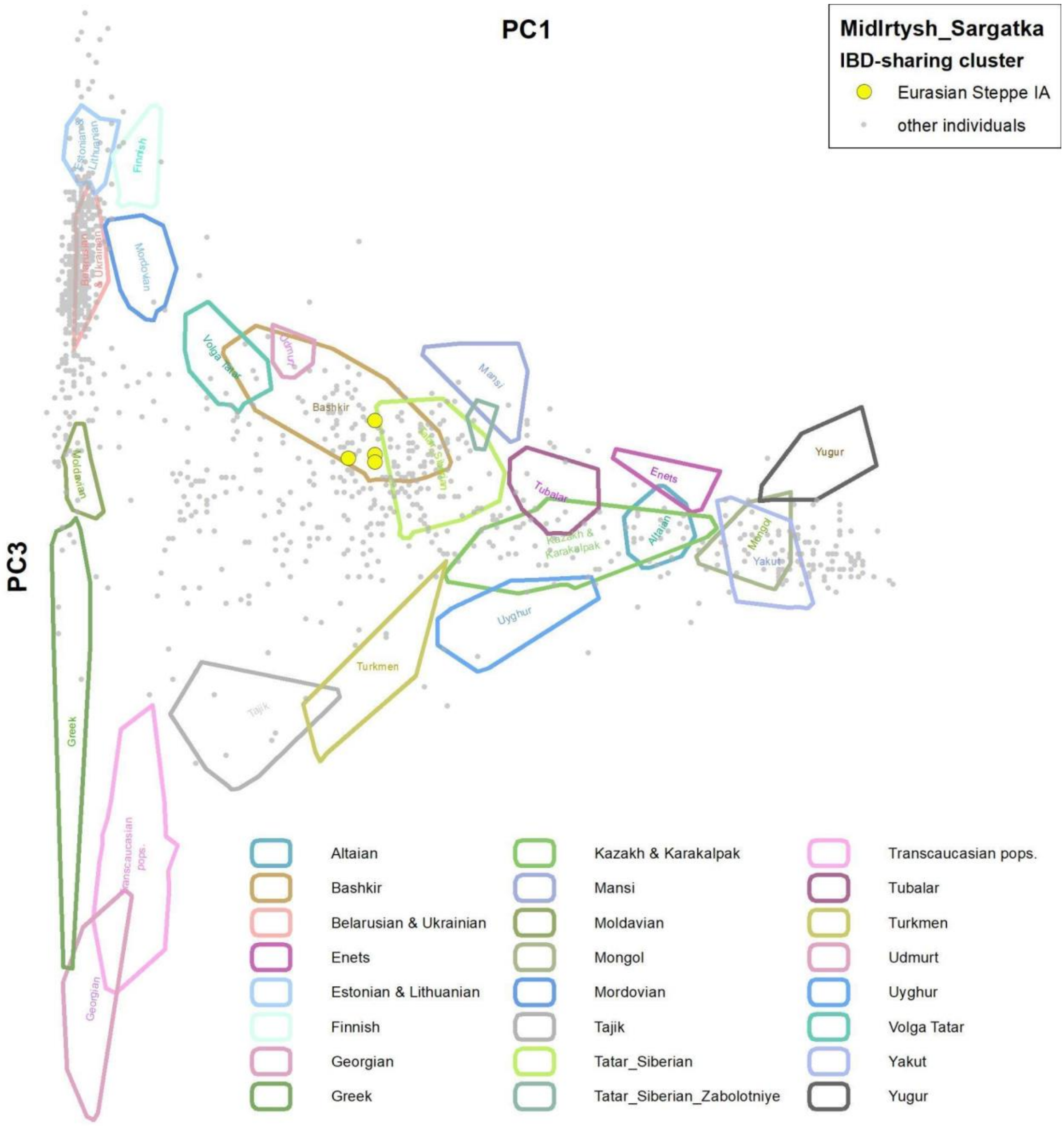


***Figure SIV.1.*** *Eurasian-scale principal component analysis (PCA), with a projection of the newly sequenced individuals onto modern genetic variation following Jeong et al. 2019. The PC1 and PC3 dimensions are shown, including the newly presented genomes. Polygons represent example populations from modern Eurasian individuals onto which the ancient samples were projected, while grey dots indicate ancient reference samples from our IBD-sharing network.*

## IV.A.1. Bogdanovo-2

The burial site is located in the Kormilovsky district of the Omsk region, near the Om' River, a tributary of the Irtysh River. It was investigated in 1977 by V.A. Mogilnikov. We involve into this study two individuals buried under mound 2, individual ID I30386, Female and individual ID I30387, Female. The central burial under that mound was completely looted, and around its grave pit, on the ancient ground surface, remains of three individuals, including an infant, were found. Based on an earring, a bronze cauldron, and iron chain reins, the burial mound is dated to the 2nd century BCE – 1st century CE. The archaeological context has not been published yet.

## IV.A.2. Borovyanka-18

### *mound 13, burial 2 (individual ID I10117, Female)*

The determined radiocarbon age of the burial is 385-206 cal BCE (2240±20 BP, PSUAMS-9091) (based on human bone).

### *mound 3, burial 1 (individual ID I10118, Female)*

The determined radiocarbon age of the burial is 382-206 cal BCE (2235±20 BP, PSUAMS-9122) (based on human bone).

# IV.B. The Migration period sites of the late phase of the Sargatka culture (MidIrtysh_LSargatka & Tobol_LSargatka)

**Genetic results.**

*In the MidIrtysh_LSargatka group, we analyzed one individual from the site of Borovyanka-17 (n=1). This male had sufficient coverage for downstream population genetic modeling (>100,000 SNPs) and was suitable for IBD-sharing analysis (>600,000 SNPs). The individual displayed a high level of West Siberian ancestry, suggesting possible gene flow from Western or Central Siberia. The supervised ADMIXTURE analysis revealed elevated levels of Yakutia_LNBA/Russia_EHG and Altai_N ancestries. The MidIrtysh_LSargatka sample did not cluster within any specific group. This individual's unique genetic composition points to a Western Siberian or even Central Siberian origin.*

*In the Tobol_LSargatka group, we analyzed two individuals from the sites of Starolybaevo-4 (n=1) and Ipkul' (n=1). Both had sufficient coverage for downstream population genetic modeling (>100,000 SNPs) and were suitable for IBD-sharing analysis (>600,000 SNPs). No close relatedness was detected among these samples, and the female-to-male ratio was 0:2. The supervised ADMIXTURE analysis revealed elevated levels of Samara_EBA_Yamnaya and Yakutia_LNBA ancestries in this group. The sample from the Ipkul site clustered within the Urals-Carpathian EMA. The similar genetic composition, the connection to the Urals-Carpathian EMA cluster, and the f-statistics-based analysis all suggest that this group could*

*be a possible ancestor of the later Karayakupovo Horizon individuals and an important stratum of the Early Medieval Magyars.*

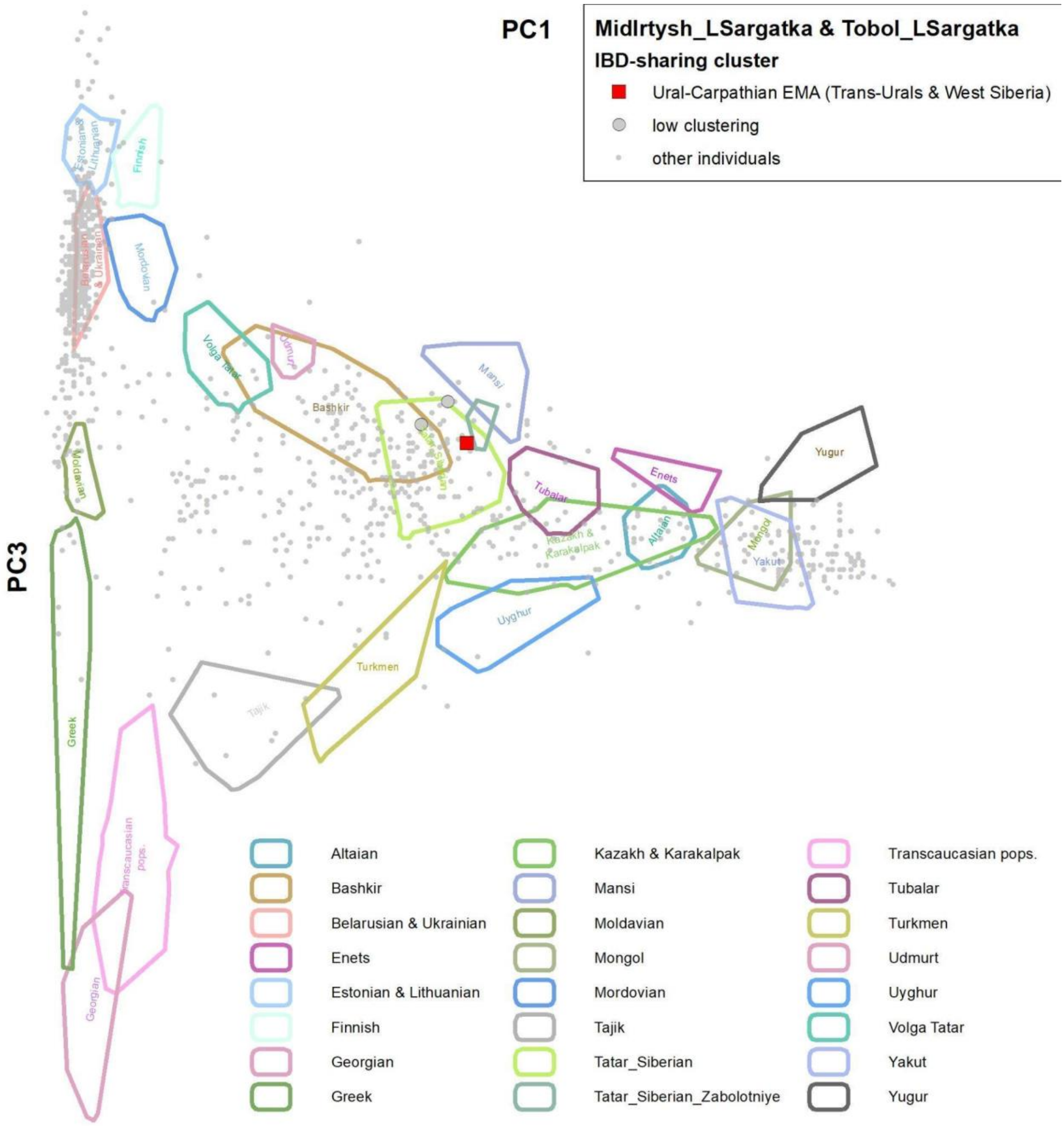


***Figure SIV.2.*** *Eurasian-scale principal component analysis (PCA), with a projection of the newly sequenced individuals onto modern genetic variation following Jeong et al. 2019. The PC1 and PC3 dimensions are shown, including the newly presented genomes. Polygons represent example populations from modern Eurasian individuals onto which the ancient samples were projected, while grey dots indicate ancient reference samples from our IBD-sharing network.*

## IV.B.1. Borovyanka-17 burial ground

### *burial 46 (individual ID I6826, Male)*

The determined radiocarbon age of the burial is 262-420 cal CE (1680±20 BP, PSUAMS-4827) (based on human bone).

## IV.B.2. Ipkul' kurgan burial site

The Ipkul’ burial site is located in the Nizhnetavdinsky District of the Tyumen Region, it is situated at the northern shore of Lake Ipkul’. The site was discovered in 1971 by M.F. Kosarev and V.F. Starkov, and it was studied in 1983 by L.N. Koryakova (1984; 1988). Subsequent research over two years by the expedition of the Ural State University excavated 7 mounds (1, 2, 3, 6, 14, 28, 39), with another 2 (27 and 44) mounds investigated by trenches (Koryakova, 1988; Koryakova, Morozov, Sukhanova, 1988).

In 2010–2011, I.Yu. Chikunova documented 36 mounds elongated in small groups in the latitudinal direction (Chikunova, 2017). The majority of the necropolis is located on an open, flood-prone clearing overgrown with meadow grass and small shrubs, while the northern part is in a mixed coniferous-deciduous forest. Some mound structures are almost entirely leveled, with a height not exceeding 0.2 m, identified only through instrumental surveying. New numbers have been assigned to the documented mound structures, except for those previously used by the Ural State University expedition. Excavations were conducted on mounds 4, 5, 7, 9, 13, 18, 19, 20, 22, 25, 29, 36, 40, 41 (12), 42 (2), 43 (3). Additionally, the raised areas 10 and 12 were investigated, revealing well-preserved piles of sand and construction debris.

The inventory from the burials, comprisyы ceramic vessels of both Sargatka and taiga-derived types, and costume details and belt fittings characteristic for the Late Sarmatian tradition. Based on a wide circle of parallels to the latter found in the European steppe and radiocarbon dates (Zelenkov and Tretyakov 2025) is dated back to the 3rd-4th centuries CE. The site represents one of the northernmost and latest manifestations of the Sargatka tradition, situated in the remote periphery of its cultural area. By this time, the culture had already disappeared from most of its core territory, and at Ipkul, Sargatka traits persisted only partially, in combination with elements from the taiga zone. These elements subsequently influenced the formation of the Bakalda culture, which flourished during the Migration Period and the early Middle Ages (4th–8th centuries CE). The Ipku’l burial site represents the residual Late Sargatka–transitional horizon, reflecting both the persistence of older traditions and their admixture with northern forest cultural traits (Sharapova 2020).

### *mound 13, burial 1, ind. 1 (individual ID I33844, Male)*

The burial in the pit of a rectangular shape was located at the center of the mound. It measured 2.1×0.9 meters with a depth of 0.2 meters and was oriented along the north-south axis. The deceased, a male aged 35-45 years, was laid with his head to the north. His hands were resting on the pelvic bones. Corroded fragments of an iron knife handle, 13 centimeters in length, with remnants of iron sheathing for leather or wooden scabbards, were found on the

right side of the pelvic bones. On the left side, near the lower edge of the ribs, a bronze round-frame buckle with a rounded shield and a movable tongue was discovered.

Pit 1, similar in configuration and orientation to burial 1, was positioned parallel to it with a displacement to the southwest. The fill consisted of dark gray loam. In the center, there was an inverted large undecorated pot of Bakalda type without a neck, filled with charcoal. No other

finds were made. The burial is dated to the second half of the third - the first decades of the 4th centuries CE (~2500-320 CE), based on the belt buckle (Sharapova 2020).

***Figure SIV.3.*** *Ipkul' burial site, mound 13, burial 1. A - a plan of the kurgan, B - a plan of the burial, C - grave goods, D - Bakalda-type pottery from the grave.*

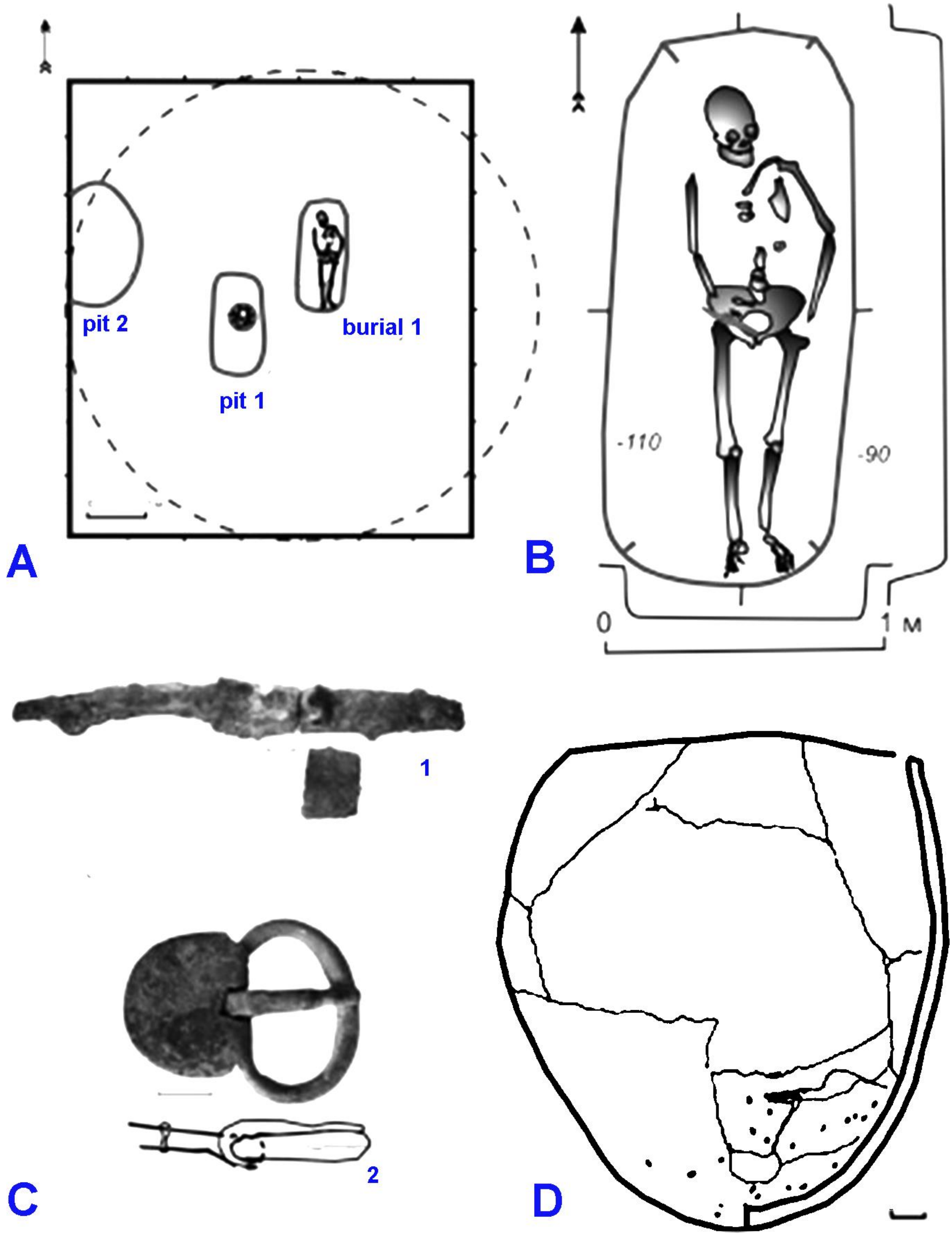

## IV.B.3. Starolybaevo-4 kurgan burial site

The burial site is located approximately 4.5 km to the northwest from the village of Staro-Lybaevo, in the Zavodoukovsky district of the Tyumen region. The cemetery occupies an area on the elevated right bank of the Iset River, covered by mixed forest, adjacent to the vicinity of the Schetkovo lake. It was discovered by I. V. Zhilin in 1982 and surveyed in 1995.

A total of 41 mounds have been identified. Undisturbed mounds are situated along the forest edge, stretching in a northwest to southeast direction, well-preserved but showing signs of looting and badger holes. These include 18 mounds numbered 1-13, 16-18, 21, and 22. Additionally, six ploughed mounds were found along a sandy country road parallel to the aforementioned kurgans, within a distance of 15-30 meters, likely belonging to the same group. Another group consisting of 15 mounds forms an irregular chain oriented from north to south, extending from the forest edge directly across the field to the Schetkovo lake.

Finds from mounds of kurgans 31, 34, and 35 date back to the Neolithic-Chalcolithic period, representing remnants of short-term settlements along the shore of the now dried-up lake, to the edge of which the burial ground is presumably associated.

### *mound 34, burial 2, NE sector, ind. 1 (individual ID I32779, Male)*

Mound 34 had a diameter of approximately 15 meters and a height of 0.35 meters. The mound was regularly plowed for a long time, making it difficult to accurately determine its contours, resulting in the conditional center of the mound being slightly shifted to the southwest. Beneath the mound, a cultural layer from the Chalcolithic period was discovered.

Several post holes indicated the presence of some ancient above-ground structure. During excavations, it was found that the ditch surrounding the platform contained two main burials and had a polygonal shape, presumably hexagonal. The width of the ditch varied from 0.8 to 1.1 meters, with a depth of 0.25-0.35 meters. On the inner side of the ditch from the west, a lens of burning measuring 1.0x0.4 meters was identified. The dimensions of the platform outlined by the ditch were 11x13 meters. In the northwest sector, a collapsed Sargatka pot with scallops was found at a depth of 40 cm below the surface.

The mound was constructed during the Sargatka period, and burials 1 and 2 are associated with the same time, as it is clear from numerous Sargatka artifacts and details of burial rites. Both burials were later disturbed by ancient robbers. The exact period when the robberies took place is unknown, however, they must be dated to the Bakalda period, as in one of the graves of the nearby kurgan 35 of the same cemetery, sherds of the Bakalda pottery were found in robbed Sargatka grave pits.

Burial 2. The central grave in the mound, with a rectangular shape, had dimensions of the entrance pit, damaged by looters, measuring 4.25x3.0 meters. Debris lenses, created to the north and southeast, slid into the grave. During the excavation of the grave infill, the contours of the mound contracted and took on a more regular form. The chamber dimensions at a depth of 0.5 meters from the ground level were 2.5x3.2 meters. Particularly, on the northeast side, a flat platform was revealed, possibly serving as a step or resulting from a secondary burial cut into the northeast edge of the central mound. Supporting the latter hypothesis is the

discovery of two intact and two damaged skulls in the grave, as well as the finding of articulated pelvic bones, femurs, radius, and ulna of the left arm belonging to a body that had not decomposed at the time of looting. This indicates multiple-stage burials in this chamber. The total depth of the grave was 0.86 meters, and the dimensions of the depression at the bottom were 2.0x1.5 meters. Due to looting, only fragments of pottery were found, the collapse of a small round-bottomed pot with notches along the shoulders, a spindle whorl, fragments of an iron sword blade, and an iron three-bladed arrowhead.

Anthropological analysis suggested the presence of six individuals: two adult women, as well as women approximately 45 years old, 40-50 years old, 30-55 years old, and around 60 years old. However, the genetic analysis confirmed male sex of the individual ID I32779, whose age was anthropologically determined as 40-50 years old.

Individual I32779 was found in the north-eastern part of burial 2 and likely belongs to the secondary burial, which partially damages the initial grave pit. This burial contains redeposited parts of skeletons that were still not completely decomposed at the moment of redeposition. This suggests that the moment of the robbery was close to the moment of the burial (not more than 20 years). Based on this observation, we date Individual I32779 to the Late Sargatka or the Bakalda phase of the cemetery.

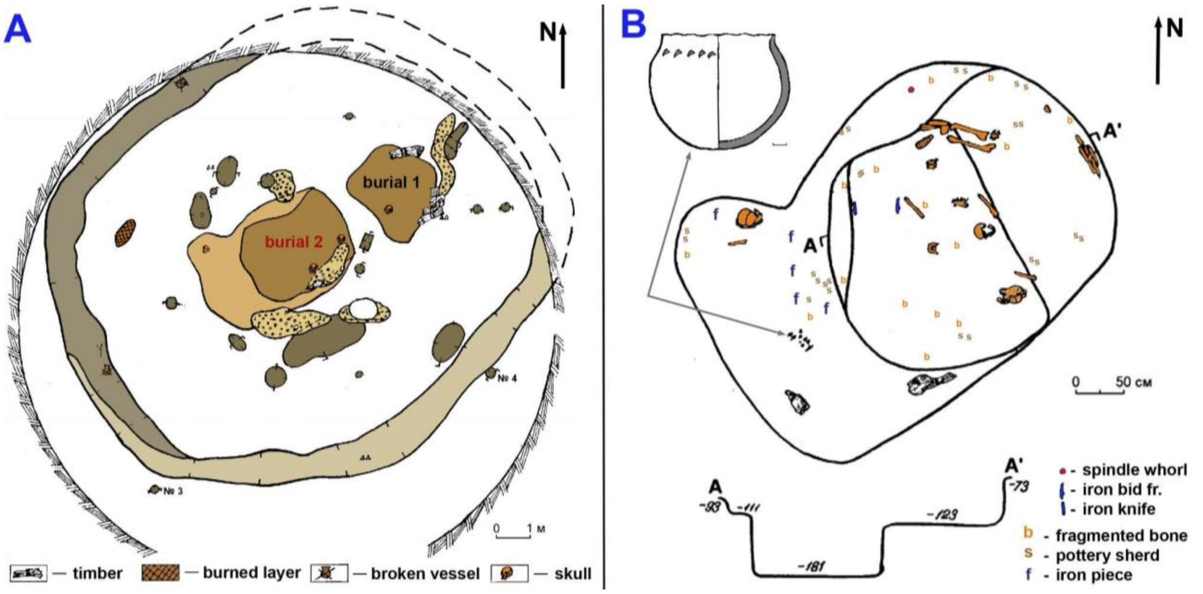


***Figure SIV.4.*** *Starolybayevo-4 burial site, mound 34. A - a plan of the kurgan, B - a plan of burial 2.*

## IV.C. The Nizhneobskaya culture (MidIrtysh_Nizhneobskaya & Ob_Nizhneobskaya)

The Nizhneobskaya culture was distributed across a vast area of the Western Siberian taiga, predominantly along the main river artery, the Ob River, and its tributaries (the Tobol, Ishim, and Irtysh Rivers). Its northern limits extended to the Arctic Ocean, while the southern boundaries reached as far as the northern edge of the forest-steppe. The distinct identification

of this culture was accomplished by V.N. Chernytsov in the mid-20th century during fieldwork at the Us-Told settlement. The Nizhneobskaya culture existed from the 4th to the 13th centuries CE and evolved through several phases:

1. Karym, 4th–6th centuries CE
2. Zelenogorsk-Ryolka, 6th–8th centuries CE
3. Kuchiminskaya, 8th–9th centuries CE
4. Kintus, 9th–13th centuries CE

Karym phase of the Nizhneobskaya culture (4th–6th centuries CE) is described based on necropolises such as Kozlov-Mys, Krasnoyarsky-4, Alekseevka-50, and Alekseevka-51, characterizing the Migration period in Western Siberia. The population of these sites resided on the periphery of the forest-steppe region inhabited by the Bakalda archaeological culture. The carriers of Karym traditions engaged in communication and exchange with the Ural population. This interaction is evidenced by the substantial quantity of imported items of Eastern European origin, such as belt sets, bronze bowls, and others.

It is reasonably inferred that the migration of Karym groups from the taiga zone to the south took place between the 3rd and 6th centuries CE. During this period, characteristic objects associated with these groups, such as pottery and ornaments, began to appear in the forest-steppe and subtaiga sites. However, this influx of population was not protracted and ceased by the 7th century CE. Moreover, this study indicates that there is no basis for positing substantial migrations of southern or eastern groups into the Lower Tobol River territory during the 3rd–6th centuries CE, as evidenced by archaeological materials (such as the emergence of a superstrate nomadic component).

Anthropological research on cranial series from the Bakalda and Karym cultural contexts in the Lower Tobol River region (burial sites Ipkul', Revda-5, Kozlov-Mys, Pereyma, and Ustyug-1), dated to the 3rd–6th centuries CE, has revealed that these populations exhibited a mixed Eurasian-Mongoloid physical type. A portion of the group was identified as descendants of the Sargatka-associated population, displaying a predominance of Europoid features, while another part aligns with inhabitants of the Western Siberian taiga.

In this study, we included Karym phase Nizhneobskaya population samples from the Ust-Tara-7 burial site, located in the Omsk Trans-Irtysh Region. Regrettably, the cranial remains from the Ust-Tara-7 burial site, due to their poor preservation, were not studied by means of physical anthropology (Borzunov, Chemiakin, 2014).

Kuchiminskaya Phase of the Nizhneobskaya Culture (8th–9th centuries CE).

The period spanning the 8th to 9th centuries CE in the forested northern zone of Western Siberia is characterized by complexes attributed to the Kuchiminskaya phase of the Nizhneobskaya culture. During this phase, main cultural markers (housing construction, fortifications, and pottery production) of the Nizhneobskaya assemblage were standardized, and a large number of imported objects, including those of long-distance origin, indicated a sharp increase in trade and exchange networks. Notably, Iranian bronze vessels were widely distributed in the region (Chemyakin, Karacharov, 2002).

The typical traits of the Kuchiminskaya phase disappeared rapidly in the latter third of the 9th century. During this period, magnificent fortifications were abandoned, pottery traditions underwent transformation, and there was an overwhelming dominance of imports from the west. These changes likely reflected dramatic political and economic transformations that occurred in the Cis-Urals and the Middle Volga regions, where the Volga Bulgaria was established.

The results of physical anthropological examination of the Kuchiminskaya phase population interred at Barsov Gorodok show its similarity to the modern Ugric-speaking population groups. However, comparative data from the middle taiga of Western Siberia for earlier medieval periods are currently lacking, complicating the identification of possible ancestral paleopopulations. It is worth noting that the series from the Barsovo burial site includes several individuals who, alongside the common characteristics shared by the entire group, exhibit certain Europoid traits that are clearly not of local origin. The closest similarities for this subseries were found in the Cis-Urals, particularly with series from the Mitino, Demyonki (6th–8th centuries CE), and Birsk-2 (3rd–7th centuries CE) burial sites. This may reflect a migration of a small population group from the Cis-Ural region into the middle taiga of Western Siberia sometime before 700 CE. However, our study examined not the migrants themselves but their descendants who intermixed with the local population. There are substantial arguments supporting the notion that this migration was a brief and momentary impulse, which did not greatly impact the population of the middle taiga during the medieval period. A comparison between the Barsov Gorodok individuals and contemporary indigenous populations leaves little doubt that they are the direct ancestors of certain Eastern Khanty groups, particularly those along the Salym and Balyk Rivers (Poshekhonova, 2010; Poshekhonova, Sleptsova, 2017).

In our study, the population attributed to the Kuchiminskaya phase of the Nizhneobskaya culture is represented by two individuals from the Barsov Gorodok cemetery. Regrettably, they were not examined anthropologically due to their poor preservation.

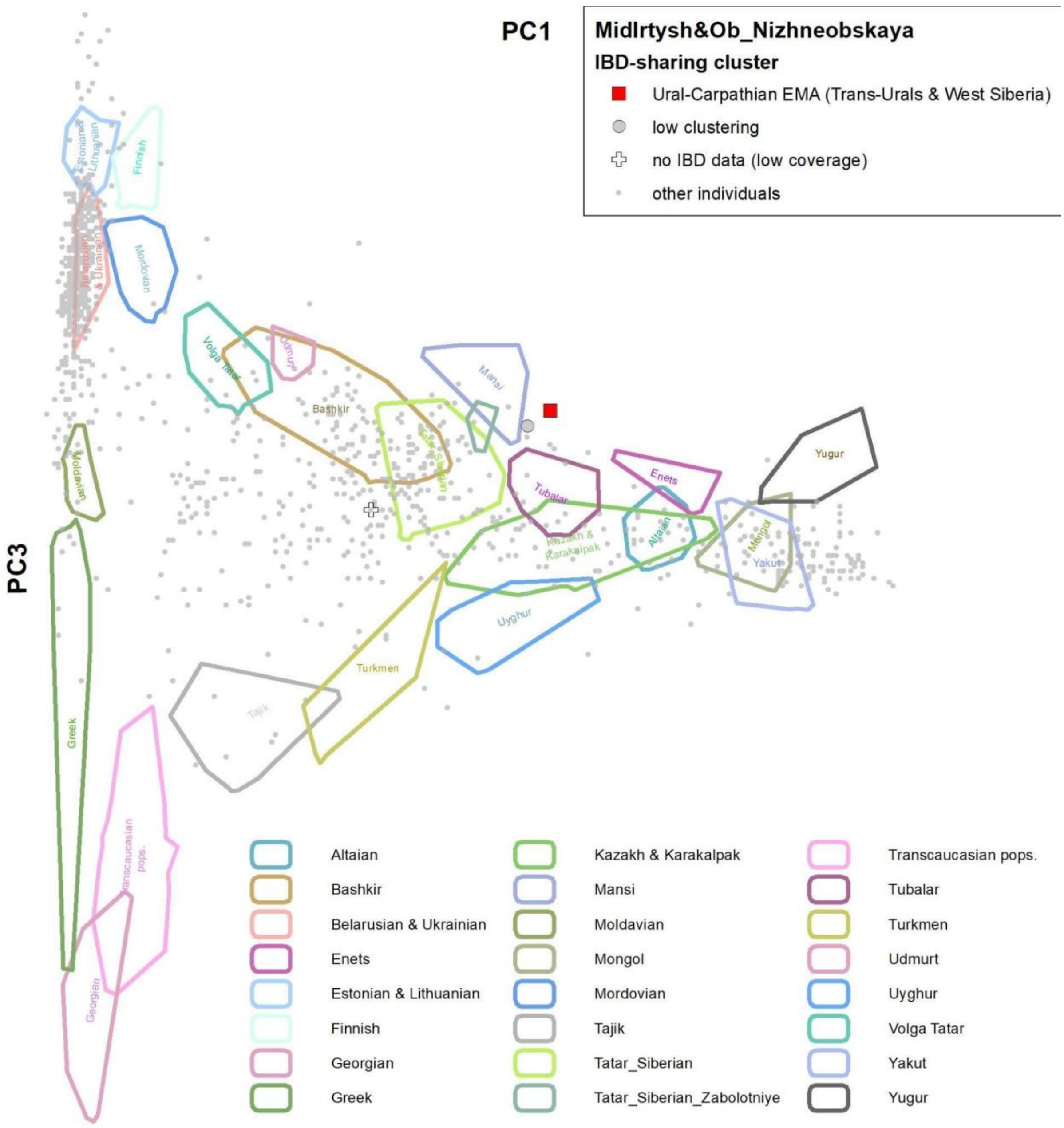


***Figure SIV.5.*** *Eurasian-scale principal component analysis (PCA), with a projection of the newly sequenced individuals onto modern genetic variation following Jeong et al. 2019. The PC1 and PC3 dimensions are shown, including the newly presented genomes. Polygons represent example populations from modern Eurasian individuals onto which the ancient samples were projected, while grey dots indicate ancient reference samples from our IBD-sharing network.*

**Genetic results.** *In the Ob_Nizhneobskaya group, we analyzed two individuals from Barsov-Gorodok (n=2). Both had sufficient coverage for downstream population genetic modeling (>100,000 SNPs) and were suitable for IBD-sharing analysis (>600,000 SNPs). No close relatedness was detected among these individuals, and the female-to-male ratio was 1:1. The samples presented the highest level of Yakutia_LNBA ancestry in the entire analyzed dataset. One sample clustered within the Urals-Carpathian EMA. The high level of Yakutia_LNBA ancestry, combined with the connection to the Urals-Carpathian cluster, suggests that there*

*may have been a connection or common ancestry between the Karayakupovo Horizon individuals and the Migration Period forest zone groups from Western Siberia.*

*In the MidIrtysh_Nizhneobskaya group, we analyzed one individual from the site of Ust-Tara-7 (n=1). This sample had sufficient coverage for downstream population genetic modeling (>100,000 SNPs) but not for IBD-sharing analysis (>600,000 SNPs). The supervised ADMIXTURE analysis revealed elevated levels of Samara_EBA_Yamnaya and Baikal_N ancestry. Compared to the Late Sargatka Mid-Irtysh group, this individual showed a unique combination of steppe-related and Eastern Eurasian steppe ancestry.*

## IV.C.1. Ust-Tara-7

The Ust-Tara-7 burial site is situated in the southern taiga zone of the Irtysh region, within the Tarsky District of the Omsk Oblast. Comprising eight burial mounds with heights of up to 0.5 meters, the site was excavated by I.E. Skandakov between 1990 and 1994 (Skandakov, Danchenko, 1999), with the subsequent publication of materials from the burial ground in 1999. The necropolis is tentatively dated to the 4th–5th centuries CE.

Analysis of the material culture, anthropology, and burial customs indicates sustained interactions between the local population and the tribes of the forest-steppe of Western Siberia and the central Kazakhstan steppes. Notably, distinct cultural traits differentiate the Ust'-Tara assemblage from the taiga-based Nizhneobskaya culture. We identify this site as a specific Karym-type tradition and propose its evolutionary trajectory leading to the later Potchevash cultural complex.

### *mound 9, burial 1 (individual ID I19098, Female)*

The burial was performed in an oval pit elongated from north to south, with a length and width of 3 x 1.9 m, respectively. The southern half of the pit has a depth of 0.72 m and a flat bottom, which gradually rises near the northern wall, forming a step-like feature approximately 0.25 m high. During the excavation of the fill in the northern part of the grave, fragments of charred wood were cleared, with the largest pieces measuring 24-36 cm in length, 8-12 cm in width, and 2-3 cm in thickness.

The skeleton of a female, approximately 20 years old, was positioned elongated on her back, with the head to the south. Due to the profiling of the pit's bottom, the lower part of the shin bones and the heels of the deceased were positioned at the edge of the step, and a clay vessel was placed near the end of the left tibia. Remains of charred vertical stakes, with a diameter of 4 cm, were also cleared around the feet. A bronze buckle with remnants of a leather belt was found near the left thigh, with a piece of charred birch bark lying on top of it. Among other findings, attention is drawn to a bronze necklace (?), consisting of four plates connected by overlapping iron rivets. To the left of the skull, which exhibited signs of artificial deformation, a wire temple pendant was discovered, while another pendant was found beneath the skull, where one end of the necklace was inserted. Small pieces of charcoal and charred birch bark were also present in this area.

The determined radiocarbon age of the burial is 124-311 cal CE (1840±30 BP, Poz-136243) (based on human bone).

## IV.C.2. Barsov Gorodok

The burial site of Barsov Gorodok is located at a locality called "Barsova Gora" in the Khanty-Mansi Autonomous Okrug - Yugra, in the vicinity of Surgut city. Geographically, it is situated in the Surgut Lowland, the northern part of the Middle Ob Lowland, which is located in the central part of the West Siberian Plain (Chemyakin, Zykov, 2004). The area of Barsova Gora, where the burial site is located, represents a section of a high-rooted terrace that borders the right-bank floodplain of the Ob River. The height of the terrace in this area reaches 40 meters, which explains its name "gora" (mountain).

The predominant natural vegetation consists of pine with some Siberian pine and birch, while lichens and taiga grasses dominate the ground cover, along with various shrubs such as bog bilberry, lingonberry, and blueberry.

In the territory occupied by the Barsov Gorodok burial site, various groups of burials are referred to as different phases of the site. Barsov Gorodok-1 is the term for the Medieval graves, while burials from the Early Iron Age are attributed to Barsov Gorodok-3. The Medieval burials (Barsov Gorodok-1) date back to no earlier than 675-700 CE, while the latest ones are no younger than 1300 CE (Karacharov, 2004).

Burials 235 and 236 were excavated in 1989 by an archaeological expedition of the Ural State University. Both burials had been looted in the past, but they were positioned inside the group of graves that date to the 8th-9th centuries CE. The dating of the preserved artifacts from burials 235 and 236 does not contradict the overall dating of the group.

### *burial 235 (individual ID I19095, Male)*

The burial pit, oriented along the west-east axis, had a subrectangular shape with rounded corners and a depth of 11-12 cm from the ground level. The western end of the pit was tapered. The width of the burial pit was approximately 50 cm, and the length was approximately 230 cm. The skeletal remains of a man, aged 20-25 years, were positioned with the skull facing the east on the dorsal surface, in such a way that there was some distance between the skull and the foot bones and the western and eastern walls of the pit (20 and 50 cm, respectively). The limb bones were aligned along the long axis of the pit, with the hands resting on the bottom of the grave next to large spits. Based on their position, the body was laid in the grave without any fixation. The skull lay with its facial surface upward, resting on the occipital and parietal bones. The position of the mandibula is unknown. The available skeletal remains were in anatomical order. At the time of excavation, the shin and foot bones, the right pelvic bone, and all the bones of the right upper limb were missing. The bones of the left arm were represented by decay, except for the distal ends of the radius and ulna, as well as the metacarpal and wrist bones. No traces of the burial construction were preserved. Between the skull and the eastern wall of the pit, a silver plate with protuberances was cleared, and remnants of an iron object (possibly a chisel) were found beneath the skull. Around the skull and near the left knee joint, eight silver appliqués were discovered. It can be assumed that these silver items have a Cis-Uralic origin.

The determined radiocarbon age of the burial is 131-336 cal CE (1810±30 BP, Poz-136239) (based on human bone). Due to archaeological reasons, the date is irrelevant and likely biased by the freshwater reservoir effect.

### *burial 236 (individual ID I19096, Female)*

The burial pit was oriented along the northeast-southwest axis and had a subrectangular shape with rounded corners, with a depth of 10-11 cm from the ground level. The southwest end of the pit was tapered. The width of the burial pit was approximately 38 cm, and the length was approximately 190 cm, nearly matching the height of the interred individual. The skeletal remains of a woman, aged 45-50 years, were positioned with the skull facing the northeast. The limb bones were initially aligned along the long axis of the pit. Based on their position, the body was laid in the grave without any fixation. However, during the looting of the burial, the left femur became dislocated, and the other leg bones were damaged. Only the shoulder bones of the arms were preserved. The skull, rotated to the left, rested on the base and basal surface of the mandibula. The remaining skeletal remains were in anatomical order, except for the left femur. No traces of the burial construction were preserved. Near the left half of the mandibula, a small ceramic vessel was cleared, and a large iron knife was placed southwest of the non-preserved bones of the right forearm. A silver appliqué, similar to the previous one, was found in the abdominal area of the interred individual. A fragment of pottery was discovered in the vicinity of the non-preserved foot bones.

The determined radiocarbon age of the burial is 261-535 cal CE (1655±30 BP, Poz-136240) (based on human bone).

## IV.D. The Potschevash culture (MidIrtysh_Potchevash)

The sites attributed to the Potchevash culture are primarily distributed in the southern taiga and forest-steppe regions along the valleys of the Ishim and Irtysh rivers, encompassing the Tyumen, Omsk, and Novosibirsk regions. The identification of this culture can be credited to V.N. Moshinsky, who based his classification on the assemblage of sites located at Chuvashsky Mys, near the city of Tobolsk, during the mid-20th century. Dated to the 6th-8th centuries CE, the Potchevash culture exhibits a heterogeneous character, incorporating elements from the Kulay, Sargatka, Nizhneobskaya, and Bakalda cultures, as well as early Turkic traditions, reflecting the cultural admixture of its population.

**Genetic results.** *In this group, we analyzed one individual from the site of Vikulovo (n=1). This sample had sufficient coverage for downstream population genetic modeling (>100,000 SNPs) and was suitable for IBD-sharing analysis (>600,000 SNPs). The supervised ADMIXTURE analysis revealed elevated levels of Samara_EBA_Yamnaya and Altai_N ancestries. The sample clustered within the Urals-Carpathian EMA cluster. His genetic composition was similar to the Late Sargatka group in the region.*

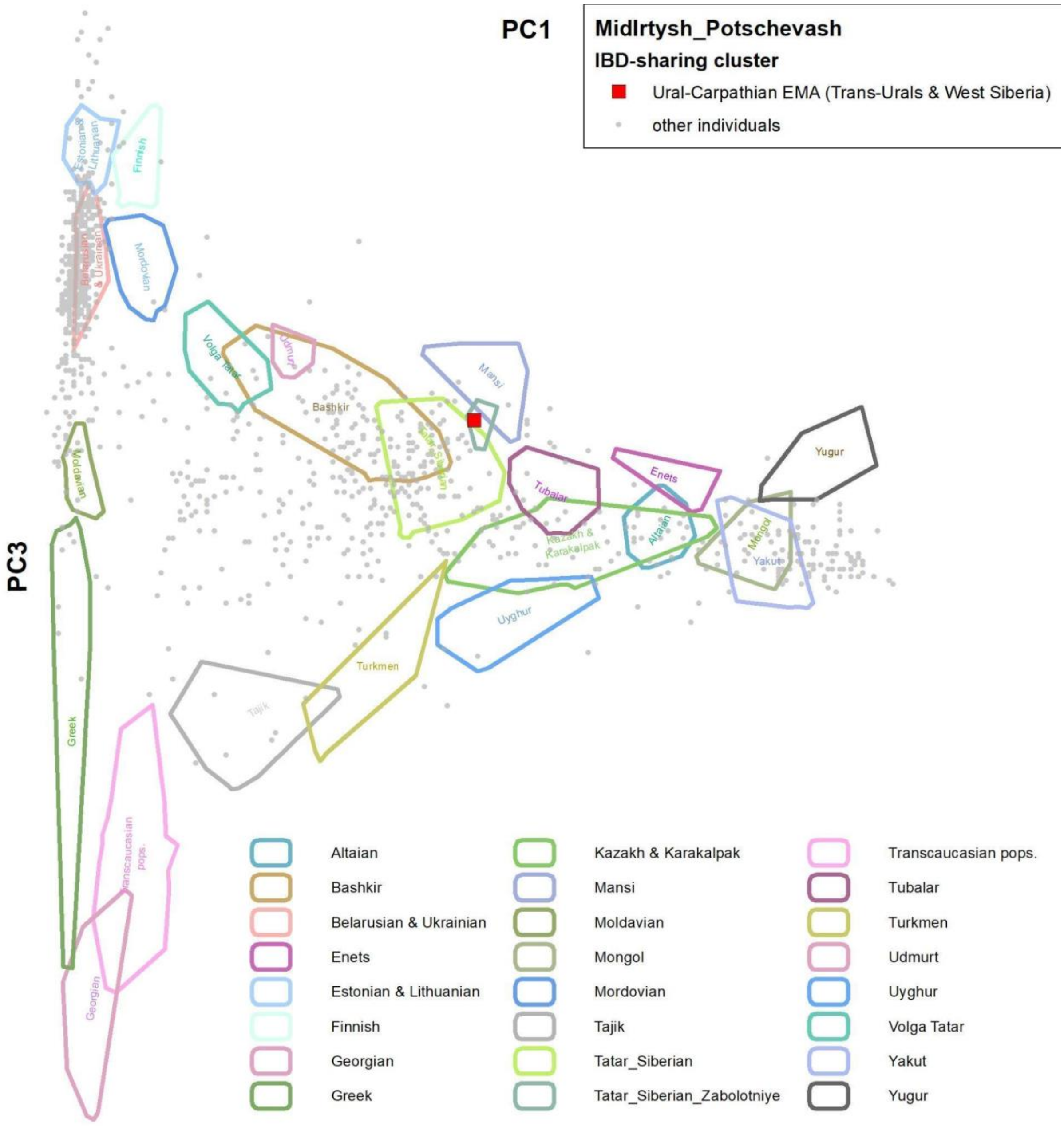


***Figure SIV.6.*** *Eurasian-scale principal component analysis (PCA), with a projection of the newly sequenced individuals onto modern genetic variation following Jeong et al. 2019. The PC1 and PC3 dimensions are shown, including the newly presented genomes. Polygons represent example populations from modern Eurasian individuals onto which the ancient samples were projected, while grey dots indicate ancient reference samples from our IBD-sharing network.*

## IV.D.1. Vikulovo

The burial ground of Vikulovo Cemetery is situated in the western part of a ridge-shaped outcrop that extends from west to east on the terrace of the Ishim River. The cemetery was excavated by Viktoria Ilyushina in 2008.

### *collective burial 1*

The burial was situated in the eastern sector of the excavation area. It consisted of a sub-rectangular grave pit with rounded corners, discovered at a depth of approximately 0.6 m from the present-day surface. The vault of the skull and clavicle were located near the northern wall of the grave, while the tibia was found near the southern wall. The grave itself measured 1.75 × 2.0 m in size, with a depth ranging from 0.04 to 0.08 m from the mainland level. Its orientation followed a north to south axis. Three pits were observed on the surface of the burial fill, likely the result of later disturbances unrelated to the original burial event. Within the grave, the remains of an estimated four individuals were discovered. The cultural layer of the archaeological site primarily consisted of loam; however, the condition of the bones was poor, likely due to disturbance caused by subsequent construction activities in the Russian period (Ilyushina, 2009).

Skeleton 1 (individual ID I19094, Male) (referred to as "skeleton 2" in the publication) was located near the western wall of the burial. The skeletal remains belonged to an adult male, aged approximately 35-40 years at the time of death. It is presumed that the individual was originally placed in a supine position, with the head oriented towards the north. The mandibula was damaged, the skull fragmented, and various skeletal elements, including cervical vertebrae, collarbones, ulna, humerus, radius, femur, and a fragment of the pelvic bone, were preserved. However, due to disturbances, it is not possible to determine the original body position during burial (Ilyushina, 2009). Of particular interest is a significant chop wound observed on the parietal and occipital bones of the male individual, suggesting a potential warrior status (unpublished data from the author).

The determined radiocarbon age of the burial is 27-213 cal CE (1915±30 BP, Poz-136238) (based on human bone). The radiocarbon date contradicts artefactual data and likely was bias due to freshwater reservoir effect.

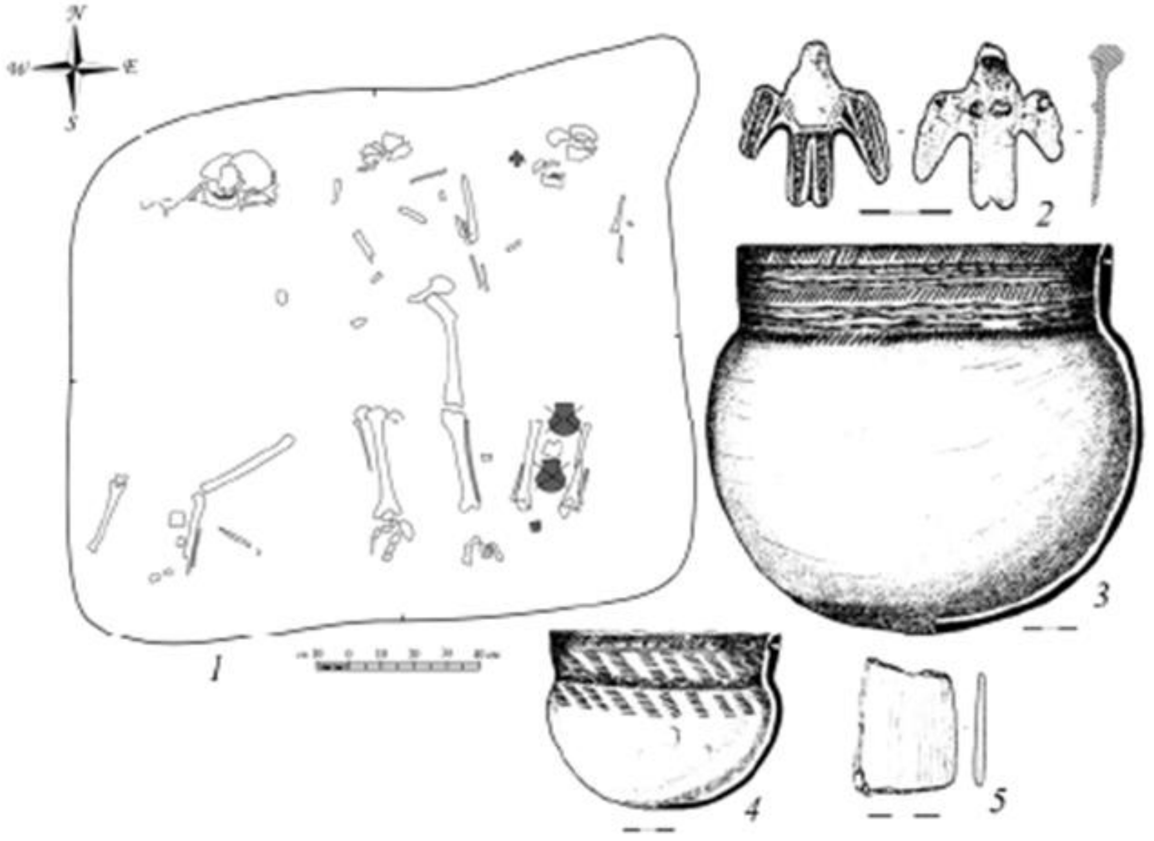


***Figure SIV.7.*** *Vikulovo burial site, collective burial 1. 1 - a plan of the burial, 2-5 - grave goods.*

## <u>IV.E. The sites of the Karayakupovo Horizon in the Trans-Urals (Trans-Urals_KH)</u>

The Karayakupovo horizon in the Southern Trans-Urals evolves in three chronological phases.

**<u>Phase 1 (second half of the 8th century).</u>** This phase is marked by the appearance of burial sites (Lagerovo, Bayramgulovo) in the forest-steppe foothills of the Southern Urals, which are not connected to preceding cultures, such as the Turbasy culture or the so-called "kurgans with moustaches". The only known site in the mountainous forested Trans-Urals, Bayramgulovo, contained duck-shaped pendants and heraldic-style belt elements. This site also yielded entirely new forms and elements of weaponry for the Trans-Urals, including chainmail, sabers, and plates of composite bows. Key sites from this period include the Cis-Ural complexes of Lagerovo (early phase), Manyak, Novo-Bikkino, Bulgar, and Tavlykaevo kurgans.

**<u>Phase 2 (late 8th – mid-9th century).</u>** During this phase, the specific traits of the material culture of the Karayakupovo Horizon became more prominent and the number of sites increased, which likely reflects consolidation of nomadic groups in the Southern Urals from the previous phase. Among the new burial sites, Husainovo, Bekeshevo, Graultry, Yamashi-Tau, Uyelgi (early phase), and Narovchat can be listed. A distinctive style characteric for this phase utilizes smooth (non-ornamented) silver belt and bridle plaques. These plaques

retained some features of the earlier styles, such as heraldic belt set compositions, Katanda, and post-Sasanian. Popular forms included rectangular plaques with slits, figurative plaques (portal, heart-shaped, crescent-shaped, etc.) with cutouts or semi-spherical protrusions. The surface design often featured minimalist decorative elements such as a central axial ridge or a dual-pitched division. A unique category of adornments appearing during this time is the bilunula plaques with paired or single semi-spherical protrusions.

**Phase 3 (mid-9th – 10th century).** To this phase, numerous sites belong, including Lagerovo (late phase), Ishimbaevo, Staro-Khalilovo, Idelbaevo kurgans, Sineglazovo, Uyelgi (late horizon), Karanaevo, Murakaevo, and Aktyuba. During this time, a shift in the style of belt decorations occurred, with gilded plaques featuring relief ornaments on the front side becoming popular. These motifs were predominantly plant-based, with some geometric, zoomorphic, or anthropomorphic designs. Elite burial sites from this period (Uyelgi, Aktyuba, Sineglazovo) revealed plaques in the so-called "Hungarian" style, distinguished by borders of alternating ovals and circles. Unlike the previous phase, burials were conducted in shallow pits while retaining general ritual characteristics. It is hypothesized that the introduction of this new style might be influenced by new migrants from the east - early Kipchak groups (possibly of the Srostki culture) from Altai and Eastern Kazakhstan.

**Genetic results.** *In this group, we analyzed eight individuals from the site of Uyelgi (n=8). All had sufficient coverage for downstream population genetic modeling (>100,000 SNPs) and were suitable for IBD-sharing analysis (>600,000 SNPs). No close relatedness was detected among these samples, and the female-to-male ratio was 1:7. The supervised ADMIXTURE analysis revealed elevated levels of Samara_EBA_Yamnaya and Yakutia_LNBA ancestries in this group. The TransUrals_KH samples are clustered within the Urals-Carpathian EMA IBD cluster. These genetic results suggest a strong connection to other Karayakupovo horizon groups with a similar genetic composition to the Migration Period Belaya Kushnarenkovo and Tobol Late Sargatka groups.*

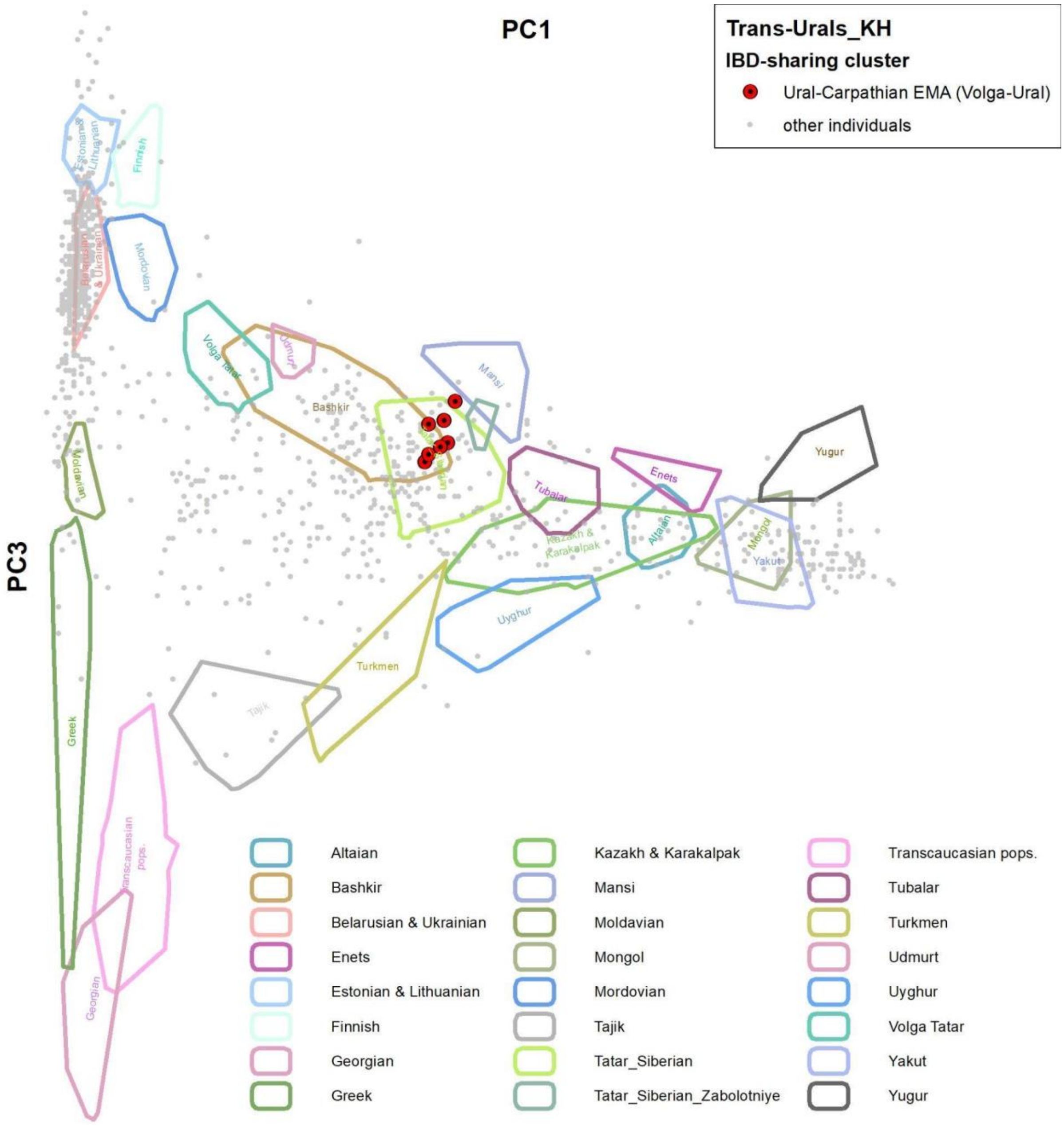


***Figure SIV.8.*** *Eurasian-scale principal component analysis (PCA), with a projection of the newly sequenced individuals onto modern genetic variation following Jeong et al. 2019. The PC1 and PC3 dimensions are shown, including the newly presented genomes. Polygons represent example populations from modern Eurasian individuals onto which the ancient samples were projected, while grey dots indicate ancient reference samples from our IBD-sharing network.*

## IV.E.1. Uyelgi

The Uyelgi kurgans are located in the Southern Urals, at the transition between the forest-steppe and steppe natural zones, in the modern Kunashaksky District of Chelyabinsk Oblast, Russia. The site is situated on a terrace between the Uyelgi and Saigerly lakes. Most of the burials under the mounds were performed using the inhumation rite, with cremations being rare.

The cultural and chronological attribution of the site is based on a distinctive group of artifacts, including:

1. Bekeshev and Husainov-type plaques (9th century),
2. Srostki-type belt sets (second half of the 9th century to the first half of the 11th century),
3. Horse harnesses and weapons dating from the late 8th to the 11th centuries,
4. Pottery of the Kushnarenkovo, Karayakupovo, and Petrogrom types.

The chronological framework is further supported by 13 radiocarbon dates. Combining archaeological and $^{14}C$ data, the site is divided into two chronological phases:

1. 9th century,
2. Late 9th to early 11th centuries.

Typological comparisons refine this range to extend from the late 8th century to the first half of the 10th century.

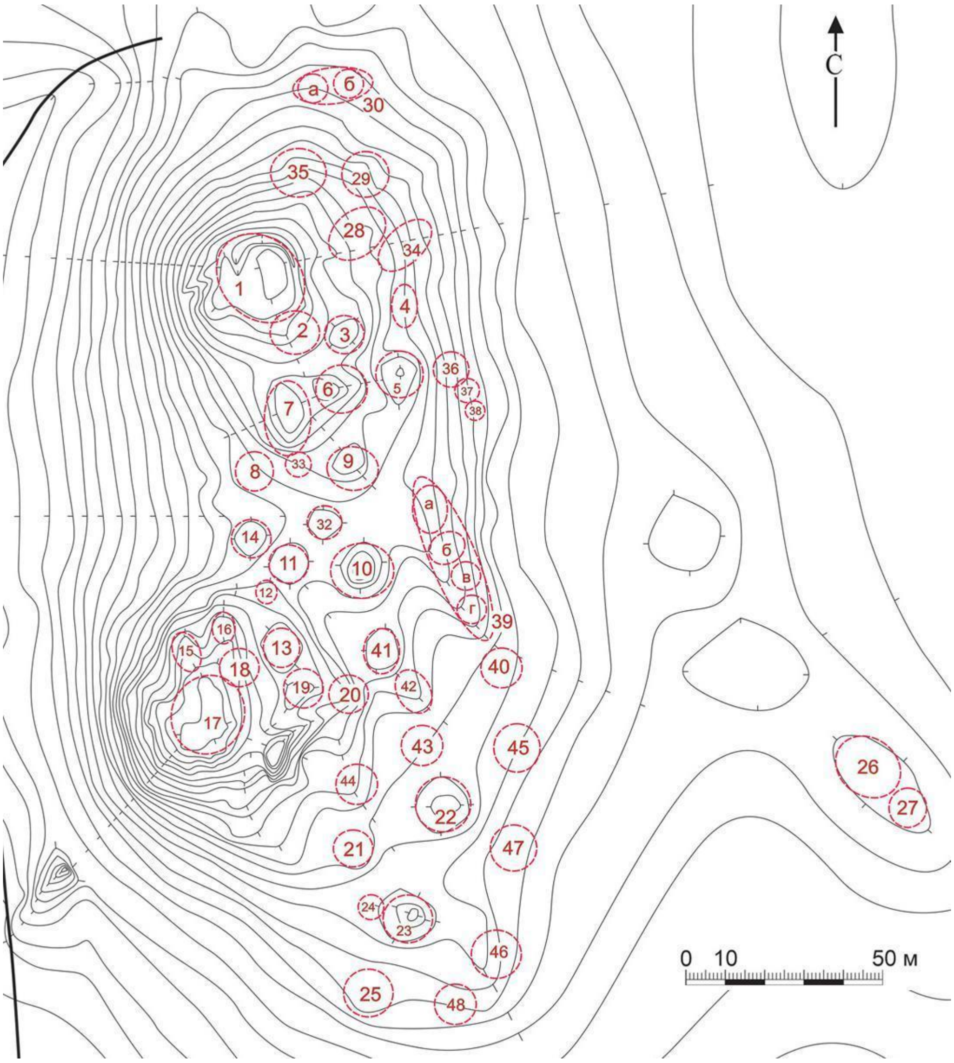


The cultural influence of the Srostki assemblage, evident at Uyelgi in transformation of burial practices and the appearance of gilded, ornamented plaques, suggests a possible influx of a population of Altai origin into the Southern Trans-Urals during the Karayakupovo horizon period, around the mid-9th century.

***Figure SIV.9.*** *A plan of the Uyelgi burial site.*

## *Kurgan 7, burial 1 (individual ID I19121, Male)*

Kurgan 7 was excavated in 2011. Burial 1 (published in Grudochko & Batalov 2013 as burial 5) features a sub-rectangular pit with a depth of 0.15 m, with remnants of a wooden coffin around the perimeter of the burial chamber. The pit contained one individual, laid out supine. Discovered artifacts include hemispherical bridle plaques, belt plaques with gilding and plant motifs characteristic for the Srostki-style ornaments of the Trans-Ural region, silver brackets for the front arch of a saddle, and an earring. The pit had been looted, preserving only fragments of long bones and the skull, making it impossible to determine the age of the interred.

Radiocarbon analysis provided a combined date of 879-1150 cal CE (1053±50, SPB_854; 888-1151 cal CE, 1040±50, SPb_852, 95.4% probability) (Grudochko & Batalov 2013). The burial is attributed to the Srostki-influenced group. The $^{14}$C date does not match archaeological context, which should be attributed to 850-900 CE.

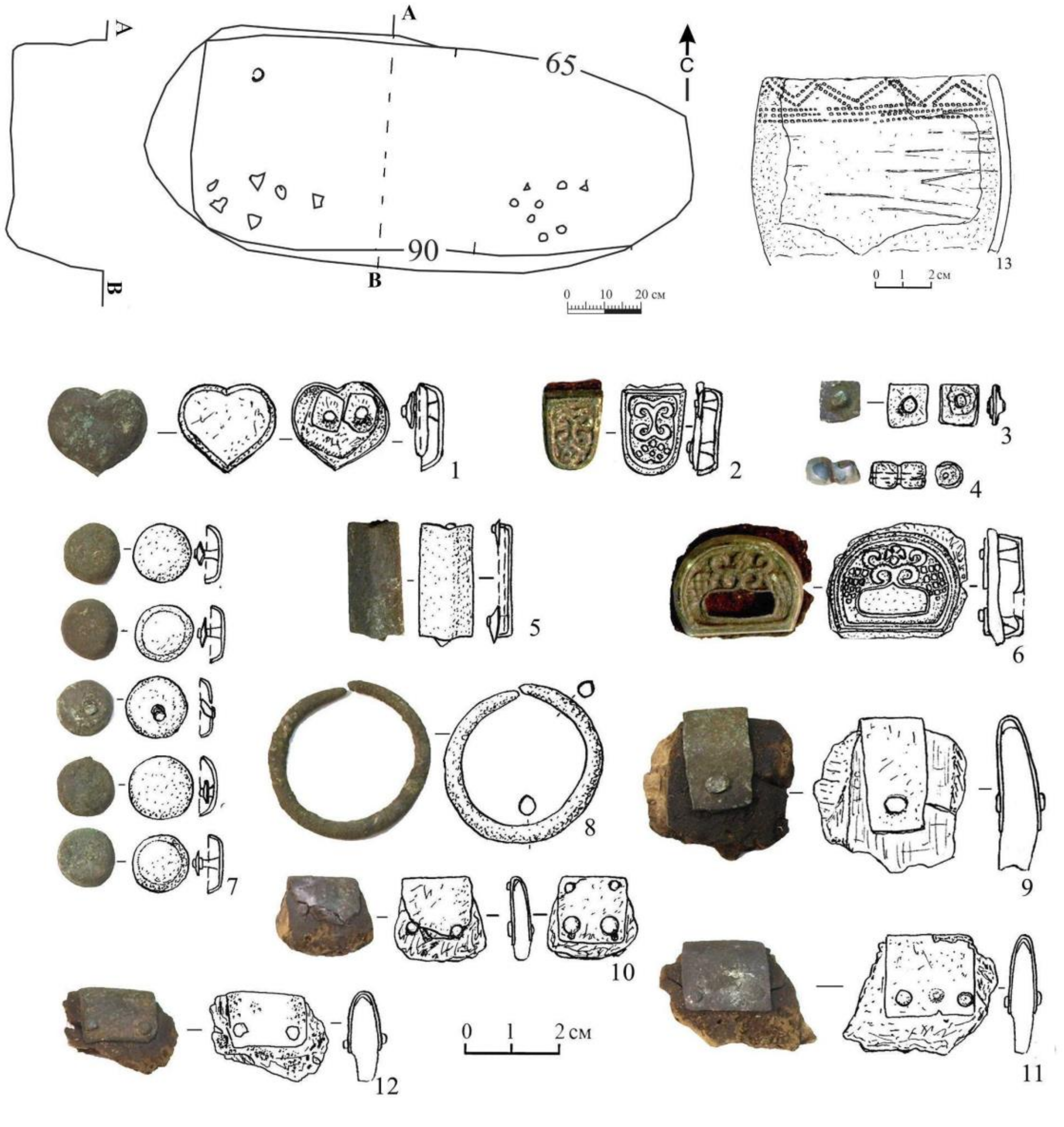


***Figure SIV.10.*** *Artifacts from burial 1 of mound 7. I - burial plan; 1, 3, 5, 8 - silver; 2, 6 - silver, gilding; 9-12 - silver, wood; 13 - ceramics.*

### *Kurgan 9, burial 5 (individual IDs I19115, Male and I19116, Male)*

The grave pit of this burial was severely damaged by looting, making it challenging to accurately reconstruct its original features. The pit's depth was 0.25 m, and its shape has been deformed. Bones are scattered, with many missing, likely belonging to two individuals who were presumably laid out in a supine position. No remains of wood or coffin elements were recorded in the grave. Artifacts recovered include a silver belt tip and Petrogrom-type pottery. Based on the archaeological context, the burial is dated to 800–950 CE. However, a more precise attribution is not possible due to the scarcity of artifacts caused by looting.

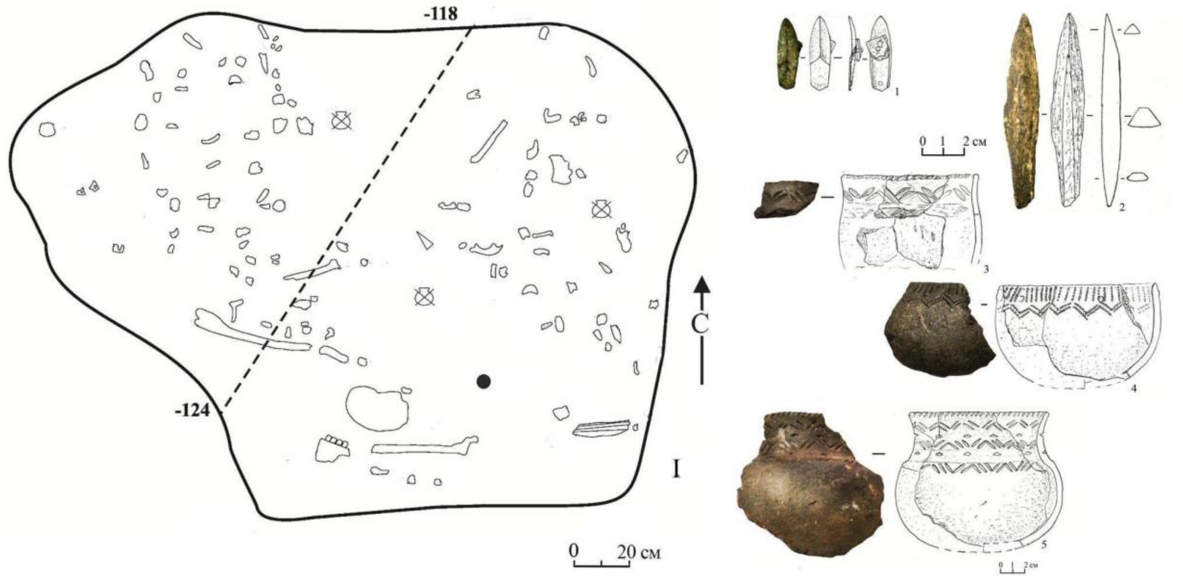


***Figure SIV.11.*** *Image of the burial and artifacts from burial 5 of mound 9. I - burial plan; 1 - silver; 2 - bone; 3-5 - ceramics.*

### *Kurgan 28, burial 5 (individual ID I19114, Female)*

Kurgan 28 was excavated in 2011. Beneath the burial mound, a pit measuring 0.25 m in depth was identified. Initially sub-rectangular in shape, the pit was disturbed in its western section and oriented along the WSW–ENE axis. It contained the remains of a single individual, laid supine with the head positioned towards the WSW.

No burial structures or accompanying artifacts were found within the burial pit itself, aside from four bone arrowheads. However, beneath the burial mound, on the buried soil surface, a set of belt decorations was unearthed. This set included a silver-gilded belt tip and 15 plaques, stylistically associated with Srostki-style artifacts. Based on this similarity, the belt set is attributed to phase 2 of the Uyelgi necropolis.

Stratigraphic observations suggest that the burial and the kurgan mound belong to the same chronological period. Consequently, the burial is dated to 850–950 CE. The kurgan's location in the later part of the burial site further supports this hypothesis.

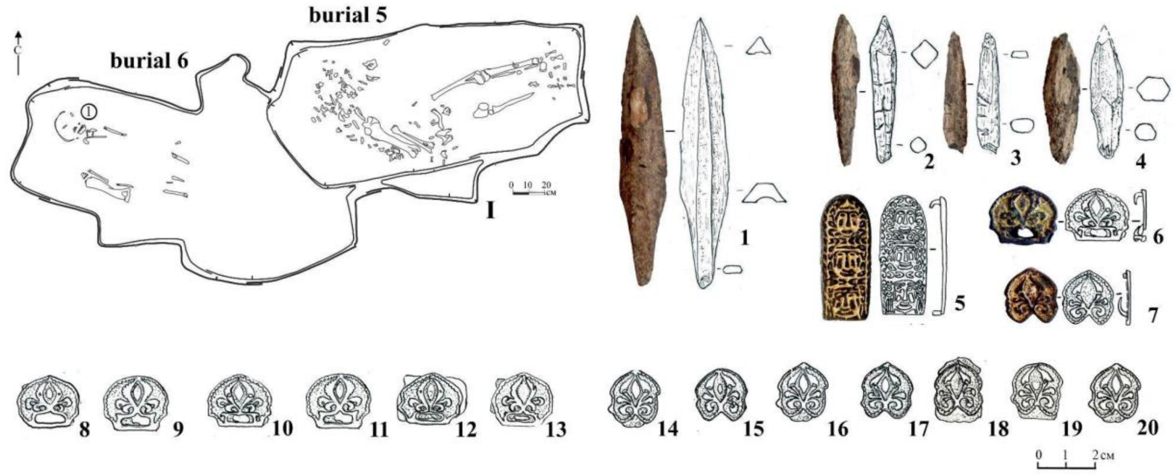


*__Figure SIV.12.__ Image of the burial and artifacts from a sub-mound horizon. I - burial plan; 1-4 - bone; 5-20 - silver, gilding.*

### *Kurgan 29, burials 1 and 2 (disturbed) (individual ID I19118, Male)*

In the central part of the mound, two amorphous pits were discovered, each containing remnants of a wooden structure along the inner perimeter. Based on the size and proportions of the pits, it is assumed that each originally held no fewer than 2–3 individuals. However, due to extensive looting, bones were scattered throughout, and the pits are now treated as a single complex feature. Fragments of skulls were recovered from the western part of the pit, suggesting a western orientation of the bodies, likely laid out in a supine position.

The grave inventory included silver buckles, buttons, overlays, and remnants of an archer's belt. Based on the archaeological context, the burial is dated to 850–900 CE, and radiocarbon analysis provided a calibrated age of 774–883 CE (95.4% probability). The upper chronological limit remains poorly documented. The burial is attributed to the Srostki-influenced group.

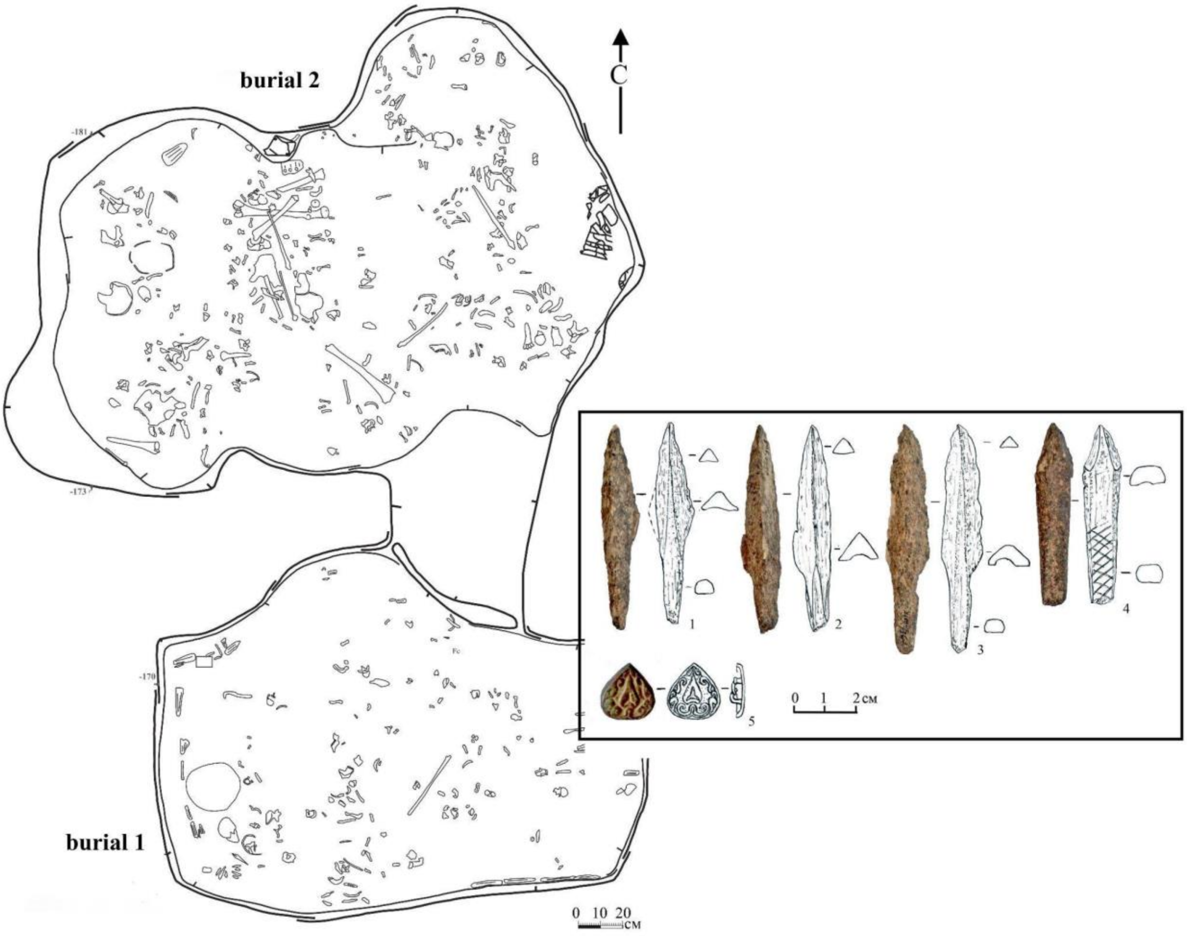


***Figure SIV.13.*** *Kurgan 29. I - burial 1; II - burial 2; 1 - 5 - artifacts from burial 2; 1-4 - bone; 5 - silver, gold plated.*

## *Kurgan 30, infill of the burial mound (individual ID I19119, Male and individual ID I19120, Male)*

Kurgan 30, excavated in 2012. The infill of the mound yielded a rich set of artifacts possibly redeposited from a robbed burial pit. The set of artifacts included bone arrowheads and buckles, belt clips, silver belt buckle, three heavily ornamented gilded silver belt plaques, gilded belt tip, and another belt tip with openwork. Among the artifacts, a human skull was found. According to the archaeological context, the burial dates to 850-950 CE. Based on the artifacts found, the redoposited burial likely belonged to the Srostki-influenced group.

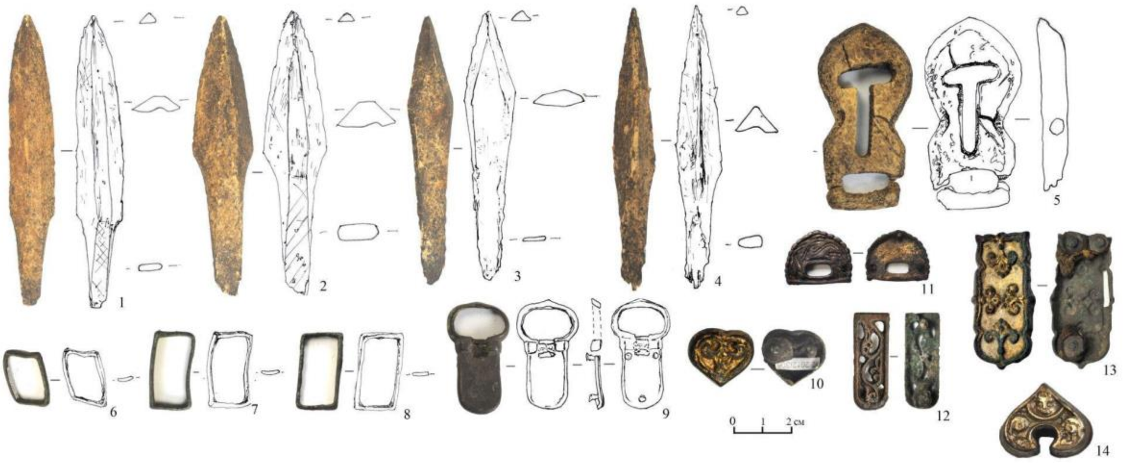

*__Figure SIV.14.__ Artifacts from the mound 30 (underground site). 1-5 – bone; 6-9 – silver; 10-14 – silver, gilding.*

### *Kurgan 32, burial 12 (individual ID I19117, Male)*

The depth of the grave pit is 0.35 m, its shape is sub-triangular, deformed due to looting. The pit contained one individual, likely laid out supine with the head to the west. The age of the interred could not be determined, but it is presumed to be an adult. The inventory of the grave contained a set of artifacts manufactured in the Khusainovo and Bekeshevo styles. According to the archaeological context, the burial dates to 800-850 CE and is likely associated with the group of nomads of the Bekeshevo cultural tradition.

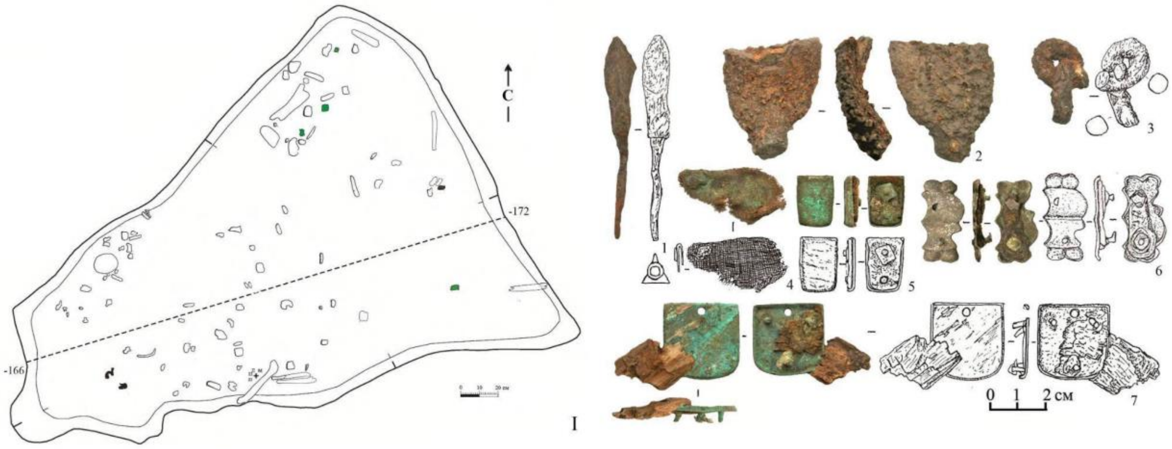

*__Figure SIV.15.__ The image of the burial and artifacts from burial 12 of mound 32. I - burial plan; 1, 2, 3 - iron; 4 - textiles; 5-7 - silver, leather, wood.*

## IV.F. The Medieval Ust'-Ishim culture (MidIrtysh_Ust-Ishim)

In the 1950s, V.N. Chernetsov attributed the medieval sites of the Lower Ob region and the southern taiga of the Omsk portion of the Irtysh River to the Kintus phase of the

Nizhneobskaya culture. However, subsequent investigations in the Omsk part of the Irtysh River led V.A. Mogilnikov (Mogilnikov, 1987) and B.A. Konikov identified a distinct cultural tradition in the region, dating from the 9th to the 13th centuries CE. B.A. Konikov demonstrated that the medieval inhabitants of the Irtysh Basin's southern taiga constructed kurgan burials and based their economy on cattle breeding, in contrast to the fishing-based Nizhneobskaya culture, whose dead were interred in flat burial grounds (Konikov, 2017).

The sites of the Ust-Ishim culture are primarily located in the sub-taiga and southern taiga zones of Western Siberia, spanning from the forest-steppe boundary in the south to the Vasyugan River in the north, and from the Tobol River in the west to the Tara River, a tributary of the Irtysh, in the east. The Ust-Ishim medieval tradition is chronologically framed between 800 and 1240 CE, supported by the presence of imported ornaments from Volga Bulgaria, Old Rus, and the medieval Kama region. The Ust-Ishim culture is believed to be associated with the ancestors of the southern Khanty, who had close interactions with the Turkic-speaking population, likely linked to the Srostki culture. However, physical anthropological studies suggest that the Siberian Tatars are the most probable descendants of the Ust-Ishim groups.

The complete collection of Ust-Ishim anthropological material includes 46 male and 22 female skulls from the Ivanov Mys-1 and Panovo-1 sites, alongside additional samples from the burial grounds of Masarly-1, Nugai-1, and Dolgovsky-1 settlement. This series appears homogeneous, likely representing a single paleopopulation resembling groups buried in the forest-steppe variant of the Kulai cultural context and the West Siberian Mongoloids associated with the Sargatka tradition burials. Among contemporary ethnic groups in Western Siberia, the Ust-Ishim people are closest in anthropological type to the Tobol-Irtysh Tatars, particularly the Tyumen and Kourdak-Sargatka groups, who are considered their direct descendants.

Slight variability in the skull series from Ivanov Mys-1 and Panovo-1 may indicate the presence of a small immigrant population and their descendants. The closest parallels for this group are found at sites linked to the Srostki and Basandaika cultures, such as the Sanatorny-1 burial ground (1000–1250 CE) and the Gilevo burial ground (700–1000 CE). Migration from South Siberia to the southern taiga of the Irtysh Basin ceased in the 14th–16th centuries (Poshekhonova, 2011).

**Genetic results.** *In this group, we analyzed eight individuals from the sites of Ivanov Mys-1 (n=5) and Panovo (n=3). All eight had sufficient coverage for downstream population genetic modeling (>100,000 SNPs), and six were suitable for IBD-sharing analysis (>600,000 SNPs). The individuals I19087 and I19090 from the Ivanov Mys-1 site are first-degree relatives. In this group the female-to-male ratio was 3:5. The samples displayed a genetic composition similar to the MidIrtysh_Sargatka group. The supervised ADMIXTURE analysis revealed elevated levels of Samara_EBA_Yamnaya and Yakutia_LNBA ancestries. The MidIrtysh_Ust-Ishim samples are clustered within the Urals-Carpathian EMA cluster, but in a distinct positioning.*

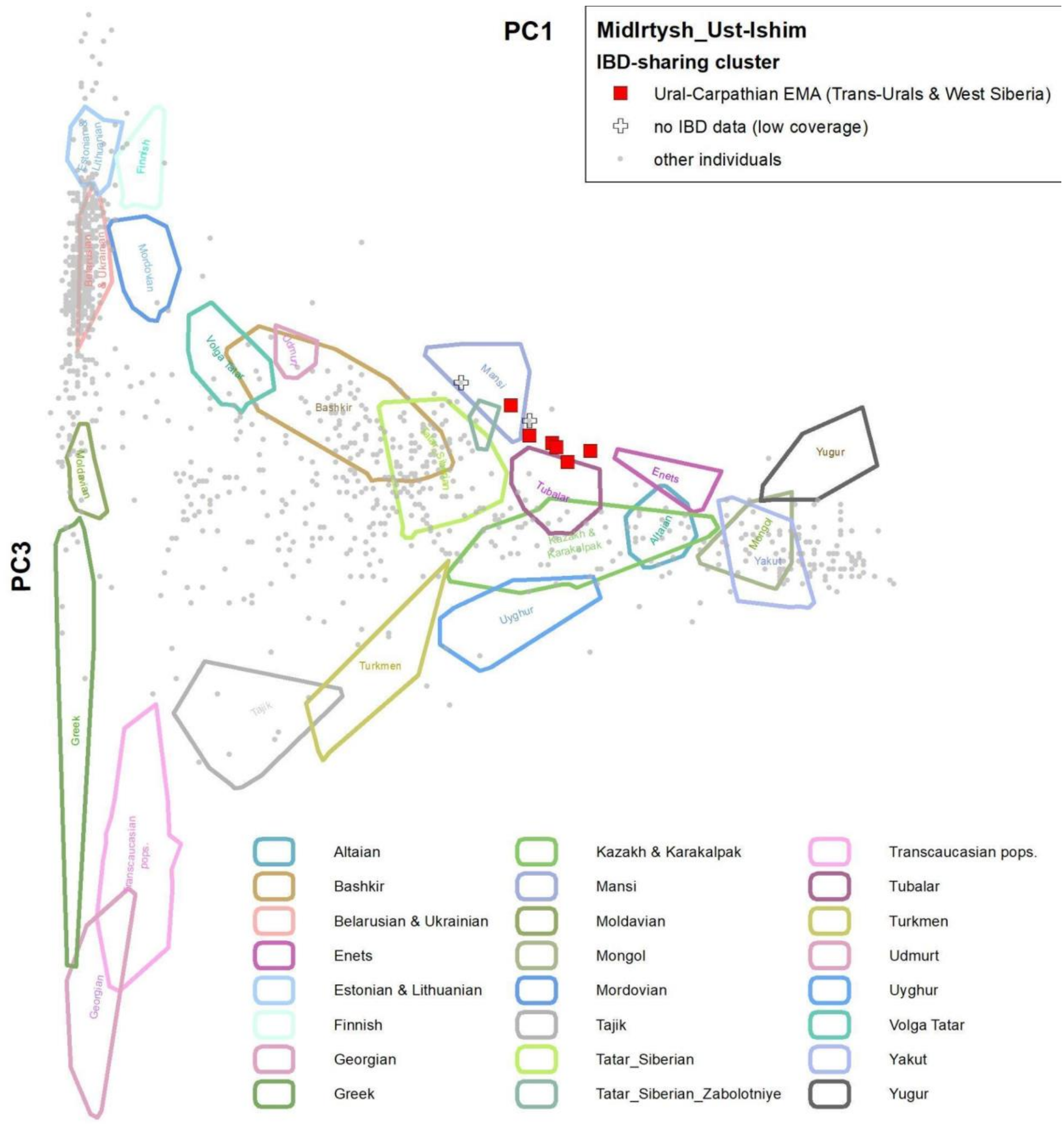


***Figure SIV.16.*** *Eurasian-scale principal component analysis (PCA), with a projection of the newly sequenced individuals onto modern genetic variation following Jeong et al. 2019. The PC1 and PC3 dimensions are shown, including the newly presented genomes. Polygons represent example populations from modern Eurasian individuals onto which the ancient samples were projected, while grey dots indicate ancient reference samples from our IBD-sharing network.*

## IV.F.1. Ivanov Mys-1

The Ivanov Mys-1 burial site is located near the village of Ivanov Mys, Tevriz District, Omsk region, 300 km north of Omsk, and consists of more than 50 burial mounds up to 14 m in diameter and 1 m high.The burial site dates back to the 13th century CE. We sequenced 3 individuals from under mound 10, one - from under mound 12, and one individual from unidentified burial (individual ID I19091, Male). This last individual was dated by $^{14}$C, and its

age was determined as 416-545 cal CE (1600±30 BP, Poz-136215), which is likely biased by freshwater reservoir effect (Konikov, 2019).

### *mound 10, burial 3 (individual ID I19087, Male)*

The burial was located in the north-east part of the mound, in a rectangular pit oriented northwest-south-east. The size of the grave was 2.15 x 1.25 m, the walls were steep, and the corners were smoothly rounded. At the bottom of the grave rested two dead people stretched out on their backs. Both bodies were oriented with their heads to the northwest. In the southeast corners, two clay vessels stood upside down, one of which was placed on the grave slab and the other on the bottom of the pit. Fragments of wooden planks and birch bark cloth were preserved on the skeletons. On the man's head there was a bronze half-tambourine, and on the sides there was one lapis lazuli figurative pendant, on the inner side of the left radial bone of the skeleton there was a single-bladed iron knife. Between the skulls there was a bronze chime.

The determined radiocarbon age of the burial is 1165-1267 cal CE (835±30 BP, Poz-136210) (based on human bone).

### *mound 10, burial 6 (individual ID I19088, Male)*

The disturbed burial was found in the western part of the mound. The skull was lying with the parietal bone to the north-north-west facial bones upwards. To the west of it, on its side, with its mouth to the north-west, was a clay bowl-shaped vessel, under which a bone petiole arrowhead lay.

The determined radiocarbon age of the burial is 250-411 cal CE (1720±30 BP, Poz-136211) (based on human bone). Due to archaeological reasons, the date is irrelevant and likely biased by the freshwater reservoir effect.

### *mound 10, burial 9 (individual ID I19089, Male)*

The determined radiocarbon age of the burial is 356-57 cal BCE (2160±30 BP Poz-136212) (based on human bone). Due to archaeological reasons, the date is irrelevant and likely biased by the freshwater reservoir effect.

### *mound 12, burial 1 (individual ID I19090, Female)*

The burial was located in a central rectangular continental pit, oriented northwest-south-east. It was robbed. The walls are steep, the corners are rounded, and the bottom is flat. The skeleton is disturbed. The deceased was stretched out on his back with his head to the northwest. An iron flat petiole arrowhead lay to the corner of the skull. Between the tibiae was an oval wooden object 7 cm in diameter. A broken bone ornamented plate lay parallel to the tibia. In the southern part of the grave fragments of plaques were discovered. A single-bladed knife in a wooden scabbard, a bronze lunette, and bone ornamented plates were placed in the grave.

### IV.F.2. Panovo

The Panovo-1 burial mound is located 4.5 km southwest of the Panovo village, Ust-Ishim region, Omsk Region, on the first supra-flood terrace of the right bank of the Irtysh River. It consists of 77 embankments up to 20 m in diameter and 1.2 m high.

#### *burial 1 (individual ID I19097, Female)*

The determined radiocarbon age of the burial is 210-353 cal CE (1785±30 BP, Poz-136241) (based on human bone). Due to archaeological reasons, the date is irrelevant and likely biased by the freshwater reservoir effect.

#### *mound 1, burial 30 (individual ID I19092, Female)*

The determined radiocarbon age of the burial is 641-775 cal CE (1350±30 BP, Poz-136236) (based on human bone). Due to archaeological reasons, the date is irrelevant and likely biased by the freshwater reservoir effect.

#### *mound 67, burial 3 (individual ID I19093, Male)*

The determined radiocarbon age of the burial is 647-775 cal CE (1335±30 BP, Poz-136237) (based on human bone). Due to archaeological reasons, the date is irrelevant and likely biased by the freshwater reservoir effect.

## IV.G. The Late Medieval burials (Tobol_Tatars)

**Genetic results.** *In this group, we analyzed one individual from the site of Putilovo (n=1). This sample had sufficient coverage for downstream population genetic modeling (>100,000 SNPs) and was suitable for IBD-sharing analysis (>600,000 SNPs). This male shows Eastern Eurasian steppe genetic affinity. The supervised ADMIXTURE analysis revealed elevated levels of Samara_EBA_Yamnaya and Baikal_N ancestries in this group. This male individual clustered within the Urals-Carpathian EMA cluster. However, this individual was not a subject for our analysis.*

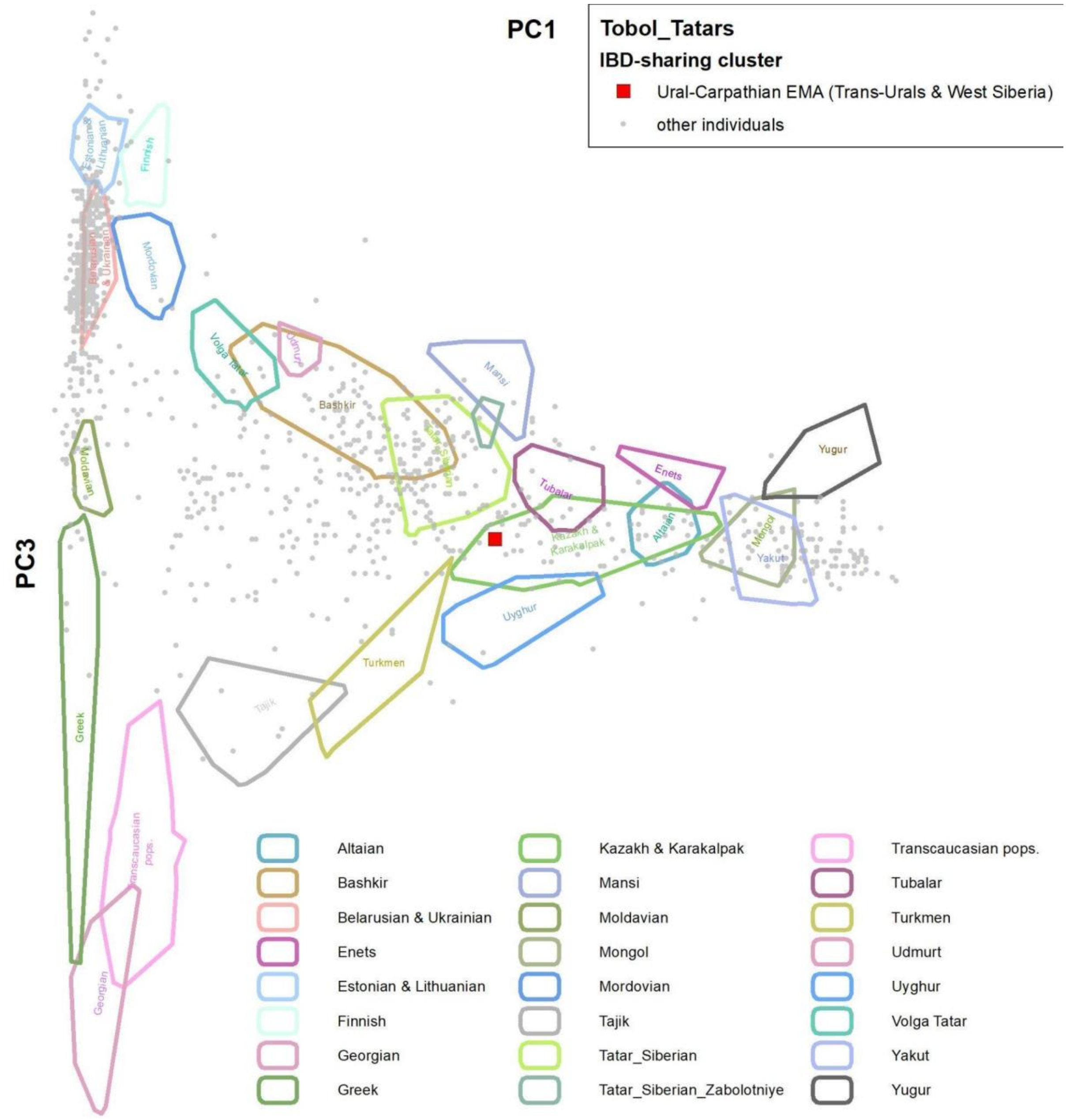


***Figure SIV.17.*** *Eurasian-scale principal component analysis (PCA), with a projection of the newly sequenced individuals onto modern genetic variation following Jeong et al. 2019. The PC1 and PC3 dimensions are shown, including the newly presented genomes. Polygons represent example populations from modern Eurasian individuals onto which the ancient samples were projected, while grey dots indicate ancient reference samples from our IBD-sharing network.*

## IV.G.1. Putilovo

### *unidentified burial (individual ID I10116, Male)*

The determined radiocarbon age of the burial is 1290-1397 cal CE (635±25 BP, PSUAMS-9121) (based on human bone).

# V. The Carpathian Basin

## V.A. The Conquest period sites (Early Medieval Magyars of the Carpathian Basin)

The Hungarian Conquest Period culture, first identified in 1834, has been extensively studied by scholars such as József Hampel, András Józsa, Nándor Fettich, Gyula László István Fodor, Csanád Bálint, László Kovács, and László Révész. The archaeological heritage of the Hungarian Conquest Period is primarily located in the Carpathian Basin, with significant presence along the Subbotsi type sites. With approximately 40,000 graves dating from the late 9th century to the early 12th century, the culture is characterized by inhumation in normal gravepits. Key artefacts such as archery equipment and horse harnesses serve as chronological indicators. The culture, primarily associated with pastoralism, exhibits no significant dietary indicators affecting radiocarbon dating or reflecting migrations. The conventional periodization places the culture in its early phase during the first half of the 10th century, with distinct variations observed in Transdanubia and the Great Hungarian Plain.

**Genetic results.** *In this group, we analyzed eleven individuals from the sites of Szeged-Öthalom (n=4), Harta-Freifelt (n=2), Balatonújlak (n=2), Makó-Igási járandó (n=2), and Kiszombor (n=1). All of these individuals had sufficient coverage for downstream population genetic modeling (>100,000 SNPs), and eight were suitable for IBD-sharing analysis (>600,000 SNPs). No close relatedness was detected among these samples, and the female-to-male ratio was 5:6. The samples show diverse genetic compositions, with some individuals displaying high Yakutia_LNBA ancestry, while Eastern Eurasian steppe-related Baikal_N component or Western Eurasian related Turkey_N ancestry were also detectable. Five samples clustered in the Urals-Carpathian EMA cluster. A generally diverse genetic composition was observed in both the previously published Early Medieval Magyars and the newly published samples. Their connection to the Urals-Carpathian EMA cluster indicates a relationship with other Early Medieval Magyars and the Karayakupovo horizon individuals, while one Szeged-Öthalom individual showed a strong connection to the Mid-Volga EVB group.*

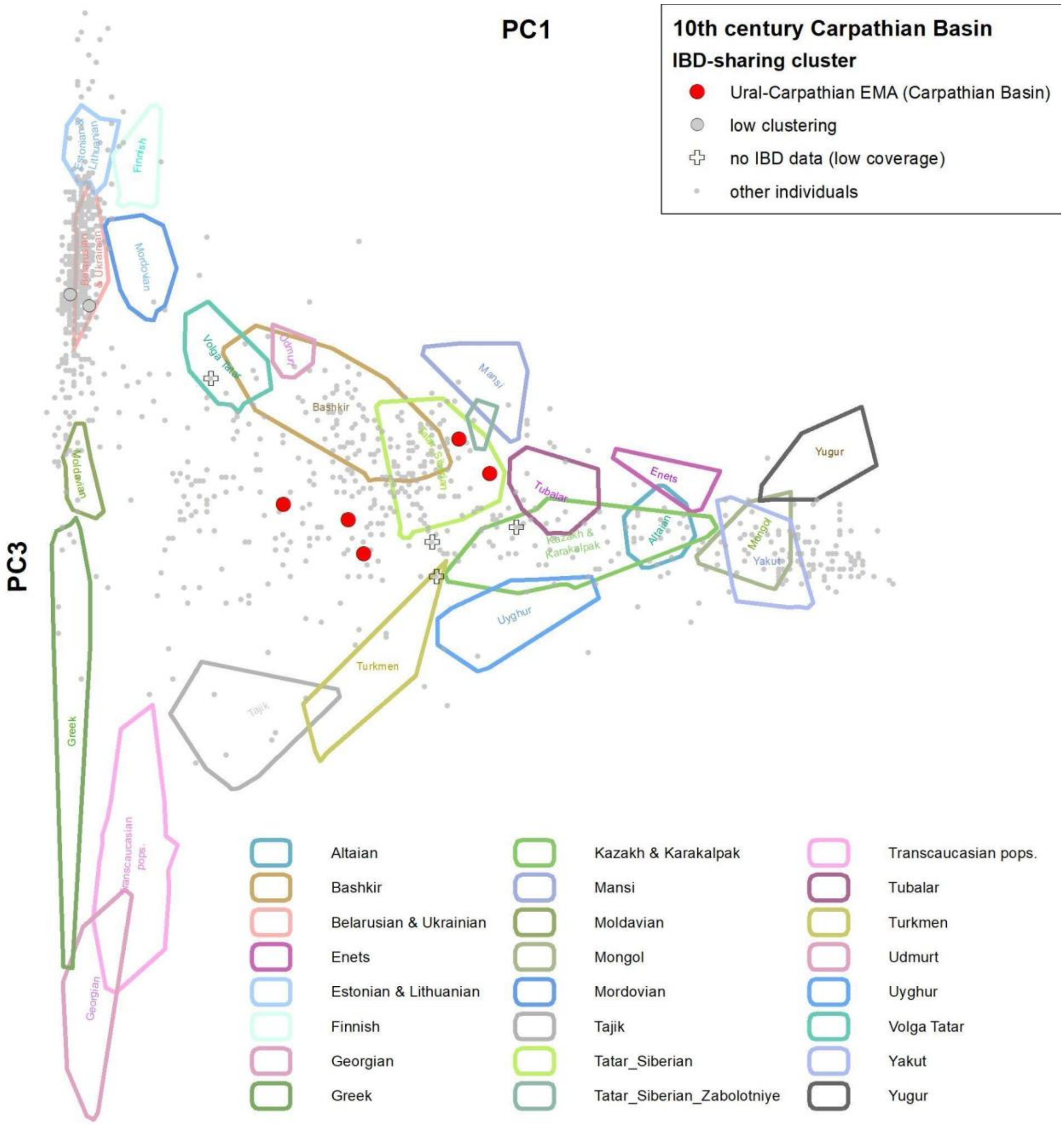


***Figure SIV.18.*** *Eurasian-scale principal component analysis (PCA), with a projection of the newly sequenced individuals onto modern genetic variation following Jeong et al. 2019. The PC1 and PC3 dimensions are shown, including the newly presented genomes. Polygons represent example populations from modern Eurasian individuals onto which the ancient samples were projected, while grey dots indicate ancient reference samples from our IBD-sharing network.*

## V.A.1. Balatonújlak

The site, discovered by Zsuzsanna Siklósi in 2003, is located on the north-northeastern edge of the Marcali hill, facing Lake Balaton. This site includes 26 graves arranged in a W-E orientation. No radiocarbon data is available, but chronological indicators such as saddles, harnesses, lozenge-shaped mounts, and caftan mounts date the grave to the mid-10th century. The cemetery represents a fully excavated site with inhumation burials, characteristic horse harnesses, and other specific artifacts. There is no evidence of cultural admixture, and

the main subsistence activity inferred is pastoralism. The cemeteries of the 10th century Carpathian Basin that have been fully excavated so far show that this is the site where most of the graves (including many children's and women's graves) contained weapon finds. In this sense, the site is associated with a community for which military life may have been important and defining.The cemetery is attributed to the early Hungarian culture based on archaeological evidence (Langó, SIklósi 2013).

### *grave 10 (individual ID I19075, Female)*

Grave 10, described by Zsuzsanna Siklósi and Péter Langó, is part of an Early Hungarian cemetery from the 10th century in the Carpathian Basin. Burial 10 is oriented west-east and belongs to a burial row. The grave is a rectangular pit without any constructions such as stone slabs or timber coverings. It contained a stretched skeleton laid on its back with no traces of a coffin. Artifacts found in the grave include characteristic horse harnesses, pear-shaped stirrups, lozenge-shaped mounts, and caftan mounts. No food or dye was discovered. The grave contained the skeleton of an adult woman and an infant, but their exact ages are unknown. The mounts date the burial to the mid-10th century, though no $^{14}C$ dates are provided. Cultural attribution details are not specified, but Western European (Italian) coins found in the cemetery suggest significant cultural interactions.

## V.A.2. Harta-Freifelt

The Early Hungarian cemetery Harta from the 10th century Carpathian Basin, first described by Rozália Kustár and Péter Langó, is situated in the Danube-Tisza interfluve region of Bács-Kiskun County. This fully excavated site, discovered by Rozália Kustár and Péter Langó in 2002, comprises 21 graves arranged in three rows, located on a hillside. The cemetery, which features inhumations with characteristic caftan mounts dating to the mid-10th century, includes typical pastoralist artifacts. The assemblage, particularly the richer female graves compared to poorer male graves, aligns with the Early Hungarian cultural practices in the Danube-Tisza interfluve region of the Hungarian Great Plain (Langó et al. 2016).

### *burial 9 (individual ID I19077, Female)*

Burial 9 is oriented W-E and lacks any visible grave marker or construction. The grave is a simple, rectangular pit with a depth of 7.5 cm and a skeleton length of 161 cm. The skeleton, a female estimated to have been 30-40 years old at death, was found lying on its back in a stretched position with the skull turned to the right and arms outstretched beside the body. No tree-trunks or coffins were present. The grave contained caftan mounts and textile remains, but no evidence of food offering. The burial, dated to the mid-10th century based on the artefactual evidence, specifically the caftan mounts, aligns with the Early Hungarian cultural practices. Despite being disturbed by animals, which displaced stones and finger bones, the remains are well-preserved. There is no cranial or odontological data, nor dietary details available. Paleopathological analysis indicated no battle injuries. The presence of small beads in the cemetery suggests the use of Byzantine luxury textiles, reinforcing the cultural attribution to the Early Hungarians.

***burial 19 (individual ID I19070, Female)***

Burial 19 with a grave depth of 32 cm, length of 250 cm, and width of 87.5 cm. The skeleton, a female estimated to have been 61-67 years old at death, measures 153 cm in length and is oriented W-E (280-100). The skeleton was found lying on its back in a stretched position with the skull turned to the left. The arms were placed next to the body, broken at the elbows, and bent over the left pelvic floor, with some phalanges displaced by animals. There were no traces of a coffin, although the position of the deceased does not exclude the possibility of one. The grave contained a silver ring but no evidence of food. Paleopathological analysis indicated no battle injuries, and there is no available cranial or odontological data, nor dietary details. Based on artefactual evidence, specifically the silver ring, the burial is dated to the mid-10th century (in this regard, it was considered one of the poorly furnished female graves in the cemetery). The cemetery is attributed to Early Hungarian culture, with the presence of small beads suggesting the use of Byzantine luxury textiles, reflecting possible cultural interactions.

## V.A.3. Szeged-Öthalom, V. homokbánya

The site, discovered during a rescue excavation by the Móra Ferenc Múzeum in 2009, is situated atop sand dunes, characteristic of the period. Referred to as a flat cemetery, it features inhumations without distinct rows, with graves positioned close to the sand dune tops. The graves typically contain single individuals in simple chamber gravepits. Radiocarbon dating from all graves suggests a chronological placement in the first third of the 10th century or the end of the 9th century, with some graves indicating a later usage around the middle of the 10th century. Artefactual evidence, including grave goods with parallels from Eastern Europe, indicates cultural connections, while the presence of west European coins from military campaigns to North Italy in the early 10th century suggests broader trade networks. The site's overall phase spans the first half of the 10th century, with evidence pointing to possible changes in community usage over time (Türk et al., 2015).

***grave 36 (individual ID I19071, Male)***

Grave 36 belongs to the flat cemetery and lacks any burial constructions. The grave is rectangular with rounded corners, oriented NNE-SE (335-155º), measuring 240 cm in length, 70 cm in width at the shoulders and feet, and 110 cm in depth. The buried individual, a male aged 40-45, was found in a supine, stretched position with the skull slightly tilted to the left side and the shoulders contracted. The arms were tightly against the ribs, while the legs were extended and brought together at the knees. Artefacts recovered from the grave include elements of horse harnesses, archery equipment, a silver hair ring, a finger ring, and some silver fragments. The presence of bones positioned higher up from the bottom of the pit suggests the use of a rounded tree coffin. A radiocarbon date (Poz-42783) obtained from the burial falls within the range of 780-874 cal CE (68.2%) and 766-895 cal CE (87.7%) (Türk et al., 2015).

***grave 124 (individual ID I19072, Male)***

The burial, identified as Szeged-Öthalom, V. homokbánya, Grave 124 is rectangular with rounded corners, oriented NE-SE (318-138º), measuring 235 cm in length and 90 cm in width

at the shoulders, with a depth ranging from 7 to 34 cm. The grave contained the skeletal remains of a mature male aged 50-59 lying on his back in a stretched position, with mechanical damage to the trephined skull and disturbance on the right side of the skeleton from an animal passage. Artefacts recovered from the grave include elements of horse harnesses, archery equipment, flintstone, kresalo, two silver hair rings, sabretashes with bronze mounts, and an iron knife. Additionally, the upper leg of a sheep was found, possibly indicative of funeral food. The presence of bones positioned higher up from the bottom of the pit suggests the use of a rounded tree coffin. A radiocarbon date (Poz-42782) obtained from the burial falls within the range of 780-877 cal CE (68.2%) and 768-897 cal CE (88.8%). The burial is associated with the first phase of the Hungarians settling in the Carpathian Basin (Türk et al., 2015).

### *grave 132 (individual ID I19073, Male)*

The burial, identified as Szeged-Öthalom, V. homokbánya, Grave 132 is rectangular with rounded corners, oriented NNW-SSE (304-124º), measuring 210 cm in length and 70 cm in width at the shoulders, with a depth of 20 cm. The grave contained the skeletal remains of a mature male aged 50-59 lying on his back in a normal position. Artefacts recovered from the grave include elements of horse harnesses, archery equipment, flintstone, kresalo, two silver fragments, sabretashes with bronze mounts, an iron knife, and a bronze tool for determining the size of belts. Additionally, the right part of a sheep shoulder was found, possibly indicative of funeral food. The presence of bones positioned higher up from the bottom of the pit suggests the use of a rounded tree coffin. A radiocarbon date (Poz-42778) obtained from the burial falls within the range of 782-891 cal CE (68.2%) and 774-900 cal CE (83.1%). The burial is culturally attributed to the classic Hungarian Conquest Period stage, with no apparent import influences in burial rites or artefacts. A separate radiocarbon date (Poz-42793) obtained from a horse found in the burial suggests a date range of 872-967 cal CE (68.2%) and 805-973 cal CE (91.1%) (Türk et al., 2015).

### *grave 237 (individual ID I19074, Male)*

Orientation: west–east (268–88°). The grave pit measured 226–230 cm in length, 80–86 cm in width, and 13–16 cm in depth. After removing the 30–35 cm thick humus layer, the grave stain became clearly visible on the subsoil. The rectangular grave pit with rounded corners had steeply sloped side walls and a flat base. Within the grave lay the relatively well-preserved skeletal remains of a mature (45–55-year-old) woman. Her shoulders were slightly raised, the arm bones were mildly bent at the elbows but extended, and were found resting on a 2–4 cm thick layer of fill.

Radiocarbon analysis of the anthropological material, conducted in Poznań (Poz-42777), dated the likely period of burial to 781 (68.2%)–888 cal CE or 771 (86.1%)–900 cal CE. At the eastern end of the grave, in front of the feet and at the grave floor level, partial remains of a 2½–3-year-old stallion were found. Two lower leg bones lay side by side, beneath the horse skull, placed crosswise with the hooves pointing southeast and northwest respectively. The fragmentarily preserved horse skull—most of which, along with two lower leg bones, was destroyed by later (modern) intrusion—lay on its jaw, oriented westward. Among the finds

were a fragmentary pair of iron stirrups, and based on the position of these stirrups and the horse bones, the saddle was likely placed crosswise at the eastern end of the grave. A small perforated silver plate fragment, part of the horse harness, was also recovered. A plain bone needle case made from the ulna of a waterfowl, containing remains of an iron needle, was found as well. A straight, single-edged iron knife with a corroded surface lay on the left side of the chest. On the central tang and one side of the blade, traces of wooden remains conserved by iron oxide were visible. Numerous glass beads were recovered from the grave, primarily from the left side of the chest and beneath the ribs (Türk et al. 2015).

## V.A.4. Makó-Igási járandó

As part of the construction of the M43 motorway, a large, multi-period archaeological site was excavated in 2008 in the Igási Járandó area, located to the north-northeast of Makó, along the route of the bypass road circumventing the town from this direction. During the excavation, 16,500 m² were investigated, revealing 467 archaeological features. These included Bronze Age (Makó and Tumulus culture), Scythian, Celtic, and Árpádian-period settlement features. At the southeastern edge of the excavation area, a Sarmatian cemetery and a 10-11th-century burial ground were also uncovered. At the southern edge of the site an Árpádian-period burial ground, consisting of 10 graves on the southeastern edge of the loess plateau, was fully excavated (Balogh, 2016).

### *grave 5 (individual ID I19099, Female)*

Grave 5 (Object 260) was oriented west to east, at an angle of 270–90°. The grave measured 197 cm in length, 55 cm in width, and 14 cm in depth. The grave was discovered after a skullcap was found during the removal of earth; no visible grave pit spot was detected. The grave pit itself was narrow and irregular in shape. Inside lay the skeleton in an extended supine position, anthropologically described as a 25–30-year-old female cranial traits attributed to the Europo-Mongoloid skull type according to classical physical anthropological definitions. Her skull was tilted to the left, and the upper body leaned slightly in the same direction. The right shoulder was markedly raised, while the left arm was bent and rested over the pelvis. The right arm was extended and lay tightly along the edge of the grave pit. The leg bones were parallel. The upper vertebrae had been disturbed by an animal burrow, which displaced them next to the right upper arm.

To the right of the sternum, one half of a hollow pendant was found in situ, with its front side facing downward, toward the base of the grave. The other half was recovered from the grave fill, in a secondary position. The hollow pendant, reassembled from the two halves, was made of a copper-based alloy. Its round loops had a triangular cross-section, and beneath them, on the neck of the button, a raised rib ran transversely. The elongated drop-shaped, hollow body was decorated with a leafy palmette motif. On the back side of the half buttons, slanted file marks were visible along the 0.1 mm wide edge. The pendant measured 2.7 cm in height and 1.5 cm at its widest point. After the removal of the skeletal remains, during the trenching of the grave, another half of a hollow pendant was found in an animal burrow at a depth of 50 cm from the bottom of the grave, around the middle of the chest area. This cast bronze piece bore traces of tinning on its convex outer surface. The loop was oval-shaped with a semicircular cross-section. The robust, hollow, drop-shaped button body was concave on the bottom, and

its surface featured two nearly parallel curved grooves running horizontally, between which elongated, curved segments were visible. The thin edge on the back had a flat surface (Balogh, 2016).

### *grave 8 (individual ID I19100, Male)*

Grave 8 (Object 512) was oriented northwest by north to southeast by south, at an angle of 300–60°. The grave measured 192 cm in length, 69 cm in width, and 14 cm in depth. The oval-shaped grave stain appeared with a greyish-brown mixed fill. In the grave pit, which had vertical sides and a flat base, lay the well-preserved skeleton of a 25–30-year-old Europid male displaying some Mongoloid features. The skeleton was in a supine, extended position, located along the eastern side of the pit. The skull was tilted backward, and the mandible had dropped. Both shoulders were strongly raised. The left arm lay extended, while the right arm was bent over the pelvis. The legs were positioned parallel to each other (Balogh, 2016).

## V.A.5. Kiszombor C

The Kiszombor C cemetery was excavated by Ferenc Móra between 1928 and 1930, in the Nagyhalom area, located 1200 m southwest of the settlement of Kiszombor. The site is situated on a natural elevation and on the side of a prehistoric kurgan built on top of it. A total of 33 graves were uncovered here. In 2003, a verification excavation was conducted (Langó, Türk 2003). Based on the artefacts excavated by Ferenc Móra, the use of the Conquest-period cemetery began in the 10th century and continued into the 11th century.

### *grave 37 (individual ID I19101, Male)*

The grave of an adult male was oriented west–east (330–130°). The grave pit was rectangular with slightly rounded corners, measuring 253 cm in length and 80–85 cm in width. The skeleton itself measured 184 cm. From the point of its appearance, the depth of the grave was 20 cm, while the total depth from the surface to the grave floor ranged between 132 and 140 cm. The western end and corner of the grave had been disturbed by another feature. On the northern side, next to the left upper arm, the grave had a ledge-like formation, although this did not continue along the entire length of the pit. One stirrup laid on this ledge, and to its east, a large-ring snaffle bit and a girth buckle were found, although these were not on the ledge but in the fill of the grave, at a height of 18–22 cm. Toward the center, the grave sloped basin-like, so the skull and legs lay 4–7 cm higher than the pelvis. The deceased's arms were bent toward the pelvis, and the head had been supported by an object made of organic material, which, after decaying, caused the skull to tilt backward and to the right. However, the cervical vertebrae remained upright in a vertical column. Next to the left upper arm, a sabre was unearthed, its pommel decorated with a pear-shaped, palmette-ornamented finial with a pronounced grip extension made of gilt silver and bronze. The cross-guard was boat-shaped, and the sabre measured 65 cm in length. Its tip was tightly positioned along the grave wall, outside the left knee. On the outer side of the sabre's hilt, at the bottom of the grave wall on the northern side, a battle axe was found embedded in the floor. The wood fibers were clearly visible on the haft. On the opposite side of the skull, near a green spot, a fragment of an open-ended hair ring was recovered, with black leather remains beneath it. The other half of the hair

ring was found on the left side of the chest, outside one of the ribs, where it left a green patina stain, suggesting that the item had already been damaged when placed in the grave. Beneath the left ilium, a strike-a-light, flint, and iron knife were discovered. On the outer side of the upper end of the left femur, a pear-shaped iron stirrup lay on its side at a slight angle, 3–6 cm higher than the grave floor, with its loop facing north. Fragments of quiver fittings were recovered from atop the right ilium and between the feet. Iron rods used to stiffen the quiver were also found beneath the sabre. On the sabre's cross-guard, one armor-piercing and two rhombus-shaped arrowheads were found, showing clear binding marks on their tangs from where they had been tied with sinew. A ¼ coin was placed in the deceased's mouth as an obolus. A sheep bone, specifically a scapula, was placed on the chest. After the removal of the skull, another open-ended hair ring was recovered at the ear location, surrounded by leather remnants. Beneath the wrist, flint and strike-a-light were found, alongside a fragment of a whetstone. On the inner side of the whetstone, a small, single-edged iron knife with wood impression was found standing on its edge. Beneath the right ilium, among the hand bones, a perforated coin fragment was discovered. Among the right-side ribs, yellowish discoloration was observed, likely indicating a textile imprint (Langó, Türk, 2003)

## References


1. **Alabin, 1860:** Алабин П.В. 1860. Ананьинский могильник (The Ananyino Burial Ground). *Вестник Императорского Русского географического общества*, Ч. XXIX, № 6, с. 87–120.

2. **Balogh, 2016:** Balogh Cs. 2016. Kora Árpád-kori szállási temető Makó-Igási járandóban = Campsite Burial Ground from the Early Árpádian Age at Makó-Igási járandó. In: Népek és kultúrák a Kárpát-medencében. Magyar Nemzeti Múzeum (MNM), Budapest, pp. 391-421. ISBN 978-615-5209-56-7
3. **Belavin, Krylasova, 2016:** Белавин, А.М., Крыласова, Н.Б., 2016. Проблема периодизации средневековых археологических культур Пермского Предуралья (The Problem of Periodization of Medieval Archaeological Cultures of the Perm Pre-Ural Region). *Вестник Пермского университета. Серия: История*, № 1 (32), С. 28–41.
4. **Belyavskaya, Protsenko, 2018:** Белявская, О.С., Проценко, А.С., 2018. Керамический комплекс городища Уфа-II как отражение этнокультурных процессов в эпоху средневековья (по материалам раскопок 2017 года) (The Ceramic Complex of Ufa-II Settlement as a Reflection of Ethnocultural Processes in the Middle Ages Based on 2017 Excavations). В: *Древние и средневековые общества Евразии: перекресток культур. Международный научный симпозиум, посвященный памяти профессора Н.А. Мажитова*. Уфа: Мир печати, С. 204–224.
5. **Borzunov, Chemiakin, 2014:** Борзунов, В.А., Чемякин, Ю.П., 2014. Карымские могильники таежного Приобья (Karym Burial Sites of the Taiga Ob Region). *Вестник археологии, антропологии и этнографии*, № 2 (25), С. 64–70.
6. **Botalov 2016**: Боталов, С. Г. Историко-культурные горизонты в эпоху раннего железного века и средневековья лесостепного Зауралья (Historical and Cultural Horizons in the Early Iron Age and the Middle Ages of the Trans-Ural Forest-Steppe). В: *Южный Урал в раннем железном веке и средневековье* / Под ред. С. Г. Боталова, А. В. Епимахова, А. И. Коровлёва. С. 468–541. Челябинск: Магеллан, 2016.
7. **Botalov 2017**: Боталов, С. Г. Погребальный комплекс Уелги и некоторые наблюдения на предмет угорского и мадьярского культурогенеза (The Uelgi Burial Complex and Some Observations on Ugric and Magyar Ethnogenesis). В: Hadak útján XXIV. A népvándorláskor fiatal kutatóinak XXIV. konferenciáján elhangzott előadások. Budapest–Esztergom, 2017. С. 267–333. DOI: 10.55722/Arpad.Kiad.2017.3.2_13
8. **Botalov 2019:** Боталов С.Г. Зауральская ойкумена эпохи Средневековья (The Trans-Ural Oecumene in the Middle Ages). *Археология евразийских степей*. 2019. № 6. С. 138–159.
9. **Brather, 2004:** Brather, S. 2004. *Ethnische Interpretationen in der frühgeschichtlichen Archäologie. Geschichte, Grundlagen und Alternativen* (Ethnic Interpretations in Prehistoric Archaeology: History, Basics, and Alternatives). Berlin; New York: Walter de Gruyter Verlag.
10. **Bugrov et al. 2010: Бугров, Д.Г., Капленко, Н.М., Ситдиков, А.Г., 2010.** К вопросу о начале мусульманизации населения восточной периферии Волжской Булгарии (On the Beginning of Islamization of the Population of the Eastern

Periphery of Volga Bulgaria). *Научный Татарстан*, № 4, С. 19–25.
11. **Bugrov, Asylgaraeva, 2020:** Бугров, Д.Г., Асылгараева, Г.Ш., 2020. Животные в погребальном обряде населения Нижнего Прикамья первой половины I тыс. н.э. (по материалам Гулюковского могильника) (Animals in the Burial Rituals of the Population of the Lower Kama Region in the First Half of the 1st Millennium AD Based on Materials of the Gulyukovo Burial Site). *Поволжская археология*, № 1 (31), С. 146–166.
12. **Bulychov, 1902:** Булычов, Н.И., 1902. *Древности из Восточной России. Вып. 1* (Antiquities from Eastern Russia. Issue 1). Москва: Товарищество А.И. Мамонтова.
13. **Chemyakin, Karacharov, 2002:** Чемякин Ю.П., Карачаров К.Г. 2002. Древняя история Сургутского Приобья (The Ancient History of the Surgut Priobie Region). *Очерки традиционного землепользования хантов* (Essays on the Traditional Land Use of the Khanty). 2-е издание, исправленное и дополненное. Екатеринбург: Тезис. С. 5-74.
**Chemyakin, Zykov, 2004:** Чемякин Ю.П., Зыков А.П. 2004. Барсова Гора: археологическая карта (Barsova Gora: Archaeological Map). Сургут — Омск: Омский дом печати.
14. **Chernykh, Chizhevsky, 2021:** Черных Е.М., Чижевский А.А. 2021. Ананьинская культурно-историческая область. Вятско-ветлужская культура (The Ananyino Cultural-Historical Region. Vyatka-Vetluga Culture). В: *Ранний железный век*, *Археология Волго-Уралья*, Т. III, отв. ред. А.А. Чижевский. Казань: ИА АН РТ, с. 217–241.
15. **Chikunova, 2017:** Чикунова И.Ю. 2017. Ипкульский могильник (The Ipkul Burial Ground). *AB ORIGINE: археолого-этнографический сборник Тюменского государственного университета* (Archaeological and Ethnographic Collection of Tyumen State University). Выпуск 9. Тюмень: Издательство Тюменского государственного университета. С. 79-109.
16. **Chizhevsky and Volkova, 2021:** Чижевский А.А., Волкова Е.В. 2021. Ананьинская культурно-историческая область. Постмаклашеевская культура. Памятники белогорского типа (The Ananyino Cultural-Historical Region: Post-Maklasheevo Culture, Belogorye-Type Monuments). В: Ранний железный век (The Early Iron Age). Археология Волго-Уралья (Archaeology of the Volga-Ural Region), том III, ответственный редактор А.А. Чижевский. Казань: Институт археологии Академии наук Республики Татарстан. С. 162–185.
17. **Chizhevsky et al., 2004:** Чижевский А.А., Шипилов А.В., Писарев Т.Н. 2004. Исследования Рысовского археологического комплекса в 2002 году (Studies of the Rysovsky Archaeological Complex in 2002). *Археологические открытия в Татарстане 2002 год* (Archaeological Discoveries in Tatarstan, 2002). Казань. С. 105-144.
18. **Chizhevsky, 2017:** Чижевский А.А. 2017. Памятники позднего периода ананьинской культурно-исторической области (Late Period Sites of the Ananyino Cultural-Historical Region). *Археология Евразийских степей*, № 4, с. 196–256.
19. **Chizhevsky, 2020:** Чижевский А.А. 2020. Ананьинская культурно-историческая область (The Ananyino Cultural-Historical Region). *Труды VI (XXII) Всероссийского археологического съезда в Самаре: В 3-х томах, Самара, 01–02 октября 2020 года. Том II* (Proceedings of the VI (XXII) All-Russian Archaeological Congress in

Samara, 1–2 October 2020. Volume II). Самара: Самарский государственный социально-педагогический университет. С. 132-133.

20. **Chizhevsky, Chernykh, and Korenyuk, 2021:** Чижевский А.А., Черных Е.М., Коренюк С.Н. 2021. Ананьинская культурно-историческая область. Ананьинская классическая культура (шнуровой керамики) (The Ananyino Cultural-Historical Region: Classic Ananyino Culture (Corded Ceramics)). В: Ранний железный век (The Early Iron Age). Археология Волго-Уралья (Archaeology of the Volga-Ural Region), том III, ответственный редактор А.А. Чижевский. Казань: Институт археологии Академии наук Республики Татарстан. С. 186–216.
21. **Chizhevsky, et al, 2004:** Чижевский А.А., Шипилов А.В., Писарев Т.Н. 2004. Исследования Рысовского археологического комплекса в 2002 году (Studies of the Rysovsky Archaeological Complex in 2002). В: Археологические открытия в Татарстане 2002 год (Archaeological Discoveries in Tatarstan, 2002), ответственный редактор Ф.Ш. Хузин. Казань. С. 105–144.
22. **Chizhevsky, et al, 2014:** Чижевский А.А., Лыганов А.В., Шипилов А.В. 2014. Рысовский археологический комплекс (The Rysovsky Archaeological Complex). В: Актуальные вопросы российской археологии (Current Issues in Russian Archaeology), выпуск 1, научный редактор В.А. Шаталов. Казань: Центр изучения археологических исследований (ЦИАИ). С. 23–53.
23. **Chizhevsky, et al, 2019:** Чижевский А.А., Лыганов А.В., Кузьминых С.В. 2019. Ранний (атабаевский) этап маклашеевской культуры (The Early (Atabaev) Stage of the Maklasheevo Culture). Археология Евразийских степей (Archaeology of the Eurasian Steppes), №2. С. 99–123.
24. **Chizhevsky, Kuzminykh, and Patrushev, 2021:** Чижевский А.А., Кузьминых С.В., Патрушев В.С. 2021. Ананьинская культурно-историческая область. Культура текстильной керамики (акозинско-ахмыловская) (The Ananyino Cultural-Historical Region: Textile Ceramics Culture (Akozino-Akhmylovskaya)). В: Ранний железный век (The Early Iron Age). Археология Волго-Уралья (Archaeology of the Volga-Ural Region), том III, ответственный редактор А.А. Чижевский. Казань: Институт археологии Академии наук Республики Татарстан. С. 141–161.
25. **Chizhevsky, Liganov, and Kuzminykh, 2021:** Чижевский А.А., Лыганов А.В., Кузьминых С.В. 2021. Маклашеевская культура (The Maklasheevo Culture). В: Энеолит и бронзовый век (The Eneolithic and Bronze Age). Археология Волго-Уралья (Archaeology of the Volga-Ural Region), том II, ответственный редактор А.А. Чижевский. Казань: Институт археологии Академии наук Республики Татарстан. С. 601–637.
26. **Faifert, 2022:** Файферт А.В. 2022. *Археологическая культура и структура археологической науки (Archaeological Culture and the Structure of Archaeological Science).* Теория и практика археологических исследований (Theory and Practice of Archaeological Research), Т. 34, №1, С. 9–29. DOI: 10.14258/tpai(2022)34(1).-01.
27. **Garustovich, et al., 2008:** Гарустович, Г.Н., Иванов, В.А., Шилов, С.А., 2008. Средневековые могильники лесостепной зоны Западной Сибири (Medieval Burial Sites of the Forest-Steppe Zone of Western Siberia). *Finno-Ugrica* (Kazan), № 11, С. 54–68.
28. **Garustovich, 2015:** Гарустович, Г.Н., 2015. Чияликская археологическая культура эпохи средневековья на Южном Урале (The Chiyalik Archaeological Culture of the

Medieval Period in the South Urals). *УАВ* (Удмуртская археологическая вертикаль), Вып. 15. Уфа, С. 181–198.

29. **Gavritukhin, Ivanov, 1999:** Гавритухин, И.О., Иванов, А.Г.**,** 1999. Погребение 552 Варнинского могильника и некоторые вопросы изучения раннесредневековых культур Поволжья (Grave 552 of the Varninsky Burial Site and Some Issues of Studying Early Medieval Cultures of the Volga Region). В: *Пермский мир в раннем средневековье / Archaeologia Permica*. Вып. 1. Ижевск, С. 99–159.
30. **Gening, 1959:** Генинг, В.Ф., 1959. Очерк этнических культур Прикамья в эпоху железа (Essay on the Ethnic Cultures of the Kama Region in the Iron Age). *Тр. Казанского филиала АН СССР, Серия гуманитарных наук*, Вып. 2. Казань, С. 157–219.
31. **Gening, 1967:** Генинг, В.Ф., 1967. Мазунинская культура в Среднем Прикамье (The Mazunino Culture in the Middle Kama Region). *ВАУ* (Вопросы археологии Урала), Вып. 7: Памятники мазунинской культуры. С. 7–84.
32. **Gening, 1977:** Генинг, В.Ф., 1977. Памятники у с. Кушнаренково на р. Белой (Sites Near the Village of Kushnarenkovo on the River Belaya). В: *Исследования по археологии Южного Урала* (Studies on the Archaeology of the Southern Urals). Уфа.
33. **Goldina et al., 2018:** Голдина Р.Д., Перевозчикова С.А., Голдина Е.В. 2018. Могильник VI–IX вв. у д. Верх-Сая в Кунгурской лесостепи (Cemetery of the 6th–9th Centuries near the Village of Verkh-Saya in the Kungur Forest-Steppe). *Материалы и исследования по культуре Восточной Европы* (MI KVAÉ), Том 19. Ижевск.
34. **Goldina, 1970:** Голдина Р.Д. 1970. Могильники VII–IX вв. в Верхнем Прикамье (Cemeteries of the 7th–9th Centuries in the Upper Kama Region). *Вопросы археологии Урала* (VAU), Выпуск 9. Свердловск. С. 57–113.
35. **Goldina, 1971:** Голдина, Р.Д., 1971. Ломоватовская культура в Верхнем Прикамье (Lomovatovo Culture in the Upper Kama Region). Автореферат диссертации на соискание ученой степени кандидата исторических наук (Abstract of the Dissertation for the Degree of Candidate of Historical Sciences). Ленинград, 21 с.
36. **Goldina, 1980:** Голдина, Р.Д., 1980. Агафоновский I могильник — памятник ломоватовской культуры на севере Пермской области (Agafonovsky I Cemetery: A Site of the Lomovatov Culture in the North of Perm Region). В: *Памятники эпохи средневековья в Верхнем Прикамье* (Sites of the Medieval Period in the Upper Kama Region). Ижевск, С. 3–66.
37. **Goldina, 1985:** Голдина, Р.Д., 1985. Ломоватовская культура в Верхнем Прикамье (Lomovatovo Culture in the Upper Kama Region). Иркутск: Издательство Иркутского университета.
38. **Goldina, 2012:** Голдина Р.Д. 2012. О датировке и хронологии неволинской культуры (конец IV – начало IX в. н.э.) (On the Dating and Chronology of the Nevolino Culture (Late 4th Century – Early 9th Century AD)). В: *Древности Прикамья эпохи железа (VI в. до н.э. – XV в. н.э.): хронологическая атрибуция* (Antiquities of the Kama Region in the Iron Age (6th Century BC – 15th Century AD): Chronological Attribution). *Материалы и исследования по культуре Восточной Европы* (MI KVAÉ), Том 25. Ижевск: Удмуртский университет. С. 203–285.
39. **Goldina, et al., 2011:** Голдина, Р.Д., Пастушенко, И.Ю., Черных, Е.М., 2011. Бартымский комплекс памятников эпох средневековья в Сылвенском поречье (The Bartym Complex of Medieval Sites in the Sylva River Basin). *МИ КВАЭ* (MI

KVAÉ), Т. 13. Ижевск; Пермь: Удмуртский гос. университет, 340 с.
40. **Goldina, Kananin, 1989:** Голдина, Р.Д., Кананин, В.А., 1989. Средневековые памятники верховьев Камы (Medieval Sites of the Upper Kama River). Свердловск: Издательство Уральского университета.
41. **Goldina, Perevozchikova, 2012:** Голдина Р.Д., Перевозчикова С.А. 2012. О датировке и хронологии Верх-Саинского могильника неволинской культуры (VI — начало IX в. н.э.) (On the Dating and Chronology of the Verkh-Saya Cemetery of the Nevolino Culture (6th – Early 9th Century AD)). В: *Древности Прикамья эпохи железа (VI в. до н.э. – XV в. н.э.): хронологическая атрибуция* (Antiquities of the Kama Region in the Iron Age (6th Century BC – 15th Century AD): Chronological Attribution). *Материалы и исследования по культуре Восточной Европы* (MI KVAÉ), Том 25. Ижевск: Удмуртский университет. С. 321–375.
42. **Goldina, Vodolago, 1982:** Голдина, Р.Д., Водолаго, Н.В., 1982. Хронология погребальных комплексов неволинской культуры (Chronology of Burial Sites of the Nevolino Culture). В: *Проблемы этногенетических исследований Европейского Северо-Востока* (Problems of Ethnogenetic Studies of the European Northeast). Сыктывкар, С. 122–136.
43. **Goldina, Vodolago, 1990:** Голдина, Р.Д., Водолаго, Н.В., 1990. Могильники неволинской культуры в Приуралье (Burial Sites of the Nevolino Culture in the Pre-Ural Region). Иркутск: Издательство Иркутского университета.
44. **Grudochko and Botalov, 2013:** Грудочко, И.В., Боталов, С.Г. 2013. Этнокультурная ситуация в Южном Зауралье в VIII-XI веках (в свете новых данных исследований погребального комплекса Уелги) (Ethnocultural Situation in the Southern Trans-Urals in the 8th–11th Centuries (In Light of New Data from the Uelgi Burial Complex)). В: *II-й Международный Мадьярский симпозиум: сборник научных трудов.* Челябинск: Рифей, с. 110–138.
45. **Ilyushina, 2009:** Илюшина, В.В., 2009. Потчевашские погребения из грунтового могильника «Викуловское кладбище» (Potchevash Burials from the Earthen Cemetery "Vikulovskoye Burial Site"). *Вестник археологии, антропологии и этнографии* (*Bulletin of Archaeology, Anthropology, and Ethnography*), № 10, С. 74–83.
46. **Ivanov A., 1998:** Иванов, А.Г., 1998. Этнокультурные и экономические связи населения бассейна р. Чепцы в эпоху средневековья (конец V – 1-я половина XIII в.) (Ethnocultural and Economic Connections of the Population in the Cheptsa River Basin During the Middle Ages (Late 5th Century – First Half of the 13th Century)). Ижевск.
47. **Ivanov A., 1999:** Иванов, А.Г.**,** 1999. Новые материалы по ранней дате поломской культуры: курганная часть Варнинского могильника (New Materials on the Early Date of the Polom Culture: The Barrow Part of the Varninsky Cemetery). В: *Пермский мир в раннем средневековье* (*The Permian World in the Early Middle Ages*) / *Archaeologia Permica*. Вып. 1. Ижевск, С. 6–52.
48. **Ivanov A., 2008:** Иванов, А.Г., 2008. Группы кушнаренковско-караякуповского населения между Камой и Вяткой (Kushnarenkovsko-Karayakupovo Population Groups Between the Kama and Vyatka Rivers). В: *Проблемы бакальской культуры: мат. науч.-практ. семинара по проблемам бакальской культуры* (*Problems of the Bakal Culture: Proceedings of the Scientific-Practical Seminar on the Problems of Bakal Culture*). Челябинск-Шадринск, С. 147–161.

49. **Ivanov V., 1990:** Иванов В.А. 1990. Этнические процессы в степной и лесостепной полосе Южного Урала и Приуралья в VII–XIV вв. (Ethnic Processes in the Steppe and Forest-Steppe Zones of the Southern Urals and the Ural Region in the 7th–14th Centuries). Автореферат диссертации на соискание ученой степени доктора исторических наук: 07.00.06. Москва. 45 с.
50. **Ivanov V., 1999:** Иванов, В.А., 1999. Древние угры-мадьяры в Восточной Европе (Ancient Ugric-Magyars in Eastern Europe). Уфа.
51. **Ivanov V., 2009:** Иванов В.А. 2009. Ещё раз о «кушнаренковско-караякуповской проблеме» (Once Again on the "Kushnarenkovsko-Karayakupovo Issue"). В: *Среднее Поволжье и Южный Урал: человек и природа в древности* (The Middle Volga Region and Southern Urals: Man and Nature in Antiquity). Сборник научных статей, посвященный 75-летию доктора исторических наук Евгения Петровича Казакова (Collection of Scientific Articles Dedicated to the 75th Anniversary of Doctor of Historical Sciences Evgeny Petrovich Kazakov). Казань: Издательство «Фэн» Академии наук Республики Татарстан. С. 177–196.
52. **Ivanov V., 2022:** Иванов В.А. 2022. Динамика миграций в Урало-Поволжье в эпоху средневековья и их результаты (Migration Dynamics in the Ural-Volga Region During the Middle Ages and Their Results). Уфимский археологический вестник (Ufa Archaeological Bulletin), Т. 22, № 2. С. 299–309. DOI: 10.31833/uav/2022.22.2.010.
53. **Jeong et al., 2019:** Jeong, C., Balanovsky, O., Lukianova, E., Kahbatkyzy, N., Flegontov, P., Zaporozhchenko, V., Immel, A., Wang, C.-C., Ixan, O., Khussainova, E., et al. 2019. *The Genetic History of Admixture Across Inner Eurasia*. *Nature Ecology and Evolution*, 3, pp. 966–976. DOI: 10.1038/S41559-019-0878-2.
54. **Karacharov, 2004:** Карачаров К. Г. 2004. Раскопки могильника Барсов Городок (Барсовский I) в 1989 году: отчет о НИР (Excavations of the Barsov Gorodok Cemetery (Barsov I) in 1989: Report on Research Work). УрГУ; К. Г. Карачаров. Екатеринбург. // Архив АСА. Ф. 1. Д. 143.
55. **Karavashkina, 2017:** Каравашкина Е.А. 2017. Сравнительный анализ орнаментальных традиций населения неволинской культуры на памятниках Среднего Поволжья (на примере поселений Подгоры-I и Ош-Пандо-Нерь) (Comparative Analysis of Ornamental Traditions of the Population of the Nevolino Culture at Sites of the Middle Volga Region (On the Example of the Podgory-I and Osh-Pando-Ner' Settlements)). В: *Труды V (XXI) Всероссийского археологического съезда в Барнауле – Белокурихе* (Proceedings of the V (XXI) Russian Archaeological Congress in Barnaul - Belokurikha), Том II. Барнаул: Издательство Алтайского государственного университета. С. 174–178.
56. **Kazakov and Lyganov, 2014:** Казаков Е.П., Лыганов А.В. 2014. Итоги изучения Измерского XVII могильника (Results of the Study of the Izmery XVII Cemetery). *Поволжская археология*, № 4, с. 43–57.
57. **Kazakov, 1981:** Казаков Е.П. 1981. Кушнаренковские памятники Нижнего Прикамья (Kushnarenkovo Sites of the Lower Kama Region). В: *Об исторических памятниках по долинам Камы и Белой* (On Historical Sites of the Kama and Belaya River Valleys). Казань: Институт Языка, Литературы и Истории, Академия Наук СССР. С. 115–135.
58. **Kazakov, 1994:** Казаков Е.П. 1994. Измерский VII могильник (The Izmery VII Cemetery). В: *Памятники древней истории Волго-Камья* (Sites of Ancient History of the Volga-Kama Region). Ответственный редактор П.Н. Старостин. Казань:

Институт Языка, Литературы и Истории, Академия Наук СССР. С. 104–123.

59. **Kazakov, 2001:** Казаков Е.П. 2001. Кушнаренковская культура (The Kushnarenkovo Culture). В: *Очерки по археологии Татарстана* (Essays on the Archaeology of Tatarstan). Ответственный редактор П.Н. Старостин. Казань: Институт Языка, Литературы и Истории, Академия Наук Республики Татарстан. С. 119–122.
60. **Kazakov, 2002:** Казаков Е.П. 2002. О некоторых итогах работы Раннеболгарской экспедиции в 2001 году (On Some Results of the Early Bulgar Expedition in 2001). Археологические открытия Татарстане: 2001 год (Archaeological Discoveries in Tatarstan: 2001), ответственный редактор Ф.Ш. Хузин. Казань. С. 83–87.
61. **Kazakov, 2002:** Казаков Е.П. 2002. О некоторых итогах работы Раннеболгарской экспедиции в 2001 году (On Some Results of the Early Bulgar Expedition in 2001). В: *Археологические открытия Татарстане: 2001 год*, отв. ред. Ф.Ш. Хузин. Казань, с. 83–87.
62. **Kazakov, 2017:** Казаков Е.П. 2017. XVI Измерский могильник маклашеевской культуры (The XVI Izmery Cemetery of the Maklasheevo Culture). *Археология Евразийских степей* (Archaeology of the Eurasian Steppes), № 3. С. 80–88.
63. **Kazakov, Lyganov, 2014:** Казаков Е.П., Лыганов А.В. 2014. Итоги изучения Измерского XVII могильника (Results of the Study of the Izmery XVII Cemetery). *Поволжская археология* (Volga Archaeology), № 4. С. 43–55.
64. **Khalikov 2022:** Халиков, А.Х**.** 2022. Великая Венгрия между Волгой и Уралом (Great Hungary Between the Volga and the Ural). Институт археологии им. А.Х. Халикова АН РТ; отв. ред. А.Г. Ситдиков. *Серия «Археология евразийских степей»*. Выпуск 27. Воронеж: АЛЕКС ПРИНТ.
65. **Khalikov, 1977:** Халиков А.Х. 1977. Волго-Камье в начале эпохи раннего железа (VIII–VI вв. до н. э.) (The Volga-Kama Region at the Beginning of the Early Iron Age (8th–6th Centuries BC)). Москва: Наука. 262 с.
66. **Khalikova and Kazakov, 1977:** Khalikova E.A., Kazakov E.P. Le cimetiere de Tankeevka. Les anciens Hongrois etethnies voisines a l'Est. Budapest, 1977.
67. **Khalikova, 1976:** Халикова Е.А. 1976. Ранневенгерские памятники Нижнего Прикамья и Приуралья (Early Hungarian Sites in the Lower Kama and Ural Regions). *Советская археология* (Soviet Archaeology). № 3. С. 141-156.
68. **Khalikova, Khalikov 2018:** Халикова Е.А., Халиков А.Х. 2018. Ранние венгры на Каме и Урале (Больше-Тиганский могильник) (Early Hungarians on the Kama and Ural Rivers (Bolshoy Tigan Burial Ground)). *Археология евразийских степей. Приложение к журналу. Выпуск 25.* Казань: Фэн.
69. **Khisyametdinova, 2012:** Хисяметдинова А.А. 2012. Приложение 2. Геолого-геоморфологический анализ района раскопок Гулюковского могильника в 2012 г. (Appendix 2. Geological and Geomorphological Analysis of the Excavation Area of the Gulyukovsky Burial Ground in 2012). *Валиев Р.Р. Отчет об археологических охранно-спасательных исследованиях Гулюковского могильника в Мензелинском районе Республики Татарстан в 2012 году* (Report on the Archaeological Protective and Rescue Research of the Gulyukovsky Burial Ground in the Menzelinsky District of the Republic of Tatarstan in 2012). Казань: Научный фонд Министерства археологии Республики Татарстан. С. 66-71.
70. **Klein, 1979:** Клейн Л.С. 1979. *Понятие типа в современной археологии (The Concept of Type in Modern Archaeology).* В: *Типы в культуре (Types in Culture).*

Ленинград. С. 50–74.
71. **Klein, 2014:** Клейн Л.С. 2014. История российской археологии: учения, школы и личности. В 2-х тт. Т. 1. Общий обзор и дореволюционное время. Т. 2. Археология советской эпохи (History of Russian Archaeology: Teachings, Schools, and Personalities. In 2 Volumes. Vol. 1. General Overview and Pre-Revolutionary Time. Vol. 2. Archaeology of the Soviet Era). St. Petersburg: Eurasia.
72. **Klein, 2018:** Клейн Л.С. 2018. *Культура и эволюция. Теоретические исследования (Culture and Evolution. Theoretical Research).* Санкт-Петербург: Евразия.
73. **Kolonskikh et al., 2024:** Колонских А.Г., Саттаров Р.Р., Ситдиков А.Г., 2024. Бустанаевский курганный могильник – новый памятник Эпохи великого переселения на северо-западе Башкортостана (Bustanaevo Burial Mound – a New Great Migration Era Monument in the North-West of Bashkortostan). *Поволжская археология* (Volga Archaeology), № 4. С. 203–218. DOI: 10.24852/pa2024.1.47.203.218
74. **Konikov, 2017:** Коников Б.А. 2007. Омское Прииртышье в раннем и развитом средневековье (Omsk Trans-Urals in the Early and High Middle Ages). Омск: Наука.
75. **Konikov, 2019:** Коников Б.А. 2019. Иванов-Мыс-I курганный могильник. Тевризский район, Омская область (Ivanov-Mys I Burial Mound. Tevriz District, Omsk Region). Омск.
76. **Korol, Konkova, 2007:** Король Г.Г., Конькова Л.В. 2007. Южносибирские импорты в Восточной Европе и проблемы этнокультурного и регионального взаимодействия в X-XIII веках (Southern Siberian Imports in Eastern Europe and Problems of Ethnocultural and Regional Interaction in the 10th-13th Centuries). В: *Археологические вести* (Archaeological News), № 14. Москва, с. 145–156.
77. **Kuzminykh and Chizhevsky, 2009:** Кузьминых С.В., Чижевский А.А. 2009. Ананьинский мир: взгляд на современное состояние проблемы (The Ananyino World: A Look at the Current State of the Problem). В: *У истоков археологии Волго-Камья (к 150-летию открытия Ананьинского могильника)*, *Археология Евразийских степей*, вып. 8, отв. ред. С.В. Кузьминых, А.А. Чижевский. Елабуга: ИИ АНРТ, с. 29–55.
78. **Kuzminykh et al., 2024:** Kuzminykh, S.V., Chizhevsky, A.A., Vaskul, I.O., Krenke, N.A., Lopatina, O.A., Pereskokov, M.L., Stolyarov, E.V. 2024. *The Early Iron Age Western Forest Zone*. In: Rehren, T., Nikita, E. (Eds.), *Encyclopedia of Archaeology*, 2nd Edition, vol. 4. London: Academic Press. Pp. 683–711.
79. **Kuzminykh, 2000:** Кузьминых С.В. 2000. Археологическое изучение ананьинского мира в XX в.: основные достижения и проблемы (Archaeological Study of the Ananyino World in the 20th Century: Key Achievements and Problems). В: *Российская археология: достижения XX и перспективы XXI в.*, отв. ред. Р.Д. Голдина. Ижевск: УдГУ, с. 104–113.
80. **Kuzminykh, Chizhevsky, 2014:** Кузьминых С.В., Чижевский А.А. 2014. Хронология раннего периода ананьинской культурно-исторической области (Chronology of the Early Period of the Ananyino Cultural-Historical Region). *Поволжская археология*, № 3, с. 101–137.
81. **Langó, Türk 2003:** Langó P., Türk A. 2003. Móra nyomában — Előzetes beszámoló a Kiszombor határában 2003-ban végzett honfoglalás kori lelőhelyek hitelesítő feltárásairól. Múzeumi Kutatások Csongrád Megyében (2003) 203–214.

82. **Langó et al. 2016**: Langó P., Kustár R., Köhler K., Csősz A. 2016. A study of the tenth-century cemetery at Harta-Freifelt ANTAEUS 34. Communicationes ex Instituto Archaeologico Academiae Scientiarum Hungaricae 2016 389-417.
83. **Langó, Siklósi 2013:** Langó P., Siklósi Zs. 2013. 10. századi temető Balatonújlak–Erdő-dűlőn in: Révész, L. & Wolf, M. (eds.): A honfoglalás kor kutatásának legújabb eredményei. Tanulmányok Kovács László 70. születésnapjára. Monográfiák a Szegedi Tudományegyetem Régészeti Tanszékéről 3, (2013) 143-160.
84. **Markov, 1988:** Марков В.Н. 1988. Нижнее Прикамье в ананьинскую эпоху (об этнокультурных компонентах ананьинской общности) (The Lower Kama Region in the Ananyino Epoch: On the Ethnocultural Components of the Ananyino Community). Диссертация на соискание ученой степени кандидата исторических наук (PhD Dissertation). Ленинград.
85. **Markov, 2007:** Марков В.Н. 2007. Нижнее Прикамье в ананьинскую эпоху (об этнокультурных компонентах ананьинской общности) (The Lower Kama Region in the Ananyino Epoch: On the Ethnocultural Components of the Ananyino Community). *Археология евразийских степей* (Archaeology of the Eurasian Steppes), выпуск 4. Казань: Институт истории Академии наук Республики Татарстан.
86. **Matveeva G., 1975:** Матвеева Г.И**.** 1975. Памятники караякуповского типа в Приуралье (Karayakupovo Type Sites in the Pre-Urals). В: *Из истории Среднего Поволжья и Приуралья* (From the History of the Middle Volga Region and the Pre-Urals), Вып. 5. Куйбышев, с. 13–22.
87. **Matveeva G., 2007:** Матвеева Г.И. 2007. О культурном и хронологическом соотношении памятников кушнаренковского и кара-якуповского типов (On the Cultural and Chronological Correlation of Kusnarankov and Karayakupov Type Sites). В: *Средневековая археология евразийских степей* (Medieval Archaeology of the Eurasian Steppes), Т. 2. Казань: ИИ АН РТ, с. 75–86.
88. **Matveeva G., 2018a:** Матвеева Г.И. 2018a. О миграциях из Западной Сибири в Европу в раннем железном веке и в эпоху Великого переселения народов (On Migrations from Western Siberia to Europe in the Early Iron Age and the Era of the Great Migration of Peoples). *Археология Евразийских степей* (Archaeology of the Eurasian Steppes), №6.
89. **Matveeva N., 1993:** Матвеева, Н. П. Саргатская культура на Среднем Тоболе (The Sargatka culture in the Middle Tobol region). Новосибирск 1993.
90. **Matveeva N., 2000:** Матвеева, Н.П. Социально-экономические структуры населения Западной Сибири в раннем железном веке (Social and economic structures of the population of Western Siberia in the Early Iron Age). Новосибирск 2000.
91. **Matveeva N., 2016:** Матвеева, Н.П. Западная Сибирь в эпоху Великого переселения народов (проблемы культурогенеза в лесостепной и подтаежной зоне). Тюмень 2016.
92. **Matveeva N., 2018a**: Матвеева, Н.П. 2018. О термине «историко-культурный горизонт» как таксономической категориив классификации древностей раннего железного века Урала и Зауралья (On the Term «Historical-cultural Horizon» as a Taxonomical Categoryin the Uralian and Trans-Uralian Antiques Classification). В: Мультидисциплинарные аспекты изучения древней и средневековой истории: К 70-летию акад. В.И. Молодина. Новосибирск: Изд-во ИАЭТ СО РАН. С. 210-219. DOI: 10.17746/0284-1.2018.210–221

93. **Matveeva N., 2018b:** Матвеева Н.П. 2018. Саргатская и гороховская культуры: исторические судьбы. Ранний железный век западносибирской лесостепи по археологическим данным / A szargatkai és gorohovói kultúra történeti interpretációja. A korai vaskor Nyugat-Szibéria erdős sztyeppi területén a régészeti adatok alapján (Sargatka and Gorokhov Cultures: Historical Interpretations. The Early Iron Age of the Western Siberian Forest-Steppe Based on Archaeological Data). III-й Международный мадьярский симпозиум по археологии. Будапешт, 6–10 июня 2016 г. / 3. Nemzetközi Korai Magyar Történeti és Régészeti Konferencia. Budapest, 2016. június 6–10. *MŐT Kiadványok 6*, 15–34. DOI: 10.55722/Arpad.Kiad.2018.6_02.
94. **Matveeva N., 2019:** Матвеева, Н.П. 2019. Гороховская культура в системе трансуральских древностей раннего железного века (The Gorokhovo Culture in the System of the Trans-Ural Antiquities of the Early Iron Age). *Российская археология*. (1), с. 4–19. DOI: 10.31857/S086960630004109-8.
95. **Matveeva N., 2021:** Матвеева Н.П. 2021. К вопросу о контактах урало-западносибирского населения со Средней Азией в раннем Средневековье (по материалам керамических коллекций) (On the Contacts between the Uralo-West Siberian Population and Central Asia in the Early Middle Ages: Based on Ceramic Collections). *Археология, этнография и антропология Евразии* (Archaeology, Ethnography, and Anthropology of Eurasia), Т. 49, № 1, с. 83–92.
96. **Mazhitov, 1963:** Мажитов Н.А. 1963. Бахмутинская культура (Население Северной Башкирии в середине I тысячелетия нашей эры) (Bakhmutino Culture: The Population of Northern Bashkiria in the Mid-1st Millennium AD). Автореферат диссертации на соискание ученой степени кандидата исторических наук. Москва.
97. **Mazhitov, 1968:** Мажитов Н.А. 1968. Бахмутинская культура. Этническая история населения Северной Башкирии середины I тысячелетия нашей эры (Bakhmutino Culture: Ethnic History of the Population of Northern Bashkiria in the Mid-1st Millennium AD). Москва.
98. **Mazhitov, 1977:** Мажитов Н.А. 1977. Южный Урал в VII–XIV вв. (The Southern Urals in the 7th–14th Centuries). Москва: Наука.
99. **Mazhitov, 1981a:** Мажитов Н.А. 1981. Курганы Южного Урала VIII-XII вв. (Barrows of the Southern Urals from the 8th to the 12th Centuries). Москва: Наука.
100. **Mazhitov, 1981b:** Мажитов, Н.А. 1981. Караякуповская культура (Karayakupovo Culture). В: *Археология СССР: Степи Евразии в эпоху средневековья* (Archaeology of the USSR: The Steppes of Eurasia in the Medieval Period). Москва, с. 27–28.
101. **Mogilnikov, 1987:** Могильников В.А. 1987. Угры и самодийцы Урала и Западной Сибири (Ugrians and Samoyeds of the Urals and Western Siberia). В: *Археология СССР. Финно-угры и балты в эпоху средневековья* (Archaeology of the USSR. Finno-Ugric Peoples and Balts in the Medieval Period). Москва, с. 145–156.
102. **Novokreshchennykh, 1914:** Новокрещенных Н.Н. 1914. Гляденовское костище (Glyadenovo Burial Site). В: *Труды Пермской ученой архивной комиссии* (Works of the Perm Scientific Archive Commission), Выпуск XI. Пермь, с. 19–97.
103. **Ostanina, 1983:** Останина Т.И. 1983. Мазунинская культура в Среднем Прикамье (Mazuninsk Culture in the Middle Kama Region). Автореферат диссертации на соискание ученой степени кандидата исторических наук: 07.00.06. Москва.

104. **Ostanina, 1997:** Останина Т.И. 1997. Население Среднего Прикамья в III–V вв. н. э. (The Population of the Middle Kama Region in the 3rd–5th Centuries AD). Ижевск: Издательство Института истории, языка и литературы Уральского отделения РАН.
105. **Ostanina, 1998:** Останина Т.И**.** 1998. Население Среднего Прикамья в III–V вв. (The Population of the Middle Kama Region in the 3rd–5th Centuries). Научный доклад на соискание ученой степени доктора исторических наук: 07.00.06. Москва.
106. **Ovsyannikov, Sungatov, 2004:** Овсянников В.В., Сунгатов Ф.А. 2004. Городище Каменная гора в среднем течении р. Уфы (Kamennaya Gora Hillfort in the Middle Course of the Ufa River). В: *УАВ* , Выпуск 5. Уфа, с. 218–240.
107. **Pastushenko, 2005:** Пастушенко И.Ю., 2005. К вопросу о чияликских «погребальных масках» (To the question of Chiyalik burial masks) *Finno-Ugrica* (Казань). №7-8. С. 36-42.
108. **Pastushenko, 2006:** Пастушенко И.Ю., 2006. Кишертский могильник в бассейне реки Сылвы (Kishert burial site in the basin of the Sylva River) *Finno-Ugrica* (Казань). №9. С. 40-80.
109. **Pastushenko, 2007:** Пастушенко И.Ю**.** 2007. Роль населения средней Камы в генезисе неволинской культуры (The Role of the Population of the Middle Kama in the Genesis of the Nevolino Culture). Пермские финны: археологические культуры и этносы (The Perm Finns: Archaeological Cultures and Ethnic Groups). Ответственный редактор Э.А. Савельева. Сыктывкар: Республиканское издательское объединение Института языка, литературы и истории Коми научного центра Уральского отделения РАН. С. 200-201.
110. **Patrushev, 1984:** Патрушев В.С. 1984. Марийский край в VII–VI вв. до н. э. (The Mari Region in the 7th–6th Centuries BC). Йошкар-Ола: Марийское книжное издательство.
111. **Poshekhonova, 2010:** Пошехонова О.Е. 2010. Краниологические особенности средневековых популяций Сургутского Приобья (по материалам могильников с Барсовой Горы) (Cranial Characteristics of Medieval Populations of the Surgut Ob Region Based on Burial Sites from Barsovaya Hill). *Вестник археологии, антропологии и этнографии* (Herald of Archaeology, Anthropology, and Ethnography). Тюмень: Издательство Института проблем освоения Севера Сибирского отделения РАН. Выпуск 2 (13). С. 91-97.
112. **Poshekhonova, 2011:** Пошехонова О.Е**.**, 2011. Антропологическая характеристика населения южно-таёжного Прииртышья (по материалам могильников усть-ишимской археологической культуры рубежа I и II тыс. н.э.) (Anthropological characteristics of the population of the southern taiga of the Irtysh region (based on the burial sites of the Ust-Ishim archaeological culture of the 1st-2nd millennium AD)) *Археология, этнография и антропология Евразии* (Archaeology, Ethnography and Anthropology of Eurasia). Новосибирск. № 4 (48). С. 142-155.
113. **Poshekhonova, Sleptsova, 2017:** Пошехонова О.Е., Слепцова А.В**.** 2017. Население Нижнего Притоболья в переходное время от раннего железного века к средневековью по данным краниологии (The Population of the Lower Tobol Region in the Transitional Period from the Early Iron Age to the Middle Ages Based on Craniological Data). *Вестник археологии, антропологии и этнографии* (Herald of Archaeology, Anthropology, and Ethnography). Тюмень: Издательство Института

проблем освоения Севера Сибирского отделения РАН. Выпуск 4 (39). С. 90-103.
114. **Savelyev, 2007:** Савельев Н.С. 2007. Месягутовская лесостепь в эпоху раннего железа (The Misyagutov Forest-Steppe in the Early Iron Age). Уфа.
115. **Semenov, 1980:** Семенов В.А. 1980. Варнинский могильник (The Varninsky Burial Ground). *Новый памятник поломской культуры* (A New Site of the Polom Culture). Ижевск. С. 5-135.
116. **Semenov, 1989:** Семенов В.А**.** 1989. Этнокультурные компоненты поломской культуры (Ethnocultural Components of the Polom Culture). *Новые исследования по этногенезу удмуртов* (New Studies on the Ethnogenesis of the Udmurts). Ижевск. С.20-33.
117. **Sharapova 2020:** Шарапова С.В., 2020. Верхняя хронологическая граница саргатской культуры (The Upper Chronological Boundary of the Sargatka Culture). *Нижневолжский археологический вестник*. Т. 19, № 2. С. 218–246. DOI: 10.15688/nav.jvolsu.2020.2.12
118. **Sharapova, Malashev 2022:** Шарапова С.В., Малашев В.Ю., 2022. Хроноиндикаторы I–III вв. н.э. из лесостепных памятников Тоболо-Иртышья (Chronoindicators of the 1st–3rd Centuries CE from Forest-Steppe Sites of the Tobol–Irtysh Region). *Нижневолжский археологический вестник*. Т. 21, № 1. С. 171–192. DOI: 10.15688/nav.jvolsu.2022.1.9
119. **Shutova, 1999:** Шутова Н.И**.** 1999. Женская одежда средневекового населения бассейна Чепцы (по данным раскопок Варнинского могильника 1990-1991 гг.) (Women's Clothing of the Medieval Population of the Cheptsa Basin Based on Excavations of the Varninsky Burial Ground, 1990–1991). *Пермский мир в раннем средневековье / Archaeologia Permica* (The Perm World in the Early Middle Ages / Archaeologia Permica). Выпуск 1. Ижевск. С. 210-231.
120. **Skandakov, Danchenko, 1999:** Скандаков И.Е., Данченко Е.М. 1999. Курганный могильник Усть-Тара VII в южно-таежном Прииртышье (The Ust-Tara Barrow Cemetery of the 7th Century in the Southern Taiga Priirtysh Region). *Гуманитарное знание. Сер. Преемственность: Ежегодн.* Омск: ОмГПУ. Вып. 3. С. 160–186.
121. **Spitsyn, 1893:** Спицын А.А. 1893. Приуральский край. Археологические розыскания о древнейших обитателях Вятской губернии (The Ural Region. Archaeological Investigations of the Earliest Inhabitants of the Vyatka Province). *МАВГР*. М. Вып. I.
122. **Swartz, 1967.** Swartz, B.K. 1967. *A Logical Sequence of Archaeological Objectives.* American Antiquity, Vol. 32, No. 4, pp. 487–497.
123. **Szeifert et al, 2022**: Szeifert, B., Gerber, D., Csáky, V., Langó, P., Stashenkov, D.A., Khokhlov, A., Sitdikov, A., Gazimzyanov, I., Volkova, E.V., Matveeva, N.P., et al. (2022). Tracing genetic connections of ancient Hungarians to the 6th–14th century populations of the Volga-Ural region. Human Molecular Genetics *31*, 3266–3280. https://doi.org/10.1093/hmg/ddac106.
124. **Taylor, 1948:** Taylor, W.W. 1948. *A Study of Archaeology.* Philadelphia: American Anthropological Association. 256 p., 2 ill. – *(American Anthropologist; Vol. 50, No. 3, Part 2; Memoir No. 69),* p. 22–256.
125. **Türk, et al, 2015:** Türk A., Lőrinczy G., Marcsik A. 2015. Régészeti és természettudományi adatok a Maros-torkolat nyugati oldalának 10. századi történetéhez (Archaeological data and scientific findings on the 10th century history of the western bank of the Muresh estuary). *Studia ad Archaeologiam Pazmaniensia* 4.

MTA BTK Magyar Őstörténeti Témacsoport Kiadványok 4. Budapest.

126. **Vasyutkin, 1968:** Васюткин, С.М. 1968. Некоторые спорные вопросы археологии Башкирии I тысячелетия н.э. (Some Controversial Questions of Bashkiria Archaeology of the 1st Millennium AD). *Советская археология*, № 1, С. 69–71.

127. **Vasyutkin, 1992:** Васюткин, С.М. 1992. О происхождении и этнической принадлежности населения кушнаренковской культуры (On the Origin and Ethnic Affiliation of the Kushnarenkovo Culture Population). В: *Проблемы этногенеза финно-угорских народов Приуралья*. Ижевск.

128. **Vyazov et al., 2019:** Vyazov, L.A., Ershova, E.G., Ponomarenko, E.V., et al. 2019. Demographic Changes, Trade Routes, and the Formation of Anthropogenic Landscapes in the Middle Volga Region in the Past 2500 Years. In: Yang, L.E., et al. (eds.). *Socio-Environmental Dynamics along the Historical Silk Road.* Springer International Publishing, pp. 425–465. DOI: 10.1007/978-3-030-00728-7_19.

129. **Yutina, 2007:** Ютина Т.К. 2007. Исследования Дербёшкинского могильника в Актанышском районе Республики Татарстан (Research of the Derbyoshkinsky Burial Ground in the Aktanysh District of the Republic of Tatarstan). *Finno-Ugrica* (Kazan). № 10. С. 73-98.

130. **Zbrueva, 1952:** Збруева А.В. 1952. История населения Прикамья в ананьинскую эпоху (The History of the Population of the Kama Region in the Ananyino Era). *Материалы и исследования по археологии (МИА)*, № 30. Москва: АН СССР.

131. **Zelenkov, Tretyakov 2025**: Зеленков А. С., Третьяков Е. А. Радиоуглеродная хронология средневековых памятников лесостепи и подтайги Тоболо-Иртышья (Radiocarbon Chronology of Medieval Sites in the Forest-Steppe and Subtaiga Zones of the Tobol–Irtysh Region). *Вестник НГУ. Серия: История, филологи*я. 2025. Т. 24, № 5: Археология и этнография. С. 94–112. DOI 10.25205/1818-7919-2025-24-5-94-112
