# Supplementary figures and images for: Long shared haplotypes identify the Southern Urals as a primary source for the 10th century Hungarians
Source: Cell. Author manuscript; Available in PMC 2025 Dec 17. (PMC12711333; doi:10.1016/j.cell.2025.09.002)

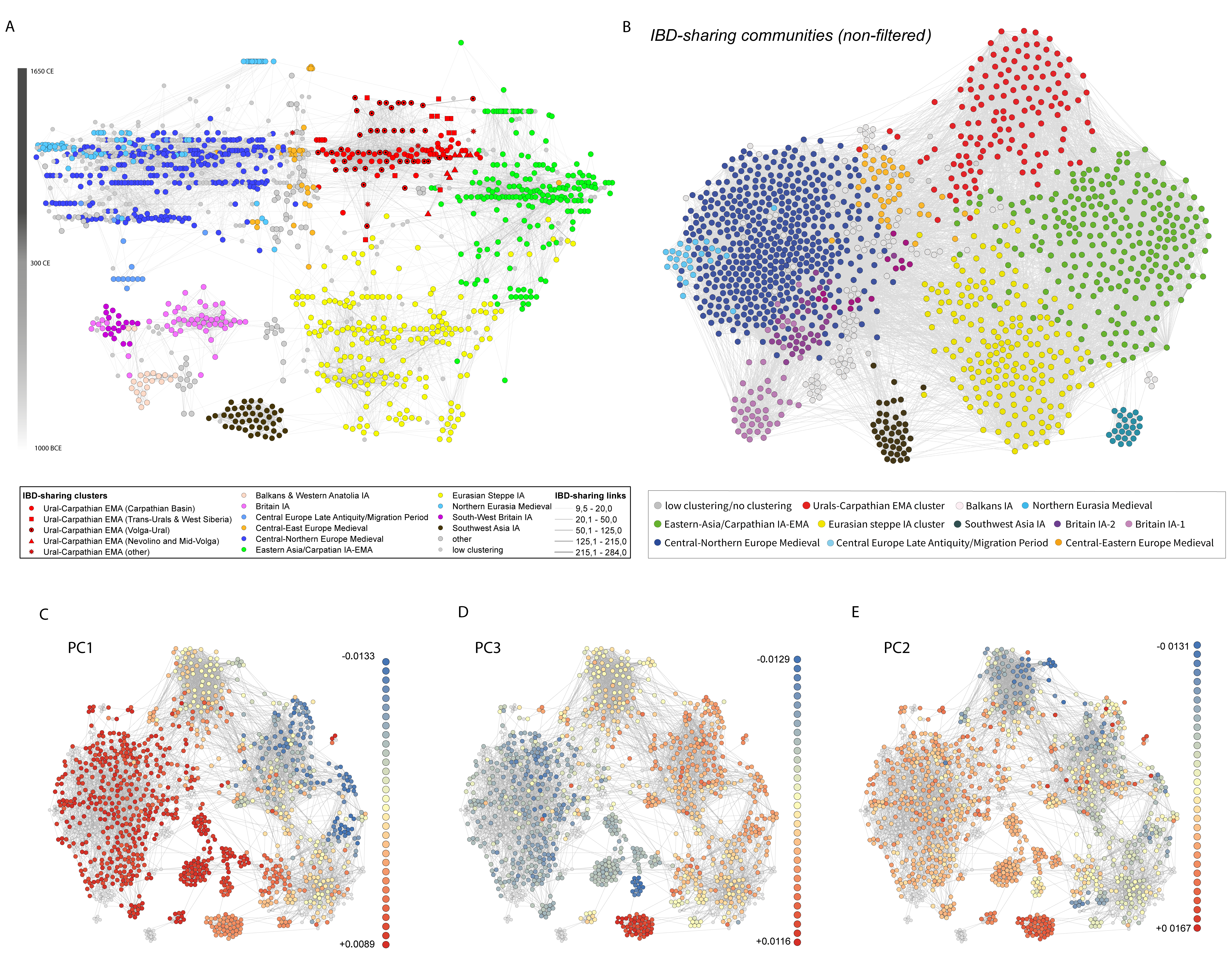

Supplement: 2 [file NIHMS2116457-supplement-2.tif]

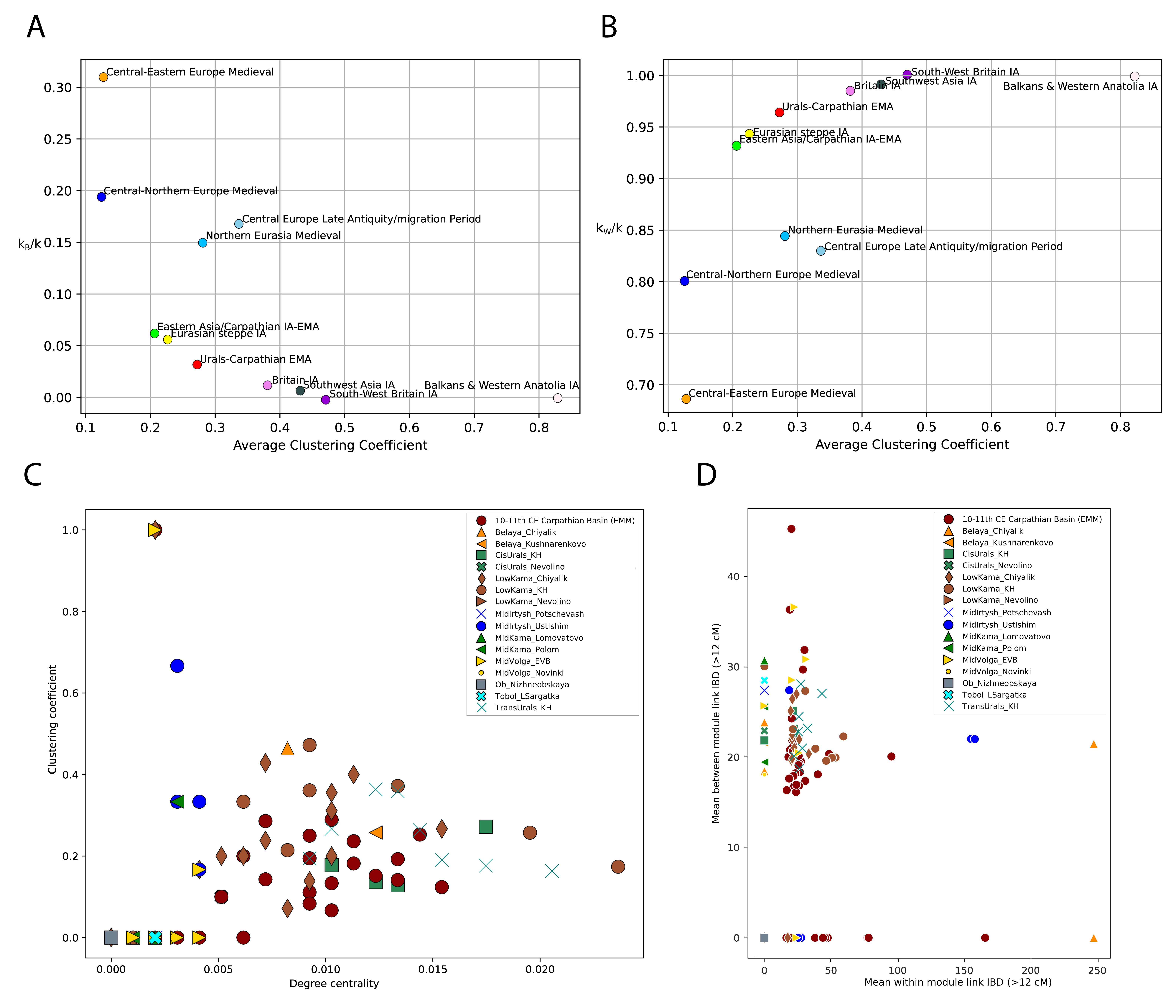

Supplement: 3 [file NIHMS2116457-supplement-3.tif]

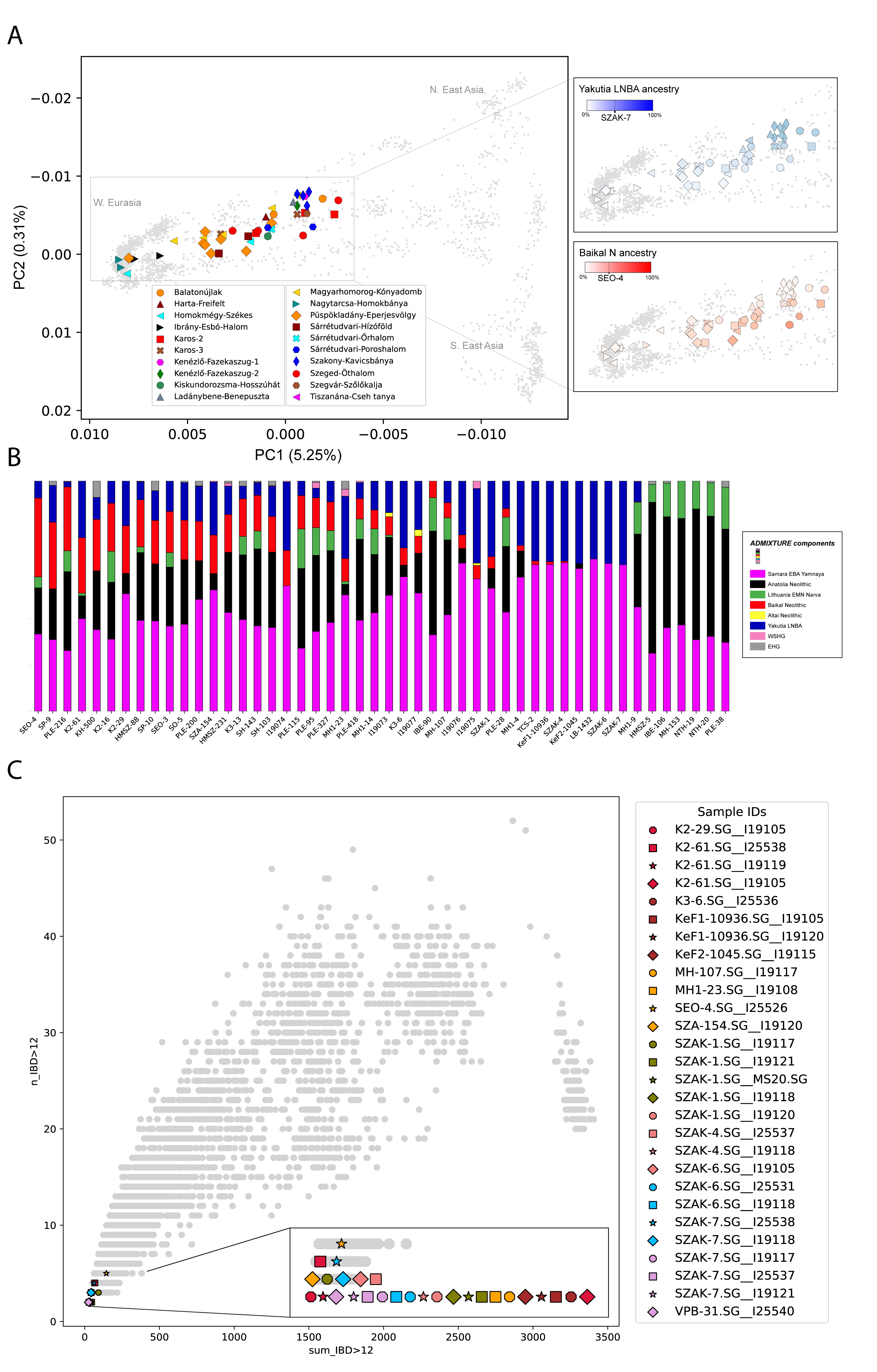

Supplement: 4 [file NIHMS2116457-supplement-4.tif]

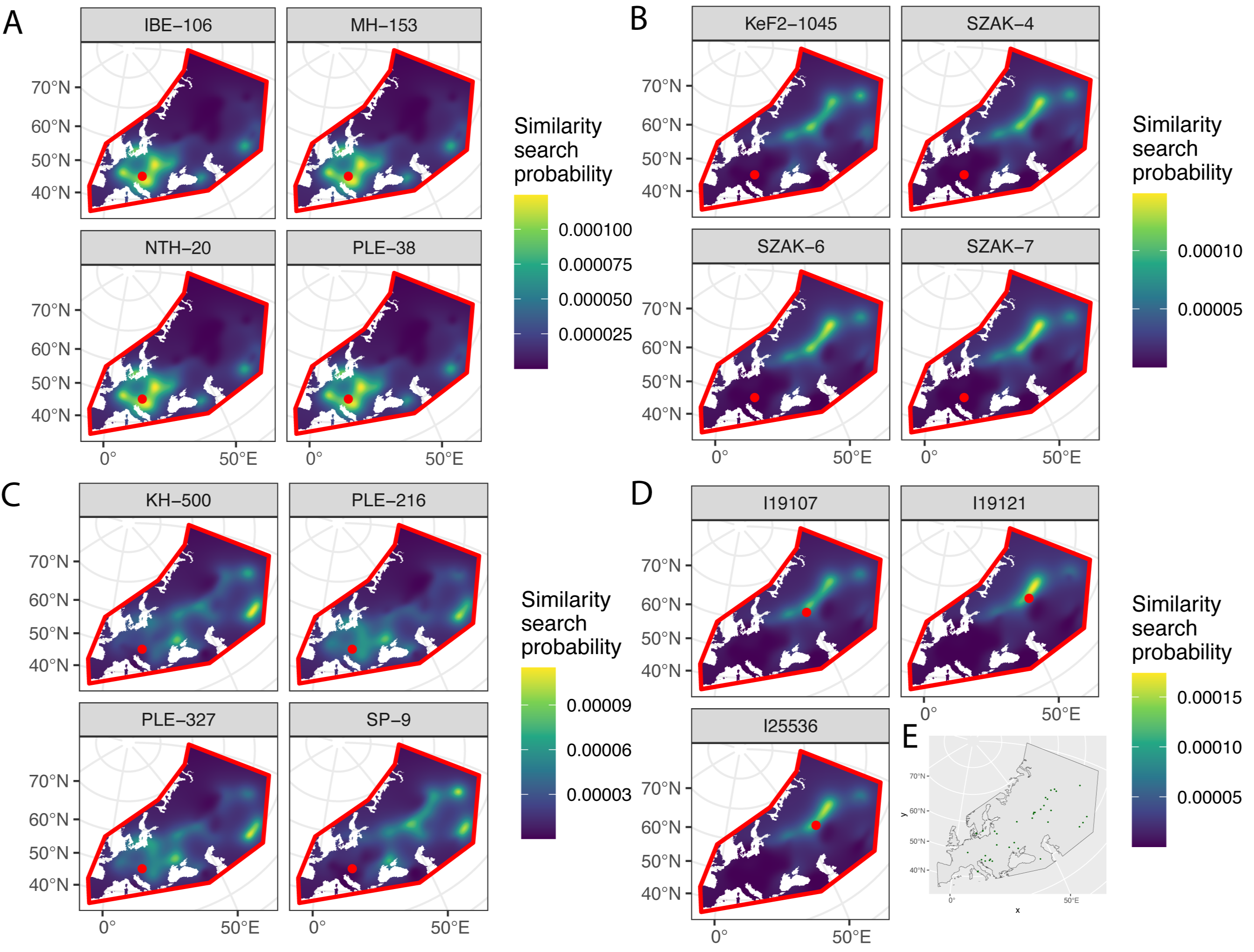

Supplement: 5 [file NIHMS2116457-supplement-5.tif]

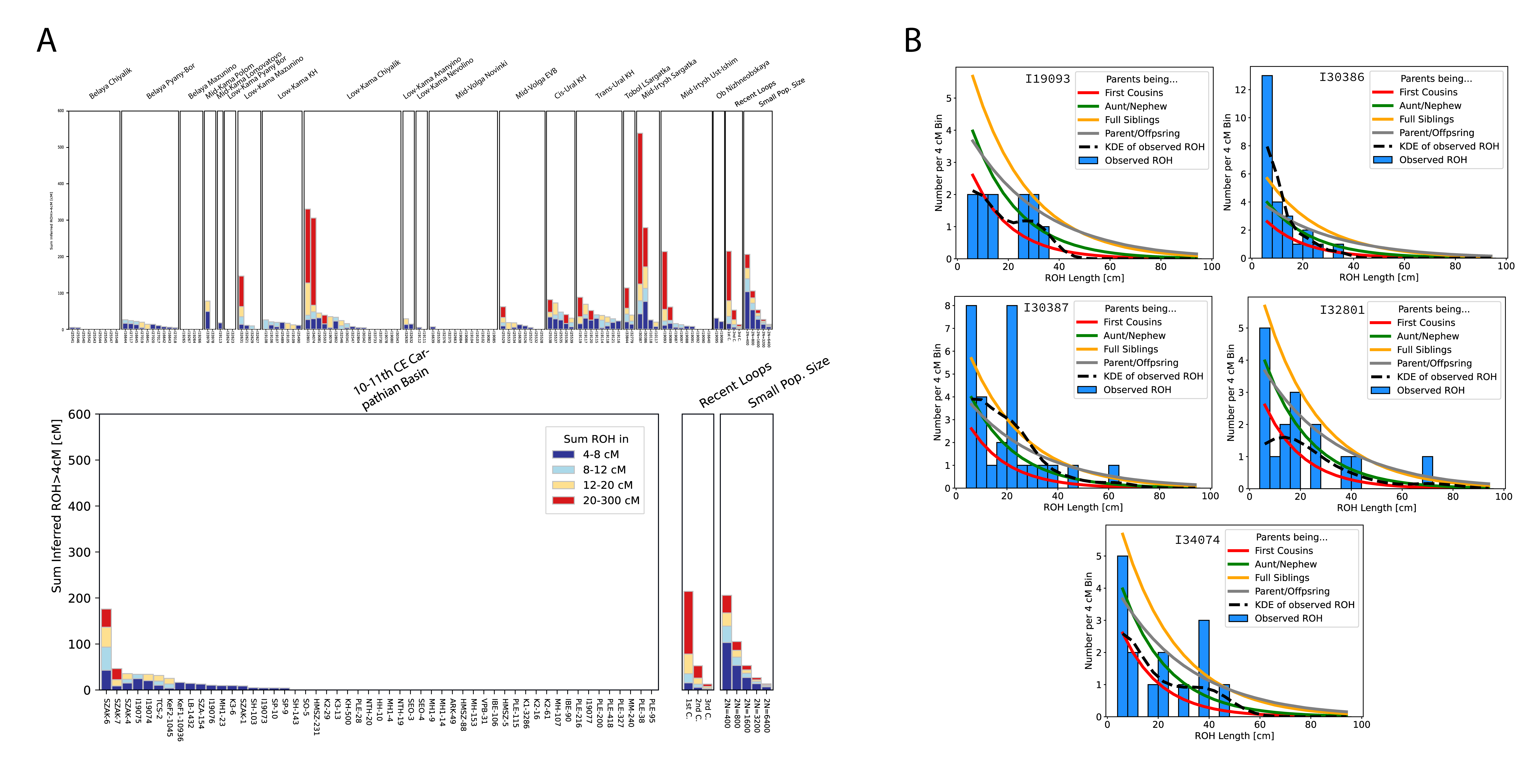

Supplement: 6 [file NIHMS2116457-supplement-6.tif]
