# Supplementary material for: Long shared haplotypes identify the Southern Urals as a primary source for the 10th century Hungarians
Source: Cell. Author manuscript; Available in PMC 2025 Dec 17. (PMC12711333; doi:10.1016/j.cell.2025.09.002)
Supplement: 7 [file NIHMS2116457-supplement-7.pdf]

GroupID1	*Ind1*	*Date1*	**Sex**	**Y/mtDNA**	*GroupID2*	*Ind2*	**Sex**	**Y/mtDNA**	*Date2*	***total length of shared IBD segments >2 x 12 cM***
10-11th century CB (EMM)	SEO-4	900-1000 CE	male	G2a/T2g1a	Mid-Volga EVB	I25526	male	Q/B5b4	850-1050 CE	144
10-11th century CB (EMM)	SZAK-1	900-1000 CE	male	N1a/T2d1b 1	Trans-Urals KH	I19117	male	N1a/N1a	***771-937 calCE***	92
10-11th century CB (EMM)	K2-61	900-950 CE	male	R1a/U4d2	Cis-Urals KH	I25538	male	N1a/U5a1g 1	664-1016 CE	67
10-11th century CB (EMM)	SZAK-7	900-1000 CE	female	-/D5a1	Trans-Urals KH	I19118	male	G2a/A+15 2	772-1152 CE	42
10-11th century CB (EMM)	SZAK-7	900-1000 CE	female	-/D5a1	Cis-Urals KH	I25538	male	N1a/U5a1g 1	664-1016 CE	63
10-11th century CB (EMM)	SZAK-4	900-1000 CE	female	-/HV4a2a	Cis-Urals KH	I25537	male	N1a/H6a1b	664-1016 CE	43
10-11th century CB (EMM)	SZA-154	900-1000 CE	female	-/B5b4	Trans-Urals KH	I19120	male	N1a/A12a	772-1152 CE	42
10-11th century CB (EMM)	SZAK-6	900-1000 CE	female	-/A16	Low-Kama KH	I19105	female	-/A12a	850-950 CE	45
10-11th century CB (EMM)	SZAK-1	900-1000 CE	male	N1a/T2d1b 1	Trans-Urals KH	I19121	male	N1a/U5a1a 1	***879-1150 calCE***	46
10-11th century CB (EMM)	K3-6	900-1000 CE	female	-/B4d1	Cis-Urals KH	I25536	male	N1a/C4a2	***664-827 calCE***	46
10-11th century CB (EMM)	SZAK-1	900-1000 CE	male	N1a/T2d1b 1	Trans-Urals KH	MS20(Gerber et al.)	male	N1a/D4j	772-1152 CE	45
10-11th century CB (EMM)	K2-61	900-950 CE	male	R1a/U4d2	Trans-Urals KH	I19119	male	N1a/C4a1	772-1152 CE	39
10-11th century CB (EMM)	SZAK-4	900-1000 CE	female	-/HV4a2a	Trans-Urals KH	I19118	male	G2a/A+15 2	772-1152 CE	37
10-11th century CB (EMM)	KeF2-1045	900-1000 CE	male	N1a/N1a	Trans-Urals KH	I19115	male	N1a/H40b	772-1152 CE	32
10-11th century CB (EMM)	SZAK-6	900-1000 CE	female	-/A16	Trans-Urals KH	I19118	male	G2a/A+15 2	772-1152 CE	31
10-11th century CB (EMM)	SZAK-1	900-1000 CE	male	N1a/T2d1b 1	Trans-Urals KH	I19118	male	G2a/A+15 2	772-1152 CE	29

10-11th century CB (EMM)	SZAK-7	900-1000 CE	female	-/D5a1	Cis-Urals KH	I25537	male	N1a/H6a1b	664-1016 CE	29
10-11th century CB (EMM)	KeF1-10936	900-1000 CE	male	Q1a/C4b	Low-Kama KH	I19105	female	-/A12a	850-950 CE	29
10-11th century CB (EMM)	KeF1-10936	900-1000 CE	male	Q1a/C4b	Trans-Urals KH	I19120	male	N1a/A12a	772-1152 CE	27
10-11th century CB (EMM)	SZAK-7	900-1000 CE	female	-/D5a1	Trans-Urals KH	I19117	male	N1a/N1a	***771-937 calCE***	38
10-11th century CB (EMM)	SZAK-6	900-1000 CE	female	-/A16	Belaya Kushnarenkovo	I25531	male	G2a/U2e1	***340-540 calCE***	37
10-11th century CB (EMM)	K2-61	900-950 CE	male	R1a/U4d2	Low-Kama KH	I19105	female	-/A12a	850-950 CE	35
10-11th century CB (EMM)	19076	900-1000 CE	male	G2a/H15a1	Low-Kama KH	I19105	female	-/A12a	850-950 CE	34
10-11th century CB (EMM)	K2-29	900-1000 CE	male	N1a/J1b	Low-Kama KH	I19105	female	-/A12a	850-950 CE	33
10-11th century CB (EMM)	MH1-23	900-1000 CE	male	D1a/N1a	Low-Kama KH	I19108	male	I1a/T1a1	850-950 CE	32
10-11th century CB (EMM)	MH-107	1000-1100 CE	female	-/H11a7	Trans-Urals KH	I19117	male	N1a/N1a	***771-937 calCE***	29
10-11th century CB (EMM)	I19075	900-1000 CE	female	-/U5a1a1	Low-Kama KH	I19106	male	R1a1a1/D4g1b	850-950 CE	29
10-11th century CB (EMM)	SZAK-7	900-1000 CE	female	-/D5a1	Trans-Urals KH	I19121	male	N1a/U5a1a1	***879-1150 calCE***	29
10-11th century CB (EMM)	SZAK-1	900-1000 CE	male	N1a/T2d1b1	Trans-Urals KH	I19120	male	N1a/A12a	772-1152 CE	26
10-11th century CB (EMM)	VPB-31	700-800 CE	female	-/HV10	Belaya Chiyalik	I25540	male	J2a/N1a	***1281-1395 calCE***	26

Table S2: Extended table of IBD connections between 10-11th century Carpathian Basin and Volga-Ural Early Medieval individuals with at least two IBD segments longer than 12 cM, related to Table 1.

10-11th century CB (EMM)	***source group***	***p-value***
SZAK-4	Russia_TransUrals_KH	0.939
SZAK-4	Russia_CisUrals_KH	0.932
SZAK-7	Russia_CisUrals_KH	0.882
SZAK-7	Russia_TransUrals_KH	0.811

MH1-23	Russia_CisUrals_KH	0.783
SZAK-6	Russia_CisUrals_KH	0.648
SZAK-1	Russia_CisUrals_KH	0.635
MH1-23	Russia_TransUrals_KH	0.594
I19075	Russia_TransUrals_KH	0.556
SZAK-1	Russia_TransUrals_KH	0.303
SZAK-6	Russia_TransUrals_KH	0.263
I19075	Russia_CisUrals_KH	0.254
KeF2-1045	Russia_CisUrals_KH	0.173
KeF2-1045	Russia_TransUrals_KH	0.116
PLE-200	Russia_LowKama_KH	0.109
TCS-2	Russia_TransUrals_KH	0.105
LB-1432	Russia_CisUrals_KH	0.100
LB-1432	Russia_TransUrals_KH	0.099
MH1-4	Russia_LowKama_KH	0.078
SP-10	Russia_LowKama_KH	0.074
K3-6	Russia_CisUrals_KH	0.068
I19076	Russia_LowKama_KH	0.060
KeF1-10936	Russia_CisUrals_KH	0.052

Table S3: Cladality test of the 10-11th century Carpathian Basin individuals with a feasible model (p-value>0.05) from the *Urals-Carpathian EMA* cluster, related to Table 1.

	N_e estimate	std.error	0.025 CI	0.975 CI
Belaya Pyany Bor	2,500	576	1,370	3,630
Trans-Urals KH	1,022	161	705	1,339
MidIrtysh Ust-Ishim	3,365	1,018	1,368	5,350
Low Kama Chiyalik	2,934	567	1,822	4,046
Low Kama KH	2,307	580	1,170	3,444
MidVolga EVB	3,252	905	1,470	5,050
MidVolga Novinki	69,000	69,000	-66,000	204,000

Table S4: Effective population sizes (N_e) for groups with at least 8 individuals estimated with *hapROH Ne vignette, considering 4-20 cM segments*, related to Figure 4.
